# Balanced CoQ_6_ biosynthesis is required for lifespan and mitophagy in yeast

**DOI:** 10.15698/mic2017.02.556

**Published:** 2017-02-03

**Authors:** Isabel González-Mariscal, Aléjandro Martín-Montalvo, Cristina Ojeda-González, Adolfo Rodríguez-Eguren, Purificación Gutiérrez-Ríos, Plácido Navas, Carlos Santos-Ocaña

**Affiliations:** 1 Centro Andaluz de Biología del Desarrollo, Universidad Pablo de Olavide-CSIC, CIBERER Instituto de Salud Carlos III, Sevilla, 41013, Spain.

**Keywords:** coenzyme Q6, regulation, mitochondria, yeast, mitophagy, chronological life span

## Abstract

Coenzyme Q is an essential lipid with redox capacity that is present in all organisms. In yeast its biosynthesis depends on a multiprotein complex in which Coq7 protein has both catalytic and regulatory functions. Coq7 modulates CoQ_6_ levels through a phosphorylation cycle, where dephosphorylation of three amino acids (Ser/Thr) by the mitochondrial phosphatase Ptc7 increases the levels of CoQ_6_. Here we analyzed the role of Ptc7 and the phosphorylation state of Coq7 in yeast mitochondrial function. The conversion of the three Ser/Thr to alanine led to a permanently active form of Coq7 that caused a 2.5-fold increase of CoQ_6_ levels, albeit decreased mitochondrial respiratory chain activity and oxidative stress resistance capacity. This resulted in an increase in endogenous ROS production and shortened the chronological life span (CLS) compared to wild type. The null *PTC7* mutant (*ptc7*∆) strain showed a lower biosynthesis rate of CoQ_6_ and a significant shortening of the CLS. The reduced CLS observed in *ptc7*Δ was restored by the overexpression of *PTC7* but not by the addition of exogenous CoQ_6_. Overexpression of *PTC7* increased mitophagy in a wild type strain. This finding suggests an additional Ptc7 function beyond the regulation of CoQ biosynthesis. Genetic disruption of *PTC7* prevented mitophagy activation in conditions of nitrogen deprivation. In brief, we show that, in yeast, Ptc7 modulates the adaptation to respiratory metabolism by dephosphorylating Coq7 to supply newly synthesized CoQ_6_, and by activating mitophagy to remove defective mitochondria at stationary phase, guaranteeing a proper CLS in yeast.

## INTRODUCTION

Coenzyme Q (CoQ) deficiency is a syndrome that belongs to the family of mitochondrial diseases 1. CoQ, a lipid embedded in cell membranes, main function is to act as an electron carrier and as an antioxidant. CoQ deficiency is classified by the observed phenotype as it is a multiple-caused syndrome 2. There are primary and secondary CoQ deficiencies; the deficiency can be a consequence of mutations in genes involved directly in the CoQ biosynthesis (primary deficiency) 3,4 or a consequence of defects or mutations in genes not directly related to CoQ biosynthesis (secondary deficiency) 5,6. The existence of secondary deficiencies introduces the idea of regulatory mechanisms of CoQ biosynthesis that could also be related to a general regulation of mitochondrial metabolism. The main defect caused by CoQ deficiency is a depletion of ATP in tissues. The variety of symptoms and the diversity of CoQ functions introduce a source of complexity in the analysis of CoQ deficiency syndrome that requires the analysis of the mechanisms regulating the biosynthesis of CoQ 7.

In yeast, CoQ_6_ is synthesized in the mitochondria after the condensation of an activated polyisoprenoid tail with a hydroxybenzoic ring; the ring is further modified by several reactions 8. The enzymes that catalyze these reactions are encoded by nuclear *COQ* genes 9. Most of the *COQ* genes encode proteins responsible for enzymatic activity; however, three proteins without enzymatic activity, Coq4, Coq8 and Coq9, play a structural role. Coq4 has been reported to support the assembly of the CoQ_6_ biosynthetic complex in yeast 10. Coq8 has been included in a family of unusual kinases 11, which also includes other proteins involved in CoQ biosynthesis 12. Recent analysis of the function of human ADCK3 protein, homologous of yeast Coq8, showed that the inhibition of ADCK3 kinase activity is required for the activation of CoQ_6_ biosynthesis 13,14. Coq9 is a membrane protein located at the matrix side of the mitochondrial inner membrane and it belongs to the CoQ_6 _biosynthetic complex, where it co-migrates with Coq3 and Coq4 at a molecular mass of approximately 1 MDa 15 and it binds to Coq7 to promote CoQ_6_ biosynthesis 16.

Yeast biosynthesis of CoQ_6_ occurs in a multi-protein complex (Q-synthome). The assembly of the Q-synthome requires the post-translational modification of Coq proteins. Several studies in the last years have demonstrated the existence of the Q-synthome 17,18,19 and several models for the assembly of the complex have been proposed 9,20. The complex assembly starts with a nucleation around the quinone-like lipid polyprenyl benzoate bound to a nucleating Coq protein such as Coq4. The nucleation step is ended with the assembly of a pre-complex that accumulates a CoQ_6_ intermediate, the demethoxy quinone (DMQ_6_) 19,21. DMQ_6_ is converted to CoQ_6_ after the activation of Coq7 by dephosphorylation 22. Coq7 catalyzes the next to last reaction of the pathway 23, the DMQ_6_ hydroxylation. Several studies have reported the existence of phosphoproteins in the family of Coq proteins: Coq3, Coq5 and Coq7 24,25, but only phosphorylation of Coq7 is known to have a physiological relevance 22. Coq7 phosphorylation leads to a low activity state, therefore accumulating DMQ_6_, while its dephosphorylation activates Coq7 and increases CoQ_6_ levels. Both activation states of Coq7 can be achieved by changing the carbon source in the culture media 22. These results were confirmed in *COQ7* null mutants yeast strains (*coq7*∆) expressing either a Coq7 version that is permanently dephosphorylated (Coq7-AAA), associated with a sharp increase of CoQ_6_ concentration, or a permanently phosphorylated version (Coq7-DED) that is associated with a significant decrease of CoQ_6_ levels 22. The Coq7-AAA version was obtained by site-directed mutagenesis of residues S20, S28 and T32 to alanine, while in the Coq7-DED version these residues were mutated to glutamic or aspartic acid. Recent studies have demonstrated the presence of another phosphorylatable residue in Coq7, the S114 26, whose modification affects the catalytic function of Coq7. The activation (i.e. dephosphorylation) of Coq7 is carried out by Ptc7, a phosphatase that belongs to the type 2C Mg^2+^ or Mn^2+^ dependent protein phosphatases, PPM 27,28. Other mitochondrial members of this family (Ptc5) have been related to the activation of pyruvate dehydrogenase (PDH) 29,30 and with the activation of mitophagy (Ptc6) 31,32. Null mutants of *PTC7* gene (*ptc7*∆) have decreased levels of CoQ_6_, decreased mitochondrial respiratory chain activities and decreased resistance to oxidative stress 28. The expression of Coq7-AAA in *ptc7*∆ did not disrupt the high amount of CoQ_6_ produced by this Coq7 version, demonstrating a relationship between Coq7 and Ptc7 28.

Here we investigate the effect of both Coq7 mutants, Coq7-AAA and Coq7-DED on mitochondrial physiology and its relationship with Ptc7 function. Our results indicate that although Coq7 mutants modify the mitochondrial physiology in a similar fashion than *ptc7*∆ relative to CoQ_6 _levels, they have different effects on chronological life span, respiratory complexes interactions and on mitophagy activation.

## RESULTS

### Coq7-phosphorylation mutants show defects in mitochondrial respiratory chain activities

**Figure 1:**
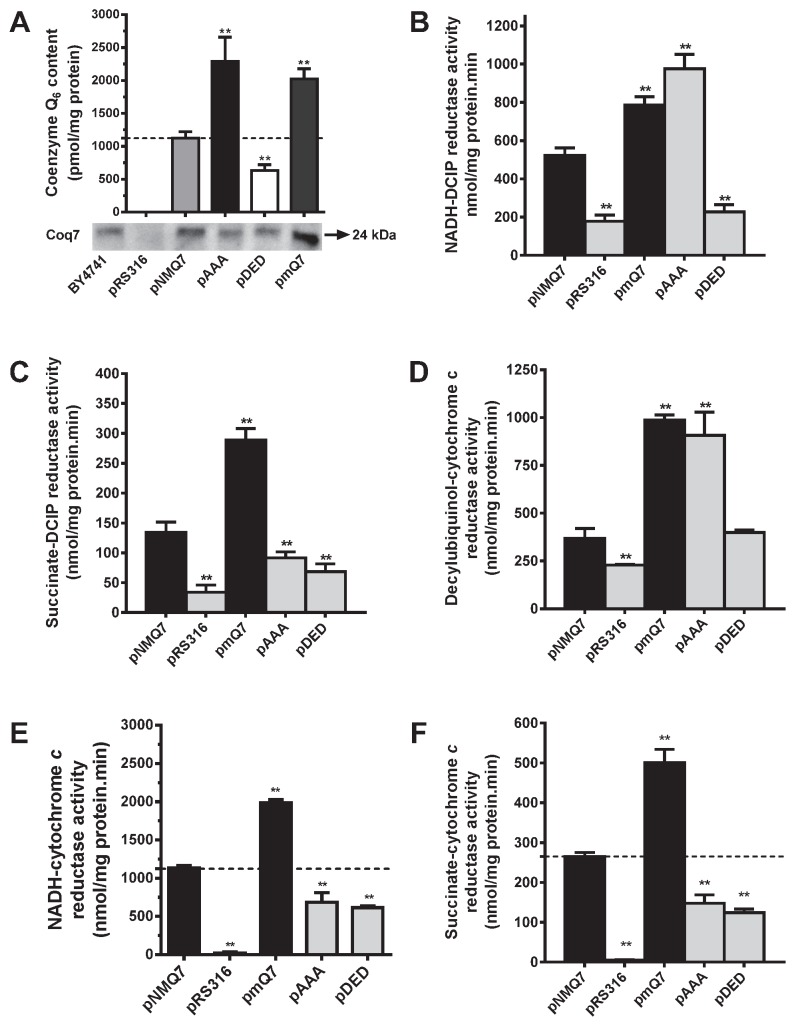
FIGURE 1: CoQ_6_-dependent mitochondrial respiratory chain activities are negatively affected in yeast expressing Coq7 phosphomutant versions. Mitochondrial activities were measured with mitochondria purified from the *coq7*∆ strain harboring the indicated plasmids. pNMQ7 corresponds to the wild type *COQ7* gene, pRS316 corresponds to the empty vector, pAAA and pDED correspond to the Coq7 phosphomutant versions and pmQ7 corresponds to a multicopy expression of wild type *COQ7* gene. **(A)** Top panel: CoQ_6_ quantification, bottom panel Western blots of mitochondrial samples probed with antibodies against Coq7. Mitochondrial activities: **(B)** NADH-DCIP reductase, **(C)** Complex II: succinate-DCIP reductase, **(D)** Complex III: Decylubiquinol-cytochrome *c* reductase, **(E)** NADH-cytochrome *c* reductase and **(F)** Complex II+III: Succinate-cytochrome *c* reductase. Results are expressed as nmol/mg mitochondrial protein^.^min. Data are mean ± SD, N ≥ 3 independent assays. ** P ≤ 0.001 compared to positive control samples.

We measured CoQ_6_ content and the activities of mitochondrial respiratory chain (MRC) in single or coupled complexes (Figure 1). The strain c*oq7*∆ did not contain CoQ_6_, which was rescued in the control strain (*coq7*∆/pNMQ7). The strain expressing permanently dephosphorylated Coq7 (*coq7*∆*/*pAAA) showed a dramatic increase of CoQ_6_, while the strain expressing permanently phosphorylated Coq7 (*coq7*∆*/*pDED) shows a significant decrease of CoQ_6_ compared to control. Multicopy *COQ7* transformed yeast (*coq7*∆*/*pmQ7) also significantly increased CoQ_6_ (Figure 1A). Under the experimental conditions and in all the strains analyzed, Coq7 was detected by western blotting at the expected size of 24 kDa. NADH-Q reductase activity, measured as NADH-DCIP reductase, was decreased in the *coq7*∆*/*pDED strain in a similar manner than negative control (*coq7*∆*/*pRS316), but in the *coq7*∆*/*pAAA strain, the activity was increased over the control, equivalent to the activity measured in the *coq7*∆*/*pmQ7 strain (Figure 1B). Complex II activity measured as succinate-DCIP reductase showed a moderated decrease in both *coq7*∆*/*pDED and *coq7*∆*/*pAAA strains (Figure 1C). However, complex II activity was increased significantly in the *coq7*∆*/*pmQ7 strain. Complex III activity (decylubiquinol-cytochrome *c* reductase) showed changes comparable to those in complex I (Figure 1D). Coupled MRC activities require CoQ_6_ as electron carrier, which is not added exogenously in the assay. Complexes activities such as NADH-cytochrome *c* reductase and succinate-cytochrome *c* reductase (Figures 1E and 1F) were decreased in both *coq7*∆*/*pAAA and *coq7*∆*/*pDED strains compared to control. Interestingly, the decrease in NADH-cytochrome *c* reductase activity in the *coq7*∆*/*pAAA strain does not correlate with the increased activity observed in single complexes (Figure 1B and 1D) and with the high amount of CoQ_6_ found in mitochondria of the *coq7*∆*/*pAAA strain. The activities of these complexes in the *coq7*∆*/*pmQ7 strain were significantly higher than in control.

### Oxidative stress conditions in Coq7 phosphomutants

**Figure 2:**
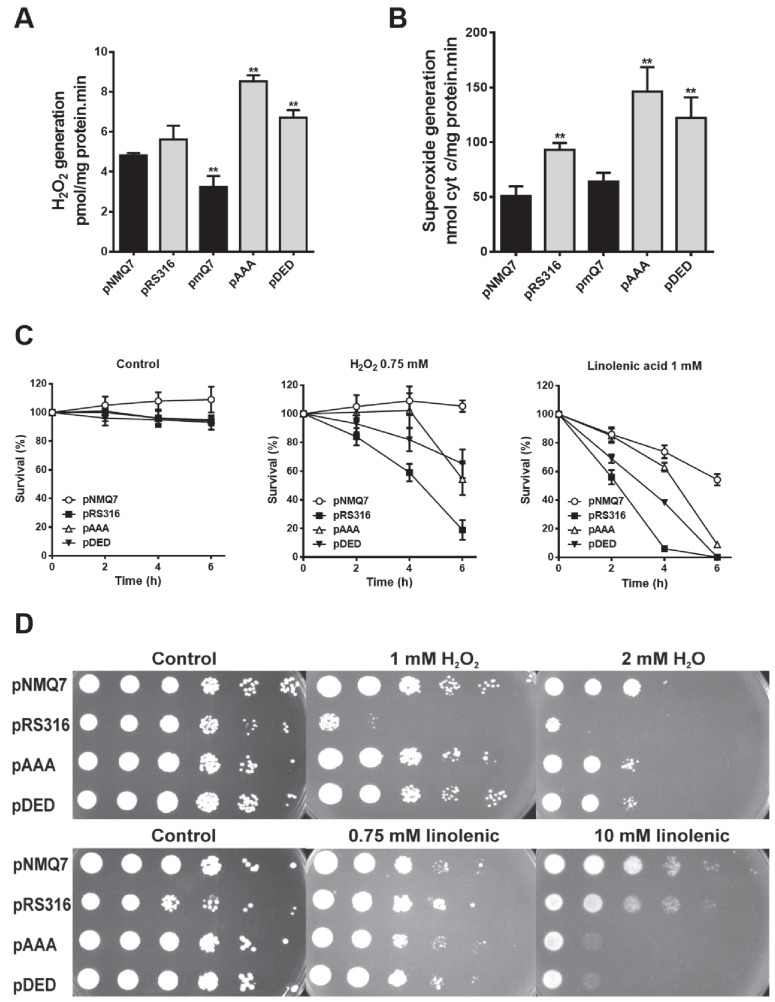
FIGURE 2: Expression of Coq7 phosphomutant versions increases both the endogenous generation and the sensitivity to oxidative stress. **(A)** Quantification of hydrogen peroxide generation was performed in presence of NADH and succinate as electron donors and 50 μg of purified mitochondria from the indicated strains. Data are mean ± SD, N ≥ 3 independent assays. **(B)** Superoxide anion generation was measured using acetylated cytochrome *c* as electron acceptor. Quantification was produced in presence of reduced decylubiquinone as electron donor and 50 μg of purified mitochondria from the indicated strains. Data are mean ± SD, N ≥ 3 independent assays. ** P ≤ 0.001 compared to positive control (*coq7*∆/pNMQ7). **(C) **Oxidative stress sensitivity was measured in the indicated strains. Cells were subjected to oxidative stress treatment by H_2_O_2_ (0.75 mM) or linolenic acid (1 mM) in 0.1 M sodium phosphate buffer plus 0.2 % glucose buffer at 150 x 10^6^ cells/ml. At the indicated times cells were harvested, diluted in sterile water and seeded in triplicate in YPD plates to calculate the number of CFU. The experiment is representative of a set of three. **(D) **Cell viability was monitored under induced oxidative stress. Yeast cells were grown in SDc -ura glucose. After two days, cells were spotted by serial dilutions (1/10) from 0.5 OD_660nm_/ml onto YPD plates containing the indicated concentrations of oxidative stress agents. Plates were incubated at 30°C for 3 days and imaged.

Due to the changes observed in MRC, we analyzed the endogenous oxidative stress, measured as H_2_O_2_ generation in mitochondria, from *coq7*∆ yeast expressing the different versions of Coq7 (Figure 2A). Expression of both Coq7-pAAA and Coq7-pDED showed an increased generation of H_2_O_2_ in mitochondria compared to control, while the *coq7*∆/pmQ7 strain showed a decreased amount, even lower than control. To determine the oxidative stress produced specifically by the complex III, we analyzed the su-peroxide anion generation using reduced decylubiquinone and acetylated cytochrome *c* (Figure 2B) 33. Strains expressing both mutated versions of Coq7 produced significantly higher amounts of superoxide, from 200 to 400%, compared to wild type. Also, superoxide was higher in the *coq7*∆/pRS316 strain. On the contrary, the *coq7*∆/pmQ7 strain showed a superoxide production comparable to control.

In yeast, CoQ_6_ acts as a powerful antioxidant and it is required to protect against lipophilic oxidants such as linolenic acid 34,35,36. The sensitivity to either H_2_O_2_ or linolenic acid was analyzed in a *coq7*∆ strain expressing both Coq7 versions harboring phosphosite modifications (Figures 2C-D). Survival was not compromised in all strains without oxidative stress insult (Figure 2C) but after incubation with H_2_O_2_ or linolenic acid the wild type strain showed the higher survival rate at 2, 4 and 6 hours, while the negative control (*coq7*∆/pRS316) showed a lower survival rate. Interestingly, both treatments in *coq7*∆/pAAA and *coq7*∆/pDED strains produced a similar effect on survival, being higher than negative control but lower than control. A similar effect was found when the assay was performed in agar plates with H_2_O_2 _(Figure 2D); the lack of CoQ_6_ in the *coq7*∆/pRS316 strain compromised the growth but *coq7*∆/pAAA and *coq7*∆/pDED growth was only slightly lower compared to control. When the assay was performed with linolenic acid at higher concentration, both coq7 mutants, *coq7*∆/pAAA and *coq7*∆/pDED, were more sensitive to the treatment, although both retain CoQ_6_ production. That result is surprising since null Coq mutants do not produce CoQ_6_ and show a high sensitivity to linolenic acid 35,36. In contrast, *coq7*∆/pAAA and *coq7*∆/pDED strains synthesize CoQ_6_, with higher production than control for *coq7*∆/pAAA strain.

### Chronological life span is differentially affected in Coq7 phosphomutants 

**Figure 3:**
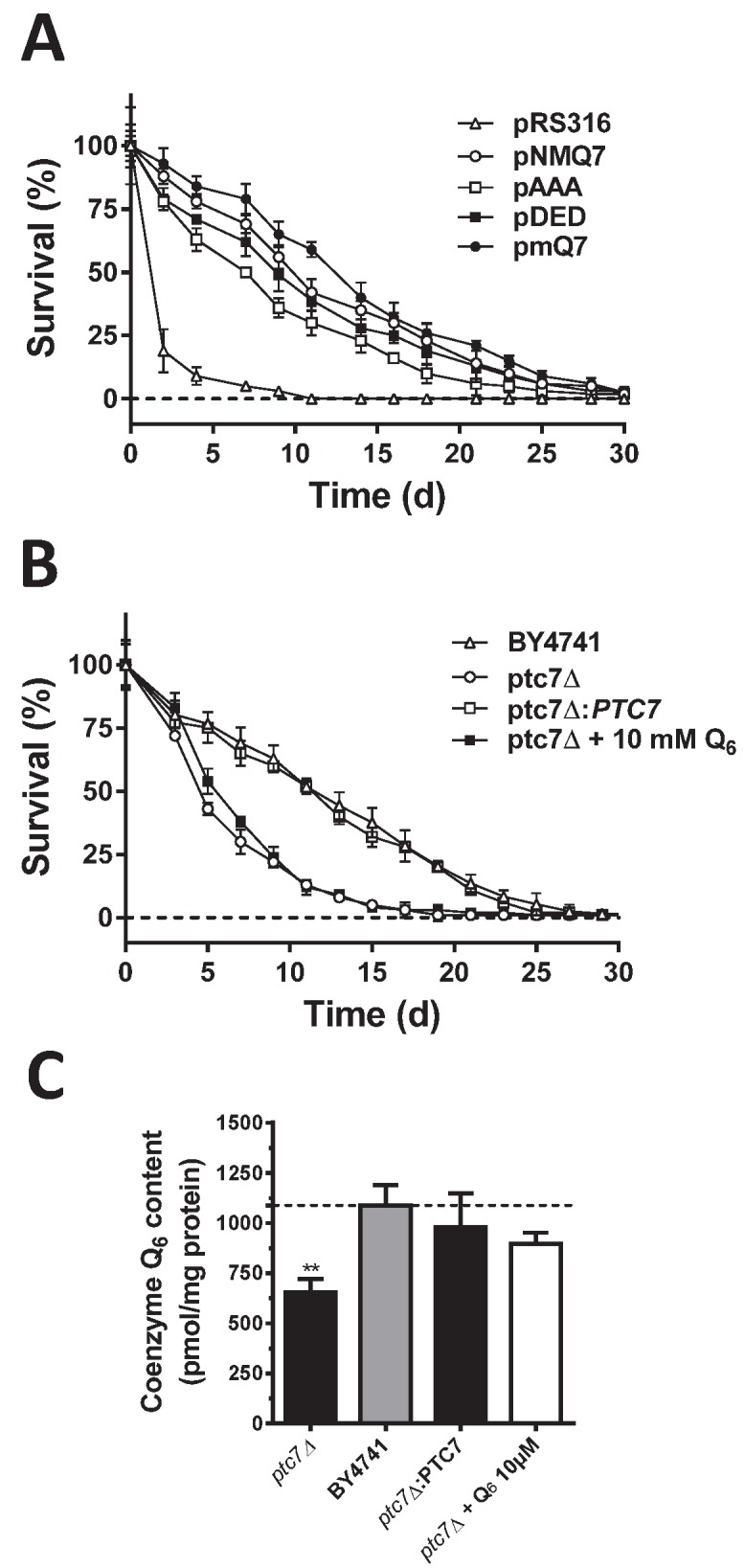
FIGURE 3: Chronological life span (CLS) is modified according to the phosphorylation state of Coq7. SDc-ura liquid cultures with 2% glucose as carbon source were inoculated at 0.1 OD_660nm_/ml with the indicated strains. After 5 days of growth, samples of each culture were harvested to measure the cell viability in YPD plates. The third day was used as control (100%). Plot shows a representative experiment repeated three times with similar results. See full dataset in Supplementary Material. **(A)** CLS in *COQ7* strains. Average life was coq7∆/pNMQ7 (12.1 ± 0.8), *coq7*∆/pRS316 (2.8 ± 0.2), *coq7*∆/pAAA (9.1 ± 0.7), *coq7*∆/pDED (11.4 ± 0.8) and *coq7*∆/pmQ7 (14 ± 0.8). **(B)** CLS in *PTC7* strains. Average life was BY4741 (12.7 ± 0.7), *ptc7*∆ (6.8 ± 0.4), *ptc7*∆ + 10 µM CoQ_6_ (6.9 ± 0.8) and *ptc7*∆*/PTC7* (12.7 ± 0.5). **(C)**CoQ_6_ of yeast strains analyzed in CLS experiment of *PTC7* gene strains. Similar data for *COQ7* gene CLS experiment was showed in Figure 1A. Cells from the indicated strains were subjected to mitochondrial purification, lipid extraction and quinone quantification. Samples were injected three times and the experiment was repeated at least three times.

Chronological lifespan (CLS) measurement refers to yeast cells longevity in an exhausted culture media after the onset of stationary phase 37. The extension of CLS depends on the existence of an intact respiratory metabolism 38,39,40,41 and also by the expression of intact antioxidant defenses 42,43,44. Given that these requirements are affected in cells expressing both Coq7-pAAA and Coq7-pDED, CLS have been measured in these cells (Figure 3A and Table S1). The *coq7*∆/pRS316 strain showed shorter mean CLS (2.8 ± 0.2 days) compared to both *coq7*∆/pNMQ7 (12.2 ± 0.7 days) and *coq7*∆/pmQ7 strains (14 ± 0.8 days). The *coq7*∆/pDED strain showed a slightly shorter mean CLS (11.4 ± 0.8 days) while the *coq7*∆*/pAAA* strain had a clearly shorter mean CLS (9.1 ± 0.7 days). These results compared to CoQ_6_ content (Figure 1A) support a model where the phosphorylation state of Coq7 affects CLS in yeast. Comparing the levels of CoQ_6_ and CLS in the Coq7 versions we found an assortment of results. Thus, CoQ_6_ level alone is not a factor that can explain the changes observed in CLS.

Ptc7 regulates CoQ_6_ biosynthesis by dephosphorylation of Coq7 28. We studied whether the lack of Ptc7 would result in a compromised CLS. The *ptc7*∆ strain displayed a shortened mean CLS compared to wild type (6.8 ± 0.4 days versus 12.7 ± 0.7 days) (Figure 3B and Table S2). These results indicated that this phosphatase might regulate the normal function of longevity-associated pathways in yeast. CoQ_6_ content of the *ptc7*∆ strain was significantly lower than both wild type and *ptc7*∆ strains rescued with the wild type allele (Figure 3C). As a result, CoQ_6_ supplementation in CoQ_6_ deficient *coq7*∆ yeast, rescues respiratory growth and oxidative stress resistance 45. This was demonstrated by measuring the CLS of our strains in CoQ_6_ supplemented media (Figure 3B). Interestingly, the addition of exogenous CoQ_6_ to the *ptc7*∆ strain increased mitochondrial CoQ_6_ to wild type levels (Figure 3C) but did not rescue CLS of the strain (7 ± 0.8 days), suggesting that the decreased CoQ_6_ content is not responsible for the shortened CLS of the *ptc7*∆ strain. These results indicate that CoQ_6_ levels cannot explain alterations of CLS in both *coq7*∆ and *ptc7*∆ mutants. Moreover, the reduction in CLS in the *ptc7*∆strain indicates that there might be additional functions of Ptc7 besides Coq7 activation.

### Respiratory supercomplexes are altered in Coq7 phosphomutants

**Figure 4:**
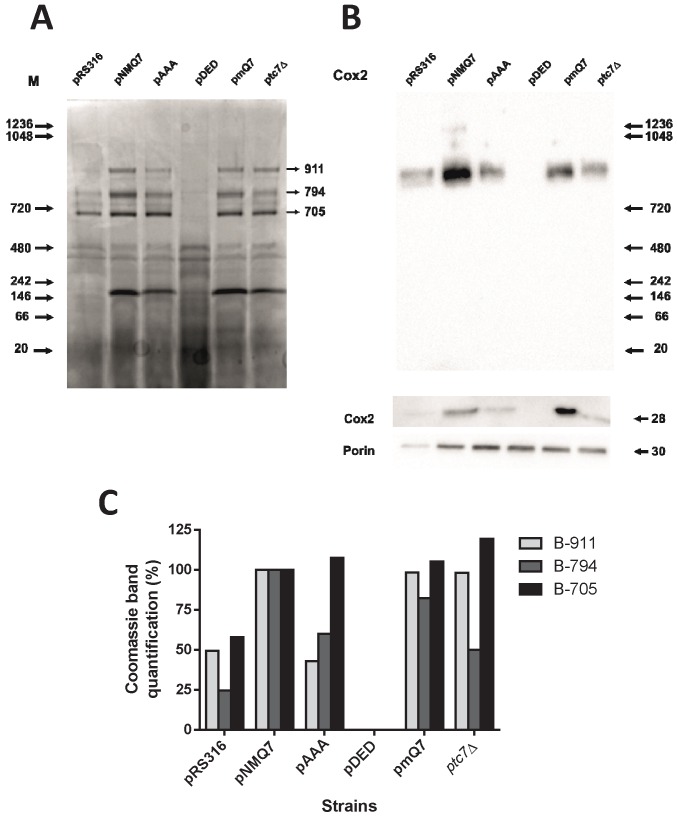
FIGURE 4: Mitochondrial supercomplexes stability is affected in yeast expressing Coq7 phosphomutant versions. Pure mitochondrial samples obtained from the indicated strains were subjected to digitonin solubilization and 200 µg of each sample were analyzed by 1D BN-PAGE. **(A)** Coomassie staining of 1D BN-PAGE gels. Molecular weight markers are indicated on the left. On the right side, the three arrows indicate the calculated molecular weight of bands analyzed by densitometry in Figure 4C. **(B)**Upper panel, Western blot analysis blotted with antibody against the subunit Cox2 of Complex IV of a 1D BN-PAGE gel obtained in parallel to the Coomassie stained gel shown in Figure 4A using the same original mitochondrial digitonin solubilized samples. Lower panel, analysis by SDS-PAGE and Western blot of mitochondrial samples blotted with antibodies against the subunit Cox2 of Complex IV and porin. **(C)** Densitometry analysis of bands indicated with arrows in 4A. The analysis was performed with the software Image Lab 4.0 of Biorad. Band optical density was normalized with the full lane density and compared with the optical density of bands corresponding to the positive control, *coq7*∆/pNMQ7. Results are representative of a set of two.

Mutant versions of Coq7 showed a clear defect on MRC activities focused in coupled reactions (NADH-cytochrome *c* reductase and succinate-cytochrome *c* reductase) that cannot be explained by the levels of CoQ_6_. In the *coq7*∆/pAAA strain the NADH-cytochrome *c* reductase activity is 40% less than wild type while the simple activity of both separated complexes is even higher than wild type. However, *coq7*∆/pAAA mitochondria contain 250% of CoQ_6_ compared to wild type (Figure 1). One possibility is that the expression of Coq7 versions can modify the stability or assembly of respiratory complexes. To this end, we analyzed the assembly of respiratory complexes by BN-PAGE in permeabilized mitochondria isolated from c*oq7*∆ yeast expressing several versions of Coq7 (Figure 4). Cells were cultured first in YPD to increase cell mass and then the culture was transferred to YPG to activate mitochondrial metabolism. The *coq7*∆/pNMQ7 strain showed a typical profile of mitochondrial supercomplexes that mostly agrees with previously published data in wild type yeast 46, with three prominent bands at 911, 794 and 705 kDa, a double band around 480 kDa and a band located at 242 kDa (Figure 4A). The mass-spectrometry analysis of the three larger bands of *coq7*∆/pNMQ7 yeast indicated that the 911 kDa band corresponds mainly to complex V, the band of 794 kDa to complex III + complex IV and 705 KDa correspond to complex V. The detailed list of detected and identified proteins can be consulted at the Table S3 of Supplementary Material. Full MASCOT analysis is included in Supplementary Material. Immunoblot of this BN-PAGE gel with anti-Cox2 antibody (Figure 4B) showed differential intensities of supercomplex at 794 kDa but not in other bands. No reaction was observed in mitochondria from *coq7*∆/pDED yeast, and very low in mitochondria from *coq7*∆/pRS316 yeast. Lower intensities were also found in both *coq7*∆/pAAA and *ptc7*∆ strains. Higher intensity was observed in control *coq7*∆/pNMQ7 and *coq7*∆/pmQ7 strains. The quantitative analysis of Coomassie staining showed that the 911 kDa and 794 kDa bands were significantly affected in both Coq7 mutants (Figure 4C). The expression of modified versions of Coq7 induced alterations in the assembly profile of respiratory complexes, being more dramatic in the *coq7*∆/pDED strain.

### Mitophagy but not autophagy is affected in *ptc7*∆ mutant strains

**Figure 5:**
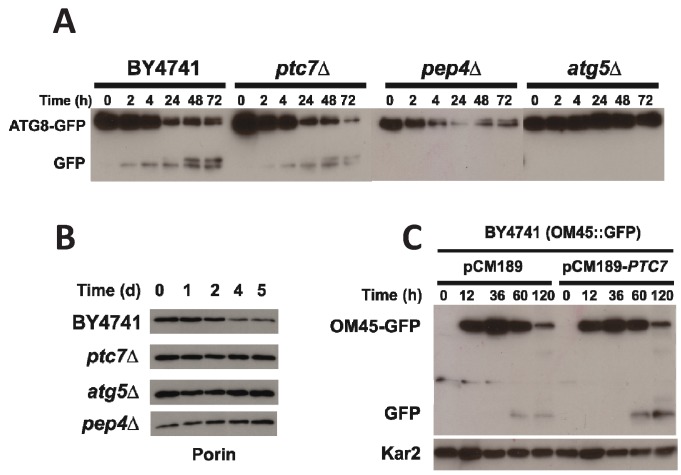
FIGURE 5: Mitophagy induction but no autophagy is compromised in Ptc7 yeast. **(A) **Yeast strains harboring an expression plasmid expressing the ATG8-GFP gene were grown in YPD 16 hours. Then, yeasts were washed and media was replaced by SDc-N with 2% glucose to activate autophagy. 0.5 OD_660 nm _of Yeast cells were harvested at indicated time points. Protein extracts were analyzed by SDS-PAGE and Western blot using anti-GFP antibody. **(B)** Mitophagy analysis by porin degradation. Yeast strains were grown in YPD for 16 hours. Then, yeasts were washed and media was replaced by SDc-N with 2% glucose to activate mitophagy. Protein extracts were analyzed using anti-porin antibody. **(C) **Ptc7 overexpression increases the mitophagy induction. Yeast strains expressing the OM45-GFP gene were grown in YPD for 16 hours. Then, yeasts were washed and media was replaced by YP Lactate. Protein extracts were analyzed using anti-GFP antibody for GFP-Om45, an outer membrane mitochondrial protein, and anti-Kar2 antibody for Kar2, an endoplasmic reticulum marker used as control for non-mitochondrial protein degradation. Original film plates used to prepare the panels are shown in Figure S1. Analysis of densitometry of panels B and C are shown in the Figure S2, S3 and S4. Data are mean ± SD, N ≥ 3 independent assays in all the experiments.

Mitophagy is a general process that degrades damaged or non-useful mitochondria, which is required to maintain CLS in eukaryotic cells 31,47,48. The *ptc7*∆ strain combines a low amount of CoQ_6_ with a low CLS while the *coq7*∆/pDED strain shows a low amount of CoQ_6_ but a CLS comparable to wild type (Figures 1A and 3A). Both strains are equivalent in terms of Coq7 phosphorylation state and therefore Ptc7 must have another function independent of CoQ_6_ biosynthesis that is responsible for the low CLS measured. We have previously demonstrated that the survival of human fibroblasts deficient in CoQ_10_ production depends on a proper recycling of dysfunctional mitochondria by mitophagy 49. We speculated that Ptc7 might regulate this process. Therefore, we analyzed whether the general process of autophagy and/or mitophagy could be compromised in *ptc7*∆. Macroautophagy was analyzed by monitoring the Atg8p proteolysis using a plasmid expressing Atg8-GFP tag at the C-terminal under endogenous promoter (ATG8-GFP) (Figure 5A) 50. Yeast cells were grown in YPD for 16 hours and subsequently were resuspended in nitrogen deprived medium (SDc-N) to induce macroautophagy. The positive control strain (BY4741; wild type) resulted in a marked increase of Atg8-GFP degradation, visible as free-GFP starting at 2 hours, which was maintained until 72 hours. According to previous reports, *pep4*∆ and *atg5*∆ strains, which are deficient in protein degradation in the vacuole or autophagy respectively, showed impaired autophagy induction 51 (Figure 5A). In accordance with induced autophagy, a decrease of intact Atg8-GFP and a subsequent increase on free GFP was also observed in *ptc7*∆ strain, indicating that macroautophagy induction is not compromised in this strain.

To further study the potential role of Ptc7 in mitochondrial recycling, mitophagy was analyzed by monitoring the degradation of porin. Yeasts were grown in YPL for 16 hours and media was replaced with a nitrogen deprived media (SDc-N) to induce mitophagy (Figure 5B). The wild type strain showed a decreased amount of porin starting at day 2 and was gradually reduced until day 5. Porin degradation was not observed in the *ptc7*∆ strain under similar conditions, suggesting that Ptc7 is involved in mitochondrial recycling. Porin degradation was not observed in the autophagy-deficient yeast strains *atg5*∆ and *pep4*∆*.* A protein expression analysis normalized with total protein loaded corroborated previous data (Figure 2S). To further determine the involvement of Ptc7 in mitophagy induction we determined the effect of Ptc7 overexpression on mitophagy (Figure 5C)*. *Wild type yeast harboring the GFP protein fused in frame with the mitochondrial outer membrane protein Om45 were transformed with the empty yeast expression plasmid (pCM189) or containing the yeast *PTC7* coding sequence (pCM189-*PTC7*). Yeast cells were cultured in YPD for 16 hours and media was replaced with YPL, a non-fermentable carbon source, to induce mitochondrial biosynthesis. Yeast cells were grown through prolonged stationary phase to induce the selective recycling of mitochondria by mitophagy 31. *PTC7* over-expression produced increased GFP free levels starting at 60 hours (270%) and at 120 hours (470%) of growth, indicating that the over-expression of *PTC7* enhances mitophagy induction (Figure S3). Remarkably, Kar2, a marker of endoplasmic reticulum, was not affected (Figure S4), indicating that macroautophagy was not activated by *PTC7* overexpression. Taken together, these data indicate that Ptc7 regulates or participates specifically in mitophagy but not in general macroautophagy, suggesting that this specific process may compromise CLS in *ptc7*∆ yeast.

## DISCUSSION

Our understanding of the role of mitochondria on cell physiology and metabolism has evolved in the past decades from only a bioenergetics role to an interconnected organelle whose functions exceed energy supply. A key factor for part of these mitochondrial functions is CoQ, as proper regulation of its biosynthesis pathway exceeds cell bioenergetics and is tightly linked to cellular homeostasis. This idea supports the pleiotropic effect observed in patients with primary CoQ deficiency 7,52. Although most Coq proteins are involved in enzymatic steps required for CoQ_6_ biosynthesis 8, some Coq proteins play a structural function and are required to stabilize the biosynthetic complex 8,53. In yeast, the shift from fermentation to respiration activates the expression of *COQ* genes to accommodate CoQ_6_ biosynthesis to respiratory metabolism 19,54, which is associated to regulatory mechanisms such as biosynthesis complex assembly and post-translational modifications 9,22,25,55.

The protein encoded by the *COQ7* gene has its catalytic activity controlled by complex regulatory mechanisms 16,22,25,28,56,57. Coq7 is a di-iron carboxylate protein with hydroxylase activity 58 that converts DMQ_6_ in demethyl ubiquinone. The lack of Coq7 in yeast fully abolishes CoQ_6_ biosynthesis and other intermediates although the expression of some point mutants such as *e2519* (E223K) accumulates DMQ_6_ 34, which would indicate that this step is a regulatory step in this pathway. DMQ_6_ is also accumulated in wild type cells after the post-diauxic shift supporting this hypothesis 19,34. A model of CoQ_6_ biosynthesis complex assembly, based on BN-PAGE, size exclusion chromatography and immunoprecipitation data, show that a precomplex of about 700 kDa is formed, which accumulates DMQ_6_ 10 that, ultimately, will be converted into CoQ_6_ after the recruitment of Coq7 to the complex 9,55. Coq7 is a phosphoprotein that, in the dephosphorylated state, activates CoQ_6_ biosynthesis probably by interacting with the 700 kDa precomplex 22. Here we have shown that the phosphomimetic Coq7 (Coq7-DED), which mimics Coq7 phosphorylation status in *ptc7*∆ strain 28, and the fully dephosphorylated Coq7 (Coq7-AAA) induced a decreased and an increased levels of CoQ_6 _respectively, indicating that the regulatory step on CoQ_6_ biosynthesis is at least partially controlled by Coq7 phosphorylation.

The physiological analysis of these strains confirms the catalytic function of Coq7 in CoQ_6_ biosynthesis and its regulatory function in mitochondrial metabolism. Expression of both Coq7-AAA and Coq7-DED decreases antioxidant protection and increases the production of endogenous oxidative stress. A similar result was reported for the *ptc7*∆ strain 28. These negative effects cannot be explained by the alteration of CoQ_6_ levels, as the *coq7*∆/pmQ7 strain, which also shows high levels of CoQ_6_, had an endogenous oxidative stress and sensitivity similar to wild type. CoQ_6_ has been reported as an antioxidant molecule mainly to protect cell membranes 35,36,59 but also as a pro-oxidant agent under physio-pathological conditions 60. CoQ is involved in ROS production in the MRC mainly because of the transfer of electrons to complex III 61,62. It has been recently demonstrated in *Drosophila* that the reduced stage of CoQ (CoQH_2_) causes CoQH_2_-mediated ROS in complex I by retrograde electron transport and contributes to extend lifespan 63. Similar conditions of higher CoQH_2 _in mammal-cultured cells contribute to the partial degradation of complex I by the same mechanism 64. Thus, we propose that the production of CoQ_6_ in strains with low Coq7 phosphorylation are generating unbalanced CoQ_6_ levels and redox stages out of the MRC complexes, which does not occur in the *coq7*Δ/pmQ7 strain. The unbalanced levels of CoQ_6_ in these strains increase their sensitivity to external oxidative stress, decreasing their longevity. It has been reported previously that linolenic acid can induce mitochondrial oxidative stress 65. The effect of linolenic acid may be enhanced by the mitochondrial dysfunction generated by the expression of pAAA and pDED versions. In fact, the expression of pAAA or pDED induces a higher level of endogenous oxidative stress and also affects the stability of respiratory complexes, which possibly make those strains more susceptible to linolenic acid-induced oxidative stress.

CoQ is a component of the respirasome and it is proposed that pools of CoQ are bound to specific super assembly stages of respiratory complexes 66, which depends on the carbon source 64. Here our data show that super-complexes are dissociated in yeast strains with unbalanced CoQ_6_ concentrations, which also showed mitochondrial dysfunction, but not in the *COQ7* multicopy transformed yeast strain (*coq7*∆/pmQ7) that exhibit a phenotype similar to wild type in both respiratory activities and assembly profile. Previous analyses of CoQ_6_ biosynthesis complex showed that Coq7 is partially located in a large size complex but it can also be detected in smaller ones even as a monomer 10. Coq7 interacts with Coq9, which shows lipid-binding activity and Coq7 interacting domains 16. Several steps are required to integrate the CoQ_6_ biosynthesis complex in yeast. First, there is an initial nucleation of Coq proteins around Coq4 10,67 to build up the Q-synthome, which requires stabilization by Coq8 68, an unusual protein kinase that makes the pre-complex formation 20 and demethoxy-Q_6_ accumulation possible 19. At this step Coq7 is mostly phosphorylated and it is not a component of the pre-complex 10; Coq7 must then be dephosphorylated by Ptc7 to get activated and to bind to the fully active Q-synthome 9,28,55. We speculate that CoQ_6_ biosynthesis must be balanced with the components of the respiratory complexes. In light of our data, we hypothesize that the interaction of Coq7 with other proteins of the biosynthesis complex is a requirement for its integration in the MRC, and that extreme phosphorylation stages might prevent this possibility. This is supported by the data obtained from the *ptc7*∆ strain that mimics Coq7-DED expressing cells without eliminating all regulation of Coq7.

In fact, yeast contains two other mitochondrial phosphatases that belong to the same family of Ptc7, Ptc5 that participates in pyruvate dehydrogenase complex (PDH) regulation 30,69 and Ptc6/Aup1 that participates in PDH regulation and is required for mitophagy activation 31,70. It is possible that these mitochondrial phosphatases may dephosphorylate Coq7 in the absence of Ptc7, which would explain the less severe effect on mitochondrial functions. Interestingly, the *ptc7*∆ strain shows shorten CLS compared to the *coq7*∆/pDED strain and it is not rescued by CoQ_6_ supplementation, although this strain shows a significant decrease of CoQ_6_ content. CLS is only rescued when it is transformed with the homologous gene, which is also able to rescue the CoQ_6_ content; overall these data indicate that Ptc7 has at least a dual function in yeast. As indicated above, Ptc6 is required for mitophagy activation 31,70 and we have shown here that Ptc7 induces mitophagy as a mechanism to recycle defective mitochondria caused by CoQ_6_ deficient MRC.

**Figure 6:**
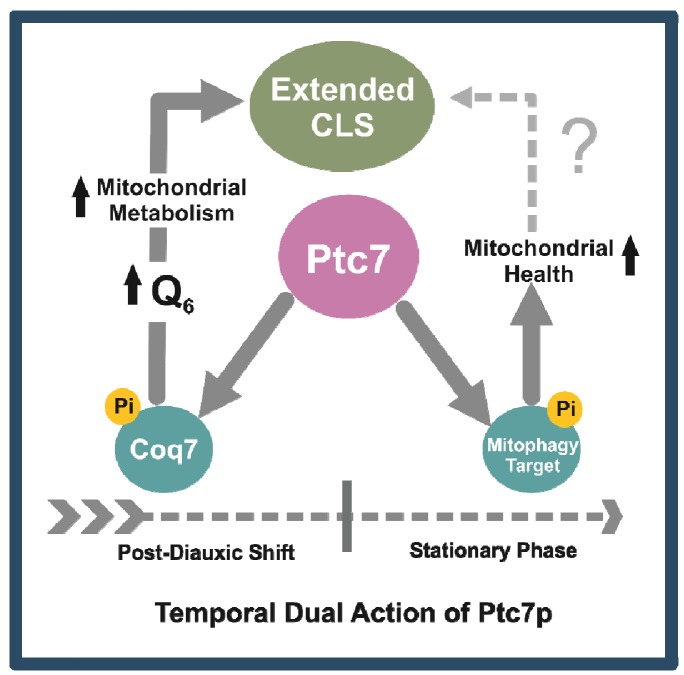
FIGURE 6: Model of Ptc7/Coq7 action to promote CLS extension. Ptc7 can regulate mitochondrial metabolisms in two different ways that are not directly related. After post diauxic shift, Coq7 expression and its activation by Ptc7 dephosphorylation leads to high levels of CoQ_6_ concomitant with an increase of mitochondrial biogenesis. CLS extension requires a respiratory growth phase previous to the entrance in the stationary phase. The excess of mitochondria after the start of the stationary phase can be recycled by mitophagy to maintain an appropriated mitochondrial homeostasis. Ptc7 participates on mitophagy most likely by dephosphorylating of unknown target(s). Both functions, at different time frames, can help to promote a CLS extension in yeast.

The lack of mitophagy in *ptc7*∆ yeast, which is a requisite to extend CLS 71,72, can explain its shorten CLS and the negative recovery after CoQ_6_ supplementation. We propose a dual and temporary differential function of Ptc7 on mitochondrial physiology and homeostasis (Figure 6). The entry of yeast on the post-diauxic shift (PDS) increases CoQ_6_ biosynthesis and therefore respiratory metabolism by Coq7 activation. However, cell homeostasis during PDS requires the recycling of the excess and defective mitochondria. Ptc7, which would trigger mitophagy by dephosphorylating an unknown target involved in this process, would be a key regulator of mitochondria homeostasis in yeast by coordinating mitochondria recycling with CoQ_6_ biosynthesis.

## MATERIAL AND METHODS

### Yeast strains and growth media

Yeast strains used in this study are listed in Table 1. Growth media for yeast and bacteria were prepared as described previously 22. Yeast cells were grown at 30°C with shaking (200 rpm).

**Table 1:** Strains used in this study.

**Strains**	**Genotype**	**Source**
***coq7Δ***	BY4741; MATa; his3∆1; leu2∆0; met15∆0; ura3∆0; YOR125c::KanMX4	Euroscarf
**JM8**	MAT α; ∆ade1;ρ^0^	74
**JM6**	MAT a; ∆his4; ρ^0^	74
***ptc7∆***	BY4742; MATα; his3∆1; leu2∆0; lys2∆0; ura3∆0; YHR076w::KanMX4	Euroscarf
**BY4742**	MATα; his3∆1; leu2∆0; lys2∆0; ura3∆0.	Euroscarf
***atg5∆***	BY4742, MATα; his3∆1; leu2∆0; lys2∆0; ura3∆0; YPL149w::kanMX4	Euroscarf
***pep4∆***	BY4742; MATα; his3∆1; leu2∆0; lys2∆0; ura3∆0, YPL154c::kanMX4	Euroscarf
**OM45::GFP**	BY4741; MATα; his3∆1; leu2∆0; met15∆0; ura3∆0; YIL136W::GFP(S65T)-HIS3MX6	ATCC

### Mitochondrial purification and BN-PAGE

Yeast cultures were grown in the appropriate culture media and mitochondria were purified according to the described method 73. To solubilize mitochondria, 2 mg of pure mitochondria was incubated in 240 μl of solubilization buffer containing digitonin in a ratio 4:1 with protein, 1 mM PMSF, 10% glycerol, 150 mM potassium acetate and 30 mM HEPES, pH 7.4 for 30 min at 4°C. Solubilized samples were subjected to two rounds of centrifugation in a Beckman Coulter Microfuge 22R (15,000 × g, 15 min, at 4°C) and the supernatant was collected to BN-PAGE. Proteins quantification was performed by Bradford method (Biorad). BN-PAGE was performed with precast 3-12% gradient gels (NativePAGE^TM^ Novex® Bis-Tris Gels) using the Xcell Sure Lock^TM^ Mini-Cell electrophoresis system, including NativePAGE^TM^ anode and cathode buffers according to the company instructions at 4°C. Lanes were loaded with 200 µg of mitochondrial solubilized supernatant and as MW marker NativeMark^TM^ Unstained Protein Ladder was used. Gels were stained with Coomassie solution or were blotted onto PVDF, blocked in 5% Blocking Reagent (Biorad) and phosphate-buffered saline. Proteins were detected by ECL using Luminata Crescendo (Millipore) and luminescence detected by Gel Doc XR+ image processing software (Bio-Rad). Cox2p (Novex Life Technologies), porin (Invitrogen) and Coq7 (Gift of Dr. C. F. Clarke, UCLA, USA) primary antibodies were used at 1: 2,000, 1: 1,000 and 1: 2,000 respectively. Goat anti-mouse and anti-rabbit secondary antibodies conjugated to horseradish peroxidase (Calbiochem) were used at a 1:5,000 dilutions.

### Mitochondrial respiratory chain (MRC) activities

Fresh mitochondria were used to measure NADH-cytochrome *c* reductase, succinate-cytochrome *c* reductase activities and superoxide generation. Other MRC activities (NADH-DCIP reductase, succinate-DCIP reductase and decylubiquinol-cytochrome *c* reductase), were performed with fresh samples subjected to one freeze-thaw cycle. All MRC activities were determined according to previously published methods 34. Superoxide generation was measured using the same method than for measuring complex III activity (decylubiquinol-cytochrome *c* reductase) but using acetylated cytochrome *c* instead cytochrome *c* 33. H_2_O_2_ generation was performed using Amplex Red kit (Invitrogen) according to the manufacturer instructions.

**Table 2:** Vectors used in this study.

**Name**	**Description**	**Specification**	**Source**
**pRS316**	Yeast expression centromeric vector with *URA3* auxotrophy	Empty control	34
**pRS426**	Yeast expression episomic vector with *URA3* auxotrophy	Empty control	34
**pNMQ7**	pRS316 harboring the full yeast *COQ7* with promotor and terminator sequences	Positive control	34
**pAAA**	pRS316 with loss of function mutations in *COQ7* (S20A, S28A and S32A)	Non-phosphorylatable *COQ7* version	22
**pDED**	pRS316 with gain of function mutations in *COQ7* (S20D, S28E and S32D)	Mimicking a permanent phosphorylated *COQ7* version	22
**pmQ7**	pRS426 harboring yeast *COQ7*	*COQ7* multicopy complementation	34
**ATG8-GFP**	pRS316 harboring *ATG8*-GFP tag	*ATG8* tagged with GFP	75
**pCM189**	Yeast centromeric expression vector with *URA3* auxotrophy	Empty control	76
**pCM189-PTC7**	pCM189 harboring yeast *PTC7*	*PTC7* null mutant complementation	28

### Chronological Life Span (CLS)

Analysis was performed as previously described 37. Briefly, cells were incubated in YPD and CLS was monitored starting at day 3 by quantification of colony forming units (CFUs) every 48 hours using the software OpenCFU 3.9 beta. The number of CFUs at day three was considered as 100% survival. Survival log-rank analyses Sigmastat 3.0 (SPSS) were calculated for each pair of lifespan analyses and average lifespan were shown in the corresponding dataset in Supplementary Material.

### Protein mass spectrometry identification

Acrylamide gel bands were distained in NH_4_HCO_3_ 25 mM water/ACN 50:50. For reduction of cysteines, samples were incubated at 56°C for 60 min in 10 mM DTT (NH_4_HCO_3_ 25 mM). Cysteine carbamidomethylation was performed embedding bands in 55 mM IAA solution and incubating at room temperature for 30 min. Samples were in gel digested by trypsin (0.2 µg/µl in 1 mM HCl) diluted in NH_4_HCO_3_. Gel bands were covered with enzyme solution and incubated at 30°C overnight. Reaction was stopped with acetonitrile, and peptide were extracted adding 0.2% TFA. Prior to protein identification by MALDI-MS, we used nano-liquid chromatography for reversed phase peptide separation. Peptide fractioning was performed in a Proxeon EASY-nLC II apparatus with a C18 column (EASY-column, 75 µm x 100 mm) and mobile phases: Buffer A: 0.1% TFA (H_2_O) and Buffer B: 0.1% TFA (ACN). Elution process was divided in the following flow steps: 0-48 min gradient 2%-45% B; 48-50 min gradient 45-100% B; 50-60 min isocratic 100% B. Fractions were collected every 15” using a Bruker Proteineer fc fraction collector and spotted onto a MALDI target plate. 192 samples were spotted and overlaid with 0.5 µl drops of HCCA matrix solution and left air dry. MALDI measurements were performed in a Bruker Ultraflextreme MALDI-TOF/TOF system, using Bruker Peptide Calibration standards as mass standards. A MALDI fingerprint spectrum was obtained for each fraction and peaks with higher intensity in each spot were selected as mass precursors for MS/MS peptide fragmentation experiments. Bruker WARP-LC software was used to process spectra and to integrate chromatography fractions data. Protein identification was carried out using MASCOT server. Ammonium bicarbonate, DL-dithiothreitol (DTT), Iodoacetamide (IAA), trypsin from porcine pancreas, trifluoroacetic acid (TFA) and α-cyano-hydroxycinnamc acid (HCCA) were purchased from Sigma-Aldrich. Water (HPLC grade) and acetonitrile (HPLC grade) were purchased from Fluka. Peptide calibration standards were purchased from Bruker.

### Total yeast protein extraction

Cells (10.10^6^ in 500 µl of water) were disrupted with 100 µl 2 M NaOH and 35% β-mercaptoethanol for 15 min on ice. Proteins were precipitated after the addition of 100 µl 3 M TCA for 15 min on ice. The pellet obtained after 15 min centrifugation on a microcentrifuge at full speed was washed with acetone, dried and resuspended in 30 µl of SDS-PAGE 1 x LB.

### Other methods

CoQ_6_ quantification was performed using mitochondrial samples according to previously published methods 28. Densitometry analysis was carried out with a Gel Doc XR+ (Bio-Rad) with Image Lab 4.0 as software analysis. Statistical (t-Student) analyses were carried out using the Sigmastat 3.0 (SPSS) statistical package. Mitochondrial DNA integrity was checked in all strains using two ρ^0^ strains, JM6 and JM8 strains. All results are expressed as the average ± SD. Statistical analyses were carried out using the Sigmastat 3.0 (SPSS) statistical package.

## SUPPLEMENTAL MATERIAL

Click here for supplemental data file.

All supplemental data for this article are also available online at http://microbialcell.com/researcharticles/balanced-coq6-biosynthesis-is-required-for-lifespan-and-mitophagy-in-yeast/.
