# Supplementary material for: Balanced CoQ6 biosynthesis is required for lifespan and mitophagy in yeast
Source: Microb Cell. 2017 Feb 3;4(2):38–51. doi: 10.15698/mic2017.02.556 (PMC5349121; doi:10.15698/mic2017.02.556)
Supplement: Supplementary file 1 [file mic-04-038-s01.pdf]

Table S1: CLS Dataset (COQ7)

	Average Life	pNMQ7	pRS316	pAAA	pDED	pmQ7
pNMQ7	12.1 ± 0.8		*p*<0.001	*p*<0.001	*p*=0.669	*p*=0.124
pRS316	2.8 ± 0.2			*p*<0.001	*p*<0.001	*p*<0.001
pAAA	9.1 ± 0.7				*p*=0.0234	*p*<0.001
pDED	11.4 ± 0.8					*p*=0.0554
pmQ7	14 ± 0.8					

Table S2: CLS Dataset (PTC7)

	Average Life	Wt	ptc7Δ	ptc7Δ + 10μM Q_6	ptc7Δ*:PTC7*
Wt	12.74 ± 0.71		*p*<0.001	*p*<0.001	p= 0.365
ptc7Δ	6.84 ± 0.41			p= 0.390	*p*<0.001
ptc7Δ + 10μM Q_6	6.98 ± 0.85				*p*<0.001
ptc7Δ*:PTC7*	12.68 ± 0.52				

Table S3 Protein detected in MS/MS analysis

Band	Protein	Score
911	ATP synthase subunit alpha, mitochondrial	519
	ATP synthase subunit beta, mitochondrial	488
	ATP synthase subunit gamma, mitochondrial	114
	ATP synthase subunit d, mitochondrial	80
	Qcr1 Cytochrome b-c1 complex subunit 2, mitochondrial	23
794	Cor1 Chain A, Structure of The Yeast Cytochrome Bc1 Complex	317
	Qcr2 Chain B, Structure of The Yeast Cytochrome Bc1 Complex	287
	Cyt1 Chain D, Yeast Cytochrome Bc1 Complex	164
	Qcr7 Chain F, Structure of The Yeast Cytochrome Bc1 Complex	83
	Atp2 Chain D, Crystal Structure of Yeast Mitochondrial F1-Atpase	70
	Cox6	69
	Cox5a	64
	Rip1 Chain E, Structure of The Yeast Cytochrome Bc1 Complex	49
	Cox2	40
	Cox13	40
	Cox7	30
	Atp1	29
705	ATP synthase subunit beta, mitochondrial	475
	ATP synthase subunit alpha, mitochondrial	414
	ATP synthase subunit d, mitochondrial	129
	ATP synthase subunit gamma, mitochondrial	85
	Qcr1 Cytochrome b-c1 complex subunit 1, mitochondrial	35
	ATP synthase subunit 5, mitochondrial	28
	ATP synthase subunit epsilon, mitochondrial	27

Figure S1 Ponceau membranes of Figure 5

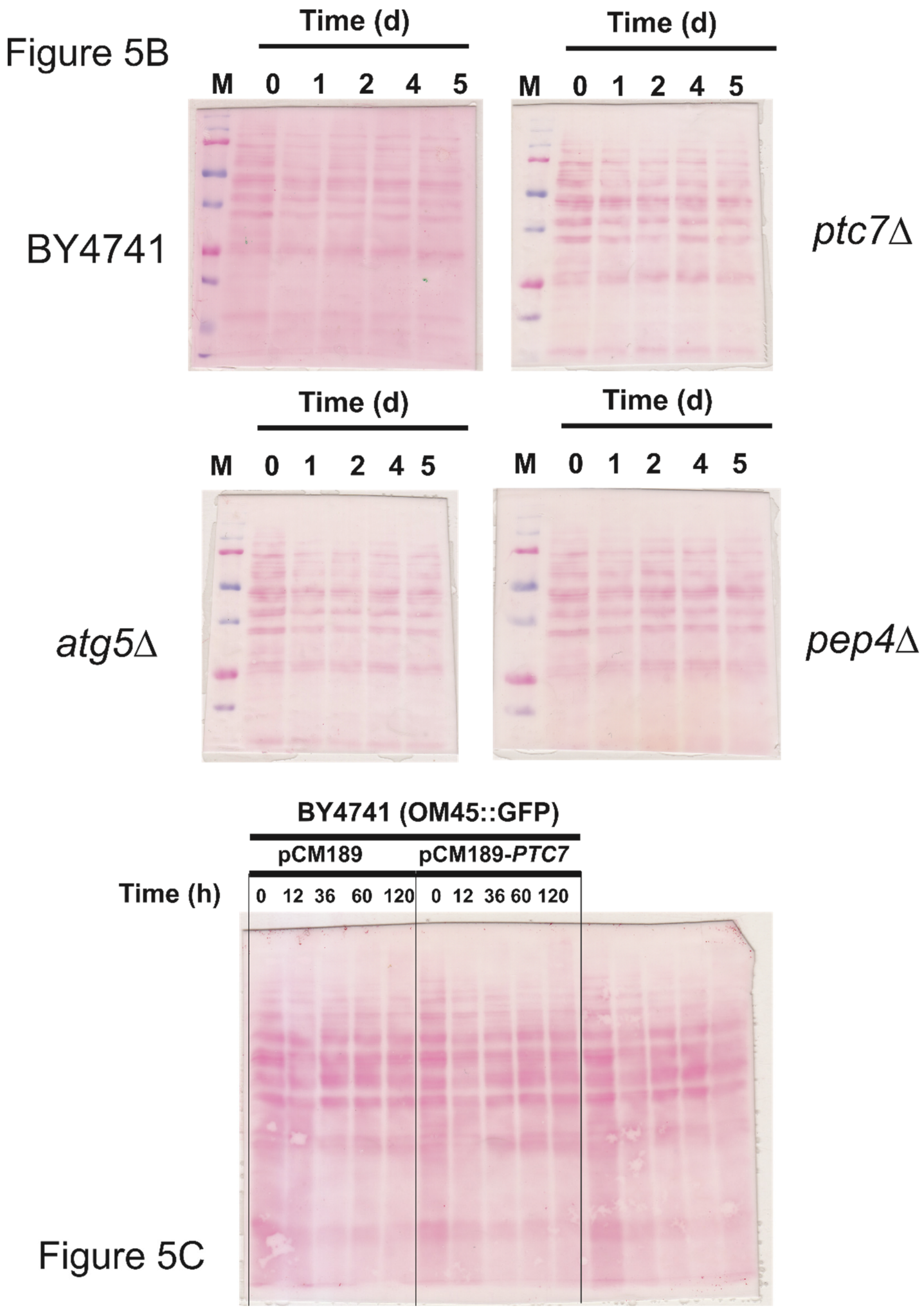

Figure S2 Quantitative analysis of Figure 5B

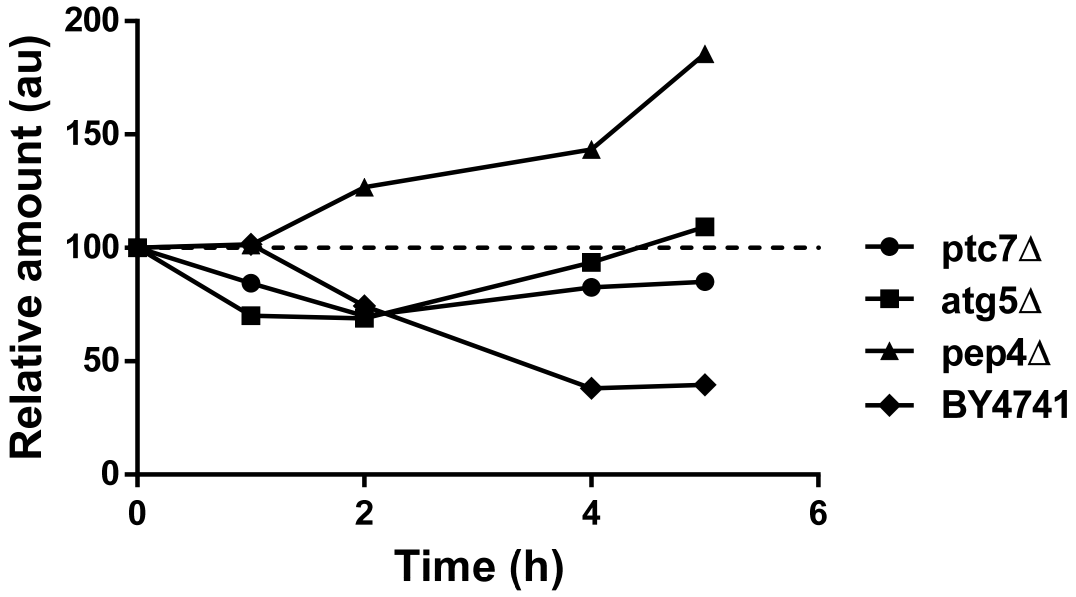

Figure S3 Quantitative analysis of Figure 5C

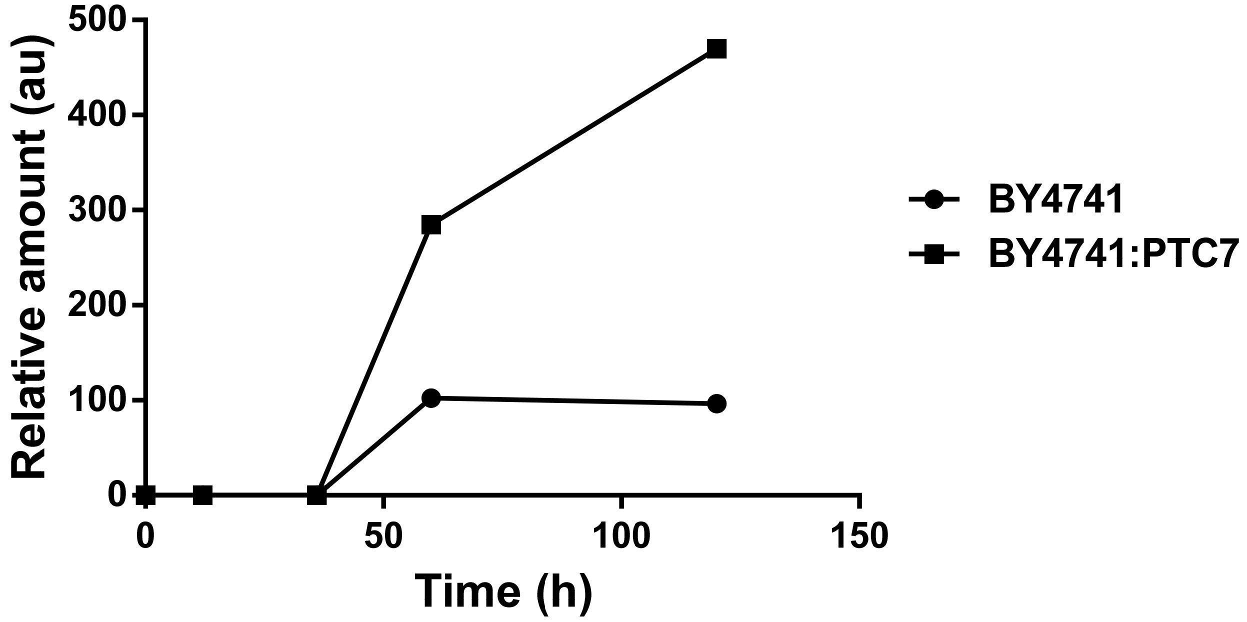

Figure S4 Quantitative analysis of Figure 5C

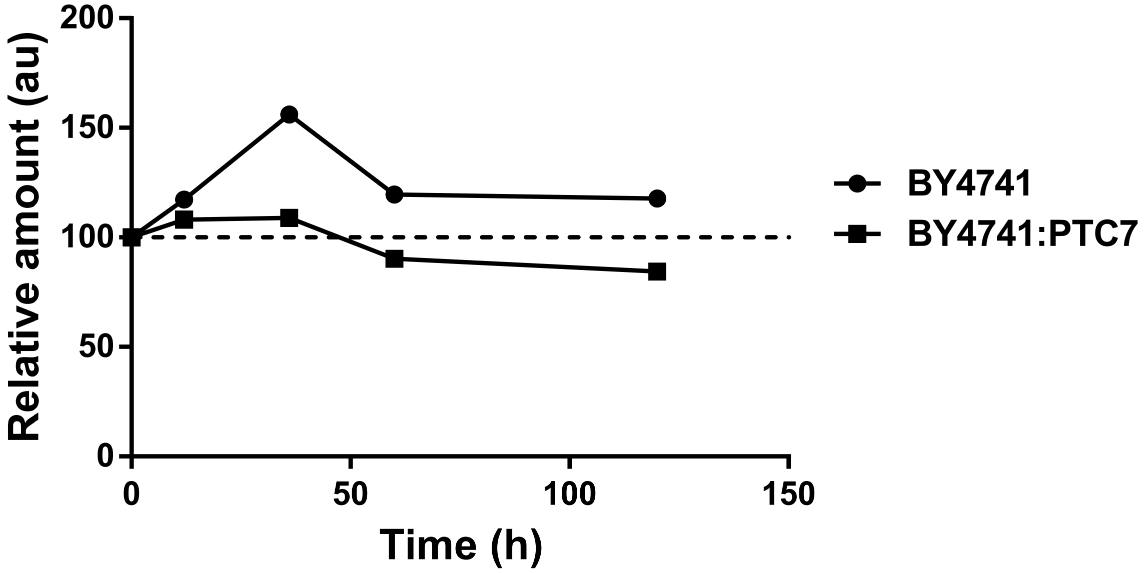

Figure Legends

Table S1: Dataset corresponding to CLS analysis of COQ7 strains (Figure 3A). Survival log-rank analyses were applied to each strain to calculate the average lifespan and for each pair of strains to obtain its statistical significance.

Table S2: Dataset corresponding to CLS analysis of PTC7 strains (Figure 3B). Survival log-rank analyses were applied to each strain to calculate the average lifespan and for each pair of strains to obtain its statistical significance.

Table S3: Summary of proteins detected in MASCOT analysis. Table containing all proteins detected in MASCOT analysis that show a positive score. They are fingerprint plus MS/MS spectra identifications.

Figure S1. Ponceau staining of membranes from Figure 5. Nitrocellulose membranes were stained with Ponceau solution before Western blot analysis to normalize the porin expression calculated in the Figure 5B (Figure S2) and also for the GFP expression calculated in the Figure 5C (Figure S3).

Figure S2. Densitometric analysis of proteins on western blots of Figure 5B. Porin bands for the indicated yeast strains (5B) were quantified with the software Image Lab 4.0 as software analysis and normalized using the Ponceau staining of the full lane (Figure S1). Data of time zero corresponds in the figure to the 100%. Other time points correspond to the ratio with 0 time.

Figure S3. Densitometric analysis of proteins on western blots of Figure 5C (GFP). GFP bands for the indicated experimental conditions (5C GFP) were quantified with the software Image Lab 4.0 as software analysis and normalized using the Ponceau staining of the full lane (Figure S1). At 0, 12 and 36 h the signal was zero and therefore the indicated data were not normalized at 0 time.

Figure S4. Densitometric analysis of proteins on western blots of Figure 5C (Kar2). Kar2 bands for the indicated yeast strains (5C Kar2) were quantified with the software Image Lab 4.0 as software analysis and normalized using the Ponceau staining of the full lane (Figure S1). Data of time zero corresponds in the figure to the 100%. Other time points correspond to the ratio with 0 time.

Figure S5: MASCOT analysis data summarized in the Table S3.

Mascot Search Results

```
User         :
Email        :
Search title :
MS data file : DATA.TXT
Database     : SwissProt 2013_10 (541561 sequences; 192480382 residues)
Taxonomy     : Saccharomyces Cerevisiae (baker's yeast) (7802 sequences)
Timestamp    : 23 Nov 2016 at 13:34:55 GMT
Protein hits : ATPB_YEAST  ATP synthase subunit beta, mitochondrial OS=Saccharomyces cerevisiae (strain ATCC 204508 / S288c) GN=ATP2 PE=1 S
               ATPA_YEAST  ATP synthase subunit alpha, mitochondrial OS=Saccharomyces cerevisiae (strain ATCC 204508 / S288c) GN=ATP1 PE=1
               ATP7_YEAST  ATP synthase subunit d, mitochondrial OS=Saccharomyces cerevisiae (strain ATCC 204508 / S288c) GN=ATP7 PE=1 SV=2
               ATPG_YEAST  ATP synthase subunit gamma, mitochondrial OS=Saccharomyces cerevisiae (strain ATCC 204508 / S288c) GN=ATP3 PE=1
               QCR1_YEAST  Cytochrome b-c1 complex subunit 1, mitochondrial OS=Saccharomyces cerevisiae (strain ATCC 204508 / S288c) GN=COR
               ATPO_YEAST  ATP synthase subunit 5, mitochondrial OS=Saccharomyces cerevisiae (strain ATCC 204508 / S288c) GN=ATP5 PE=1 SV=1
               ATP5E_YEAST ATP synthase subunit epsilon, mitochondrial OS=Saccharomyces cerevisiae (strain ATCC 204508 / S288c) GN=ATP15 PE
               YJ94_YEAST  Uncharacterized membrane protein YJR124C OS=Saccharomyces cerevisiae (strain ATCC 204508 / S288c) GN=YJR124C PE=
               VDAC1_YEAST Mitochondrial outer membrane protein porin 1 OS=Saccharomyces cerevisiae (strain ATCC 204508 / S288c) GN=POR1 PE
               SPT6_YEAST  Transcription elongation factor SPT6 OS=Saccharomyces cerevisiae (strain ATCC 204508 / S288c) GN=SPT6 PE=1 SV=1
               MGR3L_YEAST Protein MRG3-like OS=Saccharomyces cerevisiae (strain ATCC 204508 / S288c) GN=YKL133C PE=3 SV=2
               CCA1_YEAST  CCA tRNA nucleotidyltransferase, mitochondrial OS=Saccharomyces cerevisiae (strain ATCC 204508 / S288c) GN=CCA1
               HXKG_YEAST  Glucokinase-1 OS=Saccharomyces cerevisiae (strain ATCC 204508 / S288c) GN=GLK1 PE=1 SV=1
               RPB1_YEAST  DNA-directed RNA polymerase II subunit RPB1 OS=Saccharomyces cerevisiae (strain ATCC 204508 / S288c) GN=RPO21 PE
               SNF2_YEAST  Transcription regulatory protein SNF2 OS=Saccharomyces cerevisiae (strain ATCC 204508 / S288c) GN=SNF2 PE=1 SV=1
               GYP6_YEAST  GTPase-activating protein GYP6 OS=Saccharomyces cerevisiae (strain ATCC 204508 / S288c) GN=GYP6 PE=1 SV=2
               RS25A_YEAST 40S ribosomal protein S25-A OS=Saccharomyces cerevisiae (strain ATCC 204508 / S288c) GN=RPS25A PE=1 SV=1
               ELG1_YEAST  Telomere length regulation protein ELG1 OS=Saccharomyces cerevisiae (strain ATCC 204508 / S288c) GN=ELG1 PE=1 SV
               SPT2_YEAST  Protein SPT2 OS=Saccharomyces cerevisiae (strain ATCC 204508 / S288c) GN=SPT2 PE=1 SV=1
               MRT4_YEAST  mRNA turnover protein 4 OS=Saccharomyces cerevisiae (strain ATCC 204508 / S288c) GN=MRT4 PE=1 SV=1
```

Mascot Score Histogram

Ions score is -10*Log(P), where P is the probability that the observed match is a random event.
Individual ions scores > 22 indicate identity or extensive homology (p<0.05).
Protein scores are derived from ions scores as a non-probabilistic basis for ranking protein hits.

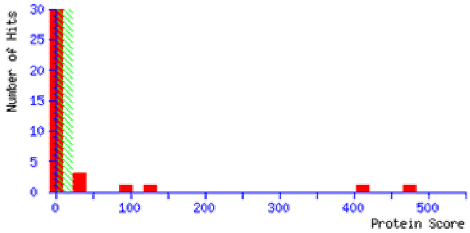

Peptide Summary Report

Format As | Peptide Summary | Help

Significance threshold p< 0.05 | Max. number of hits 20

Standard scoring ○ MudPIT scoring ● | Ions score or expect cut-off 0 | Show sub-sets 0

Show pop-ups ● Suppress pop-ups ○ | Sort unassigned Decreasing Score | Require bold red ☐

Overview Table

Click on column header to jump to entry in results list.
Move mouse over any indicator to highlight identical peptides.
Click on an indicator to see details of individual match.
Use check boxes to select sub-set of queries for new search.

Mouse over: -Query-
-Accession- -Sequence-

Hit:	1	2	3	4	5	6	7	8	9	10	11	12	13	14	15	16	17	18	19	20
☑ 1104.6168 (1+)								●		●	●	●			●	●		●		
☑ 1116.6917 (1+)	●																			●
☑ 1130.6974 (1+)																				
☑ 1138.6745 (1+)																				
☑ 1227.7187 (1+)																				
☑ 1246.7559 (1+)																				
☑ 1269.7374 (1+)																				
☑ 1297.6784 (1+)																				
☑ 1325.7435 (1+)		●																	●	
☑ 1346.7575 (1+)																				
☑ 1366.8267 (1+)	●																			
☑ 1367.8199 (1+)																	●			
☑ 1372.7843 (1+)																				
☑ 1383.8222 (1+)																				

☑ 1402.7971 (1+)																				
☑ 1410.9062 (1+)																				
☑ 1413.7663 (1+)																				
☑ 1420.7653 (1+)	●												●							
☑ 1435.8252 (1+)	●																			
☑ 1438.9222 (1+)																				
☑ 1442.8860 (1+)																				
☑ 1445.8207 (1+)																				
☑ 1453.8709 (1+)	●																			
☑ 1457.7951 (1+)																				
☑ 1458.8179 (1+)																				
☑ 1500.8292 (1+)																				
☑ 1502.9252 (1+)																				
☑ 1519.8391 (1+)		●																		
☑ 1524.9093 (1+)																				
☑ 1537.8198 (1+)																				
☑ 1541.8290 (1+)																				
☑ 1553.8132 (1+)		●																		
☑ 1563.9608 (1+)		●																		
☑ 1575.7971 (1+)																				
☑ 1585.8972 (1+)																				
☑ 1601.8958 (1+)																				
☑ 1607.8864 (1+)																				
☑ 1646.9825 (1+)																				
☑ 1660.0651 (1+)						●														
☑ 1678.0089 (1+)	●																			
☑ 1699.9891 (1+)																				
☑ 1712.9164 (1+)																				
☑ 1720.0601 (1+)		●																		
☑ 1729.0091 (1+)																				
☑ 1752.0201 (1+)							●													
☑ 1763.0879 (1+)	●																			
☑ 1774.0116 (1+)																				
☑ 1781.1189 (1+)																				
☑ 1785.0645 (1+)																				
☑ 1795.9856 (1+)																				
☑ 1801.0059 (1+)									●											
☑ 1810.0564 (1+)																				
☑ 1822.9563 (1+)																				
☑ 1841.9975 (1+)					●															
☑ 1867.0842 (1+)																				
☑ 1880.1187 (1+)							●													
☑ 1899.0401 (1+)																				
☑ 1908.0992 (1+)																				
☑ 1921.0358 (1+)																				
☑ 1930.0892 (1+)																				
☑ 1935.1114 (1+)		●																		
☑ 1946.0591 (1+)																				
☑ 1958.1835 (1+)									●											
☑ 1984.1510 (1+)																				
☑ 2036.2558 (1+)																				
☑ 2055.1467 (1+)																				
☑ 2073.1601 (1+)		●																		
☑ 2079.1208 (1+)																				
☑ 2089.1536 (1+)																				
☑ 2095.1397 (1+)																				
☑ 2190.1634 (1+)			●																	
☑ 2202.1890 (1+)																				
☑ 2260.2512 (1+)	●													●						
☑ 2276.2460 (1+)																				
☑ 2282.2311 (1+)																				
☑ 2346.2722 (1+)																				
☑ 2351.2395 (1+)				●																
☑ 2716.4936 (1+)																				
☑ 2807.4071 (1+)																				
☑ 2834.4950 (1+)																				
☑ 2851.4980 (1+)																				

☑ 2935.6509 (1+)	
☑ 3204.7301 (1+)	
☑ 3411.8335 (1+)	●

Select All | Select None | Search Selected | ☐ **Error tolerant** | Archive Report

1. ATPB_YEAST **Mass:** 54817 **Score:** 475 **Matches:** 8(7) **Sequences:** 8(7)
ATP synthase subunit beta, mitochondrial OS=Saccharomyces cerevisiae (strain ATCC 204508 / S288c) GN=ATP2 PE=1 SV=2
☐ Check to include this hit in error tolerant search or archive report

Query	Observed	Mr(expt)	Mr(calc)	ppm	Miss	Score	Expect	Rank	Unique	Peptide
2	1116.6917	1115.6845	1115.6339	45.3	0	47	0.00013	1	U	K.VVDLLAPYAR.G
11	1366.8267	1365.8194	1365.7616	42.3	0	40	0.00052	1	U	K.VLDTGGPISVPVGR.E
18	1420.7653	1419.7580	1419.6895	48.2	0	89	2.1e-008	1	U	K.AHGGFSVFTGVGER.T
19	1435.8252	1434.8180	1434.7467	49.7	0	70	1.1e-006	1	U	R.FTQAGSEVSALLGR.I
23	1453.8709	1452.8636	1452.7977	45.4	0	9	0.74	1	U	R.VALTGLTIAEYFR.D
40	1678.0089	1677.0016	1676.9210	48.1	0	113	1.9e-011	1	U	K.LVLEVAQHLGENTVR.T
46	1763.0879	1762.0806	1761.9989	46.4	1	74	6.1e-008	1	U	R.IINVIGEPIDERGPIK.S
73	2260.2512	2259.2440	2259.1569	38.5	0	159	1.1e-015	1	U	R.IPSAVGYQPTLATDMGLLQER.I

2. ATPA_YEAST **Mass:** 58629 **Score:** 414 **Matches:** 8(6) **Sequences:** 8(6)
ATP synthase subunit alpha, mitochondrial OS=Saccharomyces cerevisiae (strain ATCC 204508 / S288c) GN=ATP1 PE=1 SV=5
☐ Check to include this hit in error tolerant search or archive report

Query	Observed	Mr(expt)	Mr(calc)	ppm	Miss	Score	Expect	Rank	Unique	Peptide
9	1325.7435	1324.7362	1324.6735	47.4	0	20	0.1	1	U	K.SNHNELLTEIR.E
28	1519.8391	1518.8318	1518.7606	46.9	0	16	0.37	1	U	R.IGEFESSFLSYLK.S
32	1553.8132	1552.8059	1552.7310	48.2	0	77	2.1e-007	1	U	R.EAYPGDVFYLHSR.L
33	1563.9608	1562.9535	1562.8781	48.3	0	82	1.6e-008	1	U	R.TGNIVDVPVGPGLLGR.V
43	1720.0601	1719.0529	1718.9792	42.9	1	29	0.0025	1	U	K.RTGNIVDVPVGPGLLGR.V
61	1935.1114	1934.1041	1934.0221	42.4	1	89	8.5e-009	1	U	R.VVDALGNPIDGKGPIDAAGR.S
67	2073.1601	2072.1529	2072.0725	38.8	0	70	7.8e-007	1	U	K.GMALNLEPGQVGIVLFGSDR.L
84	3411.8335	3410.8262	3410.7106	33.9	0	151	4.3e-015	1	U	K.QNQYSPLATEEQVPLIYAGVNGHLDGIELSR.I

3. ATP7_YEAST **Mass:** 19797 **Score:** 129 **Matches:** 1(1) **Sequences:** 1(1)
ATP synthase subunit d, mitochondrial OS=Saccharomyces cerevisiae (strain ATCC 204508 / S288c) GN=ATP7 PE=1 SV=2
☐ Check to include this hit in error tolerant search or archive report

Query	Observed	Mr(expt)	Mr(calc)	ppm	Miss	Score	Expect	Rank	Unique	Peptide
71	2190.1634	2189.1561	2189.0753	36.9	0	129	1.9e-012	1	U	R.QLLELQSQPTEVDFSHYR.S

4. ATPG_YEAST **Mass:** 34443 **Score:** 85 **Matches:** 1(1) **Sequences:** 1(1)
ATP synthase subunit gamma, mitochondrial OS=Saccharomyces cerevisiae (strain ATCC 204508 / S288c) GN=ATP3 PE=1 SV=1
☐ Check to include this hit in error tolerant search or archive report

Query	Observed	Mr(expt)	Mr(calc)	ppm	Miss	Score	Expect	Rank	Unique	Peptide
77	2351.2395	2350.2323	2350.1441	37.5	1	85	4.5e-008	1	U	K.TIEQSPSFGKFEIDTDANVPR.D

5. QCR1_YEAST **Mass:** 50254 **Score:** 35 **Matches:** 1(1) **Sequences:** 1(1)
Cytochrome b-c1 complex subunit 1, mitochondrial OS=Saccharomyces cerevisiae (strain ATCC 204508 / S288c) GN=COR1 PE=1 SV=1
☐ Check to include this hit in error tolerant search or archive report

Query	Observed	Mr(expt)	Mr(calc)	ppm	Miss	Score	Expect	Rank	Unique	Peptide
54	1841.9975	1840.9903	1840.9108	43.2	0	35	0.004	1	U	K.LAAQIFGSYNAFEPASR.L

6. ATPO_YEAST **Mass:** 22857 **Score:** 28 **Matches:** 1(1) **Sequences:** 1(1)
ATP synthase subunit 5, mitochondrial OS=Saccharomyces cerevisiae (strain ATCC 204508 / S288c) GN=ATP5 PE=1 SV=1
☐ Check to include this hit in error tolerant search or archive report

Query	Observed	Mr(expt)	Mr(calc)	ppm	Miss	Score	Expect	Rank	Unique	Peptide
39	1660.0651	1659.0578	1658.9831	45.0	1	28	0.0015	1	U	K.LGHLLLNPALSLKDR.N

7. ATP5E_YEAST **Mass:** 6738 **Score:** 27 **Matches:** 2(1) **Sequences:** 2(1)
ATP synthase subunit epsilon, mitochondrial OS=Saccharomyces cerevisiae (strain ATCC 204508 / S288c) GN=ATP15 PE=1 SV=2
☐ Check to include this hit in error tolerant search or archive report

Query	Observed	Mr(expt)	Mr(calc)	ppm	Miss	Score	Expect	Rank	Unique	Peptide
45	1752.0201	1751.0129	1750.9366	43.6	0	27	0.014	1	U	K.AGISYAAYLNVAAQAIR.S
56	1880.1187	1879.1114	1879.0315	42.5	1	3	2.1	1	U	R.KAGISYAAYLNVAAQAIR.S

8. YJ94_YEAST **Mass:** 50258 **Score:** 0 **Matches:** 1(0) **Sequences:** 1(0)
Uncharacterized membrane protein YJR124C OS=Saccharomyces cerevisiae (strain ATCC 204508 / S288c) GN=YJR124C PE=1 SV=1
☐ Check to include this hit in error tolerant search or archive report

Query	Observed	Mr(expt)	Mr(calc)	ppm	Miss	Score	Expect	Rank	Unique	Peptide
1	1104.6168	1103.6095	1103.6491	-35.89	0	13	0.84	1	U	K.LLWASVFLR.L

9. VDAC1_YEAST **Mass:** 30524 **Score:** 0 **Matches:** 2(0) **Sequences:** 2(0)
Mitochondrial outer membrane protein porin 1 OS=Saccharomyces cerevisiae (strain ATCC 204508 / S288c) GN=POR1 PE=1 SV=4
☐ Check to include this hit in error tolerant search or archive report

Query	Observed	Mr(expt)	Mr(calc)	ppm	Miss	Score	Expect	Rank	Unique	Peptide
51	**1801.0059**	**1799.9986**	**1799.9207**	**43.3**	**0**	**9**	**1.3**	**1**	**U**	**K.SAVLNTTFTQPFFTAR.G**
63	**1958.1835**	**1957.1762**	**1957.0997**	**39.1**	**0**	**3**	**1.4**	**1**	**U**	**K.QLLRPGVTLGVGSSFDALK.L**

---

10. SPT6_YEAST **Score:** 0 **Matches:** 1(0) **Sequences:** 1(0)
Transcription elongation factor SPT6 OS=Saccharomyces cerevisiae (strain ATCC 204508 / S288c) GN=SPT6 PE=1 SV=1
Check to include this hit in error tolerant search or archive report

Query	Observed	Mr(expt)	Mr(calc)	ppm	Miss	Score	Expect	Rank	Unique	Peptide
1	1104.6168	1103.6095	1103.5757	30.6	0	12	1	2	U	K.ILSLTCGQGR.F

---

11. MGR3L_YEAST **Mass:** 54739 **Score:** 0 **Matches:** 1(0) **Sequences:** 1(0)
Protein MRG3-like OS=Saccharomyces cerevisiae (strain ATCC 204508 / S288c) GN=YKL133C PE=3 SV=2
Check to include this hit in error tolerant search or archive report

Query	Observed	Mr(expt)	Mr(calc)	ppm	Miss	Score	Expect	Rank	Unique	Peptide
1	1104.6168	1103.6095	1103.6121	-2.31	1	10	1.5	3	U	K.AIRISEMIR.F + Oxidation (M)

---

12. CCA1_YEAST **Mass:** 62731 **Score:** 0 **Matches:** 1(0) **Sequences:** 1(0)
CCA tRNA nucleotidyltransferase, mitochondrial OS=Saccharomyces cerevisiae (strain ATCC 204508 / S288c) GN=CCA1 PE=1 SV=1
Check to include this hit in error tolerant search or archive report

Query	Observed	Mr(expt)	Mr(calc)	ppm	Miss	Score	Expect	Rank	Unique	Peptide
1	1104.6168	1103.6095	1103.5611	43.9	0	9	1.9	4	U	K.QTFLDDPLR.V

---

13. HXKG_YEAST **Score:** 0 **Matches:** 1(0) **Sequences:** 1(0)
Glucokinase-1 OS=Saccharomyces cerevisiae (strain ATCC 204508 / S288c) GN=GLK1 PE=1 SV=1
Check to include this hit in error tolerant search or archive report

Query	Observed	Mr(expt)	Mr(calc)	ppm	Miss	Score	Expect	Rank	Unique	Peptide
18	1420.7653	1419.7580	1419.7470	7.78	0	9	2.1	2	U	R.HALALSPLGAEGER.K

---

14. RPB1_YEAST **Score:** 0 **Matches:** 1(0) **Sequences:** 1(0)
DNA-directed RNA polymerase II subunit RPB1 OS=Saccharomyces cerevisiae (strain ATCC 204508 / S288c) GN=RPO21 PE=1 SV=2
Check to include this hit in error tolerant search or archive report

Query	Observed	Mr(expt)	Mr(calc)	ppm	Miss	Score	Expect	Rank	Unique	Peptide
73	2260.2512	2259.2440	2259.1528	40.3	1	8	1.6	2	U	K.IENTMLENITLRGVENIER.V + Oxidation (M)

---

15. SNF2_YEAST **Score:** 0 **Matches:** 1(0) **Sequences:** 1(0)
Transcription regulatory protein SNF2 OS=Saccharomyces cerevisiae (strain ATCC 204508 / S288c) GN=SNF2 PE=1 SV=1
Check to include this hit in error tolerant search or archive report

Query	Observed	Mr(expt)	Mr(calc)	ppm	Miss	Score	Expect	Rank	Unique	Peptide
1	1104.6168	1103.6095	1103.5571	47.5	1	6	3.7	5	U	K.SDERSELLR.L

---

16. GYP6_YEAST **Score:** 0 **Matches:** 1(0) **Sequences:** 1(0)
GTPase-activating protein GYP6 OS=Saccharomyces cerevisiae (strain ATCC 204508 / S288c) GN=GYP6 PE=1 SV=2
Check to include this hit in error tolerant search or archive report

Query	Observed	Mr(expt)	Mr(calc)	ppm	Miss	Score	Expect	Rank	Unique	Peptide
1	1104.6168	1103.6095	1103.6604	-46.06	1	6	3.9	6	U	R.WTRLLFLR.E

---

17. RS25A_YEAST **Mass:** 12032 **Score:** 0 **Matches:** 1(0) **Sequences:** 1(0)
40S ribosomal protein S25-A OS=Saccharomyces cerevisiae (strain ATCC 204508 / S288c) GN=RPS25A PE=1 SV=1
Check to include this hit in error tolerant search or archive report

Query	Observed	Mr(expt)	Mr(calc)	ppm	Miss	Score	Expect	Rank	Unique	Peptide
12	**1367.8199**	**1366.8126**	**1366.8772**	**-47.24**	**2**	**5**	**1.6**	**1**	**U**	**R.LKIGGSLARIALR.H**

Proteins matching the same set of peptides:
RS25B_YEAST **Mass:** 12002 **Score:** 0 **Matches:** 1(0) **Sequences:** 1(0)
40S ribosomal protein S25-B OS=Saccharomyces cerevisiae (strain ATCC 204508 / S288c) GN=RPS25B PE=1 SV=1

---

18. ELG1_YEAST **Score:** 0 **Matches:** 1(0) **Sequences:** 1(0)
Telomere length regulation protein ELG1 OS=Saccharomyces cerevisiae (strain ATCC 204508 / S288c) GN=ELG1 PE=1 SV=1
Check to include this hit in error tolerant search or archive report

Query	Observed	Mr(expt)	Mr(calc)	ppm	Miss	Score	Expect	Rank	Unique	Peptide
1	1104.6168	1103.6095	1103.5934	14.6	1	4	5.7	7	U	K.SSLLNERASK.I

---

19. SPT2_YEAST **Score:** 0 **Matches:** 1(0) **Sequences:** 1(0)
Protein SPT2 OS=Saccharomyces cerevisiae (strain ATCC 204508 / S288c) GN=SPT2 PE=1 SV=1
Check to include this hit in error tolerant search or archive report

Query	Observed	Mr(expt)	Mr(calc)	ppm	Miss	Score	Expect	Rank	Unique	Peptide
9	1325.7435	1324.7362	1324.7173	14.3	1	4	4.1	2	U	-.MSFLSKLSQIR.K + Oxidation (M)

---

20. MRT4_YEAST Score: 0 Matches: 1(0) Sequences: 1(0)
mRNA turnover protein 4 OS=Saccharomyces cerevisiae (strain ATCC 204508 / S288c) GN=MRT4 PE=1 SV=1
☐ Check to include this hit in error tolerant search or archive report

Query	Observed	Mr(expt)	Mr(calc)	ppm	Miss	Score	Expect	Rank	Unique	Peptide
2	1116.6917	1115.6845	1115.6550	26.4	1	4	2.6	2	U	K.LVTLAQTDKK.G

Peptide matches not assigned to protein hits: (no details means no match)

	Query	Observed	Mr(expt)	Mr(calc)	ppm	Miss	Score	Expect	Rank	Unique	Peptide
☑	75	2282.2311	2281.2239	2281.2866	-27.50	2	4	4.5	1		AASSINRVDTIRVPTLVINSR
☑	20	1438.9222	1437.9149	1437.9031	8.21	2	4	0.44	1		RLTPVLIKEITR
☑	30	1537.8198	1536.8125	1536.8195	-4.55	1	0	14	1		CHVQLPTTKNALR
☑	3	1130.6974	1129.6901								
☑	4	1138.6745	1137.6672								
☑	5	1227.7187	1226.7114								
☑	6	1246.7559	1245.7487								
☑	7	1269.7374	1268.7302								
☑	8	1297.6784	1296.6712								
☑	10	1346.7575	1345.7503								
☑	13	1372.7843	1371.7771								
☑	14	1383.8222	1382.8149								
☑	15	1402.7971	1401.7898								
☑	16	1410.9062	1409.8990								
☑	17	1413.7663	1412.7590								
☑	21	1442.8860	1441.8787								
☑	22	1445.8207	1444.8134								
☑	24	1457.7951	1456.7879								
☑	25	1458.8179	1457.8106								
☑	26	1500.8292	1499.8220								
☑	27	1502.9252	1501.9179								
☑	29	1524.9093	1523.9020								
☑	31	1541.8290	1540.8217								
☑	34	1575.7971	1574.7898								
☑	35	1585.8972	1584.8900								
☑	36	1601.8958	1600.8885								
☑	37	1607.8864	1606.8791								
☑	38	1646.9825	1645.9753								
☑	41	1699.9891	1698.9818								
☑	42	1712.9164	1711.9091								
☑	44	1729.0091	1728.0018								
☑	47	1774.0116	1773.0043								
☑	48	1781.1189	1780.1116								
☑	49	1785.0645	1784.0573								
☑	50	1795.9856	1794.9783								
☑	52	1810.0564	1809.0492								
☑	53	1822.9563	1821.9490								
☑	55	1867.0842	1866.0769								
☑	57	1899.0401	1898.0328								
☑	58	1908.0992	1907.0919								
☑	59	1921.0358	1920.0286								
☑	60	1930.0892	1929.0819								
☑	62	1946.0591	1945.0519								
☑	64	1984.1510	1983.1438								
☑	65	2036.2558	2035.2485								
☑	66	2055.1467	2054.1394								
☑	68	2079.1208	2078.1136								
☑	69	2089.1536	2088.1463								
☑	70	2095.1397	2094.1325								
☑	72	2202.1890	2201.1817								
☑	74	2276.2460	2275.2387								
☑	76	2346.2722	2345.2649								
☑	78	2716.4936	2715.4863								
☑	79	2807.4071	2806.3998								
☑	80	2834.4950	2833.4877								
☑	81	2851.4980	2850.4907								
☑	82	2935.6509	2934.6436								
☑	83	3204.7301	3203.7228								

Search Parameters

```
Type of search         : MS/MS Ion Search
Enzyme                 : Trypsin
Fixed modifications    : Carbamidomethyl (C)
Variable modifications : Oxidation (M)
Mass values            : Monoisotopic
Protein Mass           : Unrestricted
Peptide Mass Tolerance : ± 50 ppm
Fragment Mass Tolerance: ± 0.5 Da
Max Missed Cleavages   : 2
Instrument type        : Default
Number of queries      : 84
```

Mascot Search Results

```
User          :
Email         :
Search title  :
MS data file  : DATA.TXT
Database      : Scerevisiae_PubMed PubMed_210915 (686171 sequences; 329036123 residues)
Timestamp     : 23 Nov 2016 at 13:04:27 GMT
Protein hits  : pdb|1EZV|A       Chain A, Structure Of The Yeast Cytochrome Bc1 Complex Co- Crystallized With An Antibody Fv-Fragment
                pdb|1EZV|B       Chain B, Structure Of The Yeast Cytochrome Bc1 Complex Co- Crystallized With An Antibody Fv-Fragment
                pdb|1KYO|D       Chain D, Yeast Cytochrome Bc1 Complex With Bound Substrate Cytochrome C
                pdb|1EZV|F       Chain F, Structure Of The Yeast Cytochrome Bc1 Complex Co- Crystallized With An Antibody Fv-Fragment
                pdb|2HLD|D       Chain D, Crystal Structure Of Yeast Mitochondrial F1-Atpase
                gb|AJU16346.1|   Cox6p [Saccharomyces cerevisiae YJM1356]
                gb|AKB01032.1|   Cox5ap [Saccharomyces cerevisiae YJM1434]
                pdb|1EZV|E       Chain E, Structure Of The Yeast Cytochrome Bc1 Complex Co- Crystallized With An Antibody Fv-Fragment
                emb|CCO02527.1|  cytochrome oxidase subunit 2, partial (mitochondrion) [Saccharomyces cerevisiae]
                gb|AJS00152.1|   Cox13p [Saccharomyces cerevisiae YJM1433]
                gb|AJT00070.1|   Cox7p [Saccharomyces cerevisiae YJM1573]
                emb|CAR57987.1|  unnamed protein product [Candida glabrata]
                pdb|2HLD|A       Chain A, Crystal Structure Of Yeast Mitochondrial F1-Atpase
                emb|CAF06149.1|  related to Pol II transcription elongation factor [Neurospora crassa]
                emb|CCA61330.1|  ER membrane localized phosphoryltransferase [Saccharomyces uvarum]
                pdb|1AY0|A       Chain A, Identification Of Catalytically Important Residues In Yeast Transketolase
                dbj|BAA14841.2|  enoyl-[acyl-carrier-protein] reductase, NADH-dependent [Escherichia coli str. K-12 substr. W3110]
                emb|CAR57985.1|  unnamed protein product [Candida glabrata]
                dbj|BAC56234.1|  putative SSK22 like MAPKK kinase [Neurospora crassa]
                emb|CAR28930.1|  ZYRO0F15268p [Zygosaccharomyces rouxii]
```

Mascot Score Histogram

Ions score is -10*Log(P), where P is the probability that the observed match is a random event.
Individual ions scores > 44 indicate identity or extensive homology (p<0.05).
Protein scores are derived from ions scores as a non-probabilistic basis for ranking protein hits.

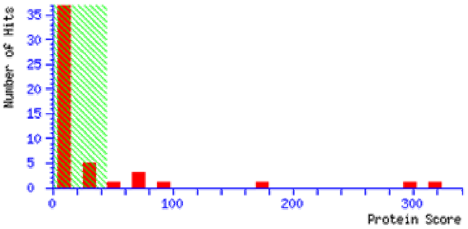

Peptide Summary Report

Format As | Peptide Summary | Help

Significance threshold p< 0.05 | Max. number of hits 20

Standard scoring ◉ MudPIT scoring ○ | Ions score or expect cut-off 0 | Show sub-sets 0

Show pop-ups ◉ Suppress pop-ups ○ | Sort unassigned Decreasing Score | Require bold red ☐

Overview Table

Click on column header to jump to entry in results list.
Move mouse over any indicator to highlight identical peptides.
Click on an indicator to see details of individual match.
Use check boxes to select sub-set of queries for new search.

Mouse over: -Query-
-Accession- -Sequence-

Hit:	1	2	3	4	5	6	7	8	9	10	11	12	13	14	15	16	17	18	19	20
☑ 1101.6886 (1+)			●																	
☑ 1116.7099 (1+)																				
☑ 1130.7178 (1+)								●												
☑ 1141.7389 (1+)																				
☑ 1152.7009 (1+)																				
☑ 1208.7621 (1+)																				
☑ 1245.7526 (1+)										●										
☑ 1266.7371 (1+)		●																		
☑ 1297.6991 (1+)	●														●					●
☑ 1338.8118 (1+)																				
☑ 1346.7832 (1+)																				
☑ 1356.7451 (1+)																●				
☑ 1362.7925 (1+)																				
☑ 1366.8490 (1+)																				
☑ 1372.8112 (1+)		●																	●	
☑ 1394.7938 (1+)																				

☑ 1402.8234 (1+)		●																		
☑ 1405.9084 (1+)																				
☑ 1413.7942 (1+)																				
☑ 1420.7913 (1+)																				
☑ 1435.8388 (1+)					●															
☑ 1438.9404 (1+)																				
☑ 1498.8679 (1+)																				
☑ 1507.7557 (1+)																				
☑ 1510.9030 (1+)																				
☑ 1530.9209 (1+)		●																		
☑ 1537.8484 (1+)																				
☑ 1541.8912 (1+)														●			●	●		
☑ 1548.9292 (1+)											●	●								
☑ 1555.8383 (1+)																				
☑ 1557.9539 (1+)																				
☑ 1563.9829 (1+)													●							
☑ 1567.9141 (1+)																				
☑ 1570.9155 (1+)																				
☑ 1585.9159 (1+)					●															
☑ 1589.9001 (1+)																				
☑ 1628.9967 (1+)																				
☑ 1670.0910 (1+)																				
☑ 1678.0309 (1+)					●															
☑ 1703.9990 (1+)																				
☑ 1711.9300 (1+)							●													
☑ 1727.9225 (1+)																				
☑ 1729.0373 (1+)		●																		
☑ 1733.9175 (1+)																				
☑ 1743.9614 (1+)																				
☑ 1747.0132 (1+)																				
☑ 1752.0388 (1+)																				
☑ 1763.1123 (1+)																				
☑ 1764.9726 (1+)																				
☑ 1768.9906 (1+)																				
☑ 1781.1490 (1+)																				
☑ 1790.9829 (1+)			●																	
☑ 1801.0359 (1+)																				
☑ 1812.9982 (1+)																				
☑ 1822.9920 (1+)																				
☑ 1842.0300 (1+)	●																			
☑ 1864.0121 (1+)																				
☑ 1867.1181 (1+)		●																		
☑ 1889.0990 (1+)																				
☑ 1901.0738 (1+)		●																		
☑ 1912.0969 (1+)									●											
☑ 1931.0702 (1+)																				
☑ 1935.1382 (1+)																				
☑ 1939.1585 (1+)																				
☑ 1958.2170 (1+)																				
☑ 1976.0935 (1+)				●																
☑ 2044.1251 (1+)																				
☑ 2074.2043 (1+)																				
☑ 2081.0684 (1+)						●														
☑ 2119.0358 (1+)			●																	
☑ 2183.1657 (1+)																				
☑ 2191.1320 (1+)																				
☑ 2201.2418 (1+)																				
☑ 2261.3203 (1+)	●																			
☑ 2351.2742 (1+)																				
☑ 2387.3799 (1+)																				
☑ 2456.3299 (1+)																				
☑ 2464.3910 (1+)																				
☑ 2478.3526 (1+)																				
☑ 2530.3976 (1+)																				
☑ 2552.3880 (1+)																				
☑ 2591.4719 (1+)																				
☑ 2713.4115 (1+)																				

☑ 2748.4453 (1+)																				
☑ 2783.3997 (1+)																				
☑ 2790.5867 (1+)																				
☑ 2807.4466 (1+)																				

Select All | Select None | Search Selected | ☐ **Error tolerant** | Archive Report

1. pdb|1EZV|A **Mass:** 47336 **Score:** 317 **Matches:** 3(3) **Sequences:** 3(3)
Chain A, Structure Of The Yeast Cytochrome Bc1 Complex Co- Crystallized With An Antibody Fv-Fragment
☐ Check to include this hit in error tolerant search or archive report

Query	Observed	Mr(expt)	Mr(calc)	ppm	Miss	Score	Expect	Rank	Unique	Peptide
9	1297.6991	1296.6918	1296.6099	63.2	0	74	8.1e-005	1	U	K.DSGLWGFSTATR.N
56	1842.0300	1841.0227	1840.9108	60.8	0	118	2.4e-009	1	U	K.LAAQIFGSYNAFEPASR.L
74	2261.3203	2260.3131	2260.1964	51.6	0	126	3.6e-010	1	U	R.VLEHLHSTAFQNTPLSLPTR.G

Proteins matching the same set of peptides:
pdb|1KB9|A **Mass:** 47423 **Score:** 317 **Matches:** 3(3) **Sequences:** 3(3)
Chain A, Yeast Cytochrome Bc1 Complex
pdb|1KYO|A **Mass:** 47336 **Score:** 317 **Matches:** 3(3) **Sequences:** 3(3)
Chain A, Yeast Cytochrome Bc1 Complex With Bound Substrate Cytochrome C
pdb|1KYO|L **Mass:** 47336 **Score:** 317 **Matches:** 3(3) **Sequences:** 3(3)
Chain L, Yeast Cytochrome Bc1 Complex With Bound Substrate Cytochrome C
pdb|1P84|A **Mass:** 47423 **Score:** 317 **Matches:** 3(3) **Sequences:** 3(3)
Chain A, Hdbt Inhibited Yeast Cytochrome Bc1 Complex
pdb|2IBZ|A **Mass:** 47423 **Score:** 317 **Matches:** 3(3) **Sequences:** 3(3)
Chain A, Yeast Cytochrome Bc1 Complex With Stigmatellin
pdb|3CX5|A **Mass:** 47423 **Score:** 317 **Matches:** 3(3) **Sequences:** 3(3)
Chain A, Structure Of Complex Iii With Bound Cytochrome C In Reduced State And Definition Of A Minimal Core Interface For Electron Trans
pdb|3CX5|L **Mass:** 47423 **Score:** 317 **Matches:** 3(3) **Sequences:** 3(3)
Chain L, Structure Of Complex Iii With Bound Cytochrome C In Reduced State And Definition Of A Minimal Core Interface For Electron Trans
pdb|3CXH|A **Mass:** 47423 **Score:** 317 **Matches:** 3(3) **Sequences:** 3(3)
Chain A, Structure Of Yeast Complex Iii With Isoform-2 Cytochrome C Bound And Definition Of A Minimal Core Interface For Electron Transf
pdb|3CXH|L **Mass:** 47423 **Score:** 317 **Matches:** 3(3) **Sequences:** 3(3)
Chain L, Structure Of Yeast Complex Iii With Isoform-2 Cytochrome C Bound And Definition Of A Minimal Core Interface For Electron Transf
pdb|4PD4|A **Mass:** 47423 **Score:** 317 **Matches:** 3(3) **Sequences:** 3(3)
Chain A, Structural Analysis Of Atovaquone-inhibited Cytochrome Bc1 Complex Reveals The Molecular Basis Of Antimalarial Drug Action
gb|AJQ00347.1| **Mass:** 50268 **Score:** 317 **Matches:** 3(3) **Sequences:** 3(3)
Cor1p [Saccharomyces cerevisiae YJM244]
gb|AJQ00736.1| **Mass:** 50254 **Score:** 317 **Matches:** 3(3) **Sequences:** 3(3)
Cor1p [Saccharomyces cerevisiae YJM248]
gb|AJQ01120.1| **Mass:** 50298 **Score:** 317 **Matches:** 3(3) **Sequences:** 3(3)
Cor1p [Saccharomyces cerevisiae YJM270]
gb|AJQ01505.1| **Mass:** 50254 **Score:** 317 **Matches:** 3(3) **Sequences:** 3(3)
Cor1p [Saccharomyces cerevisiae YJM271]
gb|AJQ01890.1| **Mass:** 50254 **Score:** 317 **Matches:** 3(3) **Sequences:** 3(3)
Cor1p [Saccharomyces cerevisiae YJM320]
gb|AJQ02278.1| **Mass:** 50254 **Score:** 317 **Matches:** 3(3) **Sequences:** 3(3)
Cor1p [Saccharomyces cerevisiae YJM326]
gb|AJQ02668.1| **Mass:** 50254 **Score:** 317 **Matches:** 3(3) **Sequences:** 3(3)
Cor1p [Saccharomyces cerevisiae YJM428]
gb|AJQ03055.1| **Mass:** 50254 **Score:** 317 **Matches:** 3(3) **Sequences:** 3(3)
Cor1p [Saccharomyces cerevisiae YJM450]
gb|AJQ03426.1| **Mass:** 50254 **Score:** 317 **Matches:** 3(3) **Sequences:** 3(3)
Cor1p [Saccharomyces cerevisiae YJM451]
gb|AJQ03810.1| **Mass:** 50254 **Score:** 317 **Matches:** 3(3) **Sequences:** 3(3)
Cor1p [Saccharomyces cerevisiae YJM453]
gb|AJQ04158.1| **Mass:** 50254 **Score:** 317 **Matches:** 3(3) **Sequences:** 3(3)
Cor1p [Saccharomyces cerevisiae YJM456]
gb|AJQ04544.1| **Mass:** 50254 **Score:** 317 **Matches:** 3(3) **Sequences:** 3(3)
Cor1p [Saccharomyces cerevisiae YJM470]
gb|AJQ04925.1| **Mass:** 50254 **Score:** 317 **Matches:** 3(3) **Sequences:** 3(3)
Cor1p [Saccharomyces cerevisiae YJM541]
gb|AJQ05311.1| **Mass:** 50254 **Score:** 317 **Matches:** 3(3) **Sequences:** 3(3)
Cor1p [Saccharomyces cerevisiae YJM554]
gb|AJQ05697.1| **Mass:** 50254 **Score:** 317 **Matches:** 3(3) **Sequences:** 3(3)
Cor1p [Saccharomyces cerevisiae YJM555]
gb|AJQ06083.1| **Mass:** 50254 **Score:** 317 **Matches:** 3(3) **Sequences:** 3(3)
Cor1p [Saccharomyces cerevisiae YJM627]
gb|AJQ06470.1| **Mass:** 50254 **Score:** 317 **Matches:** 3(3) **Sequences:** 3(3)
Cor1p [Saccharomyces cerevisiae YJM681]
gb|AJQ06834.1| **Mass:** 50254 **Score:** 317 **Matches:** 3(3) **Sequences:** 3(3)
Cor1p [Saccharomyces cerevisiae YJM682]
gb|AJQ07217.1| **Mass:** 50254 **Score:** 317 **Matches:** 3(3) **Sequences:** 3(3)
Cor1p [Saccharomyces cerevisiae YJM683]
gb|AJQ07605.1| **Mass:** 50254 **Score:** 317 **Matches:** 3(3) **Sequences:** 3(3)
Cor1p [Saccharomyces cerevisiae YJM689]
gb|AJQ07990.1| **Mass:** 50254 **Score:** 317 **Matches:** 3(3) **Sequences:** 3(3)
Cor1p [Saccharomyces cerevisiae YJM1252]
gb|AJQ08374.1| **Mass:** 50254 **Score:** 317 **Matches:** 3(3) **Sequences:** 3(3)
Cor1p [Saccharomyces cerevisiae YJM1273]
gb|AJQ08763.1| **Mass:** 50254 **Score:** 317 **Matches:** 3(3) **Sequences:** 3(3)
Cor1p [Saccharomyces cerevisiae YJM1304]
gb|AJQ09148.1| **Mass:** 50284 **Score:** 317 **Matches:** 3(3) **Sequences:** 3(3)
Cor1p [Saccharomyces cerevisiae YJM1307]
gb|AJQ09532.1| **Mass:** 50254 **Score:** 317 **Matches:** 3(3) **Sequences:** 3(3)

Cor1p [Saccharomyces cerevisiae YJM1311]
gb|AJQ09919.1| **Mass:** 50238 **Score:** 317 **Matches:** 3(3) **Sequences:** 3(3)
Cor1p [Saccharomyces cerevisiae YJM1326]
gb|AJQ10306.1| **Mass:** 50240 **Score:** 317 **Matches:** 3(3) **Sequences:** 3(3)
Cor1p [Saccharomyces cerevisiae YJM1332]
gb|AJQ10692.1| **Mass:** 50268 **Score:** 317 **Matches:** 3(3) **Sequences:** 3(3)
Cor1p [Saccharomyces cerevisiae YJM1336]
gb|AJQ11041.1| **Mass:** 50254 **Score:** 317 **Matches:** 3(3) **Sequences:** 3(3)
Cor1p [Saccharomyces cerevisiae YJM1338]
gb|AJQ11427.1| **Mass:** 50268 **Score:** 317 **Matches:** 3(3) **Sequences:** 3(3)
Cor1p [Saccharomyces cerevisiae YJM1341]
gb|AJQ11813.1| **Mass:** 50254 **Score:** 317 **Matches:** 3(3) **Sequences:** 3(3)
Cor1p [Saccharomyces cerevisiae YJM1342]
gb|AJQ12193.1| **Mass:** 50272 **Score:** 317 **Matches:** 3(3) **Sequences:** 3(3)
Cor1p [Saccharomyces cerevisiae YJM1355]
gb|AJQ12575.1| **Mass:** 50254 **Score:** 317 **Matches:** 3(3) **Sequences:** 3(3)
Cor1p [Saccharomyces cerevisiae YJM1356]
gb|AJQ12962.1| **Mass:** 50290 **Score:** 317 **Matches:** 3(3) **Sequences:** 3(3)
Cor1p [Saccharomyces cerevisiae YJM1381]
gb|AJQ13347.1| **Mass:** 50268 **Score:** 317 **Matches:** 3(3) **Sequences:** 3(3)
Cor1p [Saccharomyces cerevisiae YJM1383]
gb|AJQ13735.1| **Mass:** 50290 **Score:** 317 **Matches:** 3(3) **Sequences:** 3(3)
Cor1p [Saccharomyces cerevisiae YJM1385]
gb|AJQ14118.1| **Mass:** 50254 **Score:** 317 **Matches:** 3(3) **Sequences:** 3(3)
Cor1p [Saccharomyces cerevisiae YJM1386]
gb|AJQ14504.1| **Mass:** 50254 **Score:** 317 **Matches:** 3(3) **Sequences:** 3(3)
Cor1p [Saccharomyces cerevisiae YJM1387]
gb|AJQ14889.1| **Mass:** 50312 **Score:** 317 **Matches:** 3(3) **Sequences:** 3(3)
Cor1p [Saccharomyces cerevisiae YJM1388]
gb|AJQ15266.1| **Mass:** 50254 **Score:** 317 **Matches:** 3(3) **Sequences:** 3(3)
Cor1p [Saccharomyces cerevisiae YJM1389]
gb|AJQ15643.1| **Mass:** 50254 **Score:** 317 **Matches:** 3(3) **Sequences:** 3(3)
Cor1p [Saccharomyces cerevisiae YJM1399]
gb|AJQ16024.1| **Mass:** 50254 **Score:** 317 **Matches:** 3(3) **Sequences:** 3(3)
Cor1p [Saccharomyces cerevisiae YJM1400]
gb|AJQ16403.1| **Mass:** 50253 **Score:** 317 **Matches:** 3(3) **Sequences:** 3(3)
Cor1p [Saccharomyces cerevisiae YJM1401]
gb|AAA34508.1| **Mass:** 50254 **Score:** 317 **Matches:** 3(3) **Sequences:** 3(3)
core protein precursor [Saccharomyces cerevisiae]
gb|AJP37043.1| **Mass:** 50254 **Score:** 317 **Matches:** 3(3) **Sequences:** 3(3)
Cor1p [Saccharomyces cerevisiae YJM1078]
emb|CAA55050.1| **Mass:** 50254 **Score:** 317 **Matches:** 3(3) **Sequences:** 3(3)
YBL0403 [Saccharomyces cerevisiae]
gb|AHY74441.1| **Mass:** 50254 **Score:** 317 **Matches:** 3(3) **Sequences:** 3(3)
Cor1p [Saccharomyces cerevisiae YJM993]
emb|CAY77740.1| **Mass:** 50284 **Score:** 317 **Matches:** 3(3) **Sequences:** 3(3)
Cor1p [Saccharomyces cerevisiae EC1118]
gb|AJP81945.1| **Mass:** 50254 **Score:** 317 **Matches:** 3(3) **Sequences:** 3(3)
Cor1p [Saccharomyces cerevisiae YJM1402]
gb|AJP82325.1| **Mass:** 50268 **Score:** 317 **Matches:** 3(3) **Sequences:** 3(3)
Cor1p [Saccharomyces cerevisiae YJM1415]
gb|AJP82713.1| **Mass:** 50254 **Score:** 317 **Matches:** 3(3) **Sequences:** 3(3)
Cor1p [Saccharomyces cerevisiae YJM1417]
gb|AJP83491.1| **Mass:** 50254 **Score:** 317 **Matches:** 3(3) **Sequences:** 3(3)
Cor1p [Saccharomyces cerevisiae YJM1419]
gb|AJP83878.1| **Mass:** 50254 **Score:** 317 **Matches:** 3(3) **Sequences:** 3(3)
Cor1p [Saccharomyces cerevisiae YJM1433]
gb|AJP84253.1| **Mass:** 50254 **Score:** 317 **Matches:** 3(3) **Sequences:** 3(3)
Cor1p [Saccharomyces cerevisiae YJM1434]
gb|AJP84642.1| **Mass:** 50254 **Score:** 317 **Matches:** 3(3) **Sequences:** 3(3)
Cor1p [Saccharomyces cerevisiae YJM1439]
emb|CAA84865.1| **Mass:** 50254 **Score:** 317 **Matches:** 3(3) **Sequences:** 3(3)
COR1 [Saccharomyces cerevisiae]
gb|AJP85024.1| **Mass:** 50254 **Score:** 317 **Matches:** 3(3) **Sequences:** 3(3)
Cor1p [Saccharomyces cerevisiae YJM1443]
gb|AJP85398.1| **Mass:** 50254 **Score:** 317 **Matches:** 3(3) **Sequences:** 3(3)
Cor1p [Saccharomyces cerevisiae YJM1444]
gb|AJP85784.1| **Mass:** 50284 **Score:** 317 **Matches:** 3(3) **Sequences:** 3(3)
Cor1p [Saccharomyces cerevisiae YJM1447]
gb|AJP86157.1| **Mass:** 50268 **Score:** 317 **Matches:** 3(3) **Sequences:** 3(3)
Cor1p [Saccharomyces cerevisiae YJM1450]
gb|AJP86540.1| **Mass:** 50254 **Score:** 317 **Matches:** 3(3) **Sequences:** 3(3)
Cor1p [Saccharomyces cerevisiae YJM1460]
gb|AJP86924.1| **Mass:** 50282 **Score:** 317 **Matches:** 3(3) **Sequences:** 3(3)
Cor1p [Saccharomyces cerevisiae YJM1463]
gb|AJP87309.1| **Mass:** 50268 **Score:** 317 **Matches:** 3(3) **Sequences:** 3(3)
Cor1p [Saccharomyces cerevisiae YJM1477]
gb|AJP87698.1| **Mass:** 50254 **Score:** 317 **Matches:** 3(3) **Sequences:** 3(3)
Cor1p [Saccharomyces cerevisiae YJM1478]
gb|AJP88048.1| **Mass:** 50254 **Score:** 317 **Matches:** 3(3) **Sequences:** 3(3)
Cor1p [Saccharomyces cerevisiae YJM1479]
gb|AJP88428.1| **Mass:** 50254 **Score:** 317 **Matches:** 3(3) **Sequences:** 3(3)
Cor1p [Saccharomyces cerevisiae YJM1526]
gb|AJP88817.1| **Mass:** 50239 **Score:** 317 **Matches:** 3(3) **Sequences:** 3(3)
Cor1p [Saccharomyces cerevisiae YJM1527]
gb|AJP89206.1| **Mass:** 50254 **Score:** 317 **Matches:** 3(3) **Sequences:** 3(3)
Cor1p [Saccharomyces cerevisiae YJM1549]
gb|AJP89589.1| **Mass:** 50254 **Score:** 317 **Matches:** 3(3) **Sequences:** 3(3)

Cor1p [Saccharomyces cerevisiae YJM1573]
gb|AJP89967.1| **Mass:** 50298 **Score:** 317 **Matches:** 3(3) **Sequences:** 3(3)
Cor1p [Saccharomyces cerevisiae YJM1574]
gb|AJP90287.1| **Mass:** 50254 **Score:** 317 **Matches:** 3(3) **Sequences:** 3(3)
Cor1p [Saccharomyces cerevisiae YJM1592]
gb|AJP90672.1| **Mass:** 50254 **Score:** 317 **Matches:** 3(3) **Sequences:** 3(3)
Cor1p [Saccharomyces cerevisiae YJM1615]
gb|AJP91055.1| **Mass:** 50254 **Score:** 317 **Matches:** 3(3) **Sequences:** 3(3)
Cor1p [Saccharomyces cerevisiae YJM693]
gb|AJP91442.1| **Mass:** 50254 **Score:** 317 **Matches:** 3(3) **Sequences:** 3(3)
Cor1p [Saccharomyces cerevisiae YJM969]
gb|AJP91829.1| **Mass:** 50254 **Score:** 317 **Matches:** 3(3) **Sequences:** 3(3)
Cor1p [Saccharomyces cerevisiae YJM972]
gb|AJP92219.1| **Mass:** 50254 **Score:** 317 **Matches:** 3(3) **Sequences:** 3(3)
Cor1p [Saccharomyces cerevisiae YJM975]
gb|AJP92607.1| **Mass:** 50254 **Score:** 317 **Matches:** 3(3) **Sequences:** 3(3)
Cor1p [Saccharomyces cerevisiae YJM978]
gb|AJP92996.1| **Mass:** 50254 **Score:** 317 **Matches:** 3(3) **Sequences:** 3(3)
Cor1p [Saccharomyces cerevisiae YJM981]
gb|AAT93066.1| **Mass:** 50254 **Score:** 317 **Matches:** 3(3) **Sequences:** 3(3)
YBL045C [Saccharomyces cerevisiae]
gb|AJP93386.1| **Mass:** 50254 **Score:** 317 **Matches:** 3(3) **Sequences:** 3(3)
Cor1p [Saccharomyces cerevisiae YJM984]
gb|AJP93775.1| **Mass:** 50254 **Score:** 317 **Matches:** 3(3) **Sequences:** 3(3)
Cor1p [Saccharomyces cerevisiae YJM987]
gb|AJP94165.1| **Mass:** 50254 **Score:** 317 **Matches:** 3(3) **Sequences:** 3(3)
Cor1p [Saccharomyces cerevisiae YJM990]
gb|AJP94556.1| **Mass:** 50254 **Score:** 317 **Matches:** 3(3) **Sequences:** 3(3)
Cor1p [Saccharomyces cerevisiae YJM996]
gb|AJP94945.1| **Mass:** 50254 **Score:** 317 **Matches:** 3(3) **Sequences:** 3(3)
Cor1p [Saccharomyces cerevisiae YJM1083]
gb|AJP95330.1| **Mass:** 50254 **Score:** 317 **Matches:** 3(3) **Sequences:** 3(3)
Cor1p [Saccharomyces cerevisiae YJM1129]
gb|AJP95719.1| **Mass:** 50268 **Score:** 317 **Matches:** 3(3) **Sequences:** 3(3)
Cor1p [Saccharomyces cerevisiae YJM1133]
gb|AJP96106.1| **Mass:** 50314 **Score:** 317 **Matches:** 3(3) **Sequences:** 3(3)
Cor1p [Saccharomyces cerevisiae YJM1190]
gb|AJP96488.1| **Mass:** 50254 **Score:** 317 **Matches:** 3(3) **Sequences:** 3(3)
Cor1p [Saccharomyces cerevisiae YJM1199]
gb|AJP96874.1| **Mass:** 50290 **Score:** 317 **Matches:** 3(3) **Sequences:** 3(3)
Cor1p [Saccharomyces cerevisiae YJM1202]
gb|AJP97257.1| **Mass:** 50254 **Score:** 317 **Matches:** 3(3) **Sequences:** 3(3)
Cor1p [Saccharomyces cerevisiae YJM1208]
gb|AJP97642.1| **Mass:** 50268 **Score:** 317 **Matches:** 3(3) **Sequences:** 3(3)
Cor1p [Saccharomyces cerevisiae YJM1242]
gb|AJP98030.1| **Mass:** 50254 **Score:** 317 **Matches:** 3(3) **Sequences:** 3(3)
Cor1p [Saccharomyces cerevisiae YJM1244]
gb|AJP98417.1| **Mass:** 50254 **Score:** 317 **Matches:** 3(3) **Sequences:** 3(3)
Cor1p [Saccharomyces cerevisiae YJM1248]
gb|AJP98801.1| **Mass:** 50268 **Score:** 317 **Matches:** 3(3) **Sequences:** 3(3)
Cor1p [Saccharomyces cerevisiae YJM1250]
gb|AJP99189.1| **Mass:** 50268 **Score:** 317 **Matches:** 3(3) **Sequences:** 3(3)
Cor1p [Saccharomyces cerevisiae YJM189]
gb|AJP99579.1| **Mass:** 50254 **Score:** 317 **Matches:** 3(3) **Sequences:** 3(3)
Cor1p [Saccharomyces cerevisiae YJM193]
gb|AJP99963.1| **Mass:** 50254 **Score:** 317 **Matches:** 3(3) **Sequences:** 3(3)
Cor1p [Saccharomyces cerevisiae YJM195]

---

2. pdb|1EZV|B **Mass:** 38738 **Score:** 287 **Matches:** 7(2) **Sequences:** 7(2)
Chain B, Structure Of The Yeast Cytochrome Bc1 Complex Co- Crystallized With An Antibody Fv-Fragment
☐ Check to include this hit in error tolerant search or archive report

Query	Observed	Mr(expt)	Mr(calc)	ppm	Miss	Score	Expect	Rank	Unique	Peptide
8	1266.7371	1265.7298	1265.6517	61.8	1	33	0.92	1	U	K.FFLGEENRVR.F
15	1372.8112	1371.8039	1371.7187	62.1	0	31	1.2	1	U	K.FTDGGLFTLFVR.D
17	1402.8234	1401.8161	1401.7252	64.8	0	44	0.072	1	U	K.GLGNPLLYDGVER.V
26	1530.9209	1529.9137	1529.8202	61.1	1	14	56	1	U	R.KGLGNPLLYDGVER.V
43	1729.0373	1728.0300	1727.9247	61.0	1	46	0.032	1	U	K.LDKFTDGGLFTLFVR.D
58	1867.1181	1866.1108	1865.9999	59.4	0	89	1.3e-006	1	U	K.TAFKPHELTESVLPAAR.Y
60	1901.0738	1900.0665	1899.9538	59.4	1	31	1.2	1	U	K.ENLEVSGENVVEADLKR.F

Proteins matching the same set of peptides:
pdb|1KB9|B **Mass:** 38738 **Score:** 287 **Matches:** 7(2) **Sequences:** 7(2)
Chain B, Yeast Cytochrome Bc1 Complex
pdb|1KYO|B **Mass:** 38738 **Score:** 287 **Matches:** 7(2) **Sequences:** 7(2)
Chain B, Yeast Cytochrome Bc1 Complex With Bound Substrate Cytochrome C
pdb|1KYO|M **Mass:** 38738 **Score:** 287 **Matches:** 7(2) **Sequences:** 7(2)
Chain M, Yeast Cytochrome Bc1 Complex With Bound Substrate Cytochrome C
pdb|1P84|B **Mass:** 38738 **Score:** 287 **Matches:** 7(2) **Sequences:** 7(2)
Chain B, Hdbt Inhibited Yeast Cytochrome Bc1 Complex
pdb|2IBZ|B **Mass:** 38738 **Score:** 287 **Matches:** 7(2) **Sequences:** 7(2)
Chain B, Yeast Cytochrome Bc1 Complex With Stigmatellin
pdb|3CX5|B **Mass:** 38738 **Score:** 287 **Matches:** 7(2) **Sequences:** 7(2)
Chain B, Structure Of Complex Iii With Bound Cytochrome C In Reduced State And Definition Of A Minimal Core Interface For Electron Trans
pdb|3CX5|M **Mass:** 38738 **Score:** 287 **Matches:** 7(2) **Sequences:** 7(2)
Chain M, Structure Of Complex Iii With Bound Cytochrome C In Reduced State And Definition Of A Minimal Core Interface For Electron Trans
pdb|3CXH|B **Mass:** 38738 **Score:** 287 **Matches:** 7(2) **Sequences:** 7(2)
Chain B, Structure Of Yeast Complex Iii With Isoform-2 Cytochrome C Bound And Definition Of A Minimal Core Interface For Electron Transf

pdb|3CXH|M **Mass:** 38738 **Score:** 287 **Matches:** 7(2) **Sequences:** 7(2)
Chain M, Structure Of Yeast Complex Iii With Isoform-2 Cytochrome C Bound And Definition Of A Minimal Core Interface For Electron Transf
pdb|4PD4|B **Mass:** 38738 **Score:** 287 **Matches:** 7(2) **Sequences:** 7(2)
Chain B, Structural Analysis Of Atovaquone-inhibited Cytochrome Bc1 Complex Reveals The Molecular Basis Of Antimalarial Drug Action
gb|AJW00309.1| **Mass:** 40510 **Score:** 287 **Matches:** 7(2) **Sequences:** 7(2)
Qcr2p [Saccharomyces cerevisiae YJM450]
gb|AJW01186.1| **Mass:** 40510 **Score:** 287 **Matches:** 7(2) **Sequences:** 7(2)
Qcr2p [Saccharomyces cerevisiae YJM1381]
gb|AJW01625.1| **Mass:** 40510 **Score:** 287 **Matches:** 7(2) **Sequences:** 7(2)
Qcr2p [Saccharomyces cerevisiae YJM1383]
gb|AJW02054.1| **Mass:** 40510 **Score:** 287 **Matches:** 7(2) **Sequences:** 7(2)
Qcr2p [Saccharomyces cerevisiae YJM1385]
gb|AJW03343.1| **Mass:** 40510 **Score:** 287 **Matches:** 7(2) **Sequences:** 7(2)
Qcr2p [Saccharomyces cerevisiae YJM1388]
gb|AJW03780.1| **Mass:** 40510 **Score:** 287 **Matches:** 7(2) **Sequences:** 7(2)
Qcr2p [Saccharomyces cerevisiae YJM1389]
gb|AJW04648.1| **Mass:** 40510 **Score:** 287 **Matches:** 7(2) **Sequences:** 7(2)
Qcr2p [Saccharomyces cerevisiae YJM1400]
gb|AJW10314.1| **Mass:** 40510 **Score:** 287 **Matches:** 7(2) **Sequences:** 7(2)
Qcr2p [Saccharomyces cerevisiae YJM1450]
gb|AJW10750.1| **Mass:** 40510 **Score:** 287 **Matches:** 7(2) **Sequences:** 7(2)
Qcr2p [Saccharomyces cerevisiae YJM1083]
gb|AJW11459.1| **Mass:** 40510 **Score:** 287 **Matches:** 7(2) **Sequences:** 7(2)
Qcr2p [Saccharomyces cerevisiae YJM1133]
gb|AJW11903.1| **Mass:** 40510 **Score:** 287 **Matches:** 7(2) **Sequences:** 7(2)
Qcr2p [Saccharomyces cerevisiae YJM1190]
gb|AJW16250.1| **Mass:** 40510 **Score:** 287 **Matches:** 7(2) **Sequences:** 7(2)
Qcr2p [Saccharomyces cerevisiae YJM1304]
gb|AJW16683.1| **Mass:** 40510 **Score:** 287 **Matches:** 7(2) **Sequences:** 7(2)
Qcr2p [Saccharomyces cerevisiae YJM1307]
gb|AJW20148.1| **Mass:** 40510 **Score:** 287 **Matches:** 7(2) **Sequences:** 7(2)
Qcr2p [Saccharomyces cerevisiae YJM1355]
gb|AJW20586.1| **Mass:** 40510 **Score:** 287 **Matches:** 7(2) **Sequences:** 7(2)
Qcr2p [Saccharomyces cerevisiae YJM451]
gb|AJW21894.1| **Mass:** 40510 **Score:** 287 **Matches:** 7(2) **Sequences:** 7(2)
Qcr2p [Saccharomyces cerevisiae YJM470]
gb|AJW22335.1| **Mass:** 40510 **Score:** 287 **Matches:** 7(2) **Sequences:** 7(2)
Qcr2p [Saccharomyces cerevisiae YJM541]
gb|AJW22773.1| **Mass:** 40510 **Score:** 287 **Matches:** 7(2) **Sequences:** 7(2)
Qcr2p [Saccharomyces cerevisiae YJM554]
gb|AJW24528.1| **Mass:** 40510 **Score:** 287 **Matches:** 7(2) **Sequences:** 7(2)
Qcr2p [Saccharomyces cerevisiae YJM682]
gb|AJW24966.1| **Mass:** 40510 **Score:** 287 **Matches:** 7(2) **Sequences:** 7(2)
Qcr2p [Saccharomyces cerevisiae YJM683]
gb|AJW25375.1| **Mass:** 40510 **Score:** 287 **Matches:** 7(2) **Sequences:** 7(2)
Qcr2p [Saccharomyces cerevisiae YJM689]
emb|CAA28768.1| **Mass:** 40510 **Score:** 287 **Matches:** 7(2) **Sequences:** 7(2)
precursor QH2:cytochrome-c oxidoreductase subunit II [Saccharomyces cerevisiae]
gb|AAS56394.1| **Mass:** 40510 **Score:** 287 **Matches:** 7(2) **Sequences:** 7(2)
YPR191W [Saccharomyces cerevisiae]
gb|AAB64620.1| **Mass:** 40510 **Score:** 287 **Matches:** 7(2) **Sequences:** 7(2)
Ubiquinol-cytochrome C reductase core protein 2 [Saccharomyces cerevisiae]
gb|AJV91398.1| **Mass:** 40510 **Score:** 287 **Matches:** 7(2) **Sequences:** 7(2)
Qcr2p [Saccharomyces cerevisiae YJM1460]
gb|AJV93151.1| **Mass:** 40510 **Score:** 287 **Matches:** 7(2) **Sequences:** 7(2)
Qcr2p [Saccharomyces cerevisiae YJM1479]
gb|AJV94123.1| **Mass:** 40510 **Score:** 287 **Matches:** 7(2) **Sequences:** 7(2)
Qcr2p [Saccharomyces cerevisiae YJM1549]
gb|AJV95263.1| **Mass:** 40510 **Score:** 287 **Matches:** 7(2) **Sequences:** 7(2)
Qcr2p [Saccharomyces cerevisiae YJM1592]
gb|AJV95700.1| **Mass:** 40510 **Score:** 287 **Matches:** 7(2) **Sequences:** 7(2)
Qcr2p [Saccharomyces cerevisiae YJM1615]
gb|AJV99016.1| **Mass:** 40510 **Score:** 287 **Matches:** 7(2) **Sequences:** 7(2)
Qcr2p [Saccharomyces cerevisiae YJM320]
gb|AJV99456.1| **Mass:** 40510 **Score:** 287 **Matches:** 7(2) **Sequences:** 7(2)
Qcr2p [Saccharomyces cerevisiae YJM326]
gb|AJV99877.1| **Mass:** 40510 **Score:** 287 **Matches:** 7(2) **Sequences:** 7(2)
Qcr2p [Saccharomyces cerevisiae YJM428]

---

3. pdb|1KYO|D **Mass:** 27982 **Score:** 164 **Matches:** 3(2) **Sequences:** 3(2)
Chain D, Yeast Cytochrome Bc1 Complex With Bound Substrate Cytochrome C
☐ Check to include this hit in error tolerant search or archive report

Query	Observed	Mr(expt)	Mr(calc)	ppm	Miss	Score	Expect	Rank	Unique	Peptide
1	1101.6886	1100.6813	1100.6131	62.0	0	31	1.3	1	U	K.FVFNPPKPR.K
52	1790.9829	1789.9756	1789.8635	62.7	0	66	0.00042	1	U	K.LSDYIPGPYPNEQAAR.A
70	2119.0358	2118.0285	2117.9113	55.4	0	67	0.0003	1	U	K.DVTTFLNWCAEPEHDER.K

Proteins matching the same set of peptides:
pdb|1KYO|O **Mass:** 27982 **Score:** 164 **Matches:** 3(2) **Sequences:** 3(2)
Chain O, Yeast Cytochrome Bc1 Complex With Bound Substrate Cytochrome C
pdb|2IBZ|D **Mass:** 27982 **Score:** 164 **Matches:** 3(2) **Sequences:** 3(2)
Chain D, Yeast Cytochrome Bc1 Complex With Stigmatellin
pdb|3CX5|D **Mass:** 27982 **Score:** 164 **Matches:** 3(2) **Sequences:** 3(2)
Chain D, Structure Of Complex Iii With Bound Cytochrome C In Reduced State And Definition Of A Minimal Core Interface For Electron Trans
pdb|3CX5|O **Mass:** 27982 **Score:** 164 **Matches:** 3(2) **Sequences:** 3(2)
Chain O, Structure Of Complex Iii With Bound Cytochrome C In Reduced State And Definition Of A Minimal Core Interface For Electron Trans
pdb|3CXH|D **Mass:** 27982 **Score:** 164 **Matches:** 3(2) **Sequences:** 3(2)

Chain D, Structure Of Yeast Complex Iii With Isoform-2 Cytochrome C Bound And Definition Of A Minimal Core Interface For Electron Transf
pdb|3CXH|O **Mass:** 27982 **Score:** 164 **Matches:** 3(2) **Sequences:** 3(2)
Chain O, Structure Of Yeast Complex Iii With Isoform-2 Cytochrome C Bound And Definition Of A Minimal Core Interface For Electron Transf
pdb|4PD4|D **Mass:** 27982 **Score:** 164 **Matches:** 3(2) **Sequences:** 3(2)
Chain D, Structural Analysis Of Atovaquone-inhibited Cytochrome Bc1 Complex Reveals The Molecular Basis Of Antimalarial Drug Action
gb|AJU00338.1| **Mass:** 34241 **Score:** 164 **Matches:** 3(2) **Sequences:** 3(2)
Cyt1p [Saccharomyces cerevisiae YJM1386]
gb|AJU00821.1| **Mass:** 34261 **Score:** 164 **Matches:** 3(2) **Sequences:** 3(2)
Cyt1p [Saccharomyces cerevisiae YJM1387]
gb|AJU01309.1| **Mass:** 34261 **Score:** 164 **Matches:** 3(2) **Sequences:** 3(2)
Cyt1p [Saccharomyces cerevisiae YJM1388]
gb|AJU01793.1| **Mass:** 34261 **Score:** 164 **Matches:** 3(2) **Sequences:** 3(2)
Cyt1p [Saccharomyces cerevisiae YJM1389]
gb|AJU02279.1| **Mass:** 34227 **Score:** 164 **Matches:** 3(2) **Sequences:** 3(2)
Cyt1p [Saccharomyces cerevisiae YJM1399]
gb|AJU02770.1| **Mass:** 34261 **Score:** 164 **Matches:** 3(2) **Sequences:** 3(2)
Cyt1p [Saccharomyces cerevisiae YJM1400]
gb|AJU03262.1| **Mass:** 34261 **Score:** 164 **Matches:** 3(2) **Sequences:** 3(2)
Cyt1p [Saccharomyces cerevisiae YJM1401]
gb|AJU03762.1| **Mass:** 34261 **Score:** 164 **Matches:** 3(2) **Sequences:** 3(2)
Cyt1p [Saccharomyces cerevisiae YJM1402]
gb|AJU04248.1| **Mass:** 34261 **Score:** 164 **Matches:** 3(2) **Sequences:** 3(2)
Cyt1p [Saccharomyces cerevisiae YJM1415]
gb|AJU04738.1| **Mass:** 34261 **Score:** 164 **Matches:** 3(2) **Sequences:** 3(2)
Cyt1p [Saccharomyces cerevisiae YJM1417]
gb|AJU05240.1| **Mass:** 34261 **Score:** 164 **Matches:** 3(2) **Sequences:** 3(2)
Cyt1p [Saccharomyces cerevisiae YJM1418]
gb|AJU05700.1| **Mass:** 34261 **Score:** 164 **Matches:** 3(2) **Sequences:** 3(2)
Cyt1p [Saccharomyces cerevisiae YJM1419]
gb|AJU06191.1| **Mass:** 34261 **Score:** 164 **Matches:** 3(2) **Sequences:** 3(2)
Cyt1p [Saccharomyces cerevisiae YJM1433]
gb|AJU06697.1| **Mass:** 34261 **Score:** 164 **Matches:** 3(2) **Sequences:** 3(2)
Cyt1p [Saccharomyces cerevisiae YJM1434]
gb|AJU07185.1| **Mass:** 34319 **Score:** 164 **Matches:** 3(2) **Sequences:** 3(2)
Cyt1p [Saccharomyces cerevisiae YJM1439]
gb|AJU07676.1| **Mass:** 34261 **Score:** 164 **Matches:** 3(2) **Sequences:** 3(2)
Cyt1p [Saccharomyces cerevisiae YJM1443]
gb|AJU08168.1| **Mass:** 34261 **Score:** 164 **Matches:** 3(2) **Sequences:** 3(2)
Cyt1p [Saccharomyces cerevisiae YJM1444]
gb|AJU08650.1| **Mass:** 34261 **Score:** 164 **Matches:** 3(2) **Sequences:** 3(2)
Cyt1p [Saccharomyces cerevisiae YJM1447]
gb|AJU09118.1| **Mass:** 34261 **Score:** 164 **Matches:** 3(2) **Sequences:** 3(2)
Cyt1p [Saccharomyces cerevisiae YJM1450]
gb|AJU09607.1| **Mass:** 34261 **Score:** 164 **Matches:** 3(2) **Sequences:** 3(2)
Cyt1p [Saccharomyces cerevisiae YJM1460]
gb|AJU10092.1| **Mass:** 34261 **Score:** 164 **Matches:** 3(2) **Sequences:** 3(2)
Cyt1p [Saccharomyces cerevisiae YJM1463]
gb|AJU10580.1| **Mass:** 34261 **Score:** 164 **Matches:** 3(2) **Sequences:** 3(2)
Cyt1p [Saccharomyces cerevisiae YJM1477]
gb|AJU11074.1| **Mass:** 34274 **Score:** 164 **Matches:** 3(2) **Sequences:** 3(2)
Cyt1p [Saccharomyces cerevisiae YJM1478]
gb|AJU11564.1| **Mass:** 34261 **Score:** 164 **Matches:** 3(2) **Sequences:** 3(2)
Cyt1p [Saccharomyces cerevisiae YJM1479]
gb|AJU12548.1| **Mass:** 34227 **Score:** 164 **Matches:** 3(2) **Sequences:** 3(2)
Cyt1p [Saccharomyces cerevisiae YJM1527]
gb|AJU13028.1| **Mass:** 34261 **Score:** 164 **Matches:** 3(2) **Sequences:** 3(2)
Cyt1p [Saccharomyces cerevisiae YJM1549]
gb|AJU13527.1| **Mass:** 34261 **Score:** 164 **Matches:** 3(2) **Sequences:** 3(2)
Cyt1p [Saccharomyces cerevisiae YJM1573]
gb|AJU14018.1| **Mass:** 34227 **Score:** 164 **Matches:** 3(2) **Sequences:** 3(2)
Cyt1p [Saccharomyces cerevisiae YJM1574]
gb|AJU14510.1| **Mass:** 34261 **Score:** 164 **Matches:** 3(2) **Sequences:** 3(2)
Cyt1p [Saccharomyces cerevisiae YJM1592]
gb|AJU15009.1| **Mass:** 34261 **Score:** 164 **Matches:** 3(2) **Sequences:** 3(2)
Cyt1p [Saccharomyces cerevisiae YJM1615]
emb|CAA25375.1| **Mass:** 34261 **Score:** 164 **Matches:** 3(2) **Sequences:** 3(2)
cytochrome c1 precursor [Saccharomyces cerevisiae]
gb|AJP41596.1| **Mass:** 34261 **Score:** 164 **Matches:** 3(2) **Sequences:** 3(2)
Cyt1p [Saccharomyces cerevisiae YJM1078]
emb|CAG62193.1| **Mass:** 33290 **Score:** 164 **Matches:** 3(2) **Sequences:** 3(2)
unnamed protein product [Candida glabrata]
gb|AJT71018.1| **Mass:** 34261 **Score:** 164 **Matches:** 3(2) **Sequences:** 3(2)
Cyt1p [Saccharomyces cerevisiae YJM189]
gb|AJT71508.1| **Mass:** 34287 **Score:** 164 **Matches:** 3(2) **Sequences:** 3(2)
Cyt1p [Saccharomyces cerevisiae YJM193]
gb|AJT71996.1| **Mass:** 34261 **Score:** 164 **Matches:** 3(2) **Sequences:** 3(2)
Cyt1p [Saccharomyces cerevisiae YJM195]
gb|AJT72484.1| **Mass:** 34261 **Score:** 164 **Matches:** 3(2) **Sequences:** 3(2)
Cyt1p [Saccharomyces cerevisiae YJM244]
gb|AJT72965.1| **Mass:** 34261 **Score:** 164 **Matches:** 3(2) **Sequences:** 3(2)
Cyt1p [Saccharomyces cerevisiae YJM248]
gb|AJT73443.1| **Mass:** 34261 **Score:** 164 **Matches:** 3(2) **Sequences:** 3(2)
Cyt1p [Saccharomyces cerevisiae YJM270]
gb|AJT73937.1| **Mass:** 34287 **Score:** 164 **Matches:** 3(2) **Sequences:** 3(2)
Cyt1p [Saccharomyces cerevisiae YJM271]
gb|AJT74427.1| **Mass:** 34261 **Score:** 164 **Matches:** 3(2) **Sequences:** 3(2)
Cyt1p [Saccharomyces cerevisiae YJM320]
gb|AJT74919.1| **Mass:** 34261 **Score:** 164 **Matches:** 3(2) **Sequences:** 3(2)

Cyt1p [Saccharomyces cerevisiae YJM326]
gb|AJT75413.1| **Mass:** 34261 **Score:** 164 **Matches:** 3(2) **Sequences:** 3(2)
Cyt1p [Saccharomyces cerevisiae YJM428]
gb|AJT75899.1| **Mass:** 34261 **Score:** 164 **Matches:** 3(2) **Sequences:** 3(2)
Cyt1p [Saccharomyces cerevisiae YJM450]
gb|AJT76393.1| **Mass:** 34261 **Score:** 164 **Matches:** 3(2) **Sequences:** 3(2)
Cyt1p [Saccharomyces cerevisiae YJM451]
gb|AJT76888.1| **Mass:** 34261 **Score:** 164 **Matches:** 3(2) **Sequences:** 3(2)
Cyt1p [Saccharomyces cerevisiae YJM453]
gb|AHY77366.1| **Mass:** 34261 **Score:** 164 **Matches:** 3(2) **Sequences:** 3(2)
Cyt1p [Saccharomyces cerevisiae YJM993]
gb|AJT77380.1| **Mass:** 34261 **Score:** 164 **Matches:** 3(2) **Sequences:** 3(2)
Cyt1p [Saccharomyces cerevisiae YJM456]
gb|AJT78350.1| **Mass:** 34261 **Score:** 164 **Matches:** 3(2) **Sequences:** 3(2)
Cyt1p [Saccharomyces cerevisiae YJM541]
gb|AJT78847.1| **Mass:** 34261 **Score:** 164 **Matches:** 3(2) **Sequences:** 3(2)
Cyt1p [Saccharomyces cerevisiae YJM554]
gb|AJT79340.1| **Mass:** 34261 **Score:** 164 **Matches:** 3(2) **Sequences:** 3(2)
Cyt1p [Saccharomyces cerevisiae YJM555]
gb|AJT79831.1| **Mass:** 34261 **Score:** 164 **Matches:** 3(2) **Sequences:** 3(2)
Cyt1p [Saccharomyces cerevisiae YJM627]
gb|AJT80320.1| **Mass:** 34261 **Score:** 164 **Matches:** 3(2) **Sequences:** 3(2)
Cyt1p [Saccharomyces cerevisiae YJM681]
gb|AJT80809.1| **Mass:** 34261 **Score:** 164 **Matches:** 3(2) **Sequences:** 3(2)
Cyt1p [Saccharomyces cerevisiae YJM682]
gb|AJT81300.1| **Mass:** 34261 **Score:** 164 **Matches:** 3(2) **Sequences:** 3(2)
Cyt1p [Saccharomyces cerevisiae YJM683]
gb|AJT81801.1| **Mass:** 34261 **Score:** 164 **Matches:** 3(2) **Sequences:** 3(2)
Cyt1p [Saccharomyces cerevisiae YJM689]
gb|AJT82284.1| **Mass:** 34261 **Score:** 164 **Matches:** 3(2) **Sequences:** 3(2)
Cyt1p [Saccharomyces cerevisiae YJM693]
gb|AJT82776.1| **Mass:** 34261 **Score:** 164 **Matches:** 3(2) **Sequences:** 3(2)
Cyt1p [Saccharomyces cerevisiae YJM969]
gb|AJT83271.1| **Mass:** 34261 **Score:** 164 **Matches:** 3(2) **Sequences:** 3(2)
Cyt1p [Saccharomyces cerevisiae YJM972]
gb|AJT83758.1| **Mass:** 34261 **Score:** 164 **Matches:** 3(2) **Sequences:** 3(2)
Cyt1p [Saccharomyces cerevisiae YJM975]
gb|AJT84250.1| **Mass:** 34261 **Score:** 164 **Matches:** 3(2) **Sequences:** 3(2)
Cyt1p [Saccharomyces cerevisiae YJM978]
gb|AJT84742.1| **Mass:** 34261 **Score:** 164 **Matches:** 3(2) **Sequences:** 3(2)
Cyt1p [Saccharomyces cerevisiae YJM981]
gb|AJT85237.1| **Mass:** 34261 **Score:** 164 **Matches:** 3(2) **Sequences:** 3(2)
Cyt1p [Saccharomyces cerevisiae YJM984]
gb|AJT85725.1| **Mass:** 34261 **Score:** 164 **Matches:** 3(2) **Sequences:** 3(2)
Cyt1p [Saccharomyces cerevisiae YJM987]
gb|AJT86219.1| **Mass:** 34261 **Score:** 164 **Matches:** 3(2) **Sequences:** 3(2)
Cyt1p [Saccharomyces cerevisiae YJM990]
emb|CAY86351.1| **Mass:** 34819 **Score:** 164 **Matches:** 3(2) **Sequences:** 3(2)
Cyt1p [Saccharomyces cerevisiae EC1118]
gb|AJT86715.1| **Mass:** 34261 **Score:** 164 **Matches:** 3(2) **Sequences:** 3(2)
Cyt1p [Saccharomyces cerevisiae YJM996]
gb|AJT87188.1| **Mass:** 34261 **Score:** 164 **Matches:** 3(2) **Sequences:** 3(2)
Cyt1p [Saccharomyces cerevisiae YJM1083]
gb|AJT88174.1| **Mass:** 34261 **Score:** 164 **Matches:** 3(2) **Sequences:** 3(2)
Cyt1p [Saccharomyces cerevisiae YJM1133]
gb|AJT88663.1| **Mass:** 34261 **Score:** 164 **Matches:** 3(2) **Sequences:** 3(2)
Cyt1p [Saccharomyces cerevisiae YJM1190]
gb|AJT89157.1| **Mass:** 34261 **Score:** 164 **Matches:** 3(2) **Sequences:** 3(2)
Cyt1p [Saccharomyces cerevisiae YJM1199]
gb|AJT89645.1| **Mass:** 34261 **Score:** 164 **Matches:** 3(2) **Sequences:** 3(2)
Cyt1p [Saccharomyces cerevisiae YJM1202]
gb|AJT90137.1| **Mass:** 34261 **Score:** 164 **Matches:** 3(2) **Sequences:** 3(2)
Cyt1p [Saccharomyces cerevisiae YJM1208]
gb|AJT90624.1| **Mass:** 34261 **Score:** 164 **Matches:** 3(2) **Sequences:** 3(2)
Cyt1p [Saccharomyces cerevisiae YJM1242]
gb|AJT91119.1| **Mass:** 34261 **Score:** 164 **Matches:** 3(2) **Sequences:** 3(2)
Cyt1p [Saccharomyces cerevisiae YJM1244]
gb|AJT91599.1| **Mass:** 34319 **Score:** 164 **Matches:** 3(2) **Sequences:** 3(2)
Cyt1p [Saccharomyces cerevisiae YJM1248]
gb|AJT92493.1| **Mass:** 34261 **Score:** 164 **Matches:** 3(2) **Sequences:** 3(2)
Cyt1p [Saccharomyces cerevisiae YJM1252]
gb|AJT92989.1| **Mass:** 34261 **Score:** 164 **Matches:** 3(2) **Sequences:** 3(2)
Cyt1p [Saccharomyces cerevisiae YJM1273]
gb|AJT93473.1| **Mass:** 34261 **Score:** 164 **Matches:** 3(2) **Sequences:** 3(2)
Cyt1p [Saccharomyces cerevisiae YJM1304]
gb|AJT93959.1| **Mass:** 34261 **Score:** 164 **Matches:** 3(2) **Sequences:** 3(2)
Cyt1p [Saccharomyces cerevisiae YJM1307]
gb|AJT94443.1| **Mass:** 34261 **Score:** 164 **Matches:** 3(2) **Sequences:** 3(2)
Cyt1p [Saccharomyces cerevisiae YJM1311]
emb|CAA94550.1| **Mass:** 34261 **Score:** 164 **Matches:** 3(2) **Sequences:** 3(2)
YOR29-16 [Saccharomyces cerevisiae]
gb|AJT94933.1| **Mass:** 34261 **Score:** 164 **Matches:** 3(2) **Sequences:** 3(2)
Cyt1p [Saccharomyces cerevisiae YJM1326]
gb|AJT95412.1| **Mass:** 34261 **Score:** 164 **Matches:** 3(2) **Sequences:** 3(2)
Cyt1p [Saccharomyces cerevisiae YJM1332]
gb|AJT95907.1| **Mass:** 34261 **Score:** 164 **Matches:** 3(2) **Sequences:** 3(2)
Cyt1p [Saccharomyces cerevisiae YJM1336]
gb|AJT96394.1| **Mass:** 34227 **Score:** 164 **Matches:** 3(2) **Sequences:** 3(2)

Cyt1p [Saccharomyces cerevisiae YJM1338]
gb|AJT96890.1| **Mass:** 34261 **Score:** 164 **Matches:** 3(2) **Sequences:** 3(2)
Cyt1p [Saccharomyces cerevisiae YJM1341]
gb|AJT97385.1| **Mass:** 34261 **Score:** 164 **Matches:** 3(2) **Sequences:** 3(2)
Cyt1p [Saccharomyces cerevisiae YJM1342]
gb|AJT97884.1| **Mass:** 34227 **Score:** 164 **Matches:** 3(2) **Sequences:** 3(2)
Cyt1p [Saccharomyces cerevisiae YJM1355]
gb|AJT98373.1| **Mass:** 34261 **Score:** 164 **Matches:** 3(2) **Sequences:** 3(2)
Cyt1p [Saccharomyces cerevisiae YJM1356]
gb|AJT98863.1| **Mass:** 34261 **Score:** 164 **Matches:** 3(2) **Sequences:** 3(2)
Cyt1p [Saccharomyces cerevisiae YJM1381]
emb|CAA99258.1| **Mass:** 34261 **Score:** 164 **Matches:** 3(2) **Sequences:** 3(2)
CYT1 [Saccharomyces cerevisiae]
gb|AJT99355.1| **Mass:** 34241 **Score:** 164 **Matches:** 3(2) **Sequences:** 3(2)
Cyt1p [Saccharomyces cerevisiae YJM1383]
gb|AJT99846.1| **Mass:** 34261 **Score:** 164 **Matches:** 3(2) **Sequences:** 3(2)
Cyt1p [Saccharomyces cerevisiae YJM1385]

---

4. pdb|1EZV|F **Mass:** 14385 **Score:** 83 **Matches:** 1(1) **Sequences:** 1(1)
Chain F, Structure Of The Yeast Cytochrome Bc1 Complex Co- Crystallized With An Antibody Fv-Fragment
☐ Check to include this hit in error tolerant search or archive report

Query	Observed	Mr(expt)	Mr(calc)	ppm	Miss	Score	Expect	Rank	Unique	Peptide
66	1976.0935	1975.0862	1974.9720	57.8	0	83	7.9e-006	1	U	K.FDDLIAEENPIMQTALR.R

Proteins matching the same set of peptides:
pdb|1KB9|G **Mass:** 14385 **Score:** 83 **Matches:** 1(1) **Sequences:** 1(1)
Chain G, Yeast Cytochrome Bc1 Complex
pdb|1KYO|G **Mass:** 14482 **Score:** 83 **Matches:** 1(1) **Sequences:** 1(1)
Chain G, Yeast Cytochrome Bc1 Complex With Bound Substrate Cytochrome C
pdb|1KYO|R **Mass:** 14482 **Score:** 83 **Matches:** 1(1) **Sequences:** 1(1)
Chain R, Yeast Cytochrome Bc1 Complex With Bound Substrate Cytochrome C
pdb|1P84|G **Mass:** 14385 **Score:** 83 **Matches:** 1(1) **Sequences:** 1(1)
Chain G, Hdbt Inhibited Yeast Cytochrome Bc1 Complex
pdb|2IBZ|F **Mass:** 14613 **Score:** 83 **Matches:** 1(1) **Sequences:** 1(1)
Chain F, Yeast Cytochrome Bc1 Complex With Stigmatellin
pdb|3CX5|G **Mass:** 14482 **Score:** 83 **Matches:** 1(1) **Sequences:** 1(1)
Chain G, Structure Of Complex Iii With Bound Cytochrome C In Reduced State And Definition Of A Minimal Core Interface For Electron Trans
pdb|3CX5|R **Mass:** 14482 **Score:** 83 **Matches:** 1(1) **Sequences:** 1(1)
Chain R, Structure Of Complex Iii With Bound Cytochrome C In Reduced State And Definition Of A Minimal Core Interface For Electron Trans
pdb|3CXH|G **Mass:** 14482 **Score:** 83 **Matches:** 1(1) **Sequences:** 1(1)
Chain G, Structure Of Yeast Complex Iii With Isoform-2 Cytochrome C Bound And Definition Of A Minimal Core Interface For Electron Transf
pdb|3CXH|R **Mass:** 14482 **Score:** 83 **Matches:** 1(1) **Sequences:** 1(1)
Chain R, Structure Of Yeast Complex Iii With Isoform-2 Cytochrome C Bound And Definition Of A Minimal Core Interface For Electron Transf
pdb|4PD4|G **Mass:** 14482 **Score:** 83 **Matches:** 1(1) **Sequences:** 1(1)
Chain G, Structural Analysis Of Atovaquone-inhibited Cytochrome Bc1 Complex Reveals The Molecular Basis Of Antimalarial Drug Action
gb|AJV00294.1| **Mass:** 14613 **Score:** 83 **Matches:** 1(1) **Sequences:** 1(1)
Qcr7p [Saccharomyces cerevisiae YJM1386]
gb|AJV01010.1| **Mass:** 14613 **Score:** 83 **Matches:** 1(1) **Sequences:** 1(1)
Qcr7p [Saccharomyces cerevisiae YJM1387]
gb|AJV01721.1| **Mass:** 14613 **Score:** 83 **Matches:** 1(1) **Sequences:** 1(1)
Qcr7p [Saccharomyces cerevisiae YJM1388]
gb|AJV02417.1| **Mass:** 14613 **Score:** 83 **Matches:** 1(1) **Sequences:** 1(1)
Qcr7p [Saccharomyces cerevisiae YJM1389]
gb|AJV03100.1| **Mass:** 14673 **Score:** 83 **Matches:** 1(1) **Sequences:** 1(1)
Qcr7p [Saccharomyces cerevisiae YJM1399]
gb|AJV03804.1| **Mass:** 14613 **Score:** 83 **Matches:** 1(1) **Sequences:** 1(1)
Qcr7p [Saccharomyces cerevisiae YJM1400]
gb|AJV04512.1| **Mass:** 14613 **Score:** 83 **Matches:** 1(1) **Sequences:** 1(1)
Qcr7p [Saccharomyces cerevisiae YJM1401]
gb|AJV05227.1| **Mass:** 14613 **Score:** 83 **Matches:** 1(1) **Sequences:** 1(1)
Qcr7p [Saccharomyces cerevisiae YJM1402]
gb|AJV05930.1| **Mass:** 14613 **Score:** 83 **Matches:** 1(1) **Sequences:** 1(1)
Qcr7p [Saccharomyces cerevisiae YJM1415]
gb|AJV06635.1| **Mass:** 14613 **Score:** 83 **Matches:** 1(1) **Sequences:** 1(1)
Qcr7p [Saccharomyces cerevisiae YJM1417]
gb|AJV07349.1| **Mass:** 14613 **Score:** 83 **Matches:** 1(1) **Sequences:** 1(1)
Qcr7p [Saccharomyces cerevisiae YJM1418]
gb|AJV08035.1| **Mass:** 14613 **Score:** 83 **Matches:** 1(1) **Sequences:** 1(1)
Qcr7p [Saccharomyces cerevisiae YJM1419]
gb|AJV08721.1| **Mass:** 14613 **Score:** 83 **Matches:** 1(1) **Sequences:** 1(1)
Qcr7p [Saccharomyces cerevisiae YJM1433]
gb|AJV09433.1| **Mass:** 14613 **Score:** 83 **Matches:** 1(1) **Sequences:** 1(1)
Qcr7p [Saccharomyces cerevisiae YJM1434]
gb|AJV10136.1| **Mass:** 14613 **Score:** 83 **Matches:** 1(1) **Sequences:** 1(1)
Qcr7p [Saccharomyces cerevisiae YJM1439]
gb|AJV10838.1| **Mass:** 14613 **Score:** 83 **Matches:** 1(1) **Sequences:** 1(1)
Qcr7p [Saccharomyces cerevisiae YJM1443]
gb|AJV11491.1| **Mass:** 14613 **Score:** 83 **Matches:** 1(1) **Sequences:** 1(1)
Qcr7p [Saccharomyces cerevisiae YJM1444]
gb|AJV12167.1| **Mass:** 14613 **Score:** 83 **Matches:** 1(1) **Sequences:** 1(1)
Qcr7p [Saccharomyces cerevisiae YJM1447]
gb|AJV12817.1| **Mass:** 14613 **Score:** 83 **Matches:** 1(1) **Sequences:** 1(1)
Qcr7p [Saccharomyces cerevisiae YJM1450]
gb|AJV13528.1| **Mass:** 14613 **Score:** 83 **Matches:** 1(1) **Sequences:** 1(1)
Qcr7p [Saccharomyces cerevisiae YJM1460]
gb|AJV14192.1| **Mass:** 14613 **Score:** 83 **Matches:** 1(1) **Sequences:** 1(1)

Qcr7p [Saccharomyces cerevisiae YJM1463]
gb|AJV14893.1| **Mass:** 14613 **Score:** 83 **Matches:** 1(1) **Sequences:** 1(1)
Qcr7p [Saccharomyces cerevisiae YJM1477]
gb|AJV15613.1| **Mass:** 14613 **Score:** 83 **Matches:** 1(1) **Sequences:** 1(1)
Qcr7p [Saccharomyces cerevisiae YJM1478]
gb|AJV16317.1| **Mass:** 14613 **Score:** 83 **Matches:** 1(1) **Sequences:** 1(1)
Qcr7p [Saccharomyces cerevisiae YJM1479]
gb|AJV17021.1| **Mass:** 14613 **Score:** 83 **Matches:** 1(1) **Sequences:** 1(1)
Qcr7p [Saccharomyces cerevisiae YJM1526]
gb|AJV17735.1| **Mass:** 14613 **Score:** 83 **Matches:** 1(1) **Sequences:** 1(1)
Qcr7p [Saccharomyces cerevisiae YJM1527]
gb|AJV18449.1| **Mass:** 14613 **Score:** 83 **Matches:** 1(1) **Sequences:** 1(1)
Qcr7p [Saccharomyces cerevisiae YJM1549]
gb|AJV19161.1| **Mass:** 14613 **Score:** 83 **Matches:** 1(1) **Sequences:** 1(1)
Qcr7p [Saccharomyces cerevisiae YJM1573]
gb|AJV19866.1| **Mass:** 14613 **Score:** 83 **Matches:** 1(1) **Sequences:** 1(1)
Qcr7p [Saccharomyces cerevisiae YJM1574]
gb|AJV20577.1| **Mass:** 14613 **Score:** 83 **Matches:** 1(1) **Sequences:** 1(1)
Qcr7p [Saccharomyces cerevisiae YJM1592]
gb|AJV21294.1| **Mass:** 14613 **Score:** 83 **Matches:** 1(1) **Sequences:** 1(1)
Qcr7p [Saccharomyces cerevisiae YJM1615]
emb|CAA25064.1| **Mass:** 14612 **Score:** 83 **Matches:** 1(1) **Sequences:** 1(1)
unnamed protein product [Saccharomyces cerevisiae]
gb|AJU58321.1| **Mass:** 14613 **Score:** 83 **Matches:** 1(1) **Sequences:** 1(1)
Qcr7p [Saccharomyces cerevisiae YJM189]
gb|AJU59011.1| **Mass:** 14613 **Score:** 83 **Matches:** 1(1) **Sequences:** 1(1)
Qcr7p [Saccharomyces cerevisiae YJM193]
gb|AJU59719.1| **Mass:** 14613 **Score:** 83 **Matches:** 1(1) **Sequences:** 1(1)
Qcr7p [Saccharomyces cerevisiae YJM195]
gb|AJU61784.1| **Mass:** 14613 **Score:** 83 **Matches:** 1(1) **Sequences:** 1(1)
Qcr7p [Saccharomyces cerevisiae YJM270]
gb|AJU62478.1| **Mass:** 14613 **Score:** 83 **Matches:** 1(1) **Sequences:** 1(1)
Qcr7p [Saccharomyces cerevisiae YJM271]
gb|AJU63182.1| **Mass:** 14613 **Score:** 83 **Matches:** 1(1) **Sequences:** 1(1)
Qcr7p [Saccharomyces cerevisiae YJM320]
gb|AJU63898.1| **Mass:** 14613 **Score:** 83 **Matches:** 1(1) **Sequences:** 1(1)
Qcr7p [Saccharomyces cerevisiae YJM326]
gb|AJU64582.1| **Mass:** 14613 **Score:** 83 **Matches:** 1(1) **Sequences:** 1(1)
Qcr7p [Saccharomyces cerevisiae YJM428]
gb|AAB64968.1| **Mass:** 14613 **Score:** 83 **Matches:** 1(1) **Sequences:** 1(1)
Qcr7p: ubiquinol-cytochrome c reductase [Saccharomyces cerevisiae]
gb|AJU65297.1| **Mass:** 14613 **Score:** 83 **Matches:** 1(1) **Sequences:** 1(1)
Qcr7p [Saccharomyces cerevisiae YJM450]
gb|AJU66015.1| **Mass:** 14613 **Score:** 83 **Matches:** 1(1) **Sequences:** 1(1)
Qcr7p [Saccharomyces cerevisiae YJM451]
gb|AJU66697.1| **Mass:** 14613 **Score:** 83 **Matches:** 1(1) **Sequences:** 1(1)
Qcr7p [Saccharomyces cerevisiae YJM453]
gb|AJU67410.1| **Mass:** 14613 **Score:** 83 **Matches:** 1(1) **Sequences:** 1(1)
Qcr7p [Saccharomyces cerevisiae YJM456]
gb|AJU68126.1| **Mass:** 14613 **Score:** 83 **Matches:** 1(1) **Sequences:** 1(1)
Qcr7p [Saccharomyces cerevisiae YJM470]
gb|AJU68825.1| **Mass:** 14613 **Score:** 83 **Matches:** 1(1) **Sequences:** 1(1)
Qcr7p [Saccharomyces cerevisiae YJM541]
gb|AJU69517.1| **Mass:** 14613 **Score:** 83 **Matches:** 1(1) **Sequences:** 1(1)
Qcr7p [Saccharomyces cerevisiae YJM554]
gb|AJU70232.1| **Mass:** 14613 **Score:** 83 **Matches:** 1(1) **Sequences:** 1(1)
Qcr7p [Saccharomyces cerevisiae YJM555]
gb|AJU70918.1| **Mass:** 14613 **Score:** 83 **Matches:** 1(1) **Sequences:** 1(1)
Qcr7p [Saccharomyces cerevisiae YJM627]
gb|AJU71626.1| **Mass:** 14613 **Score:** 83 **Matches:** 1(1) **Sequences:** 1(1)
Qcr7p [Saccharomyces cerevisiae YJM681]
gb|AJU72339.1| **Mass:** 14613 **Score:** 83 **Matches:** 1(1) **Sequences:** 1(1)
Qcr7p [Saccharomyces cerevisiae YJM682]
gb|AJU73048.1| **Mass:** 14613 **Score:** 83 **Matches:** 1(1) **Sequences:** 1(1)
Qcr7p [Saccharomyces cerevisiae YJM683]
gb|AJU73762.1| **Mass:** 14613 **Score:** 83 **Matches:** 1(1) **Sequences:** 1(1)
Qcr7p [Saccharomyces cerevisiae YJM689]
gb|AJU74474.1| **Mass:** 14613 **Score:** 83 **Matches:** 1(1) **Sequences:** 1(1)
Qcr7p [Saccharomyces cerevisiae YJM693]
gb|AJU75177.1| **Mass:** 14613 **Score:** 83 **Matches:** 1(1) **Sequences:** 1(1)
Qcr7p [Saccharomyces cerevisiae YJM969]
gb|AHY75473.1| **Mass:** 14613 **Score:** 83 **Matches:** 1(1) **Sequences:** 1(1)
Qcr7p [Saccharomyces cerevisiae YJM993]
gb|AJU75885.1| **Mass:** 14613 **Score:** 83 **Matches:** 1(1) **Sequences:** 1(1)
Qcr7p [Saccharomyces cerevisiae YJM972]
gb|AJU76592.1| **Mass:** 14613 **Score:** 83 **Matches:** 1(1) **Sequences:** 1(1)
Qcr7p [Saccharomyces cerevisiae YJM975]
gb|AJU77304.1| **Mass:** 14613 **Score:** 83 **Matches:** 1(1) **Sequences:** 1(1)
Qcr7p [Saccharomyces cerevisiae YJM978]
gb|AJU78014.1| **Mass:** 14613 **Score:** 83 **Matches:** 1(1) **Sequences:** 1(1)
Qcr7p [Saccharomyces cerevisiae YJM981]
gb|AJU78726.1| **Mass:** 14613 **Score:** 83 **Matches:** 1(1) **Sequences:** 1(1)
Qcr7p [Saccharomyces cerevisiae YJM984]
emb|CAY79028.1| **Mass:** 14613 **Score:** 83 **Matches:** 1(1) **Sequences:** 1(1)
Qcr7p [Saccharomyces cerevisiae EC1118]
gb|AJU79438.1| **Mass:** 14613 **Score:** 83 **Matches:** 1(1) **Sequences:** 1(1)
Qcr7p [Saccharomyces cerevisiae YJM987]
gb|AJU80040.1| **Mass:** 14613 **Score:** 83 **Matches:** 1(1) **Sequences:** 1(1)

Qcr7p [Saccharomyces cerevisiae YJM990]
gb|AJU80748.1| **Mass:** 14613 **Score:** 83 **Matches:** 1(1) **Sequences:** 1(1)
Qcr7p [Saccharomyces cerevisiae YJM996]
gb|AJU81432.1| **Mass:** 14613 **Score:** 83 **Matches:** 1(1) **Sequences:** 1(1)
Qcr7p [Saccharomyces cerevisiae YJM1083]
gb|AJU82137.1| **Mass:** 14613 **Score:** 83 **Matches:** 1(1) **Sequences:** 1(1)
Qcr7p [Saccharomyces cerevisiae YJM1129]
gb|AJU82718.1| **Mass:** 14613 **Score:** 83 **Matches:** 1(1) **Sequences:** 1(1)
Qcr7p [Saccharomyces cerevisiae YJM1133]
gb|AJU83429.1| **Mass:** 14613 **Score:** 83 **Matches:** 1(1) **Sequences:** 1(1)
Qcr7p [Saccharomyces cerevisiae YJM1190]
gb|AJU84141.1| **Mass:** 14613 **Score:** 83 **Matches:** 1(1) **Sequences:** 1(1)
Qcr7p [Saccharomyces cerevisiae YJM1199]
gb|AJU84856.1| **Mass:** 14613 **Score:** 83 **Matches:** 1(1) **Sequences:** 1(1)
Qcr7p [Saccharomyces cerevisiae YJM1202]
gb|AJU85553.1| **Mass:** 14613 **Score:** 83 **Matches:** 1(1) **Sequences:** 1(1)
Qcr7p [Saccharomyces cerevisiae YJM1208]
gb|AJU86253.1| **Mass:** 14613 **Score:** 83 **Matches:** 1(1) **Sequences:** 1(1)
Qcr7p [Saccharomyces cerevisiae YJM1242]
gb|AJU86907.1| **Mass:** 14613 **Score:** 83 **Matches:** 1(1) **Sequences:** 1(1)
Qcr7p [Saccharomyces cerevisiae YJM1244]
gb|AJU87595.1| **Mass:** 14613 **Score:** 83 **Matches:** 1(1) **Sequences:** 1(1)
Qcr7p [Saccharomyces cerevisiae YJM1248]
gb|AJU88287.1| **Mass:** 14613 **Score:** 83 **Matches:** 1(1) **Sequences:** 1(1)
Qcr7p [Saccharomyces cerevisiae YJM1250]
gb|AJU89680.1| **Mass:** 14613 **Score:** 83 **Matches:** 1(1) **Sequences:** 1(1)
Qcr7p [Saccharomyces cerevisiae YJM1273]
gb|AJU90394.1| **Mass:** 14613 **Score:** 83 **Matches:** 1(1) **Sequences:** 1(1)
Qcr7p [Saccharomyces cerevisiae YJM1304]
gb|AJU91110.1| **Mass:** 14613 **Score:** 83 **Matches:** 1(1) **Sequences:** 1(1)
Qcr7p [Saccharomyces cerevisiae YJM1307]
gb|AJU91829.1| **Mass:** 14613 **Score:** 83 **Matches:** 1(1) **Sequences:** 1(1)
Qcr7p [Saccharomyces cerevisiae YJM1311]
gb|AJU92539.1| **Mass:** 14613 **Score:** 83 **Matches:** 1(1) **Sequences:** 1(1)
Qcr7p [Saccharomyces cerevisiae YJM1326]
gb|AJU93262.1| **Mass:** 14613 **Score:** 83 **Matches:** 1(1) **Sequences:** 1(1)
Qcr7p [Saccharomyces cerevisiae YJM1332]
gb|AJU93966.1| **Mass:** 14613 **Score:** 83 **Matches:** 1(1) **Sequences:** 1(1)
Qcr7p [Saccharomyces cerevisiae YJM1336]
gb|AJU94665.1| **Mass:** 14613 **Score:** 83 **Matches:** 1(1) **Sequences:** 1(1)
Qcr7p [Saccharomyces cerevisiae YJM1338]
gb|AJU95368.1| **Mass:** 14613 **Score:** 83 **Matches:** 1(1) **Sequences:** 1(1)
Qcr7p [Saccharomyces cerevisiae YJM1341]
gb|AJU96077.1| **Mass:** 14613 **Score:** 83 **Matches:** 1(1) **Sequences:** 1(1)
Qcr7p [Saccharomyces cerevisiae YJM1342]
gb|AJU96770.1| **Mass:** 14613 **Score:** 83 **Matches:** 1(1) **Sequences:** 1(1)
Qcr7p [Saccharomyces cerevisiae YJM1355]
gb|AJU97475.1| **Mass:** 14613 **Score:** 83 **Matches:** 1(1) **Sequences:** 1(1)
Qcr7p [Saccharomyces cerevisiae YJM1356]
gb|AJU98186.1| **Mass:** 14613 **Score:** 83 **Matches:** 1(1) **Sequences:** 1(1)
Qcr7p [Saccharomyces cerevisiae YJM1381]
gb|AJU98885.1| **Mass:** 14613 **Score:** 83 **Matches:** 1(1) **Sequences:** 1(1)
Qcr7p [Saccharomyces cerevisiae YJM1383]
gb|AJU99599.1| **Mass:** 14613 **Score:** 83 **Matches:** 1(1) **Sequences:** 1(1)
Qcr7p [Saccharomyces cerevisiae YJM1385]

---

5. pdb|2HLD|D **Mass:** 51095 **Score:** 70 **Matches:** 3(0) **Sequences:** 3(0)
Chain D, Crystal Structure Of Yeast Mitochondrial F1-Atpase
☐ Check to include this hit in error tolerant search or archive report

Query	Observed	Mr(expt)	Mr(calc)	ppm	Miss	Score	Expect	Rank	Unique	Peptide
21	1435.8388	1434.8315	1434.7467	59.1	0	13	78	1	U	R.FTQAGSEVSALLGR.I
35	1585.9159	1584.9086	1584.8082	63.4	0	15	52	1	U	K.VALVFGQMNEPPGAR.A
39	1678.0309	1677.0236	1676.9210	61.2	0	42	0.059	1	U	K.LVLEVAQHLGENTVR.T

Proteins matching the same set of peptides:
pdb|2HLD|E **Mass:** 51095 **Score:** 70 **Matches:** 3(0) **Sequences:** 3(0)
Chain E, Crystal Structure Of Yeast Mitochondrial F1-Atpase
pdb|2HLD|F **Mass:** 51095 **Score:** 70 **Matches:** 3(0) **Sequences:** 3(0)
Chain F, Crystal Structure Of Yeast Mitochondrial F1-Atpase
pdb|2HLD|M **Mass:** 51095 **Score:** 70 **Matches:** 3(0) **Sequences:** 3(0)
Chain M, Crystal Structure Of Yeast Mitochondrial F1-Atpase
pdb|2HLD|N **Mass:** 51095 **Score:** 70 **Matches:** 3(0) **Sequences:** 3(0)
Chain N, Crystal Structure Of Yeast Mitochondrial F1-Atpase
pdb|2HLD|O **Mass:** 51095 **Score:** 70 **Matches:** 3(0) **Sequences:** 3(0)
Chain O, Crystal Structure Of Yeast Mitochondrial F1-Atpase
pdb|2HLD|V **Mass:** 51095 **Score:** 70 **Matches:** 3(0) **Sequences:** 3(0)
Chain V, Crystal Structure Of Yeast Mitochondrial F1-Atpase
pdb|2HLD|W **Mass:** 51095 **Score:** 70 **Matches:** 3(0) **Sequences:** 3(0)
Chain W, Crystal Structure Of Yeast Mitochondrial F1-Atpase
pdb|2HLD|X **Mass:** 51095 **Score:** 70 **Matches:** 3(0) **Sequences:** 3(0)
Chain X, Crystal Structure Of Yeast Mitochondrial F1-Atpase
pdb|2WPD|D **Mass:** 51095 **Score:** 70 **Matches:** 3(0) **Sequences:** 3(0)
Chain D, The Mg.Adp Inhibited State Of The Yeast F1c10 Atp Synthase
pdb|2WPD|E **Mass:** 51095 **Score:** 70 **Matches:** 3(0) **Sequences:** 3(0)
Chain E, The Mg.Adp Inhibited State Of The Yeast F1c10 Atp Synthase
pdb|2WPD|F **Mass:** 51095 **Score:** 70 **Matches:** 3(0) **Sequences:** 3(0)
Chain F, The Mg.Adp Inhibited State Of The Yeast F1c10 Atp Synthase

pdb|2XOK|D **Mass:** 54817 **Score:** 70 **Matches:** 3(0) **Sequences:** 3(0)
Chain D, Refined Structure Of Yeast F1c10 Atpase Complex To 3 A Resolution
pdb|2XOK|E **Mass:** 54817 **Score:** 70 **Matches:** 3(0) **Sequences:** 3(0)
Chain E, Refined Structure Of Yeast F1c10 Atpase Complex To 3 A Resolution
pdb|2XOK|F **Mass:** 54817 **Score:** 70 **Matches:** 3(0) **Sequences:** 3(0)
Chain F, Refined Structure Of Yeast F1c10 Atpase Complex To 3 A Resolution
pdb|3FKS|D **Mass:** 51917 **Score:** 70 **Matches:** 3(0) **Sequences:** 3(0)
Chain D, Yeast F1 Atpase In The Absence Of Bound Nucleotides
pdb|3FKS|E **Mass:** 51917 **Score:** 70 **Matches:** 3(0) **Sequences:** 3(0)
Chain E, Yeast F1 Atpase In The Absence Of Bound Nucleotides
pdb|3FKS|F **Mass:** 51917 **Score:** 70 **Matches:** 3(0) **Sequences:** 3(0)
Chain F, Yeast F1 Atpase In The Absence Of Bound Nucleotides
pdb|3FKS|M **Mass:** 51917 **Score:** 70 **Matches:** 3(0) **Sequences:** 3(0)
Chain M, Yeast F1 Atpase In The Absence Of Bound Nucleotides
pdb|3FKS|N **Mass:** 51917 **Score:** 70 **Matches:** 3(0) **Sequences:** 3(0)
Chain N, Yeast F1 Atpase In The Absence Of Bound Nucleotides
pdb|3FKS|O **Mass:** 51917 **Score:** 70 **Matches:** 3(0) **Sequences:** 3(0)
Chain O, Yeast F1 Atpase In The Absence Of Bound Nucleotides
pdb|3FKS|V **Mass:** 51917 **Score:** 70 **Matches:** 3(0) **Sequences:** 3(0)
Chain V, Yeast F1 Atpase In The Absence Of Bound Nucleotides
pdb|3FKS|W **Mass:** 51917 **Score:** 70 **Matches:** 3(0) **Sequences:** 3(0)
Chain W, Yeast F1 Atpase In The Absence Of Bound Nucleotides
pdb|3FKS|X **Mass:** 51917 **Score:** 70 **Matches:** 3(0) **Sequences:** 3(0)
Chain X, Yeast F1 Atpase In The Absence Of Bound Nucleotides
pdb|3OE7|D **Mass:** 51917 **Score:** 70 **Matches:** 3(0) **Sequences:** 3(0)
Chain D, Structure Of Four Mutant Forms Of Yeast F1 Atpase: Gamma-I270t
pdb|3OE7|E **Mass:** 51917 **Score:** 70 **Matches:** 3(0) **Sequences:** 3(0)
Chain E, Structure Of Four Mutant Forms Of Yeast F1 Atpase: Gamma-I270t
pdb|3OE7|F **Mass:** 51917 **Score:** 70 **Matches:** 3(0) **Sequences:** 3(0)
Chain F, Structure Of Four Mutant Forms Of Yeast F1 Atpase: Gamma-I270t
pdb|3OE7|M **Mass:** 51917 **Score:** 70 **Matches:** 3(0) **Sequences:** 3(0)
Chain M, Structure Of Four Mutant Forms Of Yeast F1 Atpase: Gamma-I270t
pdb|3OE7|N **Mass:** 51917 **Score:** 70 **Matches:** 3(0) **Sequences:** 3(0)
Chain N, Structure Of Four Mutant Forms Of Yeast F1 Atpase: Gamma-I270t
pdb|3OE7|O **Mass:** 51917 **Score:** 70 **Matches:** 3(0) **Sequences:** 3(0)
Chain O, Structure Of Four Mutant Forms Of Yeast F1 Atpase: Gamma-I270t
pdb|3OE7|V **Mass:** 51917 **Score:** 70 **Matches:** 3(0) **Sequences:** 3(0)
Chain V, Structure Of Four Mutant Forms Of Yeast F1 Atpase: Gamma-I270t
pdb|3OE7|W **Mass:** 51917 **Score:** 70 **Matches:** 3(0) **Sequences:** 3(0)
Chain W, Structure Of Four Mutant Forms Of Yeast F1 Atpase: Gamma-I270t
pdb|3OE7|X **Mass:** 51917 **Score:** 70 **Matches:** 3(0) **Sequences:** 3(0)
Chain X, Structure Of Four Mutant Forms Of Yeast F1 Atpase: Gamma-I270t
pdb|3OEE|D **Mass:** 51917 **Score:** 70 **Matches:** 3(0) **Sequences:** 3(0)
Chain D, Structure Of Four Mutant Forms Of Yeast F1 Atpase: Alpha-F405s
pdb|3OEE|E **Mass:** 51917 **Score:** 70 **Matches:** 3(0) **Sequences:** 3(0)
Chain E, Structure Of Four Mutant Forms Of Yeast F1 Atpase: Alpha-F405s
pdb|3OEE|F **Mass:** 51917 **Score:** 70 **Matches:** 3(0) **Sequences:** 3(0)
Chain F, Structure Of Four Mutant Forms Of Yeast F1 Atpase: Alpha-F405s
pdb|3OEE|M **Mass:** 51917 **Score:** 70 **Matches:** 3(0) **Sequences:** 3(0)
Chain M, Structure Of Four Mutant Forms Of Yeast F1 Atpase: Alpha-F405s
pdb|3OEE|N **Mass:** 51917 **Score:** 70 **Matches:** 3(0) **Sequences:** 3(0)
Chain N, Structure Of Four Mutant Forms Of Yeast F1 Atpase: Alpha-F405s
pdb|3OEE|O **Mass:** 51917 **Score:** 70 **Matches:** 3(0) **Sequences:** 3(0)
Chain O, Structure Of Four Mutant Forms Of Yeast F1 Atpase: Alpha-F405s
pdb|3OEE|V **Mass:** 51917 **Score:** 70 **Matches:** 3(0) **Sequences:** 3(0)
Chain V, Structure Of Four Mutant Forms Of Yeast F1 Atpase: Alpha-F405s
pdb|3OEE|W **Mass:** 51917 **Score:** 70 **Matches:** 3(0) **Sequences:** 3(0)
Chain W, Structure Of Four Mutant Forms Of Yeast F1 Atpase: Alpha-F405s
pdb|3OEE|X **Mass:** 51917 **Score:** 70 **Matches:** 3(0) **Sequences:** 3(0)
Chain X, Structure Of Four Mutant Forms Of Yeast F1 Atpase: Alpha-F405s
pdb|3OEH|D **Mass:** 51965 **Score:** 70 **Matches:** 3(0) **Sequences:** 3(0)
Chain D, Structure Of Four Mutant Forms Of Yeast F1 Atpase: Beta-V279f
pdb|3OEH|E **Mass:** 51965 **Score:** 70 **Matches:** 3(0) **Sequences:** 3(0)
Chain E, Structure Of Four Mutant Forms Of Yeast F1 Atpase: Beta-V279f
pdb|3OEH|F **Mass:** 51965 **Score:** 70 **Matches:** 3(0) **Sequences:** 3(0)
Chain F, Structure Of Four Mutant Forms Of Yeast F1 Atpase: Beta-V279f
pdb|3OEH|M **Mass:** 51965 **Score:** 70 **Matches:** 3(0) **Sequences:** 3(0)
Chain M, Structure Of Four Mutant Forms Of Yeast F1 Atpase: Beta-V279f
pdb|3OEH|N **Mass:** 51965 **Score:** 70 **Matches:** 3(0) **Sequences:** 3(0)
Chain N, Structure Of Four Mutant Forms Of Yeast F1 Atpase: Beta-V279f
pdb|3OEH|O **Mass:** 51965 **Score:** 70 **Matches:** 3(0) **Sequences:** 3(0)
Chain O, Structure Of Four Mutant Forms Of Yeast F1 Atpase: Beta-V279f
pdb|3OEH|V **Mass:** 51965 **Score:** 70 **Matches:** 3(0) **Sequences:** 3(0)
Chain V, Structure Of Four Mutant Forms Of Yeast F1 Atpase: Beta-V279f
pdb|3OEH|W **Mass:** 51965 **Score:** 70 **Matches:** 3(0) **Sequences:** 3(0)
Chain W, Structure Of Four Mutant Forms Of Yeast F1 Atpase: Beta-V279f
pdb|3OEH|X **Mass:** 51965 **Score:** 70 **Matches:** 3(0) **Sequences:** 3(0)
Chain X, Structure Of Four Mutant Forms Of Yeast F1 Atpase: Beta-V279f
pdb|3OFN|D **Mass:** 51917 **Score:** 70 **Matches:** 3(0) **Sequences:** 3(0)
Chain D, Structure Of Four Mutant Forms Of Yeast F1 Atpase: Alpha-N67i
pdb|3OFN|E **Mass:** 51917 **Score:** 70 **Matches:** 3(0) **Sequences:** 3(0)
Chain E, Structure Of Four Mutant Forms Of Yeast F1 Atpase: Alpha-N67i
pdb|3OFN|F **Mass:** 51917 **Score:** 70 **Matches:** 3(0) **Sequences:** 3(0)
Chain F, Structure Of Four Mutant Forms Of Yeast F1 Atpase: Alpha-N67i
pdb|3OFN|M **Mass:** 51917 **Score:** 70 **Matches:** 3(0) **Sequences:** 3(0)
Chain M, Structure Of Four Mutant Forms Of Yeast F1 Atpase: Alpha-N67i
pdb|3OFN|N **Mass:** 51917 **Score:** 70 **Matches:** 3(0) **Sequences:** 3(0)

Chain N, Structure Of Four Mutant Forms Of Yeast F1 Atpase: Alpha-N67i
pdb|3OFN|O **Mass:** 51917 **Score:** 70 **Matches:** 3(0) **Sequences:** 3(0)
Chain O, Structure Of Four Mutant Forms Of Yeast F1 Atpase: Alpha-N67i
pdb|3OFN|V **Mass:** 51917 **Score:** 70 **Matches:** 3(0) **Sequences:** 3(0)
Chain V, Structure Of Four Mutant Forms Of Yeast F1 Atpase: Alpha-N67i
pdb|3OFN|W **Mass:** 51917 **Score:** 70 **Matches:** 3(0) **Sequences:** 3(0)
Chain W, Structure Of Four Mutant Forms Of Yeast F1 Atpase: Alpha-N67i
pdb|3OFN|X **Mass:** 51917 **Score:** 70 **Matches:** 3(0) **Sequences:** 3(0)
Chain X, Structure Of Four Mutant Forms Of Yeast F1 Atpase: Alpha-N67i
pdb|3ZIA|D **Mass:** 51095 **Score:** 70 **Matches:** 3(0) **Sequences:** 3(0)
Chain D, The Structure Of F1-atpase From Saccharomyces Cerevisiae Inhibited By Its Regulatory Protein If1
pdb|3ZIA|E **Mass:** 51095 **Score:** 70 **Matches:** 3(0) **Sequences:** 3(0)
Chain E, The Structure Of F1-atpase From Saccharomyces Cerevisiae Inhibited By Its Regulatory Protein If1
pdb|3ZIA|F **Mass:** 51095 **Score:** 70 **Matches:** 3(0) **Sequences:** 3(0)
Chain F, The Structure Of F1-atpase From Saccharomyces Cerevisiae Inhibited By Its Regulatory Protein If1
pdb|3ZIA|N **Mass:** 51095 **Score:** 70 **Matches:** 3(0) **Sequences:** 3(0)
Chain N, The Structure Of F1-atpase From Saccharomyces Cerevisiae Inhibited By Its Regulatory Protein If1
pdb|3ZIA|O **Mass:** 51095 **Score:** 70 **Matches:** 3(0) **Sequences:** 3(0)
Chain O, The Structure Of F1-atpase From Saccharomyces Cerevisiae Inhibited By Its Regulatory Protein If1
pdb|3ZIA|P **Mass:** 51095 **Score:** 70 **Matches:** 3(0) **Sequences:** 3(0)
Chain P, The Structure Of F1-atpase From Saccharomyces Cerevisiae Inhibited By Its Regulatory Protein If1
pdb|3ZRY|D **Mass:** 51095 **Score:** 70 **Matches:** 3(0) **Sequences:** 3(0)
Chain D, Rotor Architecture In The F(1)-C(10)-Ring Complex Of The Yeast F-Atp Synthase
pdb|3ZRY|E **Mass:** 51095 **Score:** 70 **Matches:** 3(0) **Sequences:** 3(0)
Chain E, Rotor Architecture In The F(1)-C(10)-Ring Complex Of The Yeast F-Atp Synthase
pdb|3ZRY|F **Mass:** 51095 **Score:** 70 **Matches:** 3(0) **Sequences:** 3(0)
Chain F, Rotor Architecture In The F(1)-C(10)-Ring Complex Of The Yeast F-Atp Synthase
pdb|4B2Q|D **Mass:** 50353 **Score:** 70 **Matches:** 3(0) **Sequences:** 3(0)
Chain D, Model Of The Yeast F1fo-atp Synthase Dimer Based On Subtomogram Average
pdb|4B2Q|DD **Mass:** 50353 **Score:** 70 **Matches:** 3(0) **Sequences:** 3(0)
Chain d, Model Of The Yeast F1fo-atp Synthase Dimer Based On Subtomogram Average
pdb|4B2Q|E **Mass:** 50667 **Score:** 70 **Matches:** 3(0) **Sequences:** 3(0)
Chain E, Model Of The Yeast F1fo-atp Synthase Dimer Based On Subtomogram Average
pdb|4B2Q|EE **Mass:** 50667 **Score:** 70 **Matches:** 3(0) **Sequences:** 3(0)
Chain e, Model Of The Yeast F1fo-atp Synthase Dimer Based On Subtomogram Average
pdb|4B2Q|F **Mass:** 50667 **Score:** 70 **Matches:** 3(0) **Sequences:** 3(0)
Chain F, Model Of The Yeast F1fo-atp Synthase Dimer Based On Subtomogram Average
pdb|4B2Q|FF **Mass:** 50667 **Score:** 70 **Matches:** 3(0) **Sequences:** 3(0)
Chain f, Model Of The Yeast F1fo-atp Synthase Dimer Based On Subtomogram Average
emb|CAH00634.1| **Mass:** 54035 **Score:** 70 **Matches:** 3(0) **Sequences:** 3(0)
KLLA0D10703p [Kluyveromyces lactis]
emb|CAY05714.1| **Mass:** 54817 **Score:** 70 **Matches:** 3(0) **Sequences:** 3(0)
unnamed protein product [Saccharomyces cerevisiae]
emb|CCD24340.1| **Mass:** 54837 **Score:** 70 **Matches:** 3(0) **Sequences:** 3(0)
hypothetical protein NDAI_0D00260 [Naumovozyma dairenensis CBS 421]
gb|AAA34444.1| **Mass:** 54947 **Score:** 70 **Matches:** 3(0) **Sequences:** 3(0)
F1-ATPase beta-subunit precursor [Saccharomyces cerevisiae]
gb|AJV38860.1| **Mass:** 54817 **Score:** 70 **Matches:** 3(0) **Sequences:** 3(0)
Atp2p [Saccharomyces cerevisiae YJM1273]
gb|AJV39180.1| **Mass:** 54831 **Score:** 70 **Matches:** 3(0) **Sequences:** 3(0)
Atp2p [Saccharomyces cerevisiae YJM1304]
gb|AJV39504.1| **Mass:** 54817 **Score:** 70 **Matches:** 3(0) **Sequences:** 3(0)
Atp2p [Saccharomyces cerevisiae YJM1307]
gb|AJP39802.1| **Mass:** 54817 **Score:** 70 **Matches:** 3(0) **Sequences:** 3(0)
Atp2p [Saccharomyces cerevisiae YJM1078]
gb|AJV39836.1| **Mass:** 54817 **Score:** 70 **Matches:** 3(0) **Sequences:** 3(0)
Atp2p [Saccharomyces cerevisiae YJM1311]
gb|AJV40151.1| **Mass:** 54817 **Score:** 70 **Matches:** 3(0) **Sequences:** 3(0)
Atp2p [Saccharomyces cerevisiae YJM1326]
gb|AJV40478.1| **Mass:** 54831 **Score:** 70 **Matches:** 3(0) **Sequences:** 3(0)
Atp2p [Saccharomyces cerevisiae YJM1332]
gb|AJV40804.1| **Mass:** 54831 **Score:** 70 **Matches:** 3(0) **Sequences:** 3(0)
Atp2p [Saccharomyces cerevisiae YJM1336]
gb|AJV41132.1| **Mass:** 54817 **Score:** 70 **Matches:** 3(0) **Sequences:** 3(0)
Atp2p [Saccharomyces cerevisiae YJM1338]
gb|AJV41461.1| **Mass:** 54831 **Score:** 70 **Matches:** 3(0) **Sequences:** 3(0)
Atp2p [Saccharomyces cerevisiae YJM1341]
gb|AJV41780.1| **Mass:** 54817 **Score:** 70 **Matches:** 3(0) **Sequences:** 3(0)
Atp2p [Saccharomyces cerevisiae YJM1342]
gb|AJV42108.1| **Mass:** 54831 **Score:** 70 **Matches:** 3(0) **Sequences:** 3(0)
Atp2p [Saccharomyces cerevisiae YJM1355]
gb|AJV42435.1| **Mass:** 54831 **Score:** 70 **Matches:** 3(0) **Sequences:** 3(0)
Atp2p [Saccharomyces cerevisiae YJM1356]
gb|AJV42762.1| **Mass:** 54817 **Score:** 70 **Matches:** 3(0) **Sequences:** 3(0)
Atp2p [Saccharomyces cerevisiae YJM1381]
gb|AJV43091.1| **Mass:** 54817 **Score:** 70 **Matches:** 3(0) **Sequences:** 3(0)
Atp2p [Saccharomyces cerevisiae YJM1383]
gb|AJV43421.1| **Mass:** 54817 **Score:** 70 **Matches:** 3(0) **Sequences:** 3(0)
Atp2p [Saccharomyces cerevisiae YJM1385]
gb|AJV43744.1| **Mass:** 54817 **Score:** 70 **Matches:** 3(0) **Sequences:** 3(0)
Atp2p [Saccharomyces cerevisiae YJM1386]
gb|AJV44074.1| **Mass:** 54831 **Score:** 70 **Matches:** 3(0) **Sequences:** 3(0)
Atp2p [Saccharomyces cerevisiae YJM1387]
gb|AJV44400.1| **Mass:** 54831 **Score:** 70 **Matches:** 3(0) **Sequences:** 3(0)
Atp2p [Saccharomyces cerevisiae YJM1388]
gb|AJV44731.1| **Mass:** 54831 **Score:** 70 **Matches:** 3(0) **Sequences:** 3(0)
Atp2p [Saccharomyces cerevisiae YJM1389]
gb|AJV45056.1| **Mass:** 54817 **Score:** 70 **Matches:** 3(0) **Sequences:** 3(0)

Atp2p [Saccharomyces cerevisiae YJM1399]
gb|AJV45384.1| **Mass:** 54817 **Score:** 70 **Matches:** 3(0) **Sequences:** 3(0)
Atp2p [Saccharomyces cerevisiae YJM1400]
gb|AJV45672.1| **Mass:** 54817 **Score:** 70 **Matches:** 3(0) **Sequences:** 3(0)
Atp2p [Saccharomyces cerevisiae YJM1401]
gb|AJV46001.1| **Mass:** 54817 **Score:** 70 **Matches:** 3(0) **Sequences:** 3(0)
Atp2p [Saccharomyces cerevisiae YJM1402]
emb|CDR46351.1| **Mass:** 54085 **Score:** 70 **Matches:** 3(0) **Sequences:** 3(0)
CYFA0S23e00100g1_1 [Cyberlindnera fabianii]
gb|AAC49475.1| **Mass:** 54817 **Score:** 70 **Matches:** 3(0) **Sequences:** 3(0)
F1-ATPase beta-subunit [Saccharomyces cerevisiae]
gb|AAS50941.2| **Mass:** 54067 **Score:** 70 **Matches:** 3(0) **Sequences:** 3(0)
ABR169Wp [Eremothecium gossypii ATCC 10895]
gb|AJR54065.1| **Mass:** 54831 **Score:** 70 **Matches:** 3(0) **Sequences:** 3(0)
Atp2p [Saccharomyces cerevisiae YJM681]
gb|AJR54396.1| **Mass:** 54817 **Score:** 70 **Matches:** 3(0) **Sequences:** 3(0)
Atp2p [Saccharomyces cerevisiae YJM682]
gb|AJR54731.1| **Mass:** 54817 **Score:** 70 **Matches:** 3(0) **Sequences:** 3(0)
Atp2p [Saccharomyces cerevisiae YJM683]
gb|AJR55056.1| **Mass:** 54817 **Score:** 70 **Matches:** 3(0) **Sequences:** 3(0)
Atp2p [Saccharomyces cerevisiae YJM689]
gb|AJR55365.1| **Mass:** 54817 **Score:** 70 **Matches:** 3(0) **Sequences:** 3(0)
Atp2p [Saccharomyces cerevisiae YJM693]
gb|AJR55694.1| **Mass:** 54831 **Score:** 70 **Matches:** 3(0) **Sequences:** 3(0)
Atp2p [Saccharomyces cerevisiae YJM969]
gb|AJR56024.1| **Mass:** 54831 **Score:** 70 **Matches:** 3(0) **Sequences:** 3(0)
Atp2p [Saccharomyces cerevisiae YJM972]
gb|AJR56351.1| **Mass:** 54831 **Score:** 70 **Matches:** 3(0) **Sequences:** 3(0)
Atp2p [Saccharomyces cerevisiae YJM975]
gb|AJR56678.1| **Mass:** 54831 **Score:** 70 **Matches:** 3(0) **Sequences:** 3(0)
Atp2p [Saccharomyces cerevisiae YJM978]
gb|AJR57004.1| **Mass:** 54831 **Score:** 70 **Matches:** 3(0) **Sequences:** 3(0)
Atp2p [Saccharomyces cerevisiae YJM981]
gb|AJR57331.1| **Mass:** 54831 **Score:** 70 **Matches:** 3(0) **Sequences:** 3(0)
Atp2p [Saccharomyces cerevisiae YJM984]
gb|AJR57662.1| **Mass:** 54831 **Score:** 70 **Matches:** 3(0) **Sequences:** 3(0)
Atp2p [Saccharomyces cerevisiae YJM987]
gb|AJR57990.1| **Mass:** 54831 **Score:** 70 **Matches:** 3(0) **Sequences:** 3(0)
Atp2p [Saccharomyces cerevisiae YJM990]
gb|AJR58318.1| **Mass:** 54831 **Score:** 70 **Matches:** 3(0) **Sequences:** 3(0)
Atp2p [Saccharomyces cerevisiae YJM996]
gb|AJR58642.1| **Mass:** 54817 **Score:** 70 **Matches:** 3(0) **Sequences:** 3(0)
Atp2p [Saccharomyces cerevisiae YJM1083]
gb|AJR58970.1| **Mass:** 54831 **Score:** 70 **Matches:** 3(0) **Sequences:** 3(0)
Atp2p [Saccharomyces cerevisiae YJM1129]
gb|AJR59295.1| **Mass:** 54831 **Score:** 70 **Matches:** 3(0) **Sequences:** 3(0)
Atp2p [Saccharomyces cerevisiae YJM1133]
gb|AJR59622.1| **Mass:** 54831 **Score:** 70 **Matches:** 3(0) **Sequences:** 3(0)
Atp2p [Saccharomyces cerevisiae YJM189]
emb|CAG59751.1| **Mass:** 54176 **Score:** 70 **Matches:** 3(0) **Sequences:** 3(0)
unnamed protein product [Candida glabrata]
gb|AJR59953.1| **Mass:** 54817 **Score:** 70 **Matches:** 3(0) **Sequences:** 3(0)
Atp2p [Saccharomyces cerevisiae YJM193]
gb|AJR60276.1| **Mass:** 54817 **Score:** 70 **Matches:** 3(0) **Sequences:** 3(0)
Atp2p [Saccharomyces cerevisiae YJM195]
gb|AJR60610.1| **Mass:** 54831 **Score:** 70 **Matches:** 3(0) **Sequences:** 3(0)
Atp2p [Saccharomyces cerevisiae YJM244]
gb|AJR60938.1| **Mass:** 54817 **Score:** 70 **Matches:** 3(0) **Sequences:** 3(0)
Atp2p [Saccharomyces cerevisiae YJM248]
gb|AJR61261.1| **Mass:** 54889 **Score:** 70 **Matches:** 3(0) **Sequences:** 3(0)
Atp2p [Saccharomyces cerevisiae YJM270]
gb|AJR61589.1| **Mass:** 54831 **Score:** 70 **Matches:** 3(0) **Sequences:** 3(0)
Atp2p [Saccharomyces cerevisiae YJM271]
gb|AJR61912.1| **Mass:** 54817 **Score:** 70 **Matches:** 3(0) **Sequences:** 3(0)
Atp2p [Saccharomyces cerevisiae YJM320]
gb|AJR62240.1| **Mass:** 54817 **Score:** 70 **Matches:** 3(0) **Sequences:** 3(0)
Atp2p [Saccharomyces cerevisiae YJM326]
gb|AJR62573.1| **Mass:** 54817 **Score:** 70 **Matches:** 3(0) **Sequences:** 3(0)
Atp2p [Saccharomyces cerevisiae YJM428]
gb|AJR62894.1| **Mass:** 54817 **Score:** 70 **Matches:** 3(0) **Sequences:** 3(0)
Atp2p [Saccharomyces cerevisiae YJM450]
gb|AJR63223.1| **Mass:** 54817 **Score:** 70 **Matches:** 3(0) **Sequences:** 3(0)
Atp2p [Saccharomyces cerevisiae YJM451]
gb|AJR63550.1| **Mass:** 54831 **Score:** 70 **Matches:** 3(0) **Sequences:** 3(0)
Atp2p [Saccharomyces cerevisiae YJM453]
gb|AJR63878.1| **Mass:** 54817 **Score:** 70 **Matches:** 3(0) **Sequences:** 3(0)
Atp2p [Saccharomyces cerevisiae YJM456]
gb|AJR64203.1| **Mass:** 54817 **Score:** 70 **Matches:** 3(0) **Sequences:** 3(0)
Atp2p [Saccharomyces cerevisiae YJM470]
gb|AJR64499.1| **Mass:** 54831 **Score:** 70 **Matches:** 3(0) **Sequences:** 3(0)
Atp2p [Saccharomyces cerevisiae YJM541]
gb|AJR64766.1| **Mass:** 54831 **Score:** 70 **Matches:** 3(0) **Sequences:** 3(0)
Atp2p [Saccharomyces cerevisiae YJM554]
gb|AJR65093.1| **Mass:** 54831 **Score:** 70 **Matches:** 3(0) **Sequences:** 3(0)
Atp2p [Saccharomyces cerevisiae YJM555]
gb|AJR65424.1| **Mass:** 54817 **Score:** 70 **Matches:** 3(0) **Sequences:** 3(0)
Atp2p [Saccharomyces cerevisiae YJM627]
gb|AJR65753.1| **Mass:** 54817 **Score:** 70 **Matches:** 3(0) **Sequences:** 3(0)

Atp2p [Saccharomyces cerevisiae YJM1190]
gb|AJR66081.1| **Mass:** 54817 **Score:** 70 **Matches:** 3(0) **Sequences:** 3(0)
Atp2p [Saccharomyces cerevisiae YJM1199]
gb|AJR66404.1| **Mass:** 54817 **Score:** 70 **Matches:** 3(0) **Sequences:** 3(0)
Atp2p [Saccharomyces cerevisiae YJM1202]
gb|AJR66729.1| **Mass:** 54817 **Score:** 70 **Matches:** 3(0) **Sequences:** 3(0)
Atp2p [Saccharomyces cerevisiae YJM1208]
gb|AJR67056.1| **Mass:** 54831 **Score:** 70 **Matches:** 3(0) **Sequences:** 3(0)
Atp2p [Saccharomyces cerevisiae YJM1242]
gb|AJR67384.1| **Mass:** 54831 **Score:** 70 **Matches:** 3(0) **Sequences:** 3(0)
Atp2p [Saccharomyces cerevisiae YJM1244]
gb|AJR67680.1| **Mass:** 54817 **Score:** 70 **Matches:** 3(0) **Sequences:** 3(0)
Atp2p [Saccharomyces cerevisiae YJM1248]
gb|AJR68008.1| **Mass:** 54817 **Score:** 70 **Matches:** 3(0) **Sequences:** 3(0)
Atp2p [Saccharomyces cerevisiae YJM1250]
gb|AJR68337.1| **Mass:** 54817 **Score:** 70 **Matches:** 3(0) **Sequences:** 3(0)
Atp2p [Saccharomyces cerevisiae YJM1252]
gb|AJR68654.1| **Mass:** 54831 **Score:** 70 **Matches:** 3(0) **Sequences:** 3(0)
Atp2p [Saccharomyces cerevisiae YJM1415]
gb|AJR68978.1| **Mass:** 54831 **Score:** 70 **Matches:** 3(0) **Sequences:** 3(0)
Atp2p [Saccharomyces cerevisiae YJM1417]
gb|AJR69287.1| **Mass:** 54817 **Score:** 70 **Matches:** 3(0) **Sequences:** 3(0)
Atp2p [Saccharomyces cerevisiae YJM1418]
gb|AJR69616.1| **Mass:** 54817 **Score:** 70 **Matches:** 3(0) **Sequences:** 3(0)
Atp2p [Saccharomyces cerevisiae YJM1419]
gb|AJR69948.1| **Mass:** 54831 **Score:** 70 **Matches:** 3(0) **Sequences:** 3(0)
Atp2p [Saccharomyces cerevisiae YJM1433]
gb|AJR70282.1| **Mass:** 54817 **Score:** 70 **Matches:** 3(0) **Sequences:** 3(0)
Atp2p [Saccharomyces cerevisiae YJM1434]
gb|AJR70610.1| **Mass:** 54817 **Score:** 70 **Matches:** 3(0) **Sequences:** 3(0)
Atp2p [Saccharomyces cerevisiae YJM1439]
gb|AJR70941.1| **Mass:** 54817 **Score:** 70 **Matches:** 3(0) **Sequences:** 3(0)
Atp2p [Saccharomyces cerevisiae YJM1443]
gb|AJR71271.1| **Mass:** 54817 **Score:** 70 **Matches:** 3(0) **Sequences:** 3(0)
Atp2p [Saccharomyces cerevisiae YJM1444]
gb|AJR71580.1| **Mass:** 54817 **Score:** 70 **Matches:** 3(0) **Sequences:** 3(0)
Atp2p [Saccharomyces cerevisiae YJM1447]
gb|AJR71908.1| **Mass:** 54817 **Score:** 70 **Matches:** 3(0) **Sequences:** 3(0)
Atp2p [Saccharomyces cerevisiae YJM1450]
gb|AJR72239.1| **Mass:** 54831 **Score:** 70 **Matches:** 3(0) **Sequences:** 3(0)
Atp2p [Saccharomyces cerevisiae YJM1460]
gb|AJR72567.1| **Mass:** 54817 **Score:** 70 **Matches:** 3(0) **Sequences:** 3(0)
Atp2p [Saccharomyces cerevisiae YJM1463]
gb|AJR72893.1| **Mass:** 54831 **Score:** 70 **Matches:** 3(0) **Sequences:** 3(0)
Atp2p [Saccharomyces cerevisiae YJM1477]
gb|AJR73224.1| **Mass:** 54817 **Score:** 70 **Matches:** 3(0) **Sequences:** 3(0)
Atp2p [Saccharomyces cerevisiae YJM1478]
gb|AJR73555.1| **Mass:** 54817 **Score:** 70 **Matches:** 3(0) **Sequences:** 3(0)
Atp2p [Saccharomyces cerevisiae YJM1479]
gb|AJR73871.1| **Mass:** 54861 **Score:** 70 **Matches:** 3(0) **Sequences:** 3(0)
Atp2p [Saccharomyces cerevisiae YJM1526]
gb|AJR74189.1| **Mass:** 54817 **Score:** 70 **Matches:** 3(0) **Sequences:** 3(0)
Atp2p [Saccharomyces cerevisiae YJM1527]
gb|AJR74516.1| **Mass:** 54817 **Score:** 70 **Matches:** 3(0) **Sequences:** 3(0)
Atp2p [Saccharomyces cerevisiae YJM1549]
gb|AJR74846.1| **Mass:** 54817 **Score:** 70 **Matches:** 3(0) **Sequences:** 3(0)
Atp2p [Saccharomyces cerevisiae YJM1573]
gb|AJR75181.1| **Mass:** 54831 **Score:** 70 **Matches:** 3(0) **Sequences:** 3(0)
Atp2p [Saccharomyces cerevisiae YJM1574]
gb|AJR75511.1| **Mass:** 54831 **Score:** 70 **Matches:** 3(0) **Sequences:** 3(0)
Atp2p [Saccharomyces cerevisiae YJM1592]
gb|AJR75840.1| **Mass:** 54817 **Score:** 70 **Matches:** 3(0) **Sequences:** 3(0)
Atp2p [Saccharomyces cerevisiae YJM1615]
gb|AHY79101.1| **Mass:** 54831 **Score:** 70 **Matches:** 3(0) **Sequences:** 3(0)
Atp2p [Saccharomyces cerevisiae YJM993]
emb|CAY80835.1| **Mass:** 54889 **Score:** 70 **Matches:** 3(0) **Sequences:** 3(0)
Atp2p [Saccharomyces cerevisiae EC1118]
emb|CAA89652.1| **Mass:** 54817 **Score:** 70 **Matches:** 3(0) **Sequences:** 3(0)
ATP2 [Saccharomyces cerevisiae]
gb|AEY95231.1| **Mass:** 54067 **Score:** 70 **Matches:** 3(0) **Sequences:** 3(0)
FABR169Wp [Eremothecium gossypii FDAG1]

---

6. gb|AJU16346.1| **Mass:** 17388 **Score:** 69 **Matches:** 1(1) **Sequences:** 1(1)
Cox6p [Saccharomyces cerevisiae YJM1356]
☐ Check to include this hit in error tolerant search or archive report

Query	Observed	Mr(expt)	Mr(calc)	ppm	Miss	Score	Expect	Rank	Unique	Peptide
69	2081.0684	2080.0611	2079.9425	57.0	1	69	0.00019	1	U	R.YEKEFDEAYDLFEVQR.V

Proteins matching the same set of peptides:
gb|AJU16605.1| **Mass:** 17388 **Score:** 69 **Matches:** 1(1) **Sequences:** 1(1)
Cox6p [Saccharomyces cerevisiae YJM1381]
gb|AJU16865.1| **Mass:** 17388 **Score:** 69 **Matches:** 1(1) **Sequences:** 1(1)
Cox6p [Saccharomyces cerevisiae YJM1383]
gb|AJU17117.1| **Mass:** 17388 **Score:** 69 **Matches:** 1(1) **Sequences:** 1(1)
Cox6p [Saccharomyces cerevisiae YJM1385]
gb|AJU17372.1| **Mass:** 17388 **Score:** 69 **Matches:** 1(1) **Sequences:** 1(1)

Cox6p [Saccharomyces cerevisiae YJM1386]
gb|AJU17632.1| **Mass:** 17388 **Score:** 69 **Matches:** 1(1) **Sequences:** 1(1)
Cox6p [Saccharomyces cerevisiae YJM1387]
gb|AJU17893.1| **Mass:** 17388 **Score:** 69 **Matches:** 1(1) **Sequences:** 1(1)
Cox6p [Saccharomyces cerevisiae YJM1388]
gb|AJU18147.1| **Mass:** 17388 **Score:** 69 **Matches:** 1(1) **Sequences:** 1(1)
Cox6p [Saccharomyces cerevisiae YJM1389]
gb|AJU18392.1| **Mass:** 17388 **Score:** 69 **Matches:** 1(1) **Sequences:** 1(1)
Cox6p [Saccharomyces cerevisiae YJM1399]
gb|AJU18642.1| **Mass:** 17374 **Score:** 69 **Matches:** 1(1) **Sequences:** 1(1)
Cox6p [Saccharomyces cerevisiae YJM1400]
gb|AJU18889.1| **Mass:** 17374 **Score:** 69 **Matches:** 1(1) **Sequences:** 1(1)
Cox6p [Saccharomyces cerevisiae YJM1401]
gb|AJU19141.1| **Mass:** 17388 **Score:** 69 **Matches:** 1(1) **Sequences:** 1(1)
Cox6p [Saccharomyces cerevisiae YJM1402]
gb|AJU19396.1| **Mass:** 17388 **Score:** 69 **Matches:** 1(1) **Sequences:** 1(1)
Cox6p [Saccharomyces cerevisiae YJM1415]
gb|AJU19654.1| **Mass:** 17388 **Score:** 69 **Matches:** 1(1) **Sequences:** 1(1)
Cox6p [Saccharomyces cerevisiae YJM1417]
gb|AJU19912.1| **Mass:** 17388 **Score:** 69 **Matches:** 1(1) **Sequences:** 1(1)
Cox6p [Saccharomyces cerevisiae YJM1418]
gb|AJU20166.1| **Mass:** 17388 **Score:** 69 **Matches:** 1(1) **Sequences:** 1(1)
Cox6p [Saccharomyces cerevisiae YJM1419]
gb|AJU20403.1| **Mass:** 17388 **Score:** 69 **Matches:** 1(1) **Sequences:** 1(1)
Cox6p [Saccharomyces cerevisiae YJM1433]
gb|AJU20664.1| **Mass:** 17388 **Score:** 69 **Matches:** 1(1) **Sequences:** 1(1)
Cox6p [Saccharomyces cerevisiae YJM1434]
gb|AJU20911.1| **Mass:** 17303 **Score:** 69 **Matches:** 1(1) **Sequences:** 1(1)
Cox6p [Saccharomyces cerevisiae YJM1439]
gb|AJU21154.1| **Mass:** 17388 **Score:** 69 **Matches:** 1(1) **Sequences:** 1(1)
Cox6p [Saccharomyces cerevisiae YJM1443]
gb|AJU21380.1| **Mass:** 17388 **Score:** 69 **Matches:** 1(1) **Sequences:** 1(1)
Cox6p [Saccharomyces cerevisiae YJM1444]
gb|AJU21626.1| **Mass:** 17388 **Score:** 69 **Matches:** 1(1) **Sequences:** 1(1)
Cox6p [Saccharomyces cerevisiae YJM1447]
gb|AJU21880.1| **Mass:** 17388 **Score:** 69 **Matches:** 1(1) **Sequences:** 1(1)
Cox6p [Saccharomyces cerevisiae YJM1450]
gb|AJU22139.1| **Mass:** 17388 **Score:** 69 **Matches:** 1(1) **Sequences:** 1(1)
Cox6p [Saccharomyces cerevisiae YJM1460]
gb|AJU22395.1| **Mass:** 17388 **Score:** 69 **Matches:** 1(1) **Sequences:** 1(1)
Cox6p [Saccharomyces cerevisiae YJM1463]
gb|AJU22651.1| **Mass:** 17388 **Score:** 69 **Matches:** 1(1) **Sequences:** 1(1)
Cox6p [Saccharomyces cerevisiae YJM1477]
gb|AJU22911.1| **Mass:** 17388 **Score:** 69 **Matches:** 1(1) **Sequences:** 1(1)
Cox6p [Saccharomyces cerevisiae YJM1478]
gb|AJU23168.1| **Mass:** 17374 **Score:** 69 **Matches:** 1(1) **Sequences:** 1(1)
Cox6p [Saccharomyces cerevisiae YJM1479]
gb|AJU23593.1| **Mass:** 17388 **Score:** 69 **Matches:** 1(1) **Sequences:** 1(1)
Cox6p [Saccharomyces cerevisiae YJM1526]
gb|AJU23851.1| **Mass:** 17388 **Score:** 69 **Matches:** 1(1) **Sequences:** 1(1)
Cox6p [Saccharomyces cerevisiae YJM1527]
gb|AJU24279.1| **Mass:** 17388 **Score:** 69 **Matches:** 1(1) **Sequences:** 1(1)
Cox6p [Saccharomyces cerevisiae YJM1549]
emb|CCD24479.1| **Mass:** 18285 **Score:** 69 **Matches:** 1(1) **Sequences:** 1(1)
hypothetical protein NDAI_0D01650 [Naumovozyma dairenensis CBS 421]
gb|AJU24533.1| **Mass:** 17388 **Score:** 69 **Matches:** 1(1) **Sequences:** 1(1)
Cox6p [Saccharomyces cerevisiae YJM1573]
gb|AJU24951.1| **Mass:** 17388 **Score:** 69 **Matches:** 1(1) **Sequences:** 1(1)
Cox6p [Saccharomyces cerevisiae YJM1574]
gb|AJU25201.1| **Mass:** 17388 **Score:** 69 **Matches:** 1(1) **Sequences:** 1(1)
Cox6p [Saccharomyces cerevisiae YJM1592]
gb|AJU25454.1| **Mass:** 17388 **Score:** 69 **Matches:** 1(1) **Sequences:** 1(1)
Cox6p [Saccharomyces cerevisiae YJM1615]
gb|AJU25885.1| **Mass:** 17388 **Score:** 69 **Matches:** 1(1) **Sequences:** 1(1)
Cox6p [Saccharomyces cerevisiae YJM189]
gb|AJU26135.1| **Mass:** 17388 **Score:** 69 **Matches:** 1(1) **Sequences:** 1(1)
Cox6p [Saccharomyces cerevisiae YJM193]
gb|AJU26390.1| **Mass:** 17388 **Score:** 69 **Matches:** 1(1) **Sequences:** 1(1)
Cox6p [Saccharomyces cerevisiae YJM195]
gb|AJU26640.1| **Mass:** 17388 **Score:** 69 **Matches:** 1(1) **Sequences:** 1(1)
Cox6p [Saccharomyces cerevisiae YJM244]
gb|AJV26838.1| **Mass:** 17388 **Score:** 69 **Matches:** 1(1) **Sequences:** 1(1)
Cox6p [Saccharomyces cerevisiae YJM1083]
gb|AJU26891.1| **Mass:** 17402 **Score:** 69 **Matches:** 1(1) **Sequences:** 1(1)
Cox6p [Saccharomyces cerevisiae YJM248]
gb|AJU27146.1| **Mass:** 17388 **Score:** 69 **Matches:** 1(1) **Sequences:** 1(1)
Cox6p [Saccharomyces cerevisiae YJM270]
gb|AJV27267.1| **Mass:** 17388 **Score:** 69 **Matches:** 1(1) **Sequences:** 1(1)
Cox6p [Saccharomyces cerevisiae YJM1129]
gb|AJU27399.1| **Mass:** 17388 **Score:** 69 **Matches:** 1(1) **Sequences:** 1(1)
Cox6p [Saccharomyces cerevisiae YJM271]
gb|AJV27525.1| **Mass:** 17388 **Score:** 69 **Matches:** 1(1) **Sequences:** 1(1)
Cox6p [Saccharomyces cerevisiae YJM1133]
gb|AJU27653.1| **Mass:** 17388 **Score:** 69 **Matches:** 1(1) **Sequences:** 1(1)
Cox6p [Saccharomyces cerevisiae YJM320]
gb|AJV27782.1| **Mass:** 17388 **Score:** 69 **Matches:** 1(1) **Sequences:** 1(1)
Cox6p [Saccharomyces cerevisiae YJM1190]
gb|AJU27907.1| **Mass:** 17388 **Score:** 69 **Matches:** 1(1) **Sequences:** 1(1)

Cox6p [Saccharomyces cerevisiae YJM326]
gb|AJV28025.1| **Mass:** 17388 **Score:** 69 **Matches:** 1(1) **Sequences:** 1(1)
Cox6p [Saccharomyces cerevisiae YJM1199]
gb|AJU28155.1| **Mass:** 17388 **Score:** 69 **Matches:** 1(1) **Sequences:** 1(1)
Cox6p [Saccharomyces cerevisiae YJM428]
gb|AJV28280.1| **Mass:** 17388 **Score:** 69 **Matches:** 1(1) **Sequences:** 1(1)
Cox6p [Saccharomyces cerevisiae YJM1202]
gb|AJU28412.1| **Mass:** 17388 **Score:** 69 **Matches:** 1(1) **Sequences:** 1(1)
Cox6p [Saccharomyces cerevisiae YJM450]
gb|AJV28536.1| **Mass:** 17388 **Score:** 69 **Matches:** 1(1) **Sequences:** 1(1)
Cox6p [Saccharomyces cerevisiae YJM1208]
gb|AJU28666.1| **Mass:** 17388 **Score:** 69 **Matches:** 1(1) **Sequences:** 1(1)
Cox6p [Saccharomyces cerevisiae YJM451]
gb|AJV28789.1| **Mass:** 17388 **Score:** 69 **Matches:** 1(1) **Sequences:** 1(1)
Cox6p [Saccharomyces cerevisiae YJM1242]
gb|AJU28920.1| **Mass:** 17388 **Score:** 69 **Matches:** 1(1) **Sequences:** 1(1)
Cox6p [Saccharomyces cerevisiae YJM453]
gb|AJV29049.1| **Mass:** 17388 **Score:** 69 **Matches:** 1(1) **Sequences:** 1(1)
Cox6p [Saccharomyces cerevisiae YJM1244]
gb|AJU29181.1| **Mass:** 17388 **Score:** 69 **Matches:** 1(1) **Sequences:** 1(1)
Cox6p [Saccharomyces cerevisiae YJM456]
gb|AJV29299.1| **Mass:** 17303 **Score:** 69 **Matches:** 1(1) **Sequences:** 1(1)
Cox6p [Saccharomyces cerevisiae YJM1248]
gb|AJU29429.1| **Mass:** 17388 **Score:** 69 **Matches:** 1(1) **Sequences:** 1(1)
Cox6p [Saccharomyces cerevisiae YJM470]
gb|AJV29547.1| **Mass:** 17388 **Score:** 69 **Matches:** 1(1) **Sequences:** 1(1)
Cox6p [Saccharomyces cerevisiae YJM1250]
gb|AJU29676.1| **Mass:** 17388 **Score:** 69 **Matches:** 1(1) **Sequences:** 1(1)
Cox6p [Saccharomyces cerevisiae YJM541]
gb|AJV29795.1| **Mass:** 17402 **Score:** 69 **Matches:** 1(1) **Sequences:** 1(1)
Cox6p [Saccharomyces cerevisiae YJM1252]
gb|AJU29926.1| **Mass:** 17388 **Score:** 69 **Matches:** 1(1) **Sequences:** 1(1)
Cox6p [Saccharomyces cerevisiae YJM554]
gb|AJV30051.1| **Mass:** 17388 **Score:** 69 **Matches:** 1(1) **Sequences:** 1(1)
Cox6p [Saccharomyces cerevisiae YJM1273]
gb|AJU30175.1| **Mass:** 17388 **Score:** 69 **Matches:** 1(1) **Sequences:** 1(1)
Cox6p [Saccharomyces cerevisiae YJM555]
gb|AJV30298.1| **Mass:** 17388 **Score:** 69 **Matches:** 1(1) **Sequences:** 1(1)
Cox6p [Saccharomyces cerevisiae YJM1304]
gb|AJU30429.1| **Mass:** 17388 **Score:** 69 **Matches:** 1(1) **Sequences:** 1(1)
Cox6p [Saccharomyces cerevisiae YJM627]
gb|AJV30545.1| **Mass:** 17388 **Score:** 69 **Matches:** 1(1) **Sequences:** 1(1)
Cox6p [Saccharomyces cerevisiae YJM1307]
gb|AJU30683.1| **Mass:** 17388 **Score:** 69 **Matches:** 1(1) **Sequences:** 1(1)
Cox6p [Saccharomyces cerevisiae YJM681]
gb|AJV30806.1| **Mass:** 17388 **Score:** 69 **Matches:** 1(1) **Sequences:** 1(1)
Cox6p [Saccharomyces cerevisiae YJM1311]
gb|AJU30935.1| **Mass:** 17388 **Score:** 69 **Matches:** 1(1) **Sequences:** 1(1)
Cox6p [Saccharomyces cerevisiae YJM682]
gb|AJV31069.1| **Mass:** 17388 **Score:** 69 **Matches:** 1(1) **Sequences:** 1(1)
Cox6p [Saccharomyces cerevisiae YJM1326]
gb|AJU31189.1| **Mass:** 17388 **Score:** 69 **Matches:** 1(1) **Sequences:** 1(1)
Cox6p [Saccharomyces cerevisiae YJM683]
gb|AJV31326.1| **Mass:** 17388 **Score:** 69 **Matches:** 1(1) **Sequences:** 1(1)
Cox6p [Saccharomyces cerevisiae YJM1332]
gb|AJU31442.1| **Mass:** 17388 **Score:** 69 **Matches:** 1(1) **Sequences:** 1(1)
Cox6p [Saccharomyces cerevisiae YJM689]
gb|AJV31586.1| **Mass:** 17388 **Score:** 69 **Matches:** 1(1) **Sequences:** 1(1)
Cox6p [Saccharomyces cerevisiae YJM1336]
gb|AJU31700.1| **Mass:** 17388 **Score:** 69 **Matches:** 1(1) **Sequences:** 1(1)
Cox6p [Saccharomyces cerevisiae YJM693]
gb|AJV31843.1| **Mass:** 17388 **Score:** 69 **Matches:** 1(1) **Sequences:** 1(1)
Cox6p [Saccharomyces cerevisiae YJM1338]
gb|AJV32097.1| **Mass:** 17388 **Score:** 69 **Matches:** 1(1) **Sequences:** 1(1)
Cox6p [Saccharomyces cerevisiae YJM1341]
gb|AJU32127.1| **Mass:** 17388 **Score:** 69 **Matches:** 1(1) **Sequences:** 1(1)
Cox6p [Saccharomyces cerevisiae YJM969]
gb|AJV32327.1| **Mass:** 17388 **Score:** 69 **Matches:** 1(1) **Sequences:** 1(1)
Cox6p [Saccharomyces cerevisiae YJM1342]
gb|AJU32567.1| **Mass:** 17388 **Score:** 69 **Matches:** 1(1) **Sequences:** 1(1)
Cox6p [Saccharomyces cerevisiae YJM972]
gb|AJV32585.1| **Mass:** 17388 **Score:** 69 **Matches:** 1(1) **Sequences:** 1(1)
Cox6p [Saccharomyces cerevisiae YJM1355]
gb|AJU32825.1| **Mass:** 17388 **Score:** 69 **Matches:** 1(1) **Sequences:** 1(1)
Cox6p [Saccharomyces cerevisiae YJM975]
gb|AJU33263.1| **Mass:** 17388 **Score:** 69 **Matches:** 1(1) **Sequences:** 1(1)
Cox6p [Saccharomyces cerevisiae YJM978]
gb|AJU33697.1| **Mass:** 17388 **Score:** 69 **Matches:** 1(1) **Sequences:** 1(1)
Cox6p [Saccharomyces cerevisiae YJM981]
gb|AJU33955.1| **Mass:** 17388 **Score:** 69 **Matches:** 1(1) **Sequences:** 1(1)
Cox6p [Saccharomyces cerevisiae YJM984]
gb|AJU34387.1| **Mass:** 17388 **Score:** 69 **Matches:** 1(1) **Sequences:** 1(1)
Cox6p [Saccharomyces cerevisiae YJM987]
gb|AJU34646.1| **Mass:** 17388 **Score:** 69 **Matches:** 1(1) **Sequences:** 1(1)
Cox6p [Saccharomyces cerevisiae YJM990]
gb|AJU35076.1| **Mass:** 17388 **Score:** 69 **Matches:** 1(1) **Sequences:** 1(1)
Cox6p [Saccharomyces cerevisiae YJM996]
gb|AJP39149.1| **Mass:** 17388 **Score:** 69 **Matches:** 1(1) **Sequences:** 1(1)

Cox6p [Saccharomyces cerevisiae YJM1078]
gb|AAS56604.1| **Mass:** 17388 **Score:** 69 **Matches:** 1(1) **Sequences:** 1(1)
YHR051W [Saccharomyces cerevisiae]
emb|CAG60677.1| **Mass:** 17739 **Score:** 69 **Matches:** 1(1) **Sequences:** 1(1)
unnamed protein product [Candida glabrata]
gb|AAA66900.1| **Mass:** 17388 **Score:** 69 **Matches:** 1(1) **Sequences:** 1(1)
cytochrome c oxidase subunit VI [Saccharomyces cerevisiae]
gb|AAB68899.1| **Mass:** 17388 **Score:** 69 **Matches:** 1(1) **Sequences:** 1(1)
Cox6p: cytochrome c oxidase subunit VI [Saccharomyces cerevisiae]
gb|AHY77750.1| **Mass:** 17388 **Score:** 69 **Matches:** 1(1) **Sequences:** 1(1)
Cox6p [Saccharomyces cerevisiae YJM993]
emb|CAY80338.1| **Mass:** 17388 **Score:** 69 **Matches:** 1(1) **Sequences:** 1(1)
Cox6p [Saccharomyces cerevisiae EC1118]

---

7. gb|AKB01032.1| **Mass:** 17130 **Score:** 64 **Matches:** 1(1) **Sequences:** 1(1)
Cox5ap [Saccharomyces cerevisiae YJM1434]
☐ Check to include this hit in error tolerant search or archive report

Query	Observed	Mr(expt)	Mr(calc)	ppm	Miss	Score	Expect	Rank	Unique	Peptide
41	1711.9300	1710.9227	1710.8154	62.7	0	64	0.00062	1	U	K.QAVWYISYGEWGPR.R

Proteins matching the same set of peptides:
gb|AJT01715.1| **Mass:** 17130 **Score:** 64 **Matches:** 1(1) **Sequences:** 1(1)
Cox5ap [Saccharomyces cerevisiae YJM189]
gb|AJT02084.1| **Mass:** 17130 **Score:** 64 **Matches:** 1(1) **Sequences:** 1(1)
Cox5ap [Saccharomyces cerevisiae YJM193]
gb|AJT02458.1| **Mass:** 17130 **Score:** 64 **Matches:** 1(1) **Sequences:** 1(1)
Cox5ap [Saccharomyces cerevisiae YJM195]
gb|AJT02828.1| **Mass:** 17130 **Score:** 64 **Matches:** 1(1) **Sequences:** 1(1)
Cox5ap [Saccharomyces cerevisiae YJM244]
gb|AJT03209.1| **Mass:** 17130 **Score:** 64 **Matches:** 1(1) **Sequences:** 1(1)
Cox5ap [Saccharomyces cerevisiae YJM248]
gb|AJT03572.1| **Mass:** 17130 **Score:** 64 **Matches:** 1(1) **Sequences:** 1(1)
Cox5ap [Saccharomyces cerevisiae YJM270]
gb|AJT03944.1| **Mass:** 17130 **Score:** 64 **Matches:** 1(1) **Sequences:** 1(1)
Cox5ap [Saccharomyces cerevisiae YJM271]
gb|AJT04320.1| **Mass:** 17130 **Score:** 64 **Matches:** 1(1) **Sequences:** 1(1)
Cox5ap [Saccharomyces cerevisiae YJM320]
gb|AJT04697.1| **Mass:** 17130 **Score:** 64 **Matches:** 1(1) **Sequences:** 1(1)
Cox5ap [Saccharomyces cerevisiae YJM326]
gb|AJT05067.1| **Mass:** 17130 **Score:** 64 **Matches:** 1(1) **Sequences:** 1(1)
Cox5ap [Saccharomyces cerevisiae YJM428]
gb|AJT05445.1| **Mass:** 17130 **Score:** 64 **Matches:** 1(1) **Sequences:** 1(1)
Cox5ap [Saccharomyces cerevisiae YJM450]
gb|AJT05821.1| **Mass:** 17130 **Score:** 64 **Matches:** 1(1) **Sequences:** 1(1)
Cox5ap [Saccharomyces cerevisiae YJM451]
gb|AJT06190.1| **Mass:** 17130 **Score:** 64 **Matches:** 1(1) **Sequences:** 1(1)
Cox5ap [Saccharomyces cerevisiae YJM453]
gb|AJT06566.1| **Mass:** 17130 **Score:** 64 **Matches:** 1(1) **Sequences:** 1(1)
Cox5ap [Saccharomyces cerevisiae YJM456]
gb|AJT06936.1| **Mass:** 17130 **Score:** 64 **Matches:** 1(1) **Sequences:** 1(1)
Cox5ap [Saccharomyces cerevisiae YJM470]
gb|AJT07303.1| **Mass:** 17130 **Score:** 64 **Matches:** 1(1) **Sequences:** 1(1)
Cox5ap [Saccharomyces cerevisiae YJM541]
gb|AJT07673.1| **Mass:** 17130 **Score:** 64 **Matches:** 1(1) **Sequences:** 1(1)
Cox5ap [Saccharomyces cerevisiae YJM554]
gb|AJT08048.1| **Mass:** 17130 **Score:** 64 **Matches:** 1(1) **Sequences:** 1(1)
Cox5ap [Saccharomyces cerevisiae YJM555]
gb|AJT08423.1| **Mass:** 17130 **Score:** 64 **Matches:** 1(1) **Sequences:** 1(1)
Cox5ap [Saccharomyces cerevisiae YJM627]
gb|AJT08792.1| **Mass:** 17130 **Score:** 64 **Matches:** 1(1) **Sequences:** 1(1)
Cox5ap [Saccharomyces cerevisiae YJM681]
gb|AJT09167.1| **Mass:** 17130 **Score:** 64 **Matches:** 1(1) **Sequences:** 1(1)
Cox5ap [Saccharomyces cerevisiae YJM682]
gb|AJT09541.1| **Mass:** 17130 **Score:** 64 **Matches:** 1(1) **Sequences:** 1(1)
Cox5ap [Saccharomyces cerevisiae YJM683]
gb|AJT09911.1| **Mass:** 17130 **Score:** 64 **Matches:** 1(1) **Sequences:** 1(1)
Cox5ap [Saccharomyces cerevisiae YJM689]
gb|AJT10290.1| **Mass:** 17130 **Score:** 64 **Matches:** 1(1) **Sequences:** 1(1)
Cox5ap [Saccharomyces cerevisiae YJM693]
gb|AJT10673.1| **Mass:** 17130 **Score:** 64 **Matches:** 1(1) **Sequences:** 1(1)
Cox5ap [Saccharomyces cerevisiae YJM969]
gb|AJT11052.1| **Mass:** 17130 **Score:** 64 **Matches:** 1(1) **Sequences:** 1(1)
Cox5ap [Saccharomyces cerevisiae YJM972]
gb|AJT11434.1| **Mass:** 17130 **Score:** 64 **Matches:** 1(1) **Sequences:** 1(1)
Cox5ap [Saccharomyces cerevisiae YJM975]
gb|AJT11814.1| **Mass:** 17130 **Score:** 64 **Matches:** 1(1) **Sequences:** 1(1)
Cox5ap [Saccharomyces cerevisiae YJM978]
gb|AJT12198.1| **Mass:** 17130 **Score:** 64 **Matches:** 1(1) **Sequences:** 1(1)
Cox5ap [Saccharomyces cerevisiae YJM981]
gb|AJT12579.1| **Mass:** 17130 **Score:** 64 **Matches:** 1(1) **Sequences:** 1(1)
Cox5ap [Saccharomyces cerevisiae YJM984]
gb|AJT12961.1| **Mass:** 17130 **Score:** 64 **Matches:** 1(1) **Sequences:** 1(1)
Cox5ap [Saccharomyces cerevisiae YJM987]
gb|AJT13346.1| **Mass:** 17130 **Score:** 64 **Matches:** 1(1) **Sequences:** 1(1)
Cox5ap [Saccharomyces cerevisiae YJM990]
gb|AJT13727.1| **Mass:** 17130 **Score:** 64 **Matches:** 1(1) **Sequences:** 1(1)

Cox5ap [Saccharomyces cerevisiae YJM996]
gb|AJT14108.1| **Mass:** 17130 **Score:** 64 **Matches:** 1(1) **Sequences:** 1(1)
Cox5ap [Saccharomyces cerevisiae YJM1083]
gb|AJT14479.1| **Mass:** 17130 **Score:** 64 **Matches:** 1(1) **Sequences:** 1(1)
Cox5ap [Saccharomyces cerevisiae YJM1129]
gb|AJT14848.1| **Mass:** 17130 **Score:** 64 **Matches:** 1(1) **Sequences:** 1(1)
Cox5ap [Saccharomyces cerevisiae YJM1133]
gb|AJT15223.1| **Mass:** 17130 **Score:** 64 **Matches:** 1(1) **Sequences:** 1(1)
Cox5ap [Saccharomyces cerevisiae YJM1190]
gb|AJT15596.1| **Mass:** 17130 **Score:** 64 **Matches:** 1(1) **Sequences:** 1(1)
Cox5ap [Saccharomyces cerevisiae YJM1199]
gb|AJT15969.1| **Mass:** 17130 **Score:** 64 **Matches:** 1(1) **Sequences:** 1(1)
Cox5ap [Saccharomyces cerevisiae YJM1202]
gb|AJT16340.1| **Mass:** 17130 **Score:** 64 **Matches:** 1(1) **Sequences:** 1(1)
Cox5ap [Saccharomyces cerevisiae YJM1208]
gb|AJT16715.1| **Mass:** 17130 **Score:** 64 **Matches:** 1(1) **Sequences:** 1(1)
Cox5ap [Saccharomyces cerevisiae YJM1242]
gb|AJT17093.1| **Mass:** 17130 **Score:** 64 **Matches:** 1(1) **Sequences:** 1(1)
Cox5ap [Saccharomyces cerevisiae YJM1244]
gb|AJT17466.1| **Mass:** 17130 **Score:** 64 **Matches:** 1(1) **Sequences:** 1(1)
Cox5ap [Saccharomyces cerevisiae YJM1248]
gb|AJT17836.1| **Mass:** 17130 **Score:** 64 **Matches:** 1(1) **Sequences:** 1(1)
Cox5ap [Saccharomyces cerevisiae YJM1250]
gb|AJT18219.1| **Mass:** 17130 **Score:** 64 **Matches:** 1(1) **Sequences:** 1(1)
Cox5ap [Saccharomyces cerevisiae YJM1252]
gb|AJT18591.1| **Mass:** 17130 **Score:** 64 **Matches:** 1(1) **Sequences:** 1(1)
Cox5ap [Saccharomyces cerevisiae YJM1273]
gb|AJT18960.1| **Mass:** 17130 **Score:** 64 **Matches:** 1(1) **Sequences:** 1(1)
Cox5ap [Saccharomyces cerevisiae YJM1304]
gb|AJT19329.1| **Mass:** 17130 **Score:** 64 **Matches:** 1(1) **Sequences:** 1(1)
Cox5ap [Saccharomyces cerevisiae YJM1307]
gb|AJT19697.1| **Mass:** 17130 **Score:** 64 **Matches:** 1(1) **Sequences:** 1(1)
Cox5ap [Saccharomyces cerevisiae YJM1311]
gb|AJT20065.1| **Mass:** 17130 **Score:** 64 **Matches:** 1(1) **Sequences:** 1(1)
Cox5ap [Saccharomyces cerevisiae YJM1326]
gb|AJT20442.1| **Mass:** 17130 **Score:** 64 **Matches:** 1(1) **Sequences:** 1(1)
Cox5ap [Saccharomyces cerevisiae YJM1332]
gb|AJT20815.1| **Mass:** 17130 **Score:** 64 **Matches:** 1(1) **Sequences:** 1(1)
Cox5ap [Saccharomyces cerevisiae YJM1336]
gb|AJT21189.1| **Mass:** 17130 **Score:** 64 **Matches:** 1(1) **Sequences:** 1(1)
Cox5ap [Saccharomyces cerevisiae YJM1338]
gb|AJT21562.1| **Mass:** 17130 **Score:** 64 **Matches:** 1(1) **Sequences:** 1(1)
Cox5ap [Saccharomyces cerevisiae YJM1341]
gb|AJT21934.1| **Mass:** 17130 **Score:** 64 **Matches:** 1(1) **Sequences:** 1(1)
Cox5ap [Saccharomyces cerevisiae YJM1342]
gb|AJT22261.1| **Mass:** 17130 **Score:** 64 **Matches:** 1(1) **Sequences:** 1(1)
Cox5ap [Saccharomyces cerevisiae YJM1355]
gb|AJT22637.1| **Mass:** 17130 **Score:** 64 **Matches:** 1(1) **Sequences:** 1(1)
Cox5ap [Saccharomyces cerevisiae YJM1356]
gb|AJT23015.1| **Mass:** 17130 **Score:** 64 **Matches:** 1(1) **Sequences:** 1(1)
Cox5ap [Saccharomyces cerevisiae YJM1381]
gb|AJT23391.1| **Mass:** 17130 **Score:** 64 **Matches:** 1(1) **Sequences:** 1(1)
Cox5ap [Saccharomyces cerevisiae YJM1383]
gb|AJT23767.1| **Mass:** 17130 **Score:** 64 **Matches:** 1(1) **Sequences:** 1(1)
Cox5ap [Saccharomyces cerevisiae YJM1385]
gb|AJT24142.1| **Mass:** 17130 **Score:** 64 **Matches:** 1(1) **Sequences:** 1(1)
Cox5ap [Saccharomyces cerevisiae YJM1386]
gb|AJT24517.1| **Mass:** 17130 **Score:** 64 **Matches:** 1(1) **Sequences:** 1(1)
Cox5ap [Saccharomyces cerevisiae YJM1387]
gb|AJT24884.1| **Mass:** 17130 **Score:** 64 **Matches:** 1(1) **Sequences:** 1(1)
Cox5ap [Saccharomyces cerevisiae YJM1388]
gb|AJT25247.1| **Mass:** 17130 **Score:** 64 **Matches:** 1(1) **Sequences:** 1(1)
Cox5ap [Saccharomyces cerevisiae YJM1389]
gb|AJT25611.1| **Mass:** 17130 **Score:** 64 **Matches:** 1(1) **Sequences:** 1(1)
Cox5ap [Saccharomyces cerevisiae YJM1399]
gb|AJT25980.1| **Mass:** 17130 **Score:** 64 **Matches:** 1(1) **Sequences:** 1(1)
Cox5ap [Saccharomyces cerevisiae YJM1400]
gb|AJT26343.1| **Mass:** 17130 **Score:** 64 **Matches:** 1(1) **Sequences:** 1(1)
Cox5ap [Saccharomyces cerevisiae YJM1401]
emb|CAA26403.1| **Mass:** 17130 **Score:** 64 **Matches:** 1(1) **Sequences:** 1(1)
cytochrome oxidase subunit V [Saccharomyces cerevisiae]
gb|AJT26712.1| **Mass:** 17130 **Score:** 64 **Matches:** 1(1) **Sequences:** 1(1)
Cox5ap [Saccharomyces cerevisiae YJM1402]
gb|AJT27083.1| **Mass:** 17130 **Score:** 64 **Matches:** 1(1) **Sequences:** 1(1)
Cox5ap [Saccharomyces cerevisiae YJM1415]
gb|AJT27460.1| **Mass:** 17130 **Score:** 64 **Matches:** 1(1) **Sequences:** 1(1)
Cox5ap [Saccharomyces cerevisiae YJM1417]
gb|AJT27830.1| **Mass:** 17130 **Score:** 64 **Matches:** 1(1) **Sequences:** 1(1)
Cox5ap [Saccharomyces cerevisiae YJM1418]
gb|AJT28203.1| **Mass:** 17130 **Score:** 64 **Matches:** 1(1) **Sequences:** 1(1)
Cox5ap [Saccharomyces cerevisiae YJM1419]
gb|AJT28578.1| **Mass:** 17129 **Score:** 64 **Matches:** 1(1) **Sequences:** 1(1)
Cox5ap [Saccharomyces cerevisiae YJM1433]
gb|AJT28955.1| **Mass:** 17130 **Score:** 64 **Matches:** 1(1) **Sequences:** 1(1)
Cox5ap [Saccharomyces cerevisiae YJM1439]
gb|AJT29311.1| **Mass:** 17130 **Score:** 64 **Matches:** 1(1) **Sequences:** 1(1)
Cox5ap [Saccharomyces cerevisiae YJM1443]
gb|AJT29664.1| **Mass:** 17130 **Score:** 64 **Matches:** 1(1) **Sequences:** 1(1)

Cox5ap [Saccharomyces cerevisiae YJM1444]
gb|AJT30029.1| **Mass:** 17130 **Score:** 64 **Matches:** 1(1) **Sequences:** 1(1)
Cox5ap [Saccharomyces cerevisiae YJM1447]
gb|AJT30387.1| **Mass:** 17130 **Score:** 64 **Matches:** 1(1) **Sequences:** 1(1)
Cox5ap [Saccharomyces cerevisiae YJM1450]
gb|AJT30757.1| **Mass:** 17130 **Score:** 64 **Matches:** 1(1) **Sequences:** 1(1)
Cox5ap [Saccharomyces cerevisiae YJM1460]
gb|AJT31119.1| **Mass:** 17130 **Score:** 64 **Matches:** 1(1) **Sequences:** 1(1)
Cox5ap [Saccharomyces cerevisiae YJM1463]
gb|AJT31491.1| **Mass:** 17130 **Score:** 64 **Matches:** 1(1) **Sequences:** 1(1)
Cox5ap [Saccharomyces cerevisiae YJM1477]
gb|AJT31866.1| **Mass:** 17129 **Score:** 64 **Matches:** 1(1) **Sequences:** 1(1)
Cox5ap [Saccharomyces cerevisiae YJM1478]
gb|AJT32238.1| **Mass:** 17130 **Score:** 64 **Matches:** 1(1) **Sequences:** 1(1)
Cox5ap [Saccharomyces cerevisiae YJM1479]
gb|AJT32608.1| **Mass:** 17130 **Score:** 64 **Matches:** 1(1) **Sequences:** 1(1)
Cox5ap [Saccharomyces cerevisiae YJM1526]
gb|AJT32982.1| **Mass:** 17130 **Score:** 64 **Matches:** 1(1) **Sequences:** 1(1)
Cox5ap [Saccharomyces cerevisiae YJM1527]
gb|AJT33360.1| **Mass:** 17129 **Score:** 64 **Matches:** 1(1) **Sequences:** 1(1)
Cox5ap [Saccharomyces cerevisiae YJM1549]
gb|AJT33733.1| **Mass:** 17130 **Score:** 64 **Matches:** 1(1) **Sequences:** 1(1)
Cox5ap [Saccharomyces cerevisiae YJM1573]
gb|AJT34103.1| **Mass:** 17130 **Score:** 64 **Matches:** 1(1) **Sequences:** 1(1)
Cox5ap [Saccharomyces cerevisiae YJM1574]
gb|AJT34475.1| **Mass:** 17130 **Score:** 64 **Matches:** 1(1) **Sequences:** 1(1)
Cox5ap [Saccharomyces cerevisiae YJM1592]
gb|AAA34518.1| **Mass:** 17130 **Score:** 64 **Matches:** 1(1) **Sequences:** 1(1)
cytochrome oxidase subunit 5 [Saccharomyces cerevisiae]
gb|AAA34519.1| **Mass:** 7891 **Score:** 64 **Matches:** 1(1) **Sequences:** 1(1)
cytochrome c oxidase subunit Va, partial [Saccharomyces cerevisiae]
gb|AAA34520.1| **Mass:** 17130 **Score:** 64 **Matches:** 1(1) **Sequences:** 1(1)
mitochondrial cytochrome c oxidase subunit Va [Saccharomyces cerevisiae]
gb|AJT34841.1| **Mass:** 17130 **Score:** 64 **Matches:** 1(1) **Sequences:** 1(1)
Cox5ap [Saccharomyces cerevisiae YJM1615]
gb|AJP41277.1| **Mass:** 17130 **Score:** 64 **Matches:** 1(1) **Sequences:** 1(1)
Cox5ap [Saccharomyces cerevisiae YJM1078]
gb|AAS56457.1| **Mass:** 17130 **Score:** 64 **Matches:** 1(1) **Sequences:** 1(1)
YNL052W [Saccharomyces cerevisiae]
emb|CEP63121.1| **Mass:** 16917 **Score:** 64 **Matches:** 1(1) **Sequences:** 1(1)
LALA0S07e02850g1_1 [Lachancea lanzarotensis]
gb|AHY77040.1| **Mass:** 17130 **Score:** 64 **Matches:** 1(1) **Sequences:** 1(1)
Cox5ap [Saccharomyces cerevisiae YJM993]
emb|CAY82549.1| **Mass:** 17130 **Score:** 64 **Matches:** 1(1) **Sequences:** 1(1)
Cox5ap [Saccharomyces cerevisiae EC1118]
emb|CAA95921.1| **Mass:** 17130 **Score:** 64 **Matches:** 1(1) **Sequences:** 1(1)
COX5A [Saccharomyces cerevisiae]
gb|AAA99660.1| **Mass:** 17130 **Score:** 64 **Matches:** 1(1) **Sequences:** 1(1)
cytochrome c oxidase polypeptide VA precursor CoxA [Saccharomyces cerevisiae]

---

8. pdb|1EZV|E **Mass:** 20314 **Score:** 49 **Matches:** 1(1) **Sequences:** 1(1)
Chain E, Structure Of The Yeast Cytochrome Bc1 Complex Co- Crystallized With An Antibody Fv-Fragment
☐ Check to include this hit in error tolerant search or archive report

Query	Observed	Mr(expt)	Mr(calc)	ppm	Miss	Score	Expect	Rank	Unique	Peptide
3	1130.7178	1129.7105	1129.6396	62.8	0	49	0.016	1	U	K.WQGKPVFIR.H

Proteins matching the same set of peptides:
pdb|1KB9|E **Mass:** 20314 **Score:** 49 **Matches:** 1(1) **Sequences:** 1(1)
Chain E, Yeast Cytochrome Bc1 Complex
pdb|1KYO|E **Mass:** 20314 **Score:** 49 **Matches:** 1(1) **Sequences:** 1(1)
Chain E, Yeast Cytochrome Bc1 Complex With Bound Substrate Cytochrome C
pdb|1KYO|P **Mass:** 20314 **Score:** 49 **Matches:** 1(1) **Sequences:** 1(1)
Chain P, Yeast Cytochrome Bc1 Complex With Bound Substrate Cytochrome C
pdb|1P84|E **Mass:** 20314 **Score:** 49 **Matches:** 1(1) **Sequences:** 1(1)
Chain E, Hdbt Inhibited Yeast Cytochrome Bc1 Complex
pdb|2IBZ|E **Mass:** 20314 **Score:** 49 **Matches:** 1(1) **Sequences:** 1(1)
Chain E, Yeast Cytochrome Bc1 Complex With Stigmatellin
pdb|3CX5|E **Mass:** 20314 **Score:** 49 **Matches:** 1(1) **Sequences:** 1(1)
Chain E, Structure Of Complex Iii With Bound Cytochrome C In Reduced State And Definition Of A Minimal Core Interface For Electron Trans
pdb|3CX5|P **Mass:** 20314 **Score:** 49 **Matches:** 1(1) **Sequences:** 1(1)
Chain P, Structure Of Complex Iii With Bound Cytochrome C In Reduced State And Definition Of A Minimal Core Interface For Electron Trans
pdb|3CXH|E **Mass:** 20314 **Score:** 49 **Matches:** 1(1) **Sequences:** 1(1)
Chain E, Structure Of Yeast Complex Iii With Isoform-2 Cytochrome C Bound And Definition Of A Minimal Core Interface For Electron Transf
pdb|3CXH|P **Mass:** 20314 **Score:** 49 **Matches:** 1(1) **Sequences:** 1(1)
Chain P, Structure Of Yeast Complex Iii With Isoform-2 Cytochrome C Bound And Definition Of A Minimal Core Interface For Electron Transf
pdb|4PD4|E **Mass:** 20314 **Score:** 49 **Matches:** 1(1) **Sequences:** 1(1)
Chain E, Structural Analysis Of Atovaquone-inhibited Cytochrome Bc1 Complex Reveals The Molecular Basis Of Antimalarial Drug Action
emb|CAH00060.1| **Mass:** 23189 **Score:** 49 **Matches:** 1(1) **Sequences:** 1(1)
KLLA0E22573p [Kluyveromyces lactis]
emb|CCD25819.2| **Mass:** 24572 **Score:** 49 **Matches:** 1(1) **Sequences:** 1(1)
hypothetical protein NDAI_0G00430 [Naumovozyma dairenensis CBS 421]
gb|AHL27309.1| **Mass:** 20482 **Score:** 49 **Matches:** 1(1) **Sequences:** 1(1)
ubiquinol-cytochrome-c reductase, partial [Saccharomyces cerevisiae x Saccharomyces eubayanus x Saccharomyces uvarum]
emb|CAR30617.1| **Mass:** 23461 **Score:** 49 **Matches:** 1(1) **Sequences:** 1(1)
KLTH0H14344p [Lachancea thermotolerans CBS 6340]
emb|CAR30823.1| **Mass:** 23470 **Score:** 49 **Matches:** 1(1) **Sequences:** 1(1)

ZYRO0E03146p [Zygosaccharomyces rouxii]
gb|AJV32784.1| **Mass:** 23607 **Score:** 49 **Matches:** 1(1) **Sequences:** 1(1)
Rip1p [Saccharomyces cerevisiae YJM189]
gb|AJV33039.1| **Mass:** 23621 **Score:** 49 **Matches:** 1(1) **Sequences:** 1(1)
Rip1p [Saccharomyces cerevisiae YJM193]
gb|AJV33296.1| **Mass:** 23621 **Score:** 49 **Matches:** 1(1) **Sequences:** 1(1)
Rip1p [Saccharomyces cerevisiae YJM195]
gb|AJV33550.1| **Mass:** 23635 **Score:** 49 **Matches:** 1(1) **Sequences:** 1(1)
Rip1p [Saccharomyces cerevisiae YJM244]
gb|AJV33806.1| **Mass:** 23621 **Score:** 49 **Matches:** 1(1) **Sequences:** 1(1)
Rip1p [Saccharomyces cerevisiae YJM248]
gb|AJV34061.1| **Mass:** 23651 **Score:** 49 **Matches:** 1(1) **Sequences:** 1(1)
Rip1p [Saccharomyces cerevisiae YJM270]
gb|AJV34320.1| **Mass:** 23621 **Score:** 49 **Matches:** 1(1) **Sequences:** 1(1)
Rip1p [Saccharomyces cerevisiae YJM271]
gb|AJV34573.1| **Mass:** 23621 **Score:** 49 **Matches:** 1(1) **Sequences:** 1(1)
Rip1p [Saccharomyces cerevisiae YJM320]
gb|AJV34782.1| **Mass:** 23621 **Score:** 49 **Matches:** 1(1) **Sequences:** 1(1)
Rip1p [Saccharomyces cerevisiae YJM326]
gb|AAA34980.1| **Mass:** 23635 **Score:** 49 **Matches:** 1(1) **Sequences:** 1(1)
Rieske iron-sulfur protein [Saccharomyces cerevisiae]
gb|AAA34981.1| **Mass:** 23635 **Score:** 49 **Matches:** 1(1) **Sequences:** 1(1)
Rieske iron-sulfur protein [Saccharomyces cerevisiae]
gb|AJV35036.1| **Mass:** 23621 **Score:** 49 **Matches:** 1(1) **Sequences:** 1(1)
Rip1p [Saccharomyces cerevisiae YJM428]
gb|AJV35291.1| **Mass:** 23621 **Score:** 49 **Matches:** 1(1) **Sequences:** 1(1)
Rip1p [Saccharomyces cerevisiae YJM450]
gb|AJV35548.1| **Mass:** 23621 **Score:** 49 **Matches:** 1(1) **Sequences:** 1(1)
Rip1p [Saccharomyces cerevisiae YJM451]
gb|AJV35800.1| **Mass:** 23607 **Score:** 49 **Matches:** 1(1) **Sequences:** 1(1)
Rip1p [Saccharomyces cerevisiae YJM453]
gb|AJV36062.1| **Mass:** 23607 **Score:** 49 **Matches:** 1(1) **Sequences:** 1(1)
Rip1p [Saccharomyces cerevisiae YJM456]
gb|AJV36317.1| **Mass:** 23621 **Score:** 49 **Matches:** 1(1) **Sequences:** 1(1)
Rip1p [Saccharomyces cerevisiae YJM470]
gb|AJV36570.1| **Mass:** 23621 **Score:** 49 **Matches:** 1(1) **Sequences:** 1(1)
Rip1p [Saccharomyces cerevisiae YJM541]
gb|AJV36824.1| **Mass:** 23621 **Score:** 49 **Matches:** 1(1) **Sequences:** 1(1)
Rip1p [Saccharomyces cerevisiae YJM554]
gb|AJV37076.1| **Mass:** 23607 **Score:** 49 **Matches:** 1(1) **Sequences:** 1(1)
Rip1p [Saccharomyces cerevisiae YJM555]
gb|AJV37334.1| **Mass:** 23621 **Score:** 49 **Matches:** 1(1) **Sequences:** 1(1)
Rip1p [Saccharomyces cerevisiae YJM627]
gb|AJV37588.1| **Mass:** 23607 **Score:** 49 **Matches:** 1(1) **Sequences:** 1(1)
Rip1p [Saccharomyces cerevisiae YJM681]
gb|AJV37841.1| **Mass:** 23621 **Score:** 49 **Matches:** 1(1) **Sequences:** 1(1)
Rip1p [Saccharomyces cerevisiae YJM682]
gb|AJV38093.1| **Mass:** 23621 **Score:** 49 **Matches:** 1(1) **Sequences:** 1(1)
Rip1p [Saccharomyces cerevisiae YJM683]
gb|AJP38242.1| **Mass:** 23621 **Score:** 49 **Matches:** 1(1) **Sequences:** 1(1)
Rip1p [Saccharomyces cerevisiae YJM1078]
gb|AJV38350.1| **Mass:** 23621 **Score:** 49 **Matches:** 1(1) **Sequences:** 1(1)
Rip1p [Saccharomyces cerevisiae YJM689]
emb|CDR38619.1| **Mass:** 23465 **Score:** 49 **Matches:** 1(1) **Sequences:** 1(1)
CYFA0S02e03752g1_1 [Cyberlindnera fabianii]
gb|AJU39932.1| **Mass:** 23621 **Score:** 49 **Matches:** 1(1) **Sequences:** 1(1)
Rip1p [Saccharomyces cerevisiae YJM693]
gb|AJU40186.1| **Mass:** 23621 **Score:** 49 **Matches:** 1(1) **Sequences:** 1(1)
Rip1p [Saccharomyces cerevisiae YJM969]
gb|AJU40441.1| **Mass:** 23621 **Score:** 49 **Matches:** 1(1) **Sequences:** 1(1)
Rip1p [Saccharomyces cerevisiae YJM972]
gb|AJU40701.1| **Mass:** 23621 **Score:** 49 **Matches:** 1(1) **Sequences:** 1(1)
Rip1p [Saccharomyces cerevisiae YJM975]
gb|AJU40959.1| **Mass:** 23621 **Score:** 49 **Matches:** 1(1) **Sequences:** 1(1)
Rip1p [Saccharomyces cerevisiae YJM978]
gb|AJU41186.1| **Mass:** 23621 **Score:** 49 **Matches:** 1(1) **Sequences:** 1(1)
Rip1p [Saccharomyces cerevisiae YJM981]
gb|AJU41440.1| **Mass:** 23621 **Score:** 49 **Matches:** 1(1) **Sequences:** 1(1)
Rip1p [Saccharomyces cerevisiae YJM984]
gb|AJU41695.1| **Mass:** 23621 **Score:** 49 **Matches:** 1(1) **Sequences:** 1(1)
Rip1p [Saccharomyces cerevisiae YJM987]
gb|AJU41953.1| **Mass:** 23621 **Score:** 49 **Matches:** 1(1) **Sequences:** 1(1)
Rip1p [Saccharomyces cerevisiae YJM990]
gb|AJU42211.1| **Mass:** 23621 **Score:** 49 **Matches:** 1(1) **Sequences:** 1(1)
Rip1p [Saccharomyces cerevisiae YJM996]
gb|AJU42465.1| **Mass:** 23621 **Score:** 49 **Matches:** 1(1) **Sequences:** 1(1)
Rip1p [Saccharomyces cerevisiae YJM1083]
gb|AJU42720.1| **Mass:** 23621 **Score:** 49 **Matches:** 1(1) **Sequences:** 1(1)
Rip1p [Saccharomyces cerevisiae YJM1129]
gb|AJU42980.1| **Mass:** 23621 **Score:** 49 **Matches:** 1(1) **Sequences:** 1(1)
Rip1p [Saccharomyces cerevisiae YJM1133]
gb|AJU43235.1| **Mass:** 23621 **Score:** 49 **Matches:** 1(1) **Sequences:** 1(1)
Rip1p [Saccharomyces cerevisiae YJM1190]
gb|AJU43491.1| **Mass:** 23621 **Score:** 49 **Matches:** 1(1) **Sequences:** 1(1)
Rip1p [Saccharomyces cerevisiae YJM1199]
gb|AJU43745.1| **Mass:** 23621 **Score:** 49 **Matches:** 1(1) **Sequences:** 1(1)
Rip1p [Saccharomyces cerevisiae YJM1202]
gb|AJU43985.1| **Mass:** 23621 **Score:** 49 **Matches:** 1(1) **Sequences:** 1(1)

Rip1p [Saccharomyces cerevisiae YJM1208]
gb|AJU44242.1| **Mass:** 23621 **Score:** 49 **Matches:** 1(1) **Sequences:** 1(1)
Rip1p [Saccharomyces cerevisiae YJM1242]
gb|AJU44501.1| **Mass:** 23607 **Score:** 49 **Matches:** 1(1) **Sequences:** 1(1)
Rip1p [Saccharomyces cerevisiae YJM1244]
gb|AJU44759.1| **Mass:** 23621 **Score:** 49 **Matches:** 1(1) **Sequences:** 1(1)
Rip1p [Saccharomyces cerevisiae YJM1248]
gb|AJU45014.1| **Mass:** 23621 **Score:** 49 **Matches:** 1(1) **Sequences:** 1(1)
Rip1p [Saccharomyces cerevisiae YJM1250]
gb|AJU45272.1| **Mass:** 23621 **Score:** 49 **Matches:** 1(1) **Sequences:** 1(1)
Rip1p [Saccharomyces cerevisiae YJM1615]
gb|AJU45527.1| **Mass:** 23621 **Score:** 49 **Matches:** 1(1) **Sequences:** 1(1)
Rip1p [Saccharomyces cerevisiae YJM1252]
gb|AJU45779.1| **Mass:** 23621 **Score:** 49 **Matches:** 1(1) **Sequences:** 1(1)
Rip1p [Saccharomyces cerevisiae YJM1273]
gb|AJU46038.1| **Mass:** 23621 **Score:** 49 **Matches:** 1(1) **Sequences:** 1(1)
Rip1p [Saccharomyces cerevisiae YJM1304]
gb|AJU46284.1| **Mass:** 23651 **Score:** 49 **Matches:** 1(1) **Sequences:** 1(1)
Rip1p [Saccharomyces cerevisiae YJM1307]
gb|AJU46540.1| **Mass:** 23621 **Score:** 49 **Matches:** 1(1) **Sequences:** 1(1)
Rip1p [Saccharomyces cerevisiae YJM1311]
gb|AJU46791.1| **Mass:** 23621 **Score:** 49 **Matches:** 1(1) **Sequences:** 1(1)
Rip1p [Saccharomyces cerevisiae YJM1326]
gb|AJU47051.1| **Mass:** 23635 **Score:** 49 **Matches:** 1(1) **Sequences:** 1(1)
Rip1p [Saccharomyces cerevisiae YJM1332]
gb|AJU47296.1| **Mass:** 23621 **Score:** 49 **Matches:** 1(1) **Sequences:** 1(1)
Rip1p [Saccharomyces cerevisiae YJM1336]
gb|AJU47554.1| **Mass:** 23621 **Score:** 49 **Matches:** 1(1) **Sequences:** 1(1)
Rip1p [Saccharomyces cerevisiae YJM1338]
gb|AJU47813.1| **Mass:** 23621 **Score:** 49 **Matches:** 1(1) **Sequences:** 1(1)
Rip1p [Saccharomyces cerevisiae YJM1341]
gb|AJU48070.1| **Mass:** 23621 **Score:** 49 **Matches:** 1(1) **Sequences:** 1(1)
Rip1p [Saccharomyces cerevisiae YJM1342]
gb|AJU48328.1| **Mass:** 23621 **Score:** 49 **Matches:** 1(1) **Sequences:** 1(1)
Rip1p [Saccharomyces cerevisiae YJM1355]
gb|AJU48579.1| **Mass:** 23621 **Score:** 49 **Matches:** 1(1) **Sequences:** 1(1)
Rip1p [Saccharomyces cerevisiae YJM1356]
gb|AJU48838.1| **Mass:** 23621 **Score:** 49 **Matches:** 1(1) **Sequences:** 1(1)
Rip1p [Saccharomyces cerevisiae YJM1381]
gb|AJU49095.1| **Mass:** 23635 **Score:** 49 **Matches:** 1(1) **Sequences:** 1(1)
Rip1p [Saccharomyces cerevisiae YJM1383]
gb|AJU49353.1| **Mass:** 23621 **Score:** 49 **Matches:** 1(1) **Sequences:** 1(1)
Rip1p [Saccharomyces cerevisiae YJM1385]
gb|AAC49359.1| **Mass:** 25038 **Score:** 49 **Matches:** 1(1) **Sequences:** 1(1)
Rieske iron-sulfur protein [Schizosaccharomyces pombe]
gb|AJU49603.1| **Mass:** 23621 **Score:** 49 **Matches:** 1(1) **Sequences:** 1(1)
Rip1p [Saccharomyces cerevisiae YJM1386]
gb|AJU49856.1| **Mass:** 23621 **Score:** 49 **Matches:** 1(1) **Sequences:** 1(1)
Rip1p [Saccharomyces cerevisiae YJM1387]
gb|AJU50109.1| **Mass:** 23621 **Score:** 49 **Matches:** 1(1) **Sequences:** 1(1)
Rip1p [Saccharomyces cerevisiae YJM1388]
gb|AJU50361.1| **Mass:** 23621 **Score:** 49 **Matches:** 1(1) **Sequences:** 1(1)
Rip1p [Saccharomyces cerevisiae YJM1389]
gb|AJU50611.1| **Mass:** 23621 **Score:** 49 **Matches:** 1(1) **Sequences:** 1(1)
Rip1p [Saccharomyces cerevisiae YJM1399]
gb|AJU50864.1| **Mass:** 23651 **Score:** 49 **Matches:** 1(1) **Sequences:** 1(1)
Rip1p [Saccharomyces cerevisiae YJM1400]
gb|AJU51122.1| **Mass:** 23621 **Score:** 49 **Matches:** 1(1) **Sequences:** 1(1)
Rip1p [Saccharomyces cerevisiae YJM1401]
gb|AJU51374.1| **Mass:** 23621 **Score:** 49 **Matches:** 1(1) **Sequences:** 1(1)
Rip1p [Saccharomyces cerevisiae YJM1402]
gb|AJU51632.1| **Mass:** 23621 **Score:** 49 **Matches:** 1(1) **Sequences:** 1(1)
Rip1p [Saccharomyces cerevisiae YJM1415]
gb|AJU51889.1| **Mass:** 23607 **Score:** 49 **Matches:** 1(1) **Sequences:** 1(1)
Rip1p [Saccharomyces cerevisiae YJM1417]
gb|AJU52143.1| **Mass:** 23621 **Score:** 49 **Matches:** 1(1) **Sequences:** 1(1)
Rip1p [Saccharomyces cerevisiae YJM1418]
gb|AJU52399.1| **Mass:** 23621 **Score:** 49 **Matches:** 1(1) **Sequences:** 1(1)
Rip1p [Saccharomyces cerevisiae YJM1419]
gb|AAS52618.1| **Mass:** 23119 **Score:** 49 **Matches:** 1(1) **Sequences:** 1(1)
AEL067Wp [Eremothecium gossypii ATCC 10895]
gb|AJU52655.1| **Mass:** 23621 **Score:** 49 **Matches:** 1(1) **Sequences:** 1(1)
Rip1p [Saccharomyces cerevisiae YJM1433]
gb|AJU52912.1| **Mass:** 23621 **Score:** 49 **Matches:** 1(1) **Sequences:** 1(1)
Rip1p [Saccharomyces cerevisiae YJM1434]
gb|AJU53168.1| **Mass:** 23621 **Score:** 49 **Matches:** 1(1) **Sequences:** 1(1)
Rip1p [Saccharomyces cerevisiae YJM1439]
gb|AJU53423.1| **Mass:** 23635 **Score:** 49 **Matches:** 1(1) **Sequences:** 1(1)
Rip1p [Saccharomyces cerevisiae YJM1443]
gb|AJU53676.1| **Mass:** 23621 **Score:** 49 **Matches:** 1(1) **Sequences:** 1(1)
Rip1p [Saccharomyces cerevisiae YJM1444]
gb|AJU53926.1| **Mass:** 23621 **Score:** 49 **Matches:** 1(1) **Sequences:** 1(1)
Rip1p [Saccharomyces cerevisiae YJM1447]
gb|AJU54171.1| **Mass:** 23621 **Score:** 49 **Matches:** 1(1) **Sequences:** 1(1)
Rip1p [Saccharomyces cerevisiae YJM1450]
gb|AJU54412.1| **Mass:** 23621 **Score:** 49 **Matches:** 1(1) **Sequences:** 1(1)
Rip1p [Saccharomyces cerevisiae YJM1460]
gb|AJU54667.1| **Mass:** 23621 **Score:** 49 **Matches:** 1(1) **Sequences:** 1(1)

Rip1p [Saccharomyces cerevisiae YJM1463]
gb|AJU54922.1| **Mass:** 23621 **Score:** 49 **Matches:** 1(1) **Sequences:** 1(1)
Rip1p [Saccharomyces cerevisiae YJM1477]
gb|AJU55182.1| **Mass:** 23607 **Score:** 49 **Matches:** 1(1) **Sequences:** 1(1)
Rip1p [Saccharomyces cerevisiae YJM1478]
gb|AJU55439.1| **Mass:** 23651 **Score:** 49 **Matches:** 1(1) **Sequences:** 1(1)
Rip1p [Saccharomyces cerevisiae YJM1479]
gb|AJU55696.1| **Mass:** 23621 **Score:** 49 **Matches:** 1(1) **Sequences:** 1(1)
Rip1p [Saccharomyces cerevisiae YJM1526]
gb|AJU55955.1| **Mass:** 23621 **Score:** 49 **Matches:** 1(1) **Sequences:** 1(1)
Rip1p [Saccharomyces cerevisiae YJM1527]
gb|AJU56212.1| **Mass:** 23621 **Score:** 49 **Matches:** 1(1) **Sequences:** 1(1)
Rip1p [Saccharomyces cerevisiae YJM1549]
gb|AJU56468.1| **Mass:** 23621 **Score:** 49 **Matches:** 1(1) **Sequences:** 1(1)
Rip1p [Saccharomyces cerevisiae YJM1573]
gb|AAS56667.1| **Mass:** 23635 **Score:** 49 **Matches:** 1(1) **Sequences:** 1(1)
YEL024W [Saccharomyces cerevisiae]
gb|AJU56724.1| **Mass:** 23621 **Score:** 49 **Matches:** 1(1) **Sequences:** 1(1)
Rip1p [Saccharomyces cerevisiae YJM1574]
gb|AJU56979.1| **Mass:** 23621 **Score:** 49 **Matches:** 1(1) **Sequences:** 1(1)
Rip1p [Saccharomyces cerevisiae YJM1592]
emb|CCF58216.1| **Mass:** 24389 **Score:** 49 **Matches:** 1(1) **Sequences:** 1(1)
hypothetical protein KAFR_0E00620 [Kazachstania africana CBS 2517]
emb|CAG60324.1| **Mass:** 23457 **Score:** 49 **Matches:** 1(1) **Sequences:** 1(1)
unnamed protein product [Candida glabrata]
emb|CCH60704.1| **Mass:** 21782 **Score:** 49 **Matches:** 1(1) **Sequences:** 1(1)
hypothetical protein TBLA_0D01970 [Tetrapisispora blattae CBS 6284]
emb|CEP62261.1| **Mass:** 23536 **Score:** 49 **Matches:** 1(1) **Sequences:** 1(1)
LALA0S05e01486g1_1 [Lachancea lanzarotensis]
gb|AAB64501.1| **Mass:** 23635 **Score:** 49 **Matches:** 1(1) **Sequences:** 1(1)
Rieske iron-sulfur protein of mitochondrial Ubiquinol-cytochrome c reductase [Saccharomyces cerevisiae]
emb|CCE66045.1| **Mass:** 23293 **Score:** 49 **Matches:** 1(1) **Sequences:** 1(1)
hypothetical protein TPHA_0O00760 [Tetrapisispora phaffii CBS 4417]
emb|CCK71115.1| **Mass:** 23715 **Score:** 49 **Matches:** 1(1) **Sequences:** 1(1)
hypothetical protein KNAG_0G00580 [Kazachstania naganishii CBS 8797]
gb|AHY75528.1| **Mass:** 23621 **Score:** 49 **Matches:** 1(1) **Sequences:** 1(1)
Rip1p [Saccharomyces cerevisiae YJM993]
emb|CAY79139.1| **Mass:** 23621 **Score:** 49 **Matches:** 1(1) **Sequences:** 1(1)
Rip1p [Saccharomyces cerevisiae EC1118]
emb|CCE83573.1| **Mass:** 23437 **Score:** 49 **Matches:** 1(1) **Sequences:** 1(1)
Piso0_004153 [Millerozyma farinosa CBS 7064]
emb|CCE84604.1| **Mass:** 23437 **Score:** 49 **Matches:** 1(1) **Sequences:** 1(1)
Piso0_004153 [Millerozyma farinosa CBS 7064]
emb|CAG89223.2| **Mass:** 23549 **Score:** 49 **Matches:** 1(1) **Sequences:** 1(1)
DEHA2F11726p [Debaryomyces hansenii CBS767]
emb|CCE90664.1| **Mass:** 23875 **Score:** 49 **Matches:** 1(1) **Sequences:** 1(1)
hypothetical protein TDEL_0B05350 [Torulaspora delbrueckii]
gb|AEY96922.1| **Mass:** 23119 **Score:** 49 **Matches:** 1(1) **Sequences:** 1(1)
FAEL067Wp [Eremothecium gossypii FDAG1]

---

9. emb|CCO02527.1| **Mass:** 20953 **Score:** 40 **Matches:** 1(0) **Sequences:** 1(0)
cytochrome oxidase subunit 2, partial (mitochondrion) [Saccharomyces cerevisiae]
☐ Check to include this hit in error tolerant search or archive report

Query	Observed	Mr(expt)	Mr(calc)	ppm	Miss	Score	Expect	Rank	Unique	Peptide
61	**1912.0969**	**1911.0897**	**1910.9772**	**58.9**	**0**	**40**	**0.14**	**1**	**U**	**R.LLDTDTSMVVPVDTHIR.F**

Proteins matching the same set of peptides:
gb|ABV08789.1| **Mass:** 22228 **Score:** 40 **Matches:** 1(0) **Sequences:** 1(0)
cytochrome oxidase subunit II, partial (mitochondrion) [Saccharomyces cerevisiae]
dbj|BAA09539.1| **Mass:** 17798 **Score:** 40 **Matches:** 1(0) **Sequences:** 1(0)
cytochrome c oxidase subunit II, partial (mitochondrion) [Saccharomyces cerevisiae]
gb|AJU15350.1| **Mass:** 28834 **Score:** 40 **Matches:** 1(0) **Sequences:** 1(0)
cytochrome c oxidase subunit 2 (mitochondrion) [Saccharomyces cerevisiae YJM189]
gb|AJU15360.1| **Mass:** 28777 **Score:** 40 **Matches:** 1(0) **Sequences:** 1(0)
cytochrome c oxidase subunit 2 (mitochondrion) [Saccharomyces cerevisiae YJM193]
gb|AJU15369.1| **Mass:** 28889 **Score:** 40 **Matches:** 1(0) **Sequences:** 1(0)
cytochrome c oxidase subunit 2 (mitochondrion) [Saccharomyces cerevisiae YJM195]
gb|AJU15377.1| **Mass:** 28834 **Score:** 40 **Matches:** 1(0) **Sequences:** 1(0)
cytochrome c oxidase subunit 2 (mitochondrion) [Saccharomyces cerevisiae YJM244]
gb|AJU15386.1| **Mass:** 28834 **Score:** 40 **Matches:** 1(0) **Sequences:** 1(0)
cytochrome c oxidase subunit 2 (mitochondrion) [Saccharomyces cerevisiae YJM248]
gb|AJU15394.1| **Mass:** 28834 **Score:** 40 **Matches:** 1(0) **Sequences:** 1(0)
cytochrome c oxidase subunit 2 (mitochondrion) [Saccharomyces cerevisiae YJM270]
gb|AJU15404.1| **Mass:** 28777 **Score:** 40 **Matches:** 1(0) **Sequences:** 1(0)
cytochrome c oxidase subunit 2 (mitochondrion) [Saccharomyces cerevisiae YJM271]
gb|AJU15414.1| **Mass:** 28777 **Score:** 40 **Matches:** 1(0) **Sequences:** 1(0)
cytochrome c oxidase subunit 2 (mitochondrion) [Saccharomyces cerevisiae YJM320]
gb|AJU15424.1| **Mass:** 28777 **Score:** 40 **Matches:** 1(0) **Sequences:** 1(0)
cytochrome c oxidase subunit 2 (mitochondrion) [Saccharomyces cerevisiae YJM326]
gb|AJU15434.1| **Mass:** 28777 **Score:** 40 **Matches:** 1(0) **Sequences:** 1(0)
cytochrome c oxidase subunit 2 (mitochondrion) [Saccharomyces cerevisiae YJM428]
gb|AJU15444.1| **Mass:** 28834 **Score:** 40 **Matches:** 1(0) **Sequences:** 1(0)
cytochrome c oxidase subunit 2 (mitochondrion) [Saccharomyces cerevisiae YJM450]
gb|AJU15454.1| **Mass:** 28834 **Score:** 40 **Matches:** 1(0) **Sequences:** 1(0)
cytochrome c oxidase subunit 2 (mitochondrion) [Saccharomyces cerevisiae YJM451]
gb|AJU15464.1| **Mass:** 28777 **Score:** 40 **Matches:** 1(0) **Sequences:** 1(0)

cytochrome c oxidase subunit 2 (mitochondrion) [Saccharomyces cerevisiae YJM453]
gb|AJU15474.1| **Mass:** 28834 **Score:** 40 **Matches:** 1(0) **Sequences:** 1(0)
cytochrome c oxidase subunit 2 (mitochondrion) [Saccharomyces cerevisiae YJM456]
gb|AJU15483.1| **Mass:** 28834 **Score:** 40 **Matches:** 1(0) **Sequences:** 1(0)
cytochrome c oxidase subunit 2 (mitochondrion) [Saccharomyces cerevisiae YJM470]
gb|AJU15493.1| **Mass:** 28777 **Score:** 40 **Matches:** 1(0) **Sequences:** 1(0)
cytochrome c oxidase subunit 2 (mitochondrion) [Saccharomyces cerevisiae YJM541]
gb|AJU15503.1| **Mass:** 28777 **Score:** 40 **Matches:** 1(0) **Sequences:** 1(0)
cytochrome c oxidase subunit 2 (mitochondrion) [Saccharomyces cerevisiae YJM554]
gb|AJU15513.1| **Mass:** 28834 **Score:** 40 **Matches:** 1(0) **Sequences:** 1(0)
cytochrome c oxidase subunit 2 (mitochondrion) [Saccharomyces cerevisiae YJM555]
gb|AJU15522.1| **Mass:** 28777 **Score:** 40 **Matches:** 1(0) **Sequences:** 1(0)
cytochrome c oxidase subunit 2 (mitochondrion) [Saccharomyces cerevisiae YJM627]
gb|AJU15531.1| **Mass:** 28834 **Score:** 40 **Matches:** 1(0) **Sequences:** 1(0)
cytochrome c oxidase subunit 2 (mitochondrion) [Saccharomyces cerevisiae YJM681]
gb|AJU15541.1| **Mass:** 28777 **Score:** 40 **Matches:** 1(0) **Sequences:** 1(0)
cytochrome c oxidase subunit 2 (mitochondrion) [Saccharomyces cerevisiae YJM682]
gb|AJU15551.1| **Mass:** 28777 **Score:** 40 **Matches:** 1(0) **Sequences:** 1(0)
cytochrome c oxidase subunit 2 (mitochondrion) [Saccharomyces cerevisiae YJM683]
gb|AJU15560.1| **Mass:** 28777 **Score:** 40 **Matches:** 1(0) **Sequences:** 1(0)
cytochrome c oxidase subunit 2 (mitochondrion) [Saccharomyces cerevisiae YJM689]
gb|AJU15570.1| **Mass:** 28777 **Score:** 40 **Matches:** 1(0) **Sequences:** 1(0)
cytochrome c oxidase subunit 2 (mitochondrion) [Saccharomyces cerevisiae YJM693]
gb|AJU15578.1| **Mass:** 28834 **Score:** 40 **Matches:** 1(0) **Sequences:** 1(0)
cytochrome c oxidase subunit 2 (mitochondrion) [Saccharomyces cerevisiae YJM969]
gb|AJU15586.1| **Mass:** 28834 **Score:** 40 **Matches:** 1(0) **Sequences:** 1(0)
cytochrome c oxidase subunit 2 (mitochondrion) [Saccharomyces cerevisiae YJM972]
gb|AJU15594.1| **Mass:** 28834 **Score:** 40 **Matches:** 1(0) **Sequences:** 1(0)
cytochrome c oxidase subunit 2 (mitochondrion) [Saccharomyces cerevisiae YJM975]
gb|AJU15602.1| **Mass:** 28834 **Score:** 40 **Matches:** 1(0) **Sequences:** 1(0)
cytochrome c oxidase subunit 2 (mitochondrion) [Saccharomyces cerevisiae YJM978]
gb|AJU15610.1| **Mass:** 28834 **Score:** 40 **Matches:** 1(0) **Sequences:** 1(0)
cytochrome c oxidase subunit 2 (mitochondrion) [Saccharomyces cerevisiae YJM981]
gb|AJU15618.1| **Mass:** 28834 **Score:** 40 **Matches:** 1(0) **Sequences:** 1(0)
cytochrome c oxidase subunit 2 (mitochondrion) [Saccharomyces cerevisiae YJM984]
gb|AJU15626.1| **Mass:** 28834 **Score:** 40 **Matches:** 1(0) **Sequences:** 1(0)
cytochrome c oxidase subunit 2 (mitochondrion) [Saccharomyces cerevisiae YJM987]
gb|AJU15634.1| **Mass:** 28834 **Score:** 40 **Matches:** 1(0) **Sequences:** 1(0)
cytochrome c oxidase subunit 2 (mitochondrion) [Saccharomyces cerevisiae YJM990]
gb|AJU15642.1| **Mass:** 28834 **Score:** 40 **Matches:** 1(0) **Sequences:** 1(0)
cytochrome c oxidase subunit 2 (mitochondrion) [Saccharomyces cerevisiae YJM996]
gb|AJU15659.1| **Mass:** 28777 **Score:** 40 **Matches:** 1(0) **Sequences:** 1(0)
cytochrome c oxidase subunit 2 (mitochondrion) [Saccharomyces cerevisiae YJM1083]
gb|AJU15670.1| **Mass:** 28777 **Score:** 40 **Matches:** 1(0) **Sequences:** 1(0)
cytochrome c oxidase subunit 2 (mitochondrion) [Saccharomyces cerevisiae YJM1129]
gb|AJU15682.1| **Mass:** 28777 **Score:** 40 **Matches:** 1(0) **Sequences:** 1(0)
cytochrome c oxidase subunit 2 (mitochondrion) [Saccharomyces cerevisiae YJM1133]
gb|AJU15697.1| **Mass:** 28834 **Score:** 40 **Matches:** 1(0) **Sequences:** 1(0)
cytochrome c oxidase subunit 2 (mitochondrion) [Saccharomyces cerevisiae YJM1190]
gb|AJU15712.1| **Mass:** 28834 **Score:** 40 **Matches:** 1(0) **Sequences:** 1(0)
cytochrome c oxidase subunit 2 (mitochondrion) [Saccharomyces cerevisiae YJM1199]
gb|AJU15727.1| **Mass:** 28834 **Score:** 40 **Matches:** 1(0) **Sequences:** 1(0)
cytochrome c oxidase subunit 2 (mitochondrion) [Saccharomyces cerevisiae YJM1202]
gb|AJU15740.1| **Mass:** 28834 **Score:** 40 **Matches:** 1(0) **Sequences:** 1(0)
cytochrome c oxidase subunit 2 (mitochondrion) [Saccharomyces cerevisiae YJM1208]
gb|AJU15749.1| **Mass:** 28777 **Score:** 40 **Matches:** 1(0) **Sequences:** 1(0)
cytochrome c oxidase subunit 2 (mitochondrion) [Saccharomyces cerevisiae YJM1242]
gb|AJU15760.1| **Mass:** 28777 **Score:** 40 **Matches:** 1(0) **Sequences:** 1(0)
cytochrome c oxidase subunit 2 (mitochondrion) [Saccharomyces cerevisiae YJM1244]
gb|AJU15769.1| **Mass:** 28777 **Score:** 40 **Matches:** 1(0) **Sequences:** 1(0)
cytochrome c oxidase subunit 2 (mitochondrion) [Saccharomyces cerevisiae YJM1248]
gb|AJU15780.1| **Mass:** 28834 **Score:** 40 **Matches:** 1(0) **Sequences:** 1(0)
cytochrome c oxidase subunit 2 (mitochondrion) [Saccharomyces cerevisiae YJM1250]
gb|AJU15792.1| **Mass:** 28777 **Score:** 40 **Matches:** 1(0) **Sequences:** 1(0)
cytochrome c oxidase subunit 2 (mitochondrion) [Saccharomyces cerevisiae YJM1252]
gb|AJU15804.1| **Mass:** 28834 **Score:** 40 **Matches:** 1(0) **Sequences:** 1(0)
cytochrome c oxidase subunit 2 (mitochondrion) [Saccharomyces cerevisiae YJM1273]
gb|AJU15817.1| **Mass:** 28834 **Score:** 40 **Matches:** 1(0) **Sequences:** 1(0)
cytochrome c oxidase subunit 2 (mitochondrion) [Saccharomyces cerevisiae YJM1304]
gb|AJU15829.1| **Mass:** 28777 **Score:** 40 **Matches:** 1(0) **Sequences:** 1(0)
cytochrome c oxidase subunit 2 (mitochondrion) [Saccharomyces cerevisiae YJM1307]
gb|AJU15843.1| **Mass:** 28777 **Score:** 40 **Matches:** 1(0) **Sequences:** 1(0)
cytochrome c oxidase subunit 2 (mitochondrion) [Saccharomyces cerevisiae YJM1311]
gb|AJU15855.1| **Mass:** 28834 **Score:** 40 **Matches:** 1(0) **Sequences:** 1(0)
cytochrome c oxidase subunit 2 (mitochondrion) [Saccharomyces cerevisiae YJM1326]
gb|AJU15866.1| **Mass:** 28777 **Score:** 40 **Matches:** 1(0) **Sequences:** 1(0)
cytochrome c oxidase subunit 2 (mitochondrion) [Saccharomyces cerevisiae YJM1332]
gb|AJU15877.1| **Mass:** 28777 **Score:** 40 **Matches:** 1(0) **Sequences:** 1(0)
cytochrome c oxidase subunit 2 (mitochondrion) [Saccharomyces cerevisiae YJM1336]
gb|AJU15888.1| **Mass:** 28834 **Score:** 40 **Matches:** 1(0) **Sequences:** 1(0)
cytochrome c oxidase subunit 2 (mitochondrion) [Saccharomyces cerevisiae YJM1338]
gb|AJU15898.1| **Mass:** 28834 **Score:** 40 **Matches:** 1(0) **Sequences:** 1(0)
cytochrome c oxidase subunit 2 (mitochondrion) [Saccharomyces cerevisiae YJM1341]
gb|AJU15912.1| **Mass:** 28834 **Score:** 40 **Matches:** 1(0) **Sequences:** 1(0)
cytochrome c oxidase subunit 2 (mitochondrion) [Saccharomyces cerevisiae YJM1342]
gb|AJU15924.1| **Mass:** 28777 **Score:** 40 **Matches:** 1(0) **Sequences:** 1(0)
cytochrome c oxidase subunit 2 (mitochondrion) [Saccharomyces cerevisiae YJM1355]
gb|AJU15937.1| **Mass:** 28777 **Score:** 40 **Matches:** 1(0) **Sequences:** 1(0)

cytochrome c oxidase subunit 2 (mitochondrion) [Saccharomyces cerevisiae YJM1356]
gb|AJU15950.1| **Mass:** 28777 **Score:** 40 **Matches:** 1(0) **Sequences:** 1(0)
cytochrome c oxidase subunit 2 (mitochondrion) [Saccharomyces cerevisiae YJM1381]
gb|AJU15965.1| **Mass:** 28834 **Score:** 40 **Matches:** 1(0) **Sequences:** 1(0)
cytochrome c oxidase subunit 2 (mitochondrion) [Saccharomyces cerevisiae YJM1383]
gb|AJU15975.1| **Mass:** 28777 **Score:** 40 **Matches:** 1(0) **Sequences:** 1(0)
cytochrome c oxidase subunit 2 (mitochondrion) [Saccharomyces cerevisiae YJM1385]
gb|AJU15985.1| **Mass:** 28834 **Score:** 40 **Matches:** 1(0) **Sequences:** 1(0)
cytochrome c oxidase subunit 2 (mitochondrion) [Saccharomyces cerevisiae YJM1386]
gb|AJU15994.1| **Mass:** 28834 **Score:** 40 **Matches:** 1(0) **Sequences:** 1(0)
cytochrome c oxidase subunit 2 (mitochondrion) [Saccharomyces cerevisiae YJM1387]
gb|AJU16003.1| **Mass:** 28834 **Score:** 40 **Matches:** 1(0) **Sequences:** 1(0)
cytochrome c oxidase subunit 2 (mitochondrion) [Saccharomyces cerevisiae YJM1388]
gb|AJU16012.1| **Mass:** 28834 **Score:** 40 **Matches:** 1(0) **Sequences:** 1(0)
cytochrome c oxidase subunit 2 (mitochondrion) [Saccharomyces cerevisiae YJM1389]
gb|AJU16021.1| **Mass:** 28834 **Score:** 40 **Matches:** 1(0) **Sequences:** 1(0)
cytochrome c oxidase subunit 2 (mitochondrion) [Saccharomyces cerevisiae YJM1399]
gb|AJU16030.1| **Mass:** 28777 **Score:** 40 **Matches:** 1(0) **Sequences:** 1(0)
cytochrome c oxidase subunit 2 (mitochondrion) [Saccharomyces cerevisiae YJM1400]
gb|AJU16039.1| **Mass:** 28777 **Score:** 40 **Matches:** 1(0) **Sequences:** 1(0)
cytochrome c oxidase subunit 2 (mitochondrion) [Saccharomyces cerevisiae YJM1401]
gb|AJU16047.1| **Mass:** 28834 **Score:** 40 **Matches:** 1(0) **Sequences:** 1(0)
cytochrome c oxidase subunit 2 (mitochondrion) [Saccharomyces cerevisiae YJM1402]
gb|AJU16056.1| **Mass:** 28834 **Score:** 40 **Matches:** 1(0) **Sequences:** 1(0)
cytochrome c oxidase subunit 2 (mitochondrion) [Saccharomyces cerevisiae YJM1415]
gb|AJU16064.1| **Mass:** 28834 **Score:** 40 **Matches:** 1(0) **Sequences:** 1(0)
cytochrome c oxidase subunit 2 (mitochondrion) [Saccharomyces cerevisiae YJM1417]
gb|AJU16072.1| **Mass:** 28834 **Score:** 40 **Matches:** 1(0) **Sequences:** 1(0)
cytochrome c oxidase subunit 2 (mitochondrion) [Saccharomyces cerevisiae YJM1418]
gb|AJU16082.1| **Mass:** 28834 **Score:** 40 **Matches:** 1(0) **Sequences:** 1(0)
cytochrome c oxidase subunit 2 (mitochondrion) [Saccharomyces cerevisiae YJM1419]
gb|AJU16090.1| **Mass:** 28834 **Score:** 40 **Matches:** 1(0) **Sequences:** 1(0)
cytochrome c oxidase subunit 2 (mitochondrion) [Saccharomyces cerevisiae YJM1433]
gb|AJU16098.1| **Mass:** 28834 **Score:** 40 **Matches:** 1(0) **Sequences:** 1(0)
cytochrome c oxidase subunit 2 (mitochondrion) [Saccharomyces cerevisiae YJM1434]
gb|AJU16107.1| **Mass:** 28777 **Score:** 40 **Matches:** 1(0) **Sequences:** 1(0)
cytochrome c oxidase subunit 2 (mitochondrion) [Saccharomyces cerevisiae YJM1439]
gb|AJU16116.1| **Mass:** 28834 **Score:** 40 **Matches:** 1(0) **Sequences:** 1(0)
cytochrome c oxidase subunit 2 (mitochondrion) [Saccharomyces cerevisiae YJM1443]
gb|AJU16126.1| **Mass:** 28834 **Score:** 40 **Matches:** 1(0) **Sequences:** 1(0)
cytochrome c oxidase subunit 2 (mitochondrion) [Saccharomyces cerevisiae YJM1444]
gb|AJU16134.1| **Mass:** 28834 **Score:** 40 **Matches:** 1(0) **Sequences:** 1(0)
cytochrome c oxidase subunit 2 (mitochondrion) [Saccharomyces cerevisiae YJM1447]
gb|AJU16142.1| **Mass:** 28834 **Score:** 40 **Matches:** 1(0) **Sequences:** 1(0)
cytochrome c oxidase subunit 2 (mitochondrion) [Saccharomyces cerevisiae YJM1450]
gb|AJU16151.1| **Mass:** 28834 **Score:** 40 **Matches:** 1(0) **Sequences:** 1(0)
cytochrome c oxidase subunit 2 (mitochondrion) [Saccharomyces cerevisiae YJM1460]
gb|AJU16160.1| **Mass:** 28777 **Score:** 40 **Matches:** 1(0) **Sequences:** 1(0)
cytochrome c oxidase subunit 2 (mitochondrion) [Saccharomyces cerevisiae YJM1463]
gb|AJU16169.1| **Mass:** 28777 **Score:** 40 **Matches:** 1(0) **Sequences:** 1(0)
cytochrome c oxidase subunit 2 (mitochondrion) [Saccharomyces cerevisiae YJM1477]
gb|AJU16178.1| **Mass:** 28777 **Score:** 40 **Matches:** 1(0) **Sequences:** 1(0)
cytochrome c oxidase subunit 2 (mitochondrion) [Saccharomyces cerevisiae YJM1478]
gb|AJU16187.1| **Mass:** 28777 **Score:** 40 **Matches:** 1(0) **Sequences:** 1(0)
cytochrome c oxidase subunit 2 (mitochondrion) [Saccharomyces cerevisiae YJM1479]
gb|AJU16195.1| **Mass:** 28777 **Score:** 40 **Matches:** 1(0) **Sequences:** 1(0)
cytochrome c oxidase subunit 2 (mitochondrion) [Saccharomyces cerevisiae YJM1526]
gb|AJU16203.1| **Mass:** 28834 **Score:** 40 **Matches:** 1(0) **Sequences:** 1(0)
cytochrome c oxidase subunit 2 (mitochondrion) [Saccharomyces cerevisiae YJM1527]
gb|AJU16211.1| **Mass:** 28834 **Score:** 40 **Matches:** 1(0) **Sequences:** 1(0)
cytochrome c oxidase subunit 2 (mitochondrion) [Saccharomyces cerevisiae YJM1549]
gb|AJU16219.1| **Mass:** 28834 **Score:** 40 **Matches:** 1(0) **Sequences:** 1(0)
cytochrome c oxidase subunit 2 (mitochondrion) [Saccharomyces cerevisiae YJM1573]
gb|AJU16228.1| **Mass:** 28834 **Score:** 40 **Matches:** 1(0) **Sequences:** 1(0)
cytochrome c oxidase subunit 2 (mitochondrion) [Saccharomyces cerevisiae YJM1574]
gb|AJU16237.1| **Mass:** 28834 **Score:** 40 **Matches:** 1(0) **Sequences:** 1(0)
cytochrome c oxidase subunit 2 (mitochondrion) [Saccharomyces cerevisiae YJM1592]
gb|AJU16247.1| **Mass:** 28834 **Score:** 40 **Matches:** 1(0) **Sequences:** 1(0)
cytochrome c oxidase subunit 2 (mitochondrion) [Saccharomyces cerevisiae YJM1615]
emb|CAA24056.1| **Mass:** 28777 **Score:** 40 **Matches:** 1(0) **Sequences:** 1(0)
unnamed protein product (mitochondrion) [Saccharomyces cerevisiae]
emb|CAA24078.1| **Mass:** 28777 **Score:** 40 **Matches:** 1(0) **Sequences:** 1(0)
unnamed protein product (mitochondrion) [Saccharomyces cerevisiae]
gb|ABS28697.1| **Mass:** 28834 **Score:** 40 **Matches:** 1(0) **Sequences:** 1(0)
cytochrome c oxidase subunit II (mitochondrion) [Saccharomyces cerevisiae YJM789]
emb|CAM34704.2| **Mass:** 17798 **Score:** 40 **Matches:** 1(0) **Sequences:** 1(0)
cytochrome oxidase, partial (mitochondrion) [Saccharomyces cerevisiae]
emb|CCW36724.1| **Mass:** 19875 **Score:** 40 **Matches:** 1(0) **Sequences:** 1(0)
subunit II of cytochrome c oxidase, partial (mitochondrion) [Saccharomyces cerevisiae]
emb|CCW36725.1| **Mass:** 19875 **Score:** 40 **Matches:** 1(0) **Sequences:** 1(0)
subunit II of cytochrome c oxidase, partial (mitochondrion) [Saccharomyces cerevisiae]
emb|CCW36726.1| **Mass:** 19875 **Score:** 40 **Matches:** 1(0) **Sequences:** 1(0)
subunit II of cytochrome c oxidase, partial (mitochondrion) [Saccharomyces cerevisiae]
emb|CCW36727.1| **Mass:** 19875 **Score:** 40 **Matches:** 1(0) **Sequences:** 1(0)
subunit II of cytochrome c oxidase, partial (mitochondrion) [Saccharomyces cerevisiae]
emb|CCW36728.1| **Mass:** 19899 **Score:** 40 **Matches:** 1(0) **Sequences:** 1(0)
subunit II of cytochrome c oxidase, partial (mitochondrion) [Saccharomyces cerevisiae]
emb|CCW36729.1| **Mass:** 19875 **Score:** 40 **Matches:** 1(0) **Sequences:** 1(0)

subunit II of cytochrome c oxidase, partial (mitochondrion) [Saccharomyces cerevisiae]
emb|CCW36730.1| **Mass:** 19875 **Score:** 40 **Matches:** 1(0) **Sequences:** 1(0)
subunit II of cytochrome c oxidase, partial (mitochondrion) [Saccharomyces cerevisiae]
emb|CCW36731.1| **Mass:** 19875 **Score:** 40 **Matches:** 1(0) **Sequences:** 1(0)
subunit II of cytochrome c oxidase, partial (mitochondrion) [Saccharomyces cerevisiae]
emb|CCW36732.1| **Mass:** 19932 **Score:** 40 **Matches:** 1(0) **Sequences:** 1(0)
subunit II of cytochrome c oxidase, partial (mitochondrion) [Saccharomyces cerevisiae]
emb|CCW36733.1| **Mass:** 19932 **Score:** 40 **Matches:** 1(0) **Sequences:** 1(0)
subunit II of cytochrome c oxidase, partial (mitochondrion) [Saccharomyces cerevisiae]
emb|CCW36734.1| **Mass:** 19875 **Score:** 40 **Matches:** 1(0) **Sequences:** 1(0)
subunit II of cytochrome c oxidase, partial (mitochondrion) [Saccharomyces cerevisiae]
gb|AJP42330.1| **Mass:** 28777 **Score:** 40 **Matches:** 1(0) **Sequences:** 1(0)
cytochrome c oxidase subunit 2 (mitochondrion) [Saccharomyces cerevisiae YJM1078]
gb|AAO47349.1| **Mass:** 23100 **Score:** 40 **Matches:** 1(0) **Sequences:** 1(0)
cytochrome c oxidase subunit II, partial (mitochondrion) [Saccharomyces cerevisiae]
gb|AAP57773.1| **Mass:** 22285 **Score:** 40 **Matches:** 1(0) **Sequences:** 1(0)
cytochrome oxidase subunit 2, partial (mitochondrion) [Saccharomyces cerevisiae]
gb|AAO60058.1| **Mass:** 23100 **Score:** 40 **Matches:** 1(0) **Sequences:** 1(0)
cytochrome c oxidase subunit II, partial (mitochondrion) [Saccharomyces cerevisiae]
gb|AAO60059.1| **Mass:** 23100 **Score:** 40 **Matches:** 1(0) **Sequences:** 1(0)
cytochrome c oxidase subunit II, partial (mitochondrion) [Saccharomyces cerevisiae]
gb|AAO60060.1| **Mass:** 23100 **Score:** 40 **Matches:** 1(0) **Sequences:** 1(0)
cytochrome c oxidase subunit II, partial (mitochondrion) [Saccharomyces cerevisiae]
gb|AAO60061.1| **Mass:** 23100 **Score:** 40 **Matches:** 1(0) **Sequences:** 1(0)
cytochrome c oxidase subunit II, partial (mitochondrion) [Saccharomyces cerevisiae]
gb|AAO60062.1| **Mass:** 23100 **Score:** 40 **Matches:** 1(0) **Sequences:** 1(0)
cytochrome c oxidase subunit II, partial (mitochondrion) [Saccharomyces cerevisiae]
gb|AAO60063.1| **Mass:** 23100 **Score:** 40 **Matches:** 1(0) **Sequences:** 1(0)
cytochrome c oxidase subunit II, partial (mitochondrion) [Saccharomyces cerevisiae]
gb|AAO60064.1| **Mass:** 23100 **Score:** 40 **Matches:** 1(0) **Sequences:** 1(0)
cytochrome c oxidase subunit II, partial (mitochondrion) [Saccharomyces cerevisiae]
gb|AFP66962.1| **Mass:** 22285 **Score:** 40 **Matches:** 1(0) **Sequences:** 1(0)
cytochrome c oxidase subunit II, partial (mitochondrion) [Saccharomyces cerevisiae]
gb|AFP66963.1| **Mass:** 22285 **Score:** 40 **Matches:** 1(0) **Sequences:** 1(0)
cytochrome c oxidase subunit II, partial (mitochondrion) [Saccharomyces cerevisiae]
gb|AFP66964.1| **Mass:** 22285 **Score:** 40 **Matches:** 1(0) **Sequences:** 1(0)
cytochrome c oxidase subunit II, partial (mitochondrion) [Saccharomyces cerevisiae]
gb|AFP66967.1| **Mass:** 22285 **Score:** 40 **Matches:** 1(0) **Sequences:** 1(0)
cytochrome c oxidase subunit II, partial (mitochondrion) [Saccharomyces cerevisiae]
gb|AFP66968.1| **Mass:** 22285 **Score:** 40 **Matches:** 1(0) **Sequences:** 1(0)
cytochrome c oxidase subunit II, partial (mitochondrion) [Saccharomyces cerevisiae]
gb|AFP66969.1| **Mass:** 22285 **Score:** 40 **Matches:** 1(0) **Sequences:** 1(0)
cytochrome c oxidase subunit II, partial (mitochondrion) [Saccharomyces cerevisiae]
gb|AFP66970.1| **Mass:** 22285 **Score:** 40 **Matches:** 1(0) **Sequences:** 1(0)
cytochrome c oxidase subunit II, partial (mitochondrion) [Saccharomyces cerevisiae]
gb|AFP66971.1| **Mass:** 22228 **Score:** 40 **Matches:** 1(0) **Sequences:** 1(0)
cytochrome c oxidase subunit II, partial (mitochondrion) [Saccharomyces cerevisiae]
gb|AFP66972.1| **Mass:** 22228 **Score:** 40 **Matches:** 1(0) **Sequences:** 1(0)
cytochrome c oxidase subunit II, partial (mitochondrion) [Saccharomyces cerevisiae]
gb|AFP66973.1| **Mass:** 22228 **Score:** 40 **Matches:** 1(0) **Sequences:** 1(0)
cytochrome c oxidase subunit II, partial (mitochondrion) [Saccharomyces cerevisiae]
gb|AFP66974.1| **Mass:** 22285 **Score:** 40 **Matches:** 1(0) **Sequences:** 1(0)
cytochrome c oxidase subunit II, partial (mitochondrion) [Saccharomyces cerevisiae]
gb|AFP66975.1| **Mass:** 22228 **Score:** 40 **Matches:** 1(0) **Sequences:** 1(0)
cytochrome c oxidase subunit II, partial (mitochondrion) [Saccharomyces cerevisiae]
gb|AFP66976.1| **Mass:** 22228 **Score:** 40 **Matches:** 1(0) **Sequences:** 1(0)
cytochrome c oxidase subunit II, partial (mitochondrion) [Saccharomyces cerevisiae]
gb|AFP66977.1| **Mass:** 22285 **Score:** 40 **Matches:** 1(0) **Sequences:** 1(0)
cytochrome c oxidase subunit II, partial (mitochondrion) [Saccharomyces cerevisiae]
gb|AFP66978.1| **Mass:** 22228 **Score:** 40 **Matches:** 1(0) **Sequences:** 1(0)
cytochrome c oxidase subunit II, partial (mitochondrion) [Saccharomyces cerevisiae]
gb|AFP66979.1| **Mass:** 22228 **Score:** 40 **Matches:** 1(0) **Sequences:** 1(0)
cytochrome c oxidase subunit II, partial (mitochondrion) [Saccharomyces cerevisiae]
gb|AFP66980.1| **Mass:** 22285 **Score:** 40 **Matches:** 1(0) **Sequences:** 1(0)
cytochrome c oxidase subunit II, partial (mitochondrion) [Saccharomyces cerevisiae]
gb|AFP66981.1| **Mass:** 22228 **Score:** 40 **Matches:** 1(0) **Sequences:** 1(0)
cytochrome c oxidase subunit II, partial (mitochondrion) [Saccharomyces cerevisiae]
gb|AFP66982.1| **Mass:** 22228 **Score:** 40 **Matches:** 1(0) **Sequences:** 1(0)
cytochrome c oxidase subunit II, partial (mitochondrion) [Saccharomyces cerevisiae]
gb|AFP66983.1| **Mass:** 22285 **Score:** 40 **Matches:** 1(0) **Sequences:** 1(0)
cytochrome c oxidase subunit II, partial (mitochondrion) [Saccharomyces cerevisiae]
gb|AFP66984.1| **Mass:** 22228 **Score:** 40 **Matches:** 1(0) **Sequences:** 1(0)
cytochrome c oxidase subunit II, partial (mitochondrion) [Saccharomyces cerevisiae]
gb|AFP66985.1| **Mass:** 22228 **Score:** 40 **Matches:** 1(0) **Sequences:** 1(0)
cytochrome c oxidase subunit II, partial (mitochondrion) [Saccharomyces cerevisiae]
gb|AFP66986.1| **Mass:** 22228 **Score:** 40 **Matches:** 1(0) **Sequences:** 1(0)
cytochrome c oxidase subunit II, partial (mitochondrion) [Saccharomyces cerevisiae]
gb|AFP66987.1| **Mass:** 22228 **Score:** 40 **Matches:** 1(0) **Sequences:** 1(0)
cytochrome c oxidase subunit II, partial (mitochondrion) [Saccharomyces cerevisiae]
gb|AFP66988.1| **Mass:** 22228 **Score:** 40 **Matches:** 1(0) **Sequences:** 1(0)
cytochrome c oxidase subunit II, partial (mitochondrion) [Saccharomyces cerevisiae]
gb|AFP66989.1| **Mass:** 22228 **Score:** 40 **Matches:** 1(0) **Sequences:** 1(0)
cytochrome c oxidase subunit II, partial (mitochondrion) [Saccharomyces cerevisiae]
gb|AFP66990.1| **Mass:** 22228 **Score:** 40 **Matches:** 1(0) **Sequences:** 1(0)
cytochrome c oxidase subunit II, partial (mitochondrion) [Saccharomyces cerevisiae]
gb|AFP66991.1| **Mass:** 22228 **Score:** 40 **Matches:** 1(0) **Sequences:** 1(0)
cytochrome c oxidase subunit II, partial (mitochondrion) [Saccharomyces cerevisiae]
gb|AFP66992.1| **Mass:** 22228 **Score:** 40 **Matches:** 1(0) **Sequences:** 1(0)

cytochrome c oxidase subunit II, partial (mitochondrion) [Saccharomyces cerevisiae]
gb|AFP66993.1| **Mass:** 22285 **Score:** 40 **Matches:** 1(0) **Sequences:** 1(0)
cytochrome c oxidase subunit II, partial (mitochondrion) [Saccharomyces cerevisiae]
gb|AFP66994.1| **Mass:** 22285 **Score:** 40 **Matches:** 1(0) **Sequences:** 1(0)
cytochrome c oxidase subunit II, partial (mitochondrion) [Saccharomyces cerevisiae]
gb|AFP66995.1| **Mass:** 22228 **Score:** 40 **Matches:** 1(0) **Sequences:** 1(0)
cytochrome c oxidase subunit II, partial (mitochondrion) [Saccharomyces cerevisiae]
gb|AFP66996.1| **Mass:** 22228 **Score:** 40 **Matches:** 1(0) **Sequences:** 1(0)
cytochrome c oxidase subunit II, partial (mitochondrion) [Saccharomyces cerevisiae]
gb|AFP66997.1| **Mass:** 22228 **Score:** 40 **Matches:** 1(0) **Sequences:** 1(0)
cytochrome c oxidase subunit II, partial (mitochondrion) [Saccharomyces cerevisiae]
gb|AFP66998.1| **Mass:** 22228 **Score:** 40 **Matches:** 1(0) **Sequences:** 1(0)
cytochrome c oxidase subunit II, partial (mitochondrion) [Saccharomyces cerevisiae]
gb|AFP66999.1| **Mass:** 22228 **Score:** 40 **Matches:** 1(0) **Sequences:** 1(0)
cytochrome c oxidase subunit II, partial (mitochondrion) [Saccharomyces cerevisiae]
gb|AFP67000.1| **Mass:** 22285 **Score:** 40 **Matches:** 1(0) **Sequences:** 1(0)
cytochrome c oxidase subunit II, partial (mitochondrion) [Saccharomyces cerevisiae]
gb|AFP67001.1| **Mass:** 22228 **Score:** 40 **Matches:** 1(0) **Sequences:** 1(0)
cytochrome c oxidase subunit II, partial (mitochondrion) [Saccharomyces cerevisiae]
gb|AFP67002.1| **Mass:** 22228 **Score:** 40 **Matches:** 1(0) **Sequences:** 1(0)
cytochrome c oxidase subunit II, partial (mitochondrion) [Saccharomyces cerevisiae]
gb|AFP67003.1| **Mass:** 22228 **Score:** 40 **Matches:** 1(0) **Sequences:** 1(0)
cytochrome c oxidase subunit II, partial (mitochondrion) [Saccharomyces cerevisiae]
gb|AFP67004.1| **Mass:** 22228 **Score:** 40 **Matches:** 1(0) **Sequences:** 1(0)
cytochrome c oxidase subunit II, partial (mitochondrion) [Saccharomyces cerevisiae]
gb|AFP67005.1| **Mass:** 22285 **Score:** 40 **Matches:** 1(0) **Sequences:** 1(0)
cytochrome c oxidase subunit II, partial (mitochondrion) [Saccharomyces cerevisiae]
gb|AFP67006.1| **Mass:** 22285 **Score:** 40 **Matches:** 1(0) **Sequences:** 1(0)
cytochrome c oxidase subunit II, partial (mitochondrion) [Saccharomyces cerevisiae]
gb|AFP67007.1| **Mass:** 22228 **Score:** 40 **Matches:** 1(0) **Sequences:** 1(0)
cytochrome c oxidase subunit II, partial (mitochondrion) [Saccharomyces cerevisiae]
gb|AFP67008.1| **Mass:** 22228 **Score:** 40 **Matches:** 1(0) **Sequences:** 1(0)
cytochrome c oxidase subunit II, partial (mitochondrion) [Saccharomyces cerevisiae]
gb|AFP67009.1| **Mass:** 22228 **Score:** 40 **Matches:** 1(0) **Sequences:** 1(0)
cytochrome c oxidase subunit II, partial (mitochondrion) [Saccharomyces cerevisiae]
gb|AFP67010.1| **Mass:** 22228 **Score:** 40 **Matches:** 1(0) **Sequences:** 1(0)
cytochrome c oxidase subunit II, partial (mitochondrion) [Saccharomyces cerevisiae]
gb|AFP67011.1| **Mass:** 22228 **Score:** 40 **Matches:** 1(0) **Sequences:** 1(0)
cytochrome c oxidase subunit II, partial (mitochondrion) [Saccharomyces cerevisiae]
gb|AFP67012.1| **Mass:** 22285 **Score:** 40 **Matches:** 1(0) **Sequences:** 1(0)
cytochrome c oxidase subunit II, partial (mitochondrion) [Saccharomyces cerevisiae]
gb|AFP67013.1| **Mass:** 22285 **Score:** 40 **Matches:** 1(0) **Sequences:** 1(0)
cytochrome c oxidase subunit II, partial (mitochondrion) [Saccharomyces cerevisiae]
gb|AFP67014.1| **Mass:** 22273 **Score:** 40 **Matches:** 1(0) **Sequences:** 1(0)
cytochrome c oxidase subunit II, partial (mitochondrion) [Saccharomyces cerevisiae]
gb|AFP67015.1| **Mass:** 22285 **Score:** 40 **Matches:** 1(0) **Sequences:** 1(0)
cytochrome c oxidase subunit II, partial (mitochondrion) [Saccharomyces cerevisiae]
gb|AFP67016.1| **Mass:** 22285 **Score:** 40 **Matches:** 1(0) **Sequences:** 1(0)
cytochrome c oxidase subunit II, partial (mitochondrion) [Saccharomyces cerevisiae]
gb|AFP67017.1| **Mass:** 22285 **Score:** 40 **Matches:** 1(0) **Sequences:** 1(0)
cytochrome c oxidase subunit II, partial (mitochondrion) [Saccharomyces cerevisiae]
gb|AFP67018.1| **Mass:** 22285 **Score:** 40 **Matches:** 1(0) **Sequences:** 1(0)
cytochrome c oxidase subunit II, partial (mitochondrion) [Saccharomyces cerevisiae]
gb|AFP67019.1| **Mass:** 22228 **Score:** 40 **Matches:** 1(0) **Sequences:** 1(0)
cytochrome c oxidase subunit II, partial (mitochondrion) [Saccharomyces cerevisiae]
gb|AFP67020.1| **Mass:** 22228 **Score:** 40 **Matches:** 1(0) **Sequences:** 1(0)
cytochrome c oxidase subunit II, partial (mitochondrion) [Saccharomyces cerevisiae]
gb|AFP67021.1| **Mass:** 22285 **Score:** 40 **Matches:** 1(0) **Sequences:** 1(0)
cytochrome c oxidase subunit II, partial (mitochondrion) [Saccharomyces cerevisiae]
gb|AFP67022.1| **Mass:** 22228 **Score:** 40 **Matches:** 1(0) **Sequences:** 1(0)
cytochrome c oxidase subunit II, partial (mitochondrion) [Saccharomyces cerevisiae]
gb|AFP67023.1| **Mass:** 22285 **Score:** 40 **Matches:** 1(0) **Sequences:** 1(0)
cytochrome c oxidase subunit II, partial (mitochondrion) [Saccharomyces cerevisiae]
gb|AFP67024.1| **Mass:** 22285 **Score:** 40 **Matches:** 1(0) **Sequences:** 1(0)
cytochrome c oxidase subunit II, partial (mitochondrion) [Saccharomyces cerevisiae]
gb|AFP67025.1| **Mass:** 22285 **Score:** 40 **Matches:** 1(0) **Sequences:** 1(0)
cytochrome c oxidase subunit II, partial (mitochondrion) [Saccharomyces cerevisiae]
gb|AFP67026.1| **Mass:** 22285 **Score:** 40 **Matches:** 1(0) **Sequences:** 1(0)
cytochrome c oxidase subunit II, partial (mitochondrion) [Saccharomyces cerevisiae]
gb|AFP67027.1| **Mass:** 22285 **Score:** 40 **Matches:** 1(0) **Sequences:** 1(0)
cytochrome c oxidase subunit II, partial (mitochondrion) [Saccharomyces cerevisiae]
gb|AFP67028.1| **Mass:** 22285 **Score:** 40 **Matches:** 1(0) **Sequences:** 1(0)
cytochrome c oxidase subunit II, partial (mitochondrion) [Saccharomyces cerevisiae]
gb|AFP67029.1| **Mass:** 22285 **Score:** 40 **Matches:** 1(0) **Sequences:** 1(0)
cytochrome c oxidase subunit II, partial (mitochondrion) [Saccharomyces cerevisiae]
gb|AFP67030.1| **Mass:** 22285 **Score:** 40 **Matches:** 1(0) **Sequences:** 1(0)
cytochrome c oxidase subunit II, partial (mitochondrion) [Saccharomyces cerevisiae]
gb|AFP67031.1| **Mass:** 22285 **Score:** 40 **Matches:** 1(0) **Sequences:** 1(0)
cytochrome c oxidase subunit II, partial (mitochondrion) [Saccharomyces cerevisiae]
gb|AFP67032.1| **Mass:** 22285 **Score:** 40 **Matches:** 1(0) **Sequences:** 1(0)
cytochrome c oxidase subunit II, partial (mitochondrion) [Saccharomyces cerevisiae]
gb|AFP67033.1| **Mass:** 22285 **Score:** 40 **Matches:** 1(0) **Sequences:** 1(0)
cytochrome c oxidase subunit II, partial (mitochondrion) [Saccharomyces cerevisiae]
gb|AFP67034.1| **Mass:** 22285 **Score:** 40 **Matches:** 1(0) **Sequences:** 1(0)
cytochrome c oxidase subunit II, partial (mitochondrion) [Saccharomyces cerevisiae]
gb|AFP67035.1| **Mass:** 22285 **Score:** 40 **Matches:** 1(0) **Sequences:** 1(0)
cytochrome c oxidase subunit II, partial (mitochondrion) [Saccharomyces cerevisiae]
gb|AFP67036.1| **Mass:** 22285 **Score:** 40 **Matches:** 1(0) **Sequences:** 1(0)

cytochrome c oxidase subunit II, partial (mitochondrion) [Saccharomyces cerevisiae]
gb|AFP67037.1| **Mass:** 22285 **Score:** 40 **Matches:** 1(0) **Sequences:** 1(0)
cytochrome c oxidase subunit II, partial (mitochondrion) [Saccharomyces cerevisiae]
gb|AFP67038.1| **Mass:** 22228 **Score:** 40 **Matches:** 1(0) **Sequences:** 1(0)
cytochrome c oxidase subunit II, partial (mitochondrion) [Saccharomyces cerevisiae]
gb|AFP67039.1| **Mass:** 22285 **Score:** 40 **Matches:** 1(0) **Sequences:** 1(0)
cytochrome c oxidase subunit II, partial (mitochondrion) [Saccharomyces cerevisiae]
gb|AFP67040.1| **Mass:** 22285 **Score:** 40 **Matches:** 1(0) **Sequences:** 1(0)
cytochrome c oxidase subunit II, partial (mitochondrion) [Saccharomyces cerevisiae]
gb|AFP67041.1| **Mass:** 22285 **Score:** 40 **Matches:** 1(0) **Sequences:** 1(0)
cytochrome c oxidase subunit II, partial (mitochondrion) [Saccharomyces cerevisiae]
gb|AFP67042.1| **Mass:** 22285 **Score:** 40 **Matches:** 1(0) **Sequences:** 1(0)
cytochrome c oxidase subunit II, partial (mitochondrion) [Saccharomyces cerevisiae]
gb|AFP67043.1| **Mass:** 22285 **Score:** 40 **Matches:** 1(0) **Sequences:** 1(0)
cytochrome c oxidase subunit II, partial (mitochondrion) [Saccharomyces cerevisiae]
gb|AFP67044.1| **Mass:** 22285 **Score:** 40 **Matches:** 1(0) **Sequences:** 1(0)
cytochrome c oxidase subunit II, partial (mitochondrion) [Saccharomyces cerevisiae]
gb|AFP67045.1| **Mass:** 22285 **Score:** 40 **Matches:** 1(0) **Sequences:** 1(0)
cytochrome c oxidase subunit II, partial (mitochondrion) [Saccharomyces cerevisiae]
gb|AFP67046.1| **Mass:** 22285 **Score:** 40 **Matches:** 1(0) **Sequences:** 1(0)
cytochrome c oxidase subunit II, partial (mitochondrion) [Saccharomyces cerevisiae]
gb|AFP67047.1| **Mass:** 22285 **Score:** 40 **Matches:** 1(0) **Sequences:** 1(0)
cytochrome c oxidase subunit II, partial (mitochondrion) [Saccharomyces cerevisiae]
gb|AFP67048.1| **Mass:** 22285 **Score:** 40 **Matches:** 1(0) **Sequences:** 1(0)
cytochrome c oxidase subunit II, partial (mitochondrion) [Saccharomyces cerevisiae]
gb|AFP67049.1| **Mass:** 22285 **Score:** 40 **Matches:** 1(0) **Sequences:** 1(0)
cytochrome c oxidase subunit II, partial (mitochondrion) [Saccharomyces cerevisiae]
gb|AFP67050.1| **Mass:** 22285 **Score:** 40 **Matches:** 1(0) **Sequences:** 1(0)
cytochrome c oxidase subunit II, partial (mitochondrion) [Saccharomyces cerevisiae]
gb|AFP67051.1| **Mass:** 22285 **Score:** 40 **Matches:** 1(0) **Sequences:** 1(0)
cytochrome c oxidase subunit II, partial (mitochondrion) [Saccharomyces cerevisiae]
gb|AFP67052.1| **Mass:** 22285 **Score:** 40 **Matches:** 1(0) **Sequences:** 1(0)
cytochrome c oxidase subunit II, partial (mitochondrion) [Saccharomyces cerevisiae]
gb|AFP67053.1| **Mass:** 22285 **Score:** 40 **Matches:** 1(0) **Sequences:** 1(0)
cytochrome c oxidase subunit II, partial (mitochondrion) [Saccharomyces cerevisiae]
gb|AFP67054.1| **Mass:** 22285 **Score:** 40 **Matches:** 1(0) **Sequences:** 1(0)
cytochrome c oxidase subunit II, partial (mitochondrion) [Saccharomyces cerevisiae]
gb|AFP67055.1| **Mass:** 22285 **Score:** 40 **Matches:** 1(0) **Sequences:** 1(0)
cytochrome c oxidase subunit II, partial (mitochondrion) [Saccharomyces cerevisiae]
gb|AFP67056.1| **Mass:** 22285 **Score:** 40 **Matches:** 1(0) **Sequences:** 1(0)
cytochrome c oxidase subunit II, partial (mitochondrion) [Saccharomyces cerevisiae]
gb|AFP67057.1| **Mass:** 22285 **Score:** 40 **Matches:** 1(0) **Sequences:** 1(0)
cytochrome c oxidase subunit II, partial (mitochondrion) [Saccharomyces cerevisiae]
gb|AFP67058.1| **Mass:** 22285 **Score:** 40 **Matches:** 1(0) **Sequences:** 1(0)
cytochrome c oxidase subunit II, partial (mitochondrion) [Saccharomyces cerevisiae]
gb|AFP67059.1| **Mass:** 22228 **Score:** 40 **Matches:** 1(0) **Sequences:** 1(0)
cytochrome c oxidase subunit II, partial (mitochondrion) [Saccharomyces cerevisiae]
gb|AFP67060.1| **Mass:** 22228 **Score:** 40 **Matches:** 1(0) **Sequences:** 1(0)
cytochrome c oxidase subunit II, partial (mitochondrion) [Saccharomyces cerevisiae]
gb|AFP67061.1| **Mass:** 22228 **Score:** 40 **Matches:** 1(0) **Sequences:** 1(0)
cytochrome c oxidase subunit II, partial (mitochondrion) [Saccharomyces cerevisiae]
gb|AFP67062.1| **Mass:** 22228 **Score:** 40 **Matches:** 1(0) **Sequences:** 1(0)
cytochrome c oxidase subunit II, partial (mitochondrion) [Saccharomyces cerevisiae]
gb|AFP67063.1| **Mass:** 22228 **Score:** 40 **Matches:** 1(0) **Sequences:** 1(0)
cytochrome c oxidase subunit II, partial (mitochondrion) [Saccharomyces cerevisiae]
gb|AFP67064.1| **Mass:** 22228 **Score:** 40 **Matches:** 1(0) **Sequences:** 1(0)
cytochrome c oxidase subunit II, partial (mitochondrion) [Saccharomyces cerevisiae]
gb|AFP67065.1| **Mass:** 22228 **Score:** 40 **Matches:** 1(0) **Sequences:** 1(0)
cytochrome c oxidase subunit II, partial (mitochondrion) [Saccharomyces cerevisiae]
gb|AFP67066.1| **Mass:** 22228 **Score:** 40 **Matches:** 1(0) **Sequences:** 1(0)
cytochrome c oxidase subunit II, partial (mitochondrion) [Saccharomyces cerevisiae]
gb|AFP67067.1| **Mass:** 22228 **Score:** 40 **Matches:** 1(0) **Sequences:** 1(0)
cytochrome c oxidase subunit II, partial (mitochondrion) [Saccharomyces cerevisiae]
gb|AFP67068.1| **Mass:** 22228 **Score:** 40 **Matches:** 1(0) **Sequences:** 1(0)
cytochrome c oxidase subunit II, partial (mitochondrion) [Saccharomyces cerevisiae]
gb|AFP67069.1| **Mass:** 22228 **Score:** 40 **Matches:** 1(0) **Sequences:** 1(0)
cytochrome c oxidase subunit II, partial (mitochondrion) [Saccharomyces cerevisiae]
gb|AFP67070.1| **Mass:** 22228 **Score:** 40 **Matches:** 1(0) **Sequences:** 1(0)
cytochrome c oxidase subunit II, partial (mitochondrion) [Saccharomyces cerevisiae]
gb|AFP67071.1| **Mass:** 22228 **Score:** 40 **Matches:** 1(0) **Sequences:** 1(0)
cytochrome c oxidase subunit II, partial (mitochondrion) [Saccharomyces cerevisiae]
gb|AFP67072.1| **Mass:** 22285 **Score:** 40 **Matches:** 1(0) **Sequences:** 1(0)
cytochrome c oxidase subunit II, partial (mitochondrion) [Saccharomyces cerevisiae]
gb|AFP67073.1| **Mass:** 22285 **Score:** 40 **Matches:** 1(0) **Sequences:** 1(0)
cytochrome c oxidase subunit II, partial (mitochondrion) [Saccharomyces cerevisiae]
gb|AFP67074.1| **Mass:** 22228 **Score:** 40 **Matches:** 1(0) **Sequences:** 1(0)
cytochrome c oxidase subunit II, partial (mitochondrion) [Saccharomyces cerevisiae]
gb|AFP67075.1| **Mass:** 22228 **Score:** 40 **Matches:** 1(0) **Sequences:** 1(0)
cytochrome c oxidase subunit II, partial (mitochondrion) [Saccharomyces cerevisiae]
gb|AFP67076.1| **Mass:** 22228 **Score:** 40 **Matches:** 1(0) **Sequences:** 1(0)
cytochrome c oxidase subunit II, partial (mitochondrion) [Saccharomyces cerevisiae]
gb|AFP67077.1| **Mass:** 22228 **Score:** 40 **Matches:** 1(0) **Sequences:** 1(0)
cytochrome c oxidase subunit II, partial (mitochondrion) [Saccharomyces cerevisiae]
gb|AFP67078.1| **Mass:** 22228 **Score:** 40 **Matches:** 1(0) **Sequences:** 1(0)
cytochrome c oxidase subunit II, partial (mitochondrion) [Saccharomyces cerevisiae]
gb|AFP67079.1| **Mass:** 22228 **Score:** 40 **Matches:** 1(0) **Sequences:** 1(0)
cytochrome c oxidase subunit II, partial (mitochondrion) [Saccharomyces cerevisiae]
gb|AFP67080.1| **Mass:** 22228 **Score:** 40 **Matches:** 1(0) **Sequences:** 1(0)

cytochrome c oxidase subunit II, partial (mitochondrion) [Saccharomyces cerevisiae]
gb|AFP67081.1| **Mass:** 22228 **Score:** 40 **Matches:** 1(0) **Sequences:** 1(0)
cytochrome c oxidase subunit II, partial (mitochondrion) [Saccharomyces cerevisiae]
gb|AFP67082.1| **Mass:** 22228 **Score:** 40 **Matches:** 1(0) **Sequences:** 1(0)
cytochrome c oxidase subunit II, partial (mitochondrion) [Saccharomyces cerevisiae]
gb|AFP67083.1| **Mass:** 22228 **Score:** 40 **Matches:** 1(0) **Sequences:** 1(0)
cytochrome c oxidase subunit II, partial (mitochondrion) [Saccharomyces cerevisiae]
gb|AFP67084.1| **Mass:** 22228 **Score:** 40 **Matches:** 1(0) **Sequences:** 1(0)
cytochrome c oxidase subunit II, partial (mitochondrion) [Saccharomyces cerevisiae]
gb|AFP67085.1| **Mass:** 22228 **Score:** 40 **Matches:** 1(0) **Sequences:** 1(0)
cytochrome c oxidase subunit II, partial (mitochondrion) [Saccharomyces cerevisiae]
gb|AFP67086.1| **Mass:** 22228 **Score:** 40 **Matches:** 1(0) **Sequences:** 1(0)
cytochrome c oxidase subunit II, partial (mitochondrion) [Saccharomyces cerevisiae]
gb|AFP67087.1| **Mass:** 22228 **Score:** 40 **Matches:** 1(0) **Sequences:** 1(0)
cytochrome c oxidase subunit II, partial (mitochondrion) [Saccharomyces cerevisiae]
gb|AFP67088.1| **Mass:** 22228 **Score:** 40 **Matches:** 1(0) **Sequences:** 1(0)
cytochrome c oxidase subunit II, partial (mitochondrion) [Saccharomyces cerevisiae]
gb|AFP67089.1| **Mass:** 22228 **Score:** 40 **Matches:** 1(0) **Sequences:** 1(0)
cytochrome c oxidase subunit II, partial (mitochondrion) [Saccharomyces cerevisiae]
gb|AFP67090.1| **Mass:** 22228 **Score:** 40 **Matches:** 1(0) **Sequences:** 1(0)
cytochrome c oxidase subunit II, partial (mitochondrion) [Saccharomyces cerevisiae]
gb|AFP67091.1| **Mass:** 22228 **Score:** 40 **Matches:** 1(0) **Sequences:** 1(0)
cytochrome c oxidase subunit II, partial (mitochondrion) [Saccharomyces cerevisiae]
gb|AFP67092.1| **Mass:** 22285 **Score:** 40 **Matches:** 1(0) **Sequences:** 1(0)
cytochrome c oxidase subunit II, partial (mitochondrion) [Saccharomyces cerevisiae]
gb|AFP67093.1| **Mass:** 22285 **Score:** 40 **Matches:** 1(0) **Sequences:** 1(0)
cytochrome c oxidase subunit II, partial (mitochondrion) [Saccharomyces cerevisiae]
gb|AFP67094.1| **Mass:** 22285 **Score:** 40 **Matches:** 1(0) **Sequences:** 1(0)
cytochrome c oxidase subunit II, partial (mitochondrion) [Saccharomyces cerevisiae]
gb|AFP67095.1| **Mass:** 22228 **Score:** 40 **Matches:** 1(0) **Sequences:** 1(0)
cytochrome c oxidase subunit II, partial (mitochondrion) [Saccharomyces cerevisiae]
gb|AFP67096.1| **Mass:** 22228 **Score:** 40 **Matches:** 1(0) **Sequences:** 1(0)
cytochrome c oxidase subunit II, partial (mitochondrion) [Saccharomyces cerevisiae]
gb|AFP67097.1| **Mass:** 22285 **Score:** 40 **Matches:** 1(0) **Sequences:** 1(0)
cytochrome c oxidase subunit II, partial (mitochondrion) [Saccharomyces cerevisiae]
gb|AFP67098.1| **Mass:** 22285 **Score:** 40 **Matches:** 1(0) **Sequences:** 1(0)
cytochrome c oxidase subunit II, partial (mitochondrion) [Saccharomyces cerevisiae]
gb|AFP67099.1| **Mass:** 22285 **Score:** 40 **Matches:** 1(0) **Sequences:** 1(0)
cytochrome c oxidase subunit II, partial (mitochondrion) [Saccharomyces cerevisiae]
gb|AFP67100.1| **Mass:** 22285 **Score:** 40 **Matches:** 1(0) **Sequences:** 1(0)
cytochrome c oxidase subunit II, partial (mitochondrion) [Saccharomyces cerevisiae]
gb|AFP67101.1| **Mass:** 22285 **Score:** 40 **Matches:** 1(0) **Sequences:** 1(0)
cytochrome c oxidase subunit II, partial (mitochondrion) [Saccharomyces cerevisiae]
gb|AFP67102.1| **Mass:** 22285 **Score:** 40 **Matches:** 1(0) **Sequences:** 1(0)
cytochrome c oxidase subunit II, partial (mitochondrion) [Saccharomyces cerevisiae]
gb|AFP67103.1| **Mass:** 22285 **Score:** 40 **Matches:** 1(0) **Sequences:** 1(0)
cytochrome c oxidase subunit II, partial (mitochondrion) [Saccharomyces cerevisiae]
gb|AFP67104.1| **Mass:** 22285 **Score:** 40 **Matches:** 1(0) **Sequences:** 1(0)
cytochrome c oxidase subunit II, partial (mitochondrion) [Saccharomyces cerevisiae]
gb|AFP67105.1| **Mass:** 22285 **Score:** 40 **Matches:** 1(0) **Sequences:** 1(0)
cytochrome c oxidase subunit II, partial (mitochondrion) [Saccharomyces cerevisiae]
gb|AFP67106.1| **Mass:** 22285 **Score:** 40 **Matches:** 1(0) **Sequences:** 1(0)
cytochrome c oxidase subunit II, partial (mitochondrion) [Saccharomyces cerevisiae]
gb|AFP67107.1| **Mass:** 22285 **Score:** 40 **Matches:** 1(0) **Sequences:** 1(0)
cytochrome c oxidase subunit II, partial (mitochondrion) [Saccharomyces cerevisiae]
gb|AFP67108.1| **Mass:** 22285 **Score:** 40 **Matches:** 1(0) **Sequences:** 1(0)
cytochrome c oxidase subunit II, partial (mitochondrion) [Saccharomyces cerevisiae]
gb|AFP67109.1| **Mass:** 22285 **Score:** 40 **Matches:** 1(0) **Sequences:** 1(0)
cytochrome c oxidase subunit II, partial (mitochondrion) [Saccharomyces cerevisiae]
gb|AFP67110.1| **Mass:** 22285 **Score:** 40 **Matches:** 1(0) **Sequences:** 1(0)
cytochrome c oxidase subunit II, partial (mitochondrion) [Saccharomyces cerevisiae]
gb|AFP67111.1| **Mass:** 22285 **Score:** 40 **Matches:** 1(0) **Sequences:** 1(0)
cytochrome c oxidase subunit II, partial (mitochondrion) [Saccharomyces cerevisiae]
gb|AFP67112.1| **Mass:** 22285 **Score:** 40 **Matches:** 1(0) **Sequences:** 1(0)
cytochrome c oxidase subunit II, partial (mitochondrion) [Saccharomyces cerevisiae]
gb|AFP67113.1| **Mass:** 22285 **Score:** 40 **Matches:** 1(0) **Sequences:** 1(0)
cytochrome c oxidase subunit II, partial (mitochondrion) [Saccharomyces cerevisiae]
gb|AFP67114.1| **Mass:** 22285 **Score:** 40 **Matches:** 1(0) **Sequences:** 1(0)
cytochrome c oxidase subunit II, partial (mitochondrion) [Saccharomyces cerevisiae]
gb|AFP67115.1| **Mass:** 22285 **Score:** 40 **Matches:** 1(0) **Sequences:** 1(0)
cytochrome c oxidase subunit II, partial (mitochondrion) [Saccharomyces cerevisiae]
gb|AFP67116.1| **Mass:** 22285 **Score:** 40 **Matches:** 1(0) **Sequences:** 1(0)
cytochrome c oxidase subunit II, partial (mitochondrion) [Saccharomyces cerevisiae]
gb|AFP67117.1| **Mass:** 22285 **Score:** 40 **Matches:** 1(0) **Sequences:** 1(0)
cytochrome c oxidase subunit II, partial (mitochondrion) [Saccharomyces cerevisiae]
gb|AFP67118.1| **Mass:** 22285 **Score:** 40 **Matches:** 1(0) **Sequences:** 1(0)
cytochrome c oxidase subunit II, partial (mitochondrion) [Saccharomyces cerevisiae]
gb|AFP67119.1| **Mass:** 22285 **Score:** 40 **Matches:** 1(0) **Sequences:** 1(0)
cytochrome c oxidase subunit II, partial (mitochondrion) [Saccharomyces cerevisiae]
gb|AFP67120.1| **Mass:** 22285 **Score:** 40 **Matches:** 1(0) **Sequences:** 1(0)
cytochrome c oxidase subunit II, partial (mitochondrion) [Saccharomyces cerevisiae]
gb|AFP67121.1| **Mass:** 22285 **Score:** 40 **Matches:** 1(0) **Sequences:** 1(0)
cytochrome c oxidase subunit II, partial (mitochondrion) [Saccharomyces cerevisiae]
gb|AFP67122.1| **Mass:** 22285 **Score:** 40 **Matches:** 1(0) **Sequences:** 1(0)
cytochrome c oxidase subunit II, partial (mitochondrion) [Saccharomyces cerevisiae]
gb|AFP67123.1| **Mass:** 22285 **Score:** 40 **Matches:** 1(0) **Sequences:** 1(0)
cytochrome c oxidase subunit II, partial (mitochondrion) [Saccharomyces cerevisiae]
gb|AFP67124.1| **Mass:** 22285 **Score:** 40 **Matches:** 1(0) **Sequences:** 1(0)

cytochrome c oxidase subunit II, partial (mitochondrion) [Saccharomyces cerevisiae]
gb|AFP67125.1| **Mass:** 22285 **Score:** 40 **Matches:** 1(0) **Sequences:** 1(0)
cytochrome c oxidase subunit II, partial (mitochondrion) [Saccharomyces cerevisiae]
gb|AFP67126.1| **Mass:** 22285 **Score:** 40 **Matches:** 1(0) **Sequences:** 1(0)
cytochrome c oxidase subunit II, partial (mitochondrion) [Saccharomyces cerevisiae]
gb|AFP67127.1| **Mass:** 22285 **Score:** 40 **Matches:** 1(0) **Sequences:** 1(0)
cytochrome c oxidase subunit II, partial (mitochondrion) [Saccharomyces cerevisiae]
gb|AFP67128.1| **Mass:** 22285 **Score:** 40 **Matches:** 1(0) **Sequences:** 1(0)
cytochrome c oxidase subunit II, partial (mitochondrion) [Saccharomyces cerevisiae]
gb|AFP67129.1| **Mass:** 22285 **Score:** 40 **Matches:** 1(0) **Sequences:** 1(0)
cytochrome c oxidase subunit II, partial (mitochondrion) [Saccharomyces cerevisiae]
gb|AFP67130.1| **Mass:** 22285 **Score:** 40 **Matches:** 1(0) **Sequences:** 1(0)
cytochrome c oxidase subunit II, partial (mitochondrion) [Saccharomyces cerevisiae]
gb|AFP67131.1| **Mass:** 22285 **Score:** 40 **Matches:** 1(0) **Sequences:** 1(0)
cytochrome c oxidase subunit II, partial (mitochondrion) [Saccharomyces cerevisiae]
gb|AFP67132.1| **Mass:** 22285 **Score:** 40 **Matches:** 1(0) **Sequences:** 1(0)
cytochrome c oxidase subunit II, partial (mitochondrion) [Saccharomyces cerevisiae]
gb|AFP67133.1| **Mass:** 22285 **Score:** 40 **Matches:** 1(0) **Sequences:** 1(0)
cytochrome c oxidase subunit II, partial (mitochondrion) [Saccharomyces cerevisiae]
gb|AFP67134.1| **Mass:** 22285 **Score:** 40 **Matches:** 1(0) **Sequences:** 1(0)
cytochrome c oxidase subunit II, partial (mitochondrion) [Saccharomyces cerevisiae]
gb|AFP67135.1| **Mass:** 22285 **Score:** 40 **Matches:** 1(0) **Sequences:** 1(0)
cytochrome c oxidase subunit II, partial (mitochondrion) [Saccharomyces cerevisiae]
gb|AFP67136.1| **Mass:** 22285 **Score:** 40 **Matches:** 1(0) **Sequences:** 1(0)
cytochrome c oxidase subunit II, partial (mitochondrion) [Saccharomyces cerevisiae]
gb|AFP67137.1| **Mass:** 22285 **Score:** 40 **Matches:** 1(0) **Sequences:** 1(0)
cytochrome c oxidase subunit II, partial (mitochondrion) [Saccharomyces cerevisiae]
gb|AFP67138.1| **Mass:** 22285 **Score:** 40 **Matches:** 1(0) **Sequences:** 1(0)
cytochrome c oxidase subunit II, partial (mitochondrion) [Saccharomyces cerevisiae]
gb|AFP67139.1| **Mass:** 22285 **Score:** 40 **Matches:** 1(0) **Sequences:** 1(0)
cytochrome c oxidase subunit II, partial (mitochondrion) [Saccharomyces cerevisiae]
gb|AFP67140.1| **Mass:** 22285 **Score:** 40 **Matches:** 1(0) **Sequences:** 1(0)
cytochrome c oxidase subunit II, partial (mitochondrion) [Saccharomyces cerevisiae]
gb|AFP67141.1| **Mass:** 22285 **Score:** 40 **Matches:** 1(0) **Sequences:** 1(0)
cytochrome c oxidase subunit II, partial (mitochondrion) [Saccharomyces cerevisiae]
gb|AFP67142.1| **Mass:** 22285 **Score:** 40 **Matches:** 1(0) **Sequences:** 1(0)
cytochrome c oxidase subunit II, partial (mitochondrion) [Saccharomyces cerevisiae]
gb|AFP67143.1| **Mass:** 22285 **Score:** 40 **Matches:** 1(0) **Sequences:** 1(0)
cytochrome c oxidase subunit II, partial (mitochondrion) [Saccharomyces cerevisiae]
gb|AFP67144.1| **Mass:** 22285 **Score:** 40 **Matches:** 1(0) **Sequences:** 1(0)
cytochrome c oxidase subunit II, partial (mitochondrion) [Saccharomyces cerevisiae]
gb|AFP67145.1| **Mass:** 22285 **Score:** 40 **Matches:** 1(0) **Sequences:** 1(0)
cytochrome c oxidase subunit II, partial (mitochondrion) [Saccharomyces cerevisiae]
gb|AFP67146.1| **Mass:** 22285 **Score:** 40 **Matches:** 1(0) **Sequences:** 1(0)
cytochrome c oxidase subunit II, partial (mitochondrion) [Saccharomyces cerevisiae]
gb|AFP67147.1| **Mass:** 22285 **Score:** 40 **Matches:** 1(0) **Sequences:** 1(0)
cytochrome c oxidase subunit II, partial (mitochondrion) [Saccharomyces cerevisiae]
gb|AFP67148.1| **Mass:** 22285 **Score:** 40 **Matches:** 1(0) **Sequences:** 1(0)
cytochrome c oxidase subunit II, partial (mitochondrion) [Saccharomyces cerevisiae]
gb|AFP67149.1| **Mass:** 22285 **Score:** 40 **Matches:** 1(0) **Sequences:** 1(0)
cytochrome c oxidase subunit II, partial (mitochondrion) [Saccharomyces cerevisiae]
gb|AFP67150.1| **Mass:** 22285 **Score:** 40 **Matches:** 1(0) **Sequences:** 1(0)
cytochrome c oxidase subunit II, partial (mitochondrion) [Saccharomyces cerevisiae]
gb|AFP67151.1| **Mass:** 22228 **Score:** 40 **Matches:** 1(0) **Sequences:** 1(0)
cytochrome c oxidase subunit II, partial (mitochondrion) [Saccharomyces cerevisiae]
gb|AFP67152.1| **Mass:** 22228 **Score:** 40 **Matches:** 1(0) **Sequences:** 1(0)
cytochrome c oxidase subunit II, partial (mitochondrion) [Saccharomyces cerevisiae]
gb|AFP67153.1| **Mass:** 22228 **Score:** 40 **Matches:** 1(0) **Sequences:** 1(0)
cytochrome c oxidase subunit II, partial (mitochondrion) [Saccharomyces cerevisiae]
gb|AFP67154.1| **Mass:** 22228 **Score:** 40 **Matches:** 1(0) **Sequences:** 1(0)
cytochrome c oxidase subunit II, partial (mitochondrion) [Saccharomyces cerevisiae]
gb|AFP67155.1| **Mass:** 22228 **Score:** 40 **Matches:** 1(0) **Sequences:** 1(0)
cytochrome c oxidase subunit II, partial (mitochondrion) [Saccharomyces cerevisiae]
gb|AFP67156.1| **Mass:** 22228 **Score:** 40 **Matches:** 1(0) **Sequences:** 1(0)
cytochrome c oxidase subunit II, partial (mitochondrion) [Saccharomyces cerevisiae]
gb|AFP67157.1| **Mass:** 22228 **Score:** 40 **Matches:** 1(0) **Sequences:** 1(0)
cytochrome c oxidase subunit II, partial (mitochondrion) [Saccharomyces cerevisiae]
gb|AFP67158.1| **Mass:** 22228 **Score:** 40 **Matches:** 1(0) **Sequences:** 1(0)
cytochrome c oxidase subunit II, partial (mitochondrion) [Saccharomyces cerevisiae]
gb|AFP67159.1| **Mass:** 22228 **Score:** 40 **Matches:** 1(0) **Sequences:** 1(0)
cytochrome c oxidase subunit II, partial (mitochondrion) [Saccharomyces cerevisiae]
gb|AFP67160.1| **Mass:** 22228 **Score:** 40 **Matches:** 1(0) **Sequences:** 1(0)
cytochrome c oxidase subunit II, partial (mitochondrion) [Saccharomyces cerevisiae]
gb|AFP67161.1| **Mass:** 22228 **Score:** 40 **Matches:** 1(0) **Sequences:** 1(0)
cytochrome c oxidase subunit II, partial (mitochondrion) [Saccharomyces cerevisiae]
gb|AFP67162.1| **Mass:** 22228 **Score:** 40 **Matches:** 1(0) **Sequences:** 1(0)
cytochrome c oxidase subunit II, partial (mitochondrion) [Saccharomyces cerevisiae]
gb|AFP67163.1| **Mass:** 22228 **Score:** 40 **Matches:** 1(0) **Sequences:** 1(0)
cytochrome c oxidase subunit II, partial (mitochondrion) [Saccharomyces cerevisiae]
gb|AFP67164.1| **Mass:** 22228 **Score:** 40 **Matches:** 1(0) **Sequences:** 1(0)
cytochrome c oxidase subunit II, partial (mitochondrion) [Saccharomyces cerevisiae]
gb|AFP67165.1| **Mass:** 22228 **Score:** 40 **Matches:** 1(0) **Sequences:** 1(0)
cytochrome c oxidase subunit II, partial (mitochondrion) [Saccharomyces cerevisiae]
gb|AFP67166.1| **Mass:** 22228 **Score:** 40 **Matches:** 1(0) **Sequences:** 1(0)
cytochrome c oxidase subunit II, partial (mitochondrion) [Saccharomyces cerevisiae]
gb|AFP67167.1| **Mass:** 22228 **Score:** 40 **Matches:** 1(0) **Sequences:** 1(0)
cytochrome c oxidase subunit II, partial (mitochondrion) [Saccharomyces cerevisiae]
gb|AFP67168.1| **Mass:** 22228 **Score:** 40 **Matches:** 1(0) **Sequences:** 1(0)

cytochrome c oxidase subunit II, partial (mitochondrion) [Saccharomyces cerevisiae]
gb|AFP67169.1| **Mass:** 22228 **Score:** 40 **Matches:** 1(0) **Sequences:** 1(0)
cytochrome c oxidase subunit II, partial (mitochondrion) [Saccharomyces cerevisiae]
gb|AFP67170.1| **Mass:** 22228 **Score:** 40 **Matches:** 1(0) **Sequences:** 1(0)
cytochrome c oxidase subunit II, partial (mitochondrion) [Saccharomyces cerevisiae]
gb|AFP67171.1| **Mass:** 22228 **Score:** 40 **Matches:** 1(0) **Sequences:** 1(0)
cytochrome c oxidase subunit II, partial (mitochondrion) [Saccharomyces cerevisiae]
gb|AFP67172.1| **Mass:** 22228 **Score:** 40 **Matches:** 1(0) **Sequences:** 1(0)
cytochrome c oxidase subunit II, partial (mitochondrion) [Saccharomyces cerevisiae]
gb|AFP67173.1| **Mass:** 22228 **Score:** 40 **Matches:** 1(0) **Sequences:** 1(0)
cytochrome c oxidase subunit II, partial (mitochondrion) [Saccharomyces cerevisiae]
gb|AFP67174.1| **Mass:** 22228 **Score:** 40 **Matches:** 1(0) **Sequences:** 1(0)
cytochrome c oxidase subunit II, partial (mitochondrion) [Saccharomyces cerevisiae]
gb|AFP67175.1| **Mass:** 22228 **Score:** 40 **Matches:** 1(0) **Sequences:** 1(0)
cytochrome c oxidase subunit II, partial (mitochondrion) [Saccharomyces cerevisiae]
gb|AFP67176.1| **Mass:** 22228 **Score:** 40 **Matches:** 1(0) **Sequences:** 1(0)
cytochrome c oxidase subunit II, partial (mitochondrion) [Saccharomyces cerevisiae]
gb|AFP67177.1| **Mass:** 22228 **Score:** 40 **Matches:** 1(0) **Sequences:** 1(0)
cytochrome c oxidase subunit II, partial (mitochondrion) [Saccharomyces cerevisiae]
gb|AFP67178.1| **Mass:** 22228 **Score:** 40 **Matches:** 1(0) **Sequences:** 1(0)
cytochrome c oxidase subunit II, partial (mitochondrion) [Saccharomyces cerevisiae]
gb|AFP67179.1| **Mass:** 22228 **Score:** 40 **Matches:** 1(0) **Sequences:** 1(0)
cytochrome c oxidase subunit II, partial (mitochondrion) [Saccharomyces cerevisiae]
gb|AFP67180.1| **Mass:** 22228 **Score:** 40 **Matches:** 1(0) **Sequences:** 1(0)
cytochrome c oxidase subunit II, partial (mitochondrion) [Saccharomyces cerevisiae]
gb|AFP67181.1| **Mass:** 22228 **Score:** 40 **Matches:** 1(0) **Sequences:** 1(0)
cytochrome c oxidase subunit II, partial (mitochondrion) [Saccharomyces cerevisiae]
gb|AFP67182.1| **Mass:** 22228 **Score:** 40 **Matches:** 1(0) **Sequences:** 1(0)
cytochrome c oxidase subunit II, partial (mitochondrion) [Saccharomyces cerevisiae]
gb|AFP67183.1| **Mass:** 22228 **Score:** 40 **Matches:** 1(0) **Sequences:** 1(0)
cytochrome c oxidase subunit II, partial (mitochondrion) [Saccharomyces cerevisiae]
gb|AFP67184.1| **Mass:** 22179 **Score:** 40 **Matches:** 1(0) **Sequences:** 1(0)
cytochrome c oxidase subunit II, partial (mitochondrion) [Saccharomyces cerevisiae]
gb|AFP67185.1| **Mass:** 22285 **Score:** 40 **Matches:** 1(0) **Sequences:** 1(0)
cytochrome c oxidase subunit II, partial (mitochondrion) [Saccharomyces cerevisiae]
gb|AFP67186.1| **Mass:** 22285 **Score:** 40 **Matches:** 1(0) **Sequences:** 1(0)
cytochrome c oxidase subunit II, partial (mitochondrion) [Saccharomyces cerevisiae]
gb|AFP67187.1| **Mass:** 22285 **Score:** 40 **Matches:** 1(0) **Sequences:** 1(0)
cytochrome c oxidase subunit II, partial (mitochondrion) [Saccharomyces cerevisiae]
gb|AFP67188.1| **Mass:** 22285 **Score:** 40 **Matches:** 1(0) **Sequences:** 1(0)
cytochrome c oxidase subunit II, partial (mitochondrion) [Saccharomyces cerevisiae]
gb|AFP67189.1| **Mass:** 22285 **Score:** 40 **Matches:** 1(0) **Sequences:** 1(0)
cytochrome c oxidase subunit II, partial (mitochondrion) [Saccharomyces cerevisiae]
gb|AFP67190.1| **Mass:** 22285 **Score:** 40 **Matches:** 1(0) **Sequences:** 1(0)
cytochrome c oxidase subunit II, partial (mitochondrion) [Saccharomyces cerevisiae]
gb|AFP67191.1| **Mass:** 22285 **Score:** 40 **Matches:** 1(0) **Sequences:** 1(0)
cytochrome c oxidase subunit II, partial (mitochondrion) [Saccharomyces cerevisiae]
gb|AFP67192.1| **Mass:** 22285 **Score:** 40 **Matches:** 1(0) **Sequences:** 1(0)
cytochrome c oxidase subunit II, partial (mitochondrion) [Saccharomyces cerevisiae]
gb|AFP67193.1| **Mass:** 22285 **Score:** 40 **Matches:** 1(0) **Sequences:** 1(0)
cytochrome c oxidase subunit II, partial (mitochondrion) [Saccharomyces cerevisiae]
gb|AFP67194.1| **Mass:** 22285 **Score:** 40 **Matches:** 1(0) **Sequences:** 1(0)
cytochrome c oxidase subunit II, partial (mitochondrion) [Saccharomyces cerevisiae]
gb|AFP67195.1| **Mass:** 22285 **Score:** 40 **Matches:** 1(0) **Sequences:** 1(0)
cytochrome c oxidase subunit II, partial (mitochondrion) [Saccharomyces cerevisiae]
gb|AFP67196.1| **Mass:** 22285 **Score:** 40 **Matches:** 1(0) **Sequences:** 1(0)
cytochrome c oxidase subunit II, partial (mitochondrion) [Saccharomyces cerevisiae]
gb|AFP67197.1| **Mass:** 22285 **Score:** 40 **Matches:** 1(0) **Sequences:** 1(0)
cytochrome c oxidase subunit II, partial (mitochondrion) [Saccharomyces cerevisiae]
gb|AFP67198.1| **Mass:** 22285 **Score:** 40 **Matches:** 1(0) **Sequences:** 1(0)
cytochrome c oxidase subunit II, partial (mitochondrion) [Saccharomyces cerevisiae]
gb|AFP67199.1| **Mass:** 22228 **Score:** 40 **Matches:** 1(0) **Sequences:** 1(0)
cytochrome c oxidase subunit II, partial (mitochondrion) [Saccharomyces cerevisiae]
gb|AFP67200.1| **Mass:** 22228 **Score:** 40 **Matches:** 1(0) **Sequences:** 1(0)
cytochrome c oxidase subunit II, partial (mitochondrion) [Saccharomyces cerevisiae]
gb|AFP67201.1| **Mass:** 22228 **Score:** 40 **Matches:** 1(0) **Sequences:** 1(0)
cytochrome c oxidase subunit II, partial (mitochondrion) [Saccharomyces cerevisiae]
gb|AFP67202.1| **Mass:** 22228 **Score:** 40 **Matches:** 1(0) **Sequences:** 1(0)
cytochrome c oxidase subunit II, partial (mitochondrion) [Saccharomyces cerevisiae]
gb|AFP67203.1| **Mass:** 22272 **Score:** 40 **Matches:** 1(0) **Sequences:** 1(0)
cytochrome c oxidase subunit II, partial (mitochondrion) [Saccharomyces cerevisiae]
gb|AFP67204.1| **Mass:** 22285 **Score:** 40 **Matches:** 1(0) **Sequences:** 1(0)
cytochrome c oxidase subunit II, partial (mitochondrion) [Saccharomyces cerevisiae]
gb|AFP67205.1| **Mass:** 22285 **Score:** 40 **Matches:** 1(0) **Sequences:** 1(0)
cytochrome c oxidase subunit II, partial (mitochondrion) [Saccharomyces cerevisiae]
gb|AFP67206.1| **Mass:** 22285 **Score:** 40 **Matches:** 1(0) **Sequences:** 1(0)
cytochrome c oxidase subunit II, partial (mitochondrion) [Saccharomyces cerevisiae]
gb|AFP67207.1| **Mass:** 22285 **Score:** 40 **Matches:** 1(0) **Sequences:** 1(0)
cytochrome c oxidase subunit II, partial (mitochondrion) [Saccharomyces cerevisiae]
gb|AFP67208.1| **Mass:** 22285 **Score:** 40 **Matches:** 1(0) **Sequences:** 1(0)
cytochrome c oxidase subunit II, partial (mitochondrion) [Saccharomyces cerevisiae]
gb|AFP67209.1| **Mass:** 22285 **Score:** 40 **Matches:** 1(0) **Sequences:** 1(0)
cytochrome c oxidase subunit II, partial (mitochondrion) [Saccharomyces cerevisiae]
gb|AFP67210.1| **Mass:** 22285 **Score:** 40 **Matches:** 1(0) **Sequences:** 1(0)
cytochrome c oxidase subunit II, partial (mitochondrion) [Saccharomyces cerevisiae]
gb|AFP67211.1| **Mass:** 22285 **Score:** 40 **Matches:** 1(0) **Sequences:** 1(0)
cytochrome c oxidase subunit II, partial (mitochondrion) [Saccharomyces cerevisiae]
gb|AFP67212.1| **Mass:** 22285 **Score:** 40 **Matches:** 1(0) **Sequences:** 1(0)

cytochrome c oxidase subunit II, partial (mitochondrion) [Saccharomyces cerevisiae]
gb|AFP67213.1| **Mass:** 22285 **Score:** 40 **Matches:** 1(0) **Sequences:** 1(0)
cytochrome c oxidase subunit II, partial (mitochondrion) [Saccharomyces cerevisiae]
gb|AFP67214.1| **Mass:** 22285 **Score:** 40 **Matches:** 1(0) **Sequences:** 1(0)
cytochrome c oxidase subunit II, partial (mitochondrion) [Saccharomyces cerevisiae]
gb|AFP67215.1| **Mass:** 22285 **Score:** 40 **Matches:** 1(0) **Sequences:** 1(0)
cytochrome c oxidase subunit II, partial (mitochondrion) [Saccharomyces cerevisiae]
gb|AFP67216.1| **Mass:** 22285 **Score:** 40 **Matches:** 1(0) **Sequences:** 1(0)
cytochrome c oxidase subunit II, partial (mitochondrion) [Saccharomyces cerevisiae]
gb|AFP67217.1| **Mass:** 22285 **Score:** 40 **Matches:** 1(0) **Sequences:** 1(0)
cytochrome c oxidase subunit II, partial (mitochondrion) [Saccharomyces cerevisiae]
gb|AFP67218.1| **Mass:** 22285 **Score:** 40 **Matches:** 1(0) **Sequences:** 1(0)
cytochrome c oxidase subunit II, partial (mitochondrion) [Saccharomyces cerevisiae]
gb|AFP67219.1| **Mass:** 22285 **Score:** 40 **Matches:** 1(0) **Sequences:** 1(0)
cytochrome c oxidase subunit II, partial (mitochondrion) [Saccharomyces cerevisiae]
gb|AFP67220.1| **Mass:** 22285 **Score:** 40 **Matches:** 1(0) **Sequences:** 1(0)
cytochrome c oxidase subunit II, partial (mitochondrion) [Saccharomyces cerevisiae]
gb|AFP67221.1| **Mass:** 22285 **Score:** 40 **Matches:** 1(0) **Sequences:** 1(0)
cytochrome c oxidase subunit II, partial (mitochondrion) [Saccharomyces cerevisiae]
gb|AFP67222.1| **Mass:** 22285 **Score:** 40 **Matches:** 1(0) **Sequences:** 1(0)
cytochrome c oxidase subunit II, partial (mitochondrion) [Saccharomyces cerevisiae]
gb|AFP67223.1| **Mass:** 22285 **Score:** 40 **Matches:** 1(0) **Sequences:** 1(0)
cytochrome c oxidase subunit II, partial (mitochondrion) [Saccharomyces cerevisiae]
gb|AFP67224.1| **Mass:** 22285 **Score:** 40 **Matches:** 1(0) **Sequences:** 1(0)
cytochrome c oxidase subunit II, partial (mitochondrion) [Saccharomyces cerevisiae]
gb|AFP67225.1| **Mass:** 22285 **Score:** 40 **Matches:** 1(0) **Sequences:** 1(0)
cytochrome c oxidase subunit II, partial (mitochondrion) [Saccharomyces cerevisiae]
gb|AFP67226.1| **Mass:** 22285 **Score:** 40 **Matches:** 1(0) **Sequences:** 1(0)
cytochrome c oxidase subunit II, partial (mitochondrion) [Saccharomyces cerevisiae]
gb|AFP67227.1| **Mass:** 22228 **Score:** 40 **Matches:** 1(0) **Sequences:** 1(0)
cytochrome c oxidase subunit II, partial (mitochondrion) [Saccharomyces cerevisiae]
gb|AFP67228.1| **Mass:** 22228 **Score:** 40 **Matches:** 1(0) **Sequences:** 1(0)
cytochrome c oxidase subunit II, partial (mitochondrion) [Saccharomyces cerevisiae]
gb|AFP67229.1| **Mass:** 22228 **Score:** 40 **Matches:** 1(0) **Sequences:** 1(0)
cytochrome c oxidase subunit II, partial (mitochondrion) [Saccharomyces cerevisiae]
gb|AFP67230.1| **Mass:** 22228 **Score:** 40 **Matches:** 1(0) **Sequences:** 1(0)
cytochrome c oxidase subunit II, partial (mitochondrion) [Saccharomyces cerevisiae]
gb|AFP67231.1| **Mass:** 22285 **Score:** 40 **Matches:** 1(0) **Sequences:** 1(0)
cytochrome c oxidase subunit II, partial (mitochondrion) [Saccharomyces cerevisiae]
gb|AFP67232.1| **Mass:** 22285 **Score:** 40 **Matches:** 1(0) **Sequences:** 1(0)
cytochrome c oxidase subunit II, partial (mitochondrion) [Saccharomyces cerevisiae]
gb|AFP67233.1| **Mass:** 22285 **Score:** 40 **Matches:** 1(0) **Sequences:** 1(0)
cytochrome c oxidase subunit II, partial (mitochondrion) [Saccharomyces cerevisiae]
gb|AFP67234.1| **Mass:** 22285 **Score:** 40 **Matches:** 1(0) **Sequences:** 1(0)
cytochrome c oxidase subunit II, partial (mitochondrion) [Saccharomyces cerevisiae]
gb|AFP67235.1| **Mass:** 22285 **Score:** 40 **Matches:** 1(0) **Sequences:** 1(0)
cytochrome c oxidase subunit II, partial (mitochondrion) [Saccharomyces cerevisiae]
gb|AFP67236.1| **Mass:** 22285 **Score:** 40 **Matches:** 1(0) **Sequences:** 1(0)
cytochrome c oxidase subunit II, partial (mitochondrion) [Saccharomyces cerevisiae]
gb|AFP67237.1| **Mass:** 22285 **Score:** 40 **Matches:** 1(0) **Sequences:** 1(0)
cytochrome c oxidase subunit II, partial (mitochondrion) [Saccharomyces cerevisiae]
gb|AFP67238.1| **Mass:** 22285 **Score:** 40 **Matches:** 1(0) **Sequences:** 1(0)
cytochrome c oxidase subunit II, partial (mitochondrion) [Saccharomyces cerevisiae]
gb|AFP67239.1| **Mass:** 22285 **Score:** 40 **Matches:** 1(0) **Sequences:** 1(0)
cytochrome c oxidase subunit II, partial (mitochondrion) [Saccharomyces cerevisiae]
gb|AFP67240.1| **Mass:** 22285 **Score:** 40 **Matches:** 1(0) **Sequences:** 1(0)
cytochrome c oxidase subunit II, partial (mitochondrion) [Saccharomyces cerevisiae]
gb|AFP67241.1| **Mass:** 22228 **Score:** 40 **Matches:** 1(0) **Sequences:** 1(0)
cytochrome c oxidase subunit II, partial (mitochondrion) [Saccharomyces cerevisiae]
gb|AFP67242.1| **Mass:** 22228 **Score:** 40 **Matches:** 1(0) **Sequences:** 1(0)
cytochrome c oxidase subunit II, partial (mitochondrion) [Saccharomyces cerevisiae]
gb|AFP67243.1| **Mass:** 22228 **Score:** 40 **Matches:** 1(0) **Sequences:** 1(0)
cytochrome c oxidase subunit II, partial (mitochondrion) [Saccharomyces cerevisiae]
gb|AFP67244.1| **Mass:** 22228 **Score:** 40 **Matches:** 1(0) **Sequences:** 1(0)
cytochrome c oxidase subunit II, partial (mitochondrion) [Saccharomyces cerevisiae]
gb|AFP67245.1| **Mass:** 22228 **Score:** 40 **Matches:** 1(0) **Sequences:** 1(0)
cytochrome c oxidase subunit II, partial (mitochondrion) [Saccharomyces cerevisiae]
gb|AFP67246.1| **Mass:** 22228 **Score:** 40 **Matches:** 1(0) **Sequences:** 1(0)
cytochrome c oxidase subunit II, partial (mitochondrion) [Saccharomyces cerevisiae]
gb|AFP67247.1| **Mass:** 22228 **Score:** 40 **Matches:** 1(0) **Sequences:** 1(0)
cytochrome c oxidase subunit II, partial (mitochondrion) [Saccharomyces cerevisiae]
gb|AFP67248.1| **Mass:** 22228 **Score:** 40 **Matches:** 1(0) **Sequences:** 1(0)
cytochrome c oxidase subunit II, partial (mitochondrion) [Saccharomyces cerevisiae]
gb|AFP67249.1| **Mass:** 22228 **Score:** 40 **Matches:** 1(0) **Sequences:** 1(0)
cytochrome c oxidase subunit II, partial (mitochondrion) [Saccharomyces cerevisiae]
gb|AFP67250.1| **Mass:** 22228 **Score:** 40 **Matches:** 1(0) **Sequences:** 1(0)
cytochrome c oxidase subunit II, partial (mitochondrion) [Saccharomyces cerevisiae]
gb|AFP67251.1| **Mass:** 22228 **Score:** 40 **Matches:** 1(0) **Sequences:** 1(0)
cytochrome c oxidase subunit II, partial (mitochondrion) [Saccharomyces cerevisiae]
gb|AFP67252.1| **Mass:** 22228 **Score:** 40 **Matches:** 1(0) **Sequences:** 1(0)
cytochrome c oxidase subunit II, partial (mitochondrion) [Saccharomyces cerevisiae]
gb|AFP67253.1| **Mass:** 22228 **Score:** 40 **Matches:** 1(0) **Sequences:** 1(0)
cytochrome c oxidase subunit II, partial (mitochondrion) [Saccharomyces cerevisiae]
gb|AFP67254.1| **Mass:** 22228 **Score:** 40 **Matches:** 1(0) **Sequences:** 1(0)
cytochrome c oxidase subunit II, partial (mitochondrion) [Saccharomyces cerevisiae]
gb|AFP67255.1| **Mass:** 22242 **Score:** 40 **Matches:** 1(0) **Sequences:** 1(0)
cytochrome c oxidase subunit II, partial (mitochondrion) [Saccharomyces cerevisiae]
gb|AFP67256.1| **Mass:** 22228 **Score:** 40 **Matches:** 1(0) **Sequences:** 1(0)

cytochrome c oxidase subunit II, partial (mitochondrion) [Saccharomyces cerevisiae]
gb|AFP67257.1| **Mass:** 22228 **Score:** 40 **Matches:** 1(0) **Sequences:** 1(0)
cytochrome c oxidase subunit II, partial (mitochondrion) [Saccharomyces cerevisiae]
gb|AFP67258.1| **Mass:** 22228 **Score:** 40 **Matches:** 1(0) **Sequences:** 1(0)
cytochrome c oxidase subunit II, partial (mitochondrion) [Saccharomyces cerevisiae]
gb|AFP67259.1| **Mass:** 22228 **Score:** 40 **Matches:** 1(0) **Sequences:** 1(0)
cytochrome c oxidase subunit II, partial (mitochondrion) [Saccharomyces cerevisiae]
gb|AFP67260.1| **Mass:** 22228 **Score:** 40 **Matches:** 1(0) **Sequences:** 1(0)
cytochrome c oxidase subunit II, partial (mitochondrion) [Saccharomyces cerevisiae]
gb|AFP67261.1| **Mass:** 22228 **Score:** 40 **Matches:** 1(0) **Sequences:** 1(0)
cytochrome c oxidase subunit II, partial (mitochondrion) [Saccharomyces cerevisiae]
gb|AFP67262.1| **Mass:** 22228 **Score:** 40 **Matches:** 1(0) **Sequences:** 1(0)
cytochrome c oxidase subunit II, partial (mitochondrion) [Saccharomyces cerevisiae]
gb|AFP67263.1| **Mass:** 22228 **Score:** 40 **Matches:** 1(0) **Sequences:** 1(0)
cytochrome c oxidase subunit II, partial (mitochondrion) [Saccharomyces cerevisiae]
gb|AFP67264.1| **Mass:** 22228 **Score:** 40 **Matches:** 1(0) **Sequences:** 1(0)
cytochrome c oxidase subunit II, partial (mitochondrion) [Saccharomyces cerevisiae]
gb|AFP67265.1| **Mass:** 22228 **Score:** 40 **Matches:** 1(0) **Sequences:** 1(0)
cytochrome c oxidase subunit II, partial (mitochondrion) [Saccharomyces cerevisiae]
gb|AFP67266.1| **Mass:** 22228 **Score:** 40 **Matches:** 1(0) **Sequences:** 1(0)
cytochrome c oxidase subunit II, partial (mitochondrion) [Saccharomyces cerevisiae]
gb|AFP67267.1| **Mass:** 22228 **Score:** 40 **Matches:** 1(0) **Sequences:** 1(0)
cytochrome c oxidase subunit II, partial (mitochondrion) [Saccharomyces cerevisiae]
gb|AFP67268.1| **Mass:** 22228 **Score:** 40 **Matches:** 1(0) **Sequences:** 1(0)
cytochrome c oxidase subunit II, partial (mitochondrion) [Saccharomyces cerevisiae]
gb|AFP67269.1| **Mass:** 22228 **Score:** 40 **Matches:** 1(0) **Sequences:** 1(0)
cytochrome c oxidase subunit II, partial (mitochondrion) [Saccharomyces cerevisiae]
gb|AFP67270.1| **Mass:** 22228 **Score:** 40 **Matches:** 1(0) **Sequences:** 1(0)
cytochrome c oxidase subunit II, partial (mitochondrion) [Saccharomyces cerevisiae]
gb|AFP67271.1| **Mass:** 22228 **Score:** 40 **Matches:** 1(0) **Sequences:** 1(0)
cytochrome c oxidase subunit II, partial (mitochondrion) [Saccharomyces cerevisiae]
gb|AFP67272.1| **Mass:** 22228 **Score:** 40 **Matches:** 1(0) **Sequences:** 1(0)
cytochrome c oxidase subunit II, partial (mitochondrion) [Saccharomyces cerevisiae]
gb|AFP67273.1| **Mass:** 22228 **Score:** 40 **Matches:** 1(0) **Sequences:** 1(0)
cytochrome c oxidase subunit II, partial (mitochondrion) [Saccharomyces cerevisiae]
gb|AFP67274.1| **Mass:** 22228 **Score:** 40 **Matches:** 1(0) **Sequences:** 1(0)
cytochrome c oxidase subunit II, partial (mitochondrion) [Saccharomyces cerevisiae]
gb|AFP67275.1| **Mass:** 22228 **Score:** 40 **Matches:** 1(0) **Sequences:** 1(0)
cytochrome c oxidase subunit II, partial (mitochondrion) [Saccharomyces cerevisiae]
gb|AFP67276.1| **Mass:** 22228 **Score:** 40 **Matches:** 1(0) **Sequences:** 1(0)
cytochrome c oxidase subunit II, partial (mitochondrion) [Saccharomyces cerevisiae]
gb|AFP67277.1| **Mass:** 22228 **Score:** 40 **Matches:** 1(0) **Sequences:** 1(0)
cytochrome c oxidase subunit II, partial (mitochondrion) [Saccharomyces cerevisiae]
gb|AFP67278.1| **Mass:** 22228 **Score:** 40 **Matches:** 1(0) **Sequences:** 1(0)
cytochrome c oxidase subunit II, partial (mitochondrion) [Saccharomyces cerevisiae]
gb|AFP67279.1| **Mass:** 22228 **Score:** 40 **Matches:** 1(0) **Sequences:** 1(0)
cytochrome c oxidase subunit II, partial (mitochondrion) [Saccharomyces cerevisiae]
gb|AFP67280.1| **Mass:** 22228 **Score:** 40 **Matches:** 1(0) **Sequences:** 1(0)
cytochrome c oxidase subunit II, partial (mitochondrion) [Saccharomyces cerevisiae]
gb|AFP67281.1| **Mass:** 22228 **Score:** 40 **Matches:** 1(0) **Sequences:** 1(0)
cytochrome c oxidase subunit II, partial (mitochondrion) [Saccharomyces cerevisiae]
gb|AFP67282.1| **Mass:** 22228 **Score:** 40 **Matches:** 1(0) **Sequences:** 1(0)
cytochrome c oxidase subunit II, partial (mitochondrion) [Saccharomyces cerevisiae]
gb|AFP67283.1| **Mass:** 22228 **Score:** 40 **Matches:** 1(0) **Sequences:** 1(0)
cytochrome c oxidase subunit II, partial (mitochondrion) [Saccharomyces cerevisiae]
gb|AFP67284.1| **Mass:** 22228 **Score:** 40 **Matches:** 1(0) **Sequences:** 1(0)
cytochrome c oxidase subunit II, partial (mitochondrion) [Saccharomyces cerevisiae]
gb|AFP67285.1| **Mass:** 22285 **Score:** 40 **Matches:** 1(0) **Sequences:** 1(0)
cytochrome c oxidase subunit II, partial (mitochondrion) [Saccharomyces cerevisiae]
gb|AFP67286.1| **Mass:** 22285 **Score:** 40 **Matches:** 1(0) **Sequences:** 1(0)
cytochrome c oxidase subunit II, partial (mitochondrion) [Saccharomyces cerevisiae]
gb|AFP67287.1| **Mass:** 22285 **Score:** 40 **Matches:** 1(0) **Sequences:** 1(0)
cytochrome c oxidase subunit II, partial (mitochondrion) [Saccharomyces cerevisiae]
gb|AFP67288.1| **Mass:** 22285 **Score:** 40 **Matches:** 1(0) **Sequences:** 1(0)
cytochrome c oxidase subunit II, partial (mitochondrion) [Saccharomyces cerevisiae]
gb|AFP67289.1| **Mass:** 22285 **Score:** 40 **Matches:** 1(0) **Sequences:** 1(0)
cytochrome c oxidase subunit II, partial (mitochondrion) [Saccharomyces cerevisiae]
gb|AFP67290.1| **Mass:** 22285 **Score:** 40 **Matches:** 1(0) **Sequences:** 1(0)
cytochrome c oxidase subunit II, partial (mitochondrion) [Saccharomyces cerevisiae]
gb|AFP67291.1| **Mass:** 22285 **Score:** 40 **Matches:** 1(0) **Sequences:** 1(0)
cytochrome c oxidase subunit II, partial (mitochondrion) [Saccharomyces cerevisiae]
gb|AFP67292.1| **Mass:** 22285 **Score:** 40 **Matches:** 1(0) **Sequences:** 1(0)
cytochrome c oxidase subunit II, partial (mitochondrion) [Saccharomyces cerevisiae]
gb|AFP67293.1| **Mass:** 22285 **Score:** 40 **Matches:** 1(0) **Sequences:** 1(0)
cytochrome c oxidase subunit II, partial (mitochondrion) [Saccharomyces cerevisiae]
gb|AFP67294.1| **Mass:** 22285 **Score:** 40 **Matches:** 1(0) **Sequences:** 1(0)
cytochrome c oxidase subunit II, partial (mitochondrion) [Saccharomyces cerevisiae]
gb|AFP67295.1| **Mass:** 22285 **Score:** 40 **Matches:** 1(0) **Sequences:** 1(0)
cytochrome c oxidase subunit II, partial (mitochondrion) [Saccharomyces cerevisiae]
gb|AFP67296.1| **Mass:** 22285 **Score:** 40 **Matches:** 1(0) **Sequences:** 1(0)
cytochrome c oxidase subunit II, partial (mitochondrion) [Saccharomyces cerevisiae]
gb|AFP67297.1| **Mass:** 22285 **Score:** 40 **Matches:** 1(0) **Sequences:** 1(0)
cytochrome c oxidase subunit II, partial (mitochondrion) [Saccharomyces cerevisiae]
gb|AFP67298.1| **Mass:** 22285 **Score:** 40 **Matches:** 1(0) **Sequences:** 1(0)
cytochrome c oxidase subunit II, partial (mitochondrion) [Saccharomyces cerevisiae]
gb|AFP67299.1| **Mass:** 22285 **Score:** 40 **Matches:** 1(0) **Sequences:** 1(0)
cytochrome c oxidase subunit II, partial (mitochondrion) [Saccharomyces cerevisiae]
gb|AFP67300.1| **Mass:** 22285 **Score:** 40 **Matches:** 1(0) **Sequences:** 1(0)

cytochrome c oxidase subunit II, partial (mitochondrion) [Saccharomyces cerevisiae]
gb|AFP67301.1| **Mass:** 22285 **Score:** 40 **Matches:** 1(0) **Sequences:** 1(0)
cytochrome c oxidase subunit II, partial (mitochondrion) [Saccharomyces cerevisiae]
gb|AFP67302.1| **Mass:** 22285 **Score:** 40 **Matches:** 1(0) **Sequences:** 1(0)
cytochrome c oxidase subunit II, partial (mitochondrion) [Saccharomyces cerevisiae]
gb|AFP67303.1| **Mass:** 22285 **Score:** 40 **Matches:** 1(0) **Sequences:** 1(0)
cytochrome c oxidase subunit II, partial (mitochondrion) [Saccharomyces cerevisiae]
gb|AFP67304.1| **Mass:** 22285 **Score:** 40 **Matches:** 1(0) **Sequences:** 1(0)
cytochrome c oxidase subunit II, partial (mitochondrion) [Saccharomyces cerevisiae]
gb|AFP67305.1| **Mass:** 22285 **Score:** 40 **Matches:** 1(0) **Sequences:** 1(0)
cytochrome c oxidase subunit II, partial (mitochondrion) [Saccharomyces cerevisiae]
gb|AFP67306.1| **Mass:** 22285 **Score:** 40 **Matches:** 1(0) **Sequences:** 1(0)
cytochrome c oxidase subunit II, partial (mitochondrion) [Saccharomyces cerevisiae]
gb|AFP67307.1| **Mass:** 22285 **Score:** 40 **Matches:** 1(0) **Sequences:** 1(0)
cytochrome c oxidase subunit II, partial (mitochondrion) [Saccharomyces cerevisiae]
gb|AFP67308.1| **Mass:** 22285 **Score:** 40 **Matches:** 1(0) **Sequences:** 1(0)
cytochrome c oxidase subunit II, partial (mitochondrion) [Saccharomyces cerevisiae]
gb|AFP67309.1| **Mass:** 22285 **Score:** 40 **Matches:** 1(0) **Sequences:** 1(0)
cytochrome c oxidase subunit II, partial (mitochondrion) [Saccharomyces cerevisiae]
gb|AFP67310.1| **Mass:** 22285 **Score:** 40 **Matches:** 1(0) **Sequences:** 1(0)
cytochrome c oxidase subunit II, partial (mitochondrion) [Saccharomyces cerevisiae]
gb|AFP67311.1| **Mass:** 22285 **Score:** 40 **Matches:** 1(0) **Sequences:** 1(0)
cytochrome c oxidase subunit II, partial (mitochondrion) [Saccharomyces cerevisiae]
gb|AFP67312.1| **Mass:** 22285 **Score:** 40 **Matches:** 1(0) **Sequences:** 1(0)
cytochrome c oxidase subunit II, partial (mitochondrion) [Saccharomyces cerevisiae]
gb|AFP67313.1| **Mass:** 22285 **Score:** 40 **Matches:** 1(0) **Sequences:** 1(0)
cytochrome c oxidase subunit II, partial (mitochondrion) [Saccharomyces cerevisiae]
gb|AFP67314.1| **Mass:** 22285 **Score:** 40 **Matches:** 1(0) **Sequences:** 1(0)
cytochrome c oxidase subunit II, partial (mitochondrion) [Saccharomyces cerevisiae]
gb|AFP67315.1| **Mass:** 22285 **Score:** 40 **Matches:** 1(0) **Sequences:** 1(0)
cytochrome c oxidase subunit II, partial (mitochondrion) [Saccharomyces cerevisiae]
gb|AFP67316.1| **Mass:** 22198 **Score:** 40 **Matches:** 1(0) **Sequences:** 1(0)
cytochrome c oxidase subunit II, partial (mitochondrion) [Saccharomyces cerevisiae]
gb|AFP67317.1| **Mass:** 22285 **Score:** 40 **Matches:** 1(0) **Sequences:** 1(0)
cytochrome c oxidase subunit II, partial (mitochondrion) [Saccharomyces cerevisiae]
gb|AFP67318.1| **Mass:** 22285 **Score:** 40 **Matches:** 1(0) **Sequences:** 1(0)
cytochrome c oxidase subunit II, partial (mitochondrion) [Saccharomyces cerevisiae]
gb|AFP67319.1| **Mass:** 22288 **Score:** 40 **Matches:** 1(0) **Sequences:** 1(0)
cytochrome c oxidase subunit II, partial (mitochondrion) [Saccharomyces cerevisiae]
gb|AFP67320.1| **Mass:** 22285 **Score:** 40 **Matches:** 1(0) **Sequences:** 1(0)
cytochrome c oxidase subunit II, partial (mitochondrion) [Saccharomyces cerevisiae]
gb|AFP67321.1| **Mass:** 22285 **Score:** 40 **Matches:** 1(0) **Sequences:** 1(0)
cytochrome c oxidase subunit II, partial (mitochondrion) [Saccharomyces cerevisiae]
gb|AFP67322.1| **Mass:** 22182 **Score:** 40 **Matches:** 1(0) **Sequences:** 1(0)
cytochrome c oxidase subunit II, partial (mitochondrion) [Saccharomyces cerevisiae]
gb|AFP67323.1| **Mass:** 22285 **Score:** 40 **Matches:** 1(0) **Sequences:** 1(0)
cytochrome c oxidase subunit II, partial (mitochondrion) [Saccharomyces cerevisiae]
gb|AFP67324.1| **Mass:** 22285 **Score:** 40 **Matches:** 1(0) **Sequences:** 1(0)
cytochrome c oxidase subunit II, partial (mitochondrion) [Saccharomyces cerevisiae]
gb|AFP67325.1| **Mass:** 22285 **Score:** 40 **Matches:** 1(0) **Sequences:** 1(0)
cytochrome c oxidase subunit II, partial (mitochondrion) [Saccharomyces cerevisiae]
gb|AFP67326.1| **Mass:** 22285 **Score:** 40 **Matches:** 1(0) **Sequences:** 1(0)
cytochrome c oxidase subunit II, partial (mitochondrion) [Saccharomyces cerevisiae]
gb|AFP67327.1| **Mass:** 22285 **Score:** 40 **Matches:** 1(0) **Sequences:** 1(0)
cytochrome c oxidase subunit II, partial (mitochondrion) [Saccharomyces cerevisiae]
gb|AFP67328.1| **Mass:** 22285 **Score:** 40 **Matches:** 1(0) **Sequences:** 1(0)
cytochrome c oxidase subunit II, partial (mitochondrion) [Saccharomyces cerevisiae]
gb|AFP67329.1| **Mass:** 22285 **Score:** 40 **Matches:** 1(0) **Sequences:** 1(0)
cytochrome c oxidase subunit II, partial (mitochondrion) [Saccharomyces cerevisiae]
gb|AFP67330.1| **Mass:** 22285 **Score:** 40 **Matches:** 1(0) **Sequences:** 1(0)
cytochrome c oxidase subunit II, partial (mitochondrion) [Saccharomyces cerevisiae]
gb|AFP67331.1| **Mass:** 22228 **Score:** 40 **Matches:** 1(0) **Sequences:** 1(0)
cytochrome c oxidase subunit II, partial (mitochondrion) [Saccharomyces cerevisiae]
gb|AFP67332.1| **Mass:** 22228 **Score:** 40 **Matches:** 1(0) **Sequences:** 1(0)
cytochrome c oxidase subunit II, partial (mitochondrion) [Saccharomyces cerevisiae]
gb|AFP67333.1| **Mass:** 22228 **Score:** 40 **Matches:** 1(0) **Sequences:** 1(0)
cytochrome c oxidase subunit II, partial (mitochondrion) [Saccharomyces cerevisiae]
gb|AFP67334.1| **Mass:** 22228 **Score:** 40 **Matches:** 1(0) **Sequences:** 1(0)
cytochrome c oxidase subunit II, partial (mitochondrion) [Saccharomyces cerevisiae]
gb|AFP67335.1| **Mass:** 22198 **Score:** 40 **Matches:** 1(0) **Sequences:** 1(0)
cytochrome c oxidase subunit II, partial (mitochondrion) [Saccharomyces cerevisiae]
gb|AFP67336.1| **Mass:** 22228 **Score:** 40 **Matches:** 1(0) **Sequences:** 1(0)
cytochrome c oxidase subunit II, partial (mitochondrion) [Saccharomyces cerevisiae]
gb|AFP67337.1| **Mass:** 22228 **Score:** 40 **Matches:** 1(0) **Sequences:** 1(0)
cytochrome c oxidase subunit II, partial (mitochondrion) [Saccharomyces cerevisiae]
gb|AFP67338.1| **Mass:** 22228 **Score:** 40 **Matches:** 1(0) **Sequences:** 1(0)
cytochrome c oxidase subunit II, partial (mitochondrion) [Saccharomyces cerevisiae]
gb|AFP67339.1| **Mass:** 22228 **Score:** 40 **Matches:** 1(0) **Sequences:** 1(0)
cytochrome c oxidase subunit II, partial (mitochondrion) [Saccharomyces cerevisiae]
gb|AFP67340.1| **Mass:** 22228 **Score:** 40 **Matches:** 1(0) **Sequences:** 1(0)
cytochrome c oxidase subunit II, partial (mitochondrion) [Saccharomyces cerevisiae]
gb|AFP67341.1| **Mass:** 22228 **Score:** 40 **Matches:** 1(0) **Sequences:** 1(0)
cytochrome c oxidase subunit II, partial (mitochondrion) [Saccharomyces cerevisiae]
gb|AFP67342.1| **Mass:** 22228 **Score:** 40 **Matches:** 1(0) **Sequences:** 1(0)
cytochrome c oxidase subunit II, partial (mitochondrion) [Saccharomyces cerevisiae]
gb|AFP67343.1| **Mass:** 22228 **Score:** 40 **Matches:** 1(0) **Sequences:** 1(0)
cytochrome c oxidase subunit II, partial (mitochondrion) [Saccharomyces cerevisiae]
gb|AFP67344.1| **Mass:** 22286 **Score:** 40 **Matches:** 1(0) **Sequences:** 1(0)

cytochrome c oxidase subunit II, partial (mitochondrion) [Saccharomyces cerevisiae]
gb|AFP67345.1| **Mass:** 22286 **Score:** 40 **Matches:** 1(0) **Sequences:** 1(0)
cytochrome c oxidase subunit II, partial (mitochondrion) [Saccharomyces cerevisiae]
gb|AFP67414.1| **Mass:** 22285 **Score:** 40 **Matches:** 1(0) **Sequences:** 1(0)
cytochrome c oxidase subunit II, partial (mitochondrion) [Saccharomyces cerevisiae x Saccharomyces kudriavzevii]
gb|AFP67420.1| **Mass:** 22228 **Score:** 40 **Matches:** 1(0) **Sequences:** 1(0)
cytochrome c oxidase subunit II, partial (mitochondrion) [Saccharomyces cerevisiae]
gb|AFP67421.1| **Mass:** 22285 **Score:** 40 **Matches:** 1(0) **Sequences:** 1(0)
cytochrome c oxidase subunit II, partial (mitochondrion) [Saccharomyces cerevisiae]
gb|AAA67528.1| **Mass:** 28777 **Score:** 40 **Matches:** 1(0) **Sequences:** 1(0)
oxiI (mitochondrion) [Saccharomyces cerevisiae]
gb|AAO67715.1| **Mass:** 23157 **Score:** 40 **Matches:** 1(0) **Sequences:** 1(0)
cytochrome c oxidase subunit II, partial (mitochondrion) [Saccharomyces cerevisiae]
gb|AHY74306.1| **Mass:** 28834 **Score:** 40 **Matches:** 1(0) **Sequences:** 1(0)
cytochrome c oxidase subunit 2 (mitochondrion) [Saccharomyces cerevisiae YJM993]
gb|AFK74338.1| **Mass:** 24476 **Score:** 40 **Matches:** 1(0) **Sequences:** 1(0)
cytochrome c oxidase subunit II, partial (mitochondrion) [Saccharomyces cerevisiae]
gb|AFK74339.1| **Mass:** 24476 **Score:** 40 **Matches:** 1(0) **Sequences:** 1(0)
cytochrome c oxidase subunit II, partial (mitochondrion) [Saccharomyces cerevisiae]
gb|AFK74340.1| **Mass:** 24476 **Score:** 40 **Matches:** 1(0) **Sequences:** 1(0)
cytochrome c oxidase subunit II, partial (mitochondrion) [Saccharomyces cerevisiae]
gb|AFK74341.1| **Mass:** 24476 **Score:** 40 **Matches:** 1(0) **Sequences:** 1(0)
cytochrome c oxidase subunit II, partial (mitochondrion) [Saccharomyces cerevisiae]
gb|AFK74342.1| **Mass:** 24419 **Score:** 40 **Matches:** 1(0) **Sequences:** 1(0)
cytochrome c oxidase subunit II, partial (mitochondrion) [Saccharomyces cerevisiae]
gb|AFK74343.1| **Mass:** 24476 **Score:** 40 **Matches:** 1(0) **Sequences:** 1(0)
cytochrome c oxidase subunit II, partial (mitochondrion) [Saccharomyces cerevisiae]
gb|AFK74344.1| **Mass:** 24476 **Score:** 40 **Matches:** 1(0) **Sequences:** 1(0)
cytochrome c oxidase subunit II, partial (mitochondrion) [Saccharomyces cerevisiae]
gb|AFK74345.1| **Mass:** 24476 **Score:** 40 **Matches:** 1(0) **Sequences:** 1(0)
cytochrome c oxidase subunit II, partial (mitochondrion) [Saccharomyces cerevisiae]
gb|AFK74346.1| **Mass:** 24476 **Score:** 40 **Matches:** 1(0) **Sequences:** 1(0)
cytochrome c oxidase subunit II, partial (mitochondrion) [Saccharomyces cerevisiae]
gb|AFK74347.1| **Mass:** 24476 **Score:** 40 **Matches:** 1(0) **Sequences:** 1(0)
cytochrome c oxidase subunit II, partial (mitochondrion) [Saccharomyces cerevisiae]
gb|AFK74348.1| **Mass:** 24476 **Score:** 40 **Matches:** 1(0) **Sequences:** 1(0)
cytochrome c oxidase subunit II, partial (mitochondrion) [Saccharomyces cerevisiae]
gb|AFK74349.1| **Mass:** 24476 **Score:** 40 **Matches:** 1(0) **Sequences:** 1(0)
cytochrome c oxidase subunit II, partial (mitochondrion) [Saccharomyces cerevisiae]
gb|AFK74350.1| **Mass:** 24476 **Score:** 40 **Matches:** 1(0) **Sequences:** 1(0)
cytochrome c oxidase subunit II, partial (mitochondrion) [Saccharomyces cerevisiae]
gb|AFK74351.1| **Mass:** 24476 **Score:** 40 **Matches:** 1(0) **Sequences:** 1(0)
cytochrome c oxidase subunit II, partial (mitochondrion) [Saccharomyces cerevisiae]
gb|AFK74352.1| **Mass:** 24476 **Score:** 40 **Matches:** 1(0) **Sequences:** 1(0)
cytochrome c oxidase subunit II, partial (mitochondrion) [Saccharomyces cerevisiae]
gb|AFK74353.1| **Mass:** 24476 **Score:** 40 **Matches:** 1(0) **Sequences:** 1(0)
cytochrome c oxidase subunit II, partial (mitochondrion) [Saccharomyces cerevisiae]
gb|AFK74354.1| **Mass:** 24476 **Score:** 40 **Matches:** 1(0) **Sequences:** 1(0)
cytochrome c oxidase subunit II, partial (mitochondrion) [Saccharomyces cerevisiae]
gb|AFK74355.1| **Mass:** 24476 **Score:** 40 **Matches:** 1(0) **Sequences:** 1(0)
cytochrome c oxidase subunit II, partial (mitochondrion) [Saccharomyces cerevisiae]
gb|AFK74356.1| **Mass:** 24476 **Score:** 40 **Matches:** 1(0) **Sequences:** 1(0)
cytochrome c oxidase subunit II, partial (mitochondrion) [Saccharomyces cerevisiae]
gb|AFK74357.1| **Mass:** 24476 **Score:** 40 **Matches:** 1(0) **Sequences:** 1(0)
cytochrome c oxidase subunit II, partial (mitochondrion) [Saccharomyces cerevisiae]
gb|AFK74358.1| **Mass:** 24476 **Score:** 40 **Matches:** 1(0) **Sequences:** 1(0)
cytochrome c oxidase subunit II, partial (mitochondrion) [Saccharomyces cerevisiae]
gb|AFK74359.1| **Mass:** 24476 **Score:** 40 **Matches:** 1(0) **Sequences:** 1(0)
cytochrome c oxidase subunit II, partial (mitochondrion) [Saccharomyces cerevisiae]
gb|AFK74360.1| **Mass:** 24476 **Score:** 40 **Matches:** 1(0) **Sequences:** 1(0)
cytochrome c oxidase subunit II, partial (mitochondrion) [Saccharomyces cerevisiae]
gb|AFK74361.1| **Mass:** 24476 **Score:** 40 **Matches:** 1(0) **Sequences:** 1(0)
cytochrome c oxidase subunit II, partial (mitochondrion) [Saccharomyces cerevisiae]
gb|AFK74362.1| **Mass:** 24476 **Score:** 40 **Matches:** 1(0) **Sequences:** 1(0)
cytochrome c oxidase subunit II, partial (mitochondrion) [Saccharomyces cerevisiae]
gb|AFK74363.1| **Mass:** 24419 **Score:** 40 **Matches:** 1(0) **Sequences:** 1(0)
cytochrome c oxidase subunit II, partial (mitochondrion) [Saccharomyces cerevisiae]
gb|AFK74364.1| **Mass:** 24419 **Score:** 40 **Matches:** 1(0) **Sequences:** 1(0)
cytochrome c oxidase subunit II, partial (mitochondrion) [Saccharomyces cerevisiae]
gb|AFK74365.1| **Mass:** 24419 **Score:** 40 **Matches:** 1(0) **Sequences:** 1(0)
cytochrome c oxidase subunit II, partial (mitochondrion) [Saccharomyces cerevisiae]
gb|AFK74366.1| **Mass:** 24419 **Score:** 40 **Matches:** 1(0) **Sequences:** 1(0)
cytochrome c oxidase subunit II, partial (mitochondrion) [Saccharomyces cerevisiae]
gb|AFK74367.1| **Mass:** 24419 **Score:** 40 **Matches:** 1(0) **Sequences:** 1(0)
cytochrome c oxidase subunit II, partial (mitochondrion) [Saccharomyces cerevisiae]
gb|AFK74368.1| **Mass:** 24419 **Score:** 40 **Matches:** 1(0) **Sequences:** 1(0)
cytochrome c oxidase subunit II, partial (mitochondrion) [Saccharomyces cerevisiae]
gb|AFK74369.1| **Mass:** 24419 **Score:** 40 **Matches:** 1(0) **Sequences:** 1(0)
cytochrome c oxidase subunit II, partial (mitochondrion) [Saccharomyces cerevisiae]
gb|AFK74370.1| **Mass:** 24419 **Score:** 40 **Matches:** 1(0) **Sequences:** 1(0)
cytochrome c oxidase subunit II, partial (mitochondrion) [Saccharomyces cerevisiae]
gb|AFK74371.1| **Mass:** 24419 **Score:** 40 **Matches:** 1(0) **Sequences:** 1(0)
cytochrome c oxidase subunit II, partial (mitochondrion) [Saccharomyces cerevisiae]
gb|AFK74372.1| **Mass:** 24419 **Score:** 40 **Matches:** 1(0) **Sequences:** 1(0)
cytochrome c oxidase subunit II, partial (mitochondrion) [Saccharomyces cerevisiae]
gb|AFK74373.1| **Mass:** 24419 **Score:** 40 **Matches:** 1(0) **Sequences:** 1(0)
cytochrome c oxidase subunit II, partial (mitochondrion) [Saccharomyces cerevisiae]
gb|AFK74374.1| **Mass:** 24419 **Score:** 40 **Matches:** 1(0) **Sequences:** 1(0)

cytochrome c oxidase subunit II, partial (mitochondrion) [Saccharomyces cerevisiae]
gb|AFK74375.1| **Mass:** 24419 **Score:** 40 **Matches:** 1(0) **Sequences:** 1(0)
cytochrome c oxidase subunit II, partial (mitochondrion) [Saccharomyces cerevisiae]
gb|AFK74376.1| **Mass:** 24476 **Score:** 40 **Matches:** 1(0) **Sequences:** 1(0)
cytochrome c oxidase subunit II, partial (mitochondrion) [Saccharomyces cerevisiae]
gb|AFK74377.1| **Mass:** 24476 **Score:** 40 **Matches:** 1(0) **Sequences:** 1(0)
cytochrome c oxidase subunit II, partial (mitochondrion) [Saccharomyces cerevisiae]
gb|AFK74378.1| **Mass:** 24419 **Score:** 40 **Matches:** 1(0) **Sequences:** 1(0)
cytochrome c oxidase subunit II, partial (mitochondrion) [Saccharomyces cerevisiae]
gb|AFK74379.1| **Mass:** 24419 **Score:** 40 **Matches:** 1(0) **Sequences:** 1(0)
cytochrome c oxidase subunit II, partial (mitochondrion) [Saccharomyces cerevisiae]
gb|AFK74380.1| **Mass:** 24419 **Score:** 40 **Matches:** 1(0) **Sequences:** 1(0)
cytochrome c oxidase subunit II, partial (mitochondrion) [Saccharomyces cerevisiae]
gb|AFK74381.1| **Mass:** 24419 **Score:** 40 **Matches:** 1(0) **Sequences:** 1(0)
cytochrome c oxidase subunit II, partial (mitochondrion) [Saccharomyces cerevisiae]
gb|AFK74382.1| **Mass:** 24419 **Score:** 40 **Matches:** 1(0) **Sequences:** 1(0)
cytochrome c oxidase subunit II, partial (mitochondrion) [Saccharomyces cerevisiae]
gb|AFK74383.1| **Mass:** 24419 **Score:** 40 **Matches:** 1(0) **Sequences:** 1(0)
cytochrome c oxidase subunit II, partial (mitochondrion) [Saccharomyces cerevisiae]
gb|AFK74384.1| **Mass:** 24419 **Score:** 40 **Matches:** 1(0) **Sequences:** 1(0)
cytochrome c oxidase subunit II, partial (mitochondrion) [Saccharomyces cerevisiae]
gb|AFK74385.1| **Mass:** 24419 **Score:** 40 **Matches:** 1(0) **Sequences:** 1(0)
cytochrome c oxidase subunit II, partial (mitochondrion) [Saccharomyces cerevisiae]
gb|AFK74386.1| **Mass:** 24419 **Score:** 40 **Matches:** 1(0) **Sequences:** 1(0)
cytochrome c oxidase subunit II, partial (mitochondrion) [Saccharomyces cerevisiae]
gb|AFK74387.1| **Mass:** 24419 **Score:** 40 **Matches:** 1(0) **Sequences:** 1(0)
cytochrome c oxidase subunit II, partial (mitochondrion) [Saccharomyces cerevisiae]
gb|AFK74388.1| **Mass:** 24419 **Score:** 40 **Matches:** 1(0) **Sequences:** 1(0)
cytochrome c oxidase subunit II, partial (mitochondrion) [Saccharomyces cerevisiae]
gb|AFK74389.1| **Mass:** 24419 **Score:** 40 **Matches:** 1(0) **Sequences:** 1(0)
cytochrome c oxidase subunit II, partial (mitochondrion) [Saccharomyces cerevisiae]
gb|AFK74390.1| **Mass:** 24419 **Score:** 40 **Matches:** 1(0) **Sequences:** 1(0)
cytochrome c oxidase subunit II, partial (mitochondrion) [Saccharomyces cerevisiae]
gb|AFK74391.1| **Mass:** 24419 **Score:** 40 **Matches:** 1(0) **Sequences:** 1(0)
cytochrome c oxidase subunit II, partial (mitochondrion) [Saccharomyces cerevisiae]
gb|AFK74392.1| **Mass:** 24419 **Score:** 40 **Matches:** 1(0) **Sequences:** 1(0)
cytochrome c oxidase subunit II, partial (mitochondrion) [Saccharomyces cerevisiae]
gb|AFK74393.1| **Mass:** 24419 **Score:** 40 **Matches:** 1(0) **Sequences:** 1(0)
cytochrome c oxidase subunit II, partial (mitochondrion) [Saccharomyces cerevisiae]
gb|AFK74394.1| **Mass:** 24419 **Score:** 40 **Matches:** 1(0) **Sequences:** 1(0)
cytochrome c oxidase subunit II, partial (mitochondrion) [Saccharomyces cerevisiae]
gb|AFK74395.1| **Mass:** 24419 **Score:** 40 **Matches:** 1(0) **Sequences:** 1(0)
cytochrome c oxidase subunit II, partial (mitochondrion) [Saccharomyces cerevisiae]
gb|AFK74396.1| **Mass:** 24476 **Score:** 40 **Matches:** 1(0) **Sequences:** 1(0)
cytochrome c oxidase subunit II, partial (mitochondrion) [Saccharomyces cerevisiae]
gb|AFK74397.1| **Mass:** 24476 **Score:** 40 **Matches:** 1(0) **Sequences:** 1(0)
cytochrome c oxidase subunit II, partial (mitochondrion) [Saccharomyces cerevisiae]
gb|AFK74398.1| **Mass:** 24476 **Score:** 40 **Matches:** 1(0) **Sequences:** 1(0)
cytochrome c oxidase subunit II, partial (mitochondrion) [Saccharomyces cerevisiae]
gb|AFK74399.1| **Mass:** 24419 **Score:** 40 **Matches:** 1(0) **Sequences:** 1(0)
cytochrome c oxidase subunit II, partial (mitochondrion) [Saccharomyces cerevisiae]
gb|AFK74400.1| **Mass:** 24419 **Score:** 40 **Matches:** 1(0) **Sequences:** 1(0)
cytochrome c oxidase subunit II, partial (mitochondrion) [Saccharomyces cerevisiae]
gb|AFK74401.1| **Mass:** 24476 **Score:** 40 **Matches:** 1(0) **Sequences:** 1(0)
cytochrome c oxidase subunit II, partial (mitochondrion) [Saccharomyces cerevisiae]
gb|AFK74402.1| **Mass:** 24476 **Score:** 40 **Matches:** 1(0) **Sequences:** 1(0)
cytochrome c oxidase subunit II, partial (mitochondrion) [Saccharomyces cerevisiae]
gb|AFK74403.1| **Mass:** 24476 **Score:** 40 **Matches:** 1(0) **Sequences:** 1(0)
cytochrome c oxidase subunit II, partial (mitochondrion) [Saccharomyces cerevisiae]
gb|AFK74404.1| **Mass:** 24476 **Score:** 40 **Matches:** 1(0) **Sequences:** 1(0)
cytochrome c oxidase subunit II, partial (mitochondrion) [Saccharomyces cerevisiae]
gb|AFK74405.1| **Mass:** 24476 **Score:** 40 **Matches:** 1(0) **Sequences:** 1(0)
cytochrome c oxidase subunit II, partial (mitochondrion) [Saccharomyces cerevisiae]
gb|AFK74406.1| **Mass:** 24476 **Score:** 40 **Matches:** 1(0) **Sequences:** 1(0)
cytochrome c oxidase subunit II, partial (mitochondrion) [Saccharomyces cerevisiae]
gb|AKJ77231.1| **Mass:** 28834 **Score:** 40 **Matches:** 1(0) **Sequences:** 1(0)
cytchrome c oxidase subunit 2 (mitochondrion) [Saccharomyces cerevisiae]
gb|AKJ77239.1| **Mass:** 28834 **Score:** 40 **Matches:** 1(0) **Sequences:** 1(0)
cytchrome c oxidase subunit 2 (mitochondrion) [Saccharomyces cerevisiae]
dbj|BAK78971.1| **Mass:** 28777 **Score:** 40 **Matches:** 1(0) **Sequences:** 1(0)
K7_Cox2p (mitochondrion) [Saccharomyces cerevisiae Kyokai no. 7]
emb|CAI79374.1| **Mass:** 22230 **Score:** 40 **Matches:** 1(0) **Sequences:** 1(0)
cytochrome c oxidase, subunit II, partial (mitochondrion) [Saccharomyces sp. W27]
emb|CAI79375.1| **Mass:** 22230 **Score:** 40 **Matches:** 1(0) **Sequences:** 1(0)
cytochrome c oxidase, subunit II, partial (mitochondrion) [Saccharomyces sp. W46]
emb|CAI79376.1| **Mass:** 22230 **Score:** 40 **Matches:** 1(0) **Sequences:** 1(0)
cytochrome c oxidase, subunit II, partial (mitochondrion) [Saccharomyces sp. 441]
emb|CAI79377.1| **Mass:** 22230 **Score:** 40 **Matches:** 1(0) **Sequences:** 1(0)
cytochrome c oxidase, subunit II, partial (mitochondrion) [Saccharomyces sp. 172]
emb|CAI79378.1| **Mass:** 22230 **Score:** 40 **Matches:** 1(0) **Sequences:** 1(0)
cytochrome c oxidase, subunit II, partial (mitochondrion) [Saccharomyces sp. 319]
emb|CAI79379.1| **Mass:** 22230 **Score:** 40 **Matches:** 1(0) **Sequences:** 1(0)
cytochrome c oxidase, subunit II, partial (mitochondrion) [Saccharomyces sp. 126]
emb|CAI79380.1| **Mass:** 22230 **Score:** 40 **Matches:** 1(0) **Sequences:** 1(0)
cytochrome c oxidase, subunit II, partial (mitochondrion) [Saccharomyces sp. SPG14-91]
emb|CAI79381.1| **Mass:** 22230 **Score:** 40 **Matches:** 1(0) **Sequences:** 1(0)
cytochrome c oxidase, subunit II, partial (mitochondrion) [Saccharomyces sp. SPG16-91]
emb|CAI79383.1| **Mass:** 22285 **Score:** 40 **Matches:** 1(0) **Sequences:** 1(0)

cytochrome c oxidase, subunit II, partial (mitochondrion) [Saccharomyces sp. CBS 8615]
emb|CAI79384.1| **Mass:** 22230 **Score:** 40 **Matches:** 1(0) **Sequences:** 1(0)
cytochrome c oxidase, subunit II, partial (mitochondrion) [Saccharomyces sp. CBS 2834]
emb|CAI79385.1| **Mass:** 22271 **Score:** 40 **Matches:** 1(0) **Sequences:** 1(0)
cytochrome c oxidase, subunit II, partial (mitochondrion) [Saccharomyces sp. CBS 8614]
emb|CAC80025.1| **Mass:** 20953 **Score:** 40 **Matches:** 1(0) **Sequences:** 1(0)
cytochrome oxidase subunit II, partial (mitochondrion) [Saccharomyces cerevisiae]
gb|AKL82692.1| **Mass:** 28777 **Score:** 40 **Matches:** 1(0) **Sequences:** 1(0)
cytochrome c oxidase subunit 2 (mitochondrion) [Saccharomyces cerevisiae]
gb|AKL82736.1| **Mass:** 28834 **Score:** 40 **Matches:** 1(0) **Sequences:** 1(0)
cytochrome c oxidase subunit 2 (mitochondrion) [Saccharomyces cerevisiae]
gb|AKL82767.1| **Mass:** 28834 **Score:** 40 **Matches:** 1(0) **Sequences:** 1(0)
cytochrome c oxidase subunit 2 (mitochondrion) [Saccharomyces cerevisiae]
gb|AKL82783.1| **Mass:** 28777 **Score:** 40 **Matches:** 1(0) **Sequences:** 1(0)
cytochrome c oxidase subunit 2 (mitochondrion) [Saccharomyces cerevisiae]
gb|AKL82797.1| **Mass:** 28834 **Score:** 40 **Matches:** 1(0) **Sequences:** 1(0)
cytochrome c oxidase subunit 2 (mitochondrion) [Saccharomyces cerevisiae]
gb|AKL82875.1| **Mass:** 28834 **Score:** 40 **Matches:** 1(0) **Sequences:** 1(0)
cytochrome c oxidase subunit 2 (mitochondrion) [Saccharomyces cerevisiae]
gb|AKL82892.1| **Mass:** 28834 **Score:** 40 **Matches:** 1(0) **Sequences:** 1(0)
cytochrome c oxidase subunit 2 (mitochondrion) [Saccharomyces cerevisiae]
gb|AKL82954.1| **Mass:** 28834 **Score:** 40 **Matches:** 1(0) **Sequences:** 1(0)
cytochrome c oxidase subunit 2 (mitochondrion) [Saccharomyces cerevisiae]
gb|AKL82986.1| **Mass:** 28834 **Score:** 40 **Matches:** 1(0) **Sequences:** 1(0)
cytochrome c oxidase subunit 2 (mitochondrion) [Saccharomyces cerevisiae]
gb|AKL83030.1| **Mass:** 28834 **Score:** 40 **Matches:** 1(0) **Sequences:** 1(0)
cytochrome c oxidase subunit 2 (mitochondrion) [Saccharomyces cerevisiae]
gb|AKL83047.1| **Mass:** 28834 **Score:** 40 **Matches:** 1(0) **Sequences:** 1(0)
cytochrome c oxidase subunit 2 (mitochondrion) [Saccharomyces cerevisiae]
gb|AKL83078.1| **Mass:** 28777 **Score:** 40 **Matches:** 1(0) **Sequences:** 1(0)
cytochrome c oxidase subunit 2 (mitochondrion) [Saccharomyces cerevisiae]
gb|AKL83094.1| **Mass:** 28834 **Score:** 40 **Matches:** 1(0) **Sequences:** 1(0)
cytochrome c oxidase subunit 2 (mitochondrion) [Saccharomyces cerevisiae]
gb|AKL83114.1| **Mass:** 28834 **Score:** 40 **Matches:** 1(0) **Sequences:** 1(0)
cytochrome c oxidase subunit 2 (mitochondrion) [Saccharomyces cerevisiae]
gb|AAM83122.1| **Mass:** 22228 **Score:** 40 **Matches:** 1(0) **Sequences:** 1(0)
cytochrome oxidase subunit II, partial (mitochondrion) [Saccharomyces cerevisiae]
gb|AAM83123.1| **Mass:** 18841 **Score:** 40 **Matches:** 1(0) **Sequences:** 1(0)
cytochrome oxidase subunit II, partial (mitochondrion) [Saccharomyces cerevisiae]
gb|AAM83124.1| **Mass:** 22285 **Score:** 40 **Matches:** 1(0) **Sequences:** 1(0)
cytochrome oxidase subunit II, partial (mitochondrion) [Saccharomyces cerevisiae]
gb|AAM83125.1| **Mass:** 22228 **Score:** 40 **Matches:** 1(0) **Sequences:** 1(0)
cytochrome oxidase subunit II, partial (mitochondrion) [Saccharomyces cerevisiae]
emb|CAI84895.1| **Mass:** 22271 **Score:** 40 **Matches:** 1(0) **Sequences:** 1(0)
cytochrome c oxidase, subunit II, partial (mitochondrion) [Saccharomyces sp. CECT 1388]
emb|CAI84896.1| **Mass:** 22271 **Score:** 40 **Matches:** 1(0) **Sequences:** 1(0)
cytochrome c oxidase, subunit II, partial (mitochondrion) [Saccharomyces sp. CECT 1990]
emb|CAI84898.1| **Mass:** 22271 **Score:** 40 **Matches:** 1(0) **Sequences:** 1(0)
cytochrome c oxidase, subunit II, partial (mitochondrion) [Saccharomyces sp. CECT 11002]
emb|CAI84899.1| **Mass:** 22230 **Score:** 40 **Matches:** 1(0) **Sequences:** 1(0)
cytochrome c oxidase, subunit II, partial (mitochondrion) [Saccharomyces sp. CECT 11003]
emb|CAI84900.1| **Mass:** 22230 **Score:** 40 **Matches:** 1(0) **Sequences:** 1(0)
cytochrome c oxidase, subunit II, partial (mitochondrion) [Saccharomyces sp. CECT 11004]
emb|CAI84901.1| **Mass:** 22271 **Score:** 40 **Matches:** 1(0) **Sequences:** 1(0)
cytochrome c oxidase, subunit II, partial (mitochondrion) [Saccharomyces sp. CECT 11011]
emb|CAY86125.1| **Mass:** 24006 **Score:** 40 **Matches:** 1(0) **Sequences:** 1(0)
cytochrome oxidase subunit 2, partial (mitochondrion) [Saccharomyces cerevisiae]
emb|CAY86126.1| **Mass:** 22882 **Score:** 40 **Matches:** 1(0) **Sequences:** 1(0)
cytochrome oxidase subunit 2, partial (mitochondrion) [Saccharomyces cerevisiae]
emb|CAY86127.1| **Mass:** 23093 **Score:** 40 **Matches:** 1(0) **Sequences:** 1(0)
cytochrome oxidase subunit 2, partial (mitochondrion) [Saccharomyces cerevisiae]
emb|CAY86128.1| **Mass:** 22711 **Score:** 40 **Matches:** 1(0) **Sequences:** 1(0)
cytochrome oxidase subunit 2, partial (mitochondrion) [Saccharomyces cerevisiae]
emb|CAY86129.1| **Mass:** 24006 **Score:** 40 **Matches:** 1(0) **Sequences:** 1(0)
cytochrome oxidase subunit 2, partial (mitochondrion) [Saccharomyces cerevisiae]
emb|CAY86130.1| **Mass:** 23893 **Score:** 40 **Matches:** 1(0) **Sequences:** 1(0)
cytochrome oxidase subunit 2, partial (mitochondrion) [Saccharomyces cerevisiae]
gb|AEA95743.1| **Mass:** 21449 **Score:** 40 **Matches:** 1(0) **Sequences:** 1(0)
cytochrome c oxidase subunit II, partial (mitochondrion) [Saccharomyces cerevisiae x Saccharomyces kudriavzevii]
gb|AEA95744.1| **Mass:** 21449 **Score:** 40 **Matches:** 1(0) **Sequences:** 1(0)
cytochrome c oxidase subunit II, partial (mitochondrion) [Saccharomyces cerevisiae x Saccharomyces kudriavzevii]
gb|AEA95745.1| **Mass:** 21490 **Score:** 40 **Matches:** 1(0) **Sequences:** 1(0)
cytochrome c oxidase subunit II, partial (mitochondrion) [Saccharomyces cerevisiae x Saccharomyces kudriavzevii]
gb|AEA95746.1| **Mass:** 21504 **Score:** 40 **Matches:** 1(0) **Sequences:** 1(0)
cytochrome c oxidase subunit II, partial (mitochondrion) [Saccharomyces cerevisiae x Saccharomyces kudriavzevii]
gb|AEA95747.1| **Mass:** 21447 **Score:** 40 **Matches:** 1(0) **Sequences:** 1(0)
cytochrome c oxidase subunit II, partial (mitochondrion) [Saccharomyces cerevisiae x Saccharomyces kudriavzevii]
gb|AEA95748.1| **Mass:** 21490 **Score:** 40 **Matches:** 1(0) **Sequences:** 1(0)
cytochrome c oxidase subunit II, partial (mitochondrion) [Saccharomyces cerevisiae x Saccharomyces kudriavzevii]
gb|AEA95749.1| **Mass:** 21437 **Score:** 40 **Matches:** 1(0) **Sequences:** 1(0)
cytochrome c oxidase subunit II, partial (mitochondrion) [Saccharomyces kudriavzevii]
gb|AEA95750.1| **Mass:** 21449 **Score:** 40 **Matches:** 1(0) **Sequences:** 1(0)
cytochrome c oxidase subunit II, partial (mitochondrion) [Saccharomyces kudriavzevii]
gb|AEA95751.1| **Mass:** 21449 **Score:** 40 **Matches:** 1(0) **Sequences:** 1(0)
cytochrome c oxidase subunit II, partial (mitochondrion) [Saccharomyces cerevisiae x Saccharomyces kudriavzevii]
gb|AEA95752.1| **Mass:** 21449 **Score:** 40 **Matches:** 1(0) **Sequences:** 1(0)
cytochrome c oxidase subunit II, partial (mitochondrion) [Saccharomyces cerevisiae x Saccharomyces kudriavzevii]
gb|AEA95753.1| **Mass:** 21449 **Score:** 40 **Matches:** 1(0) **Sequences:** 1(0)

cytochrome c oxidase subunit II, partial (mitochondrion) [Saccharomyces cerevisiae x Saccharomyces kudriavzevii]
gb|AEA95754.1| **Mass:** 21449 **Score:** 40 **Matches:** 1(0) **Sequences:** 1(0)
cytochrome c oxidase subunit II, partial (mitochondrion) [Saccharomyces cerevisiae x Saccharomyces kudriavzevii]
gb|AIZ98897.1| **Mass:** 28777 **Score:** 40 **Matches:** 1(0) **Sequences:** 1(0)
cytochrome c oxidase subunit 2 (mitochondrion) [Saccharomyces cerevisiae S288c]

---

10. gb|AJS00152.1| **Mass:** 15069 **Score:** 40 **Matches:** 1(0) **Sequences:** 1(0)
Cox13p [Saccharomyces cerevisiae YJM1433]
☐ Check to include this hit in error tolerant search or archive report

Query	Observed	Mr(expt)	Mr(calc)	ppm	Miss	Score	Expect	Rank	Unique	Peptide
7	1245.7526	1244.7454	1244.6666	63.3	0	40	0.18	1	U	K.TLFWNPVVNR.H

Proteins matching the same set of peptides:
gb|AJS00643.1| **Mass:** 15069 **Score:** 40 **Matches:** 1(0) **Sequences:** 1(0)
Cox13p [Saccharomyces cerevisiae YJM1434]
gb|AJS01143.1| **Mass:** 15069 **Score:** 40 **Matches:** 1(0) **Sequences:** 1(0)
Cox13p [Saccharomyces cerevisiae YJM1439]
gb|AKB01211.1| **Mass:** 15069 **Score:** 40 **Matches:** 1(0) **Sequences:** 1(0)
Cox13p [Saccharomyces cerevisiae YJM1304]
gb|AJS01628.1| **Mass:** 15069 **Score:** 40 **Matches:** 1(0) **Sequences:** 1(0)
Cox13p [Saccharomyces cerevisiae YJM1443]
gb|AJS02122.1| **Mass:** 15069 **Score:** 40 **Matches:** 1(0) **Sequences:** 1(0)
Cox13p [Saccharomyces cerevisiae YJM1444]
gb|AJS02606.1| **Mass:** 15069 **Score:** 40 **Matches:** 1(0) **Sequences:** 1(0)
Cox13p [Saccharomyces cerevisiae YJM1447]
gb|AJS03096.1| **Mass:** 15069 **Score:** 40 **Matches:** 1(0) **Sequences:** 1(0)
Cox13p [Saccharomyces cerevisiae YJM1450]
gb|AJS03599.1| **Mass:** 15069 **Score:** 40 **Matches:** 1(0) **Sequences:** 1(0)
Cox13p [Saccharomyces cerevisiae YJM1460]
gb|AJS04052.1| **Mass:** 15069 **Score:** 40 **Matches:** 1(0) **Sequences:** 1(0)
Cox13p [Saccharomyces cerevisiae YJM1463]
gb|AJS04552.1| **Mass:** 15069 **Score:** 40 **Matches:** 1(0) **Sequences:** 1(0)
Cox13p [Saccharomyces cerevisiae YJM1477]
gb|AJS05027.1| **Mass:** 15069 **Score:** 40 **Matches:** 1(0) **Sequences:** 1(0)
Cox13p [Saccharomyces cerevisiae YJM1478]
gb|AJS05527.1| **Mass:** 15069 **Score:** 40 **Matches:** 1(0) **Sequences:** 1(0)
Cox13p [Saccharomyces cerevisiae YJM1479]
gb|AJS06024.1| **Mass:** 15069 **Score:** 40 **Matches:** 1(0) **Sequences:** 1(0)
Cox13p [Saccharomyces cerevisiae YJM1526]
gb|AJS06514.1| **Mass:** 15069 **Score:** 40 **Matches:** 1(0) **Sequences:** 1(0)
Cox13p [Saccharomyces cerevisiae YJM1527]
gb|AJS07016.1| **Mass:** 15069 **Score:** 40 **Matches:** 1(0) **Sequences:** 1(0)
Cox13p [Saccharomyces cerevisiae YJM1549]
gb|AJS07517.1| **Mass:** 15069 **Score:** 40 **Matches:** 1(0) **Sequences:** 1(0)
Cox13p [Saccharomyces cerevisiae YJM1573]
gb|AJS08017.1| **Mass:** 15069 **Score:** 40 **Matches:** 1(0) **Sequences:** 1(0)
Cox13p [Saccharomyces cerevisiae YJM1574]
gb|AJS08514.1| **Mass:** 15069 **Score:** 40 **Matches:** 1(0) **Sequences:** 1(0)
Cox13p [Saccharomyces cerevisiae YJM1592]
gb|AJS08939.1| **Mass:** 15069 **Score:** 40 **Matches:** 1(0) **Sequences:** 1(0)
Cox13p [Saccharomyces cerevisiae YJM1615]
gb|AJS19173.1| **Mass:** 15069 **Score:** 40 **Matches:** 1(0) **Sequences:** 1(0)
Cox13p [Saccharomyces cerevisiae YJM1273]
gb|AJS19675.1| **Mass:** 15069 **Score:** 40 **Matches:** 1(0) **Sequences:** 1(0)
Cox13p [Saccharomyces cerevisiae YJM1307]
gb|AJS20161.1| **Mass:** 15069 **Score:** 40 **Matches:** 1(0) **Sequences:** 1(0)
Cox13p [Saccharomyces cerevisiae YJM1311]
gb|AJS20659.1| **Mass:** 15069 **Score:** 40 **Matches:** 1(0) **Sequences:** 1(0)
Cox13p [Saccharomyces cerevisiae YJM1326]
gb|AJS21160.1| **Mass:** 15069 **Score:** 40 **Matches:** 1(0) **Sequences:** 1(0)
Cox13p [Saccharomyces cerevisiae YJM1332]
gb|AJS21664.1| **Mass:** 15069 **Score:** 40 **Matches:** 1(0) **Sequences:** 1(0)
Cox13p [Saccharomyces cerevisiae YJM1336]
gb|AJS22157.1| **Mass:** 15069 **Score:** 40 **Matches:** 1(0) **Sequences:** 1(0)
Cox13p [Saccharomyces cerevisiae YJM1338]
gb|AJS22656.1| **Mass:** 15069 **Score:** 40 **Matches:** 1(0) **Sequences:** 1(0)
Cox13p [Saccharomyces cerevisiae YJM1341]
gb|AJS23155.1| **Mass:** 15069 **Score:** 40 **Matches:** 1(0) **Sequences:** 1(0)
Cox13p [Saccharomyces cerevisiae YJM1342]
emb|CCD23304.1| **Mass:** 15053 **Score:** 40 **Matches:** 1(0) **Sequences:** 1(0)
hypothetical protein NDAI_0B02690 [Naumovozyma dairenensis CBS 421]
gb|AJS23652.1| **Mass:** 15069 **Score:** 40 **Matches:** 1(0) **Sequences:** 1(0)
Cox13p [Saccharomyces cerevisiae YJM1355]
gb|AAB23912.1| **Mass:** 1245 **Score:** 40 **Matches:** 1(0) **Sequences:** 1(0)
cytochrome c oxidase VIa subunit homolog [Saccharomyces cerevisiae, JHRY1-2 alpha, Peptide Partial, 10 aa, segment 5 of 5]
gb|AJS24155.1| **Mass:** 15069 **Score:** 40 **Matches:** 1(0) **Sequences:** 1(0)
Cox13p [Saccharomyces cerevisiae YJM1356]
gb|AJS24649.1| **Mass:** 15069 **Score:** 40 **Matches:** 1(0) **Sequences:** 1(0)
Cox13p [Saccharomyces cerevisiae YJM1381]
gb|AJS25152.1| **Mass:** 15069 **Score:** 40 **Matches:** 1(0) **Sequences:** 1(0)
Cox13p [Saccharomyces cerevisiae YJM1383]
gb|AJS25648.1| **Mass:** 15069 **Score:** 40 **Matches:** 1(0) **Sequences:** 1(0)
Cox13p [Saccharomyces cerevisiae YJM1385]
gb|AJS26148.1| **Mass:** 15069 **Score:** 40 **Matches:** 1(0) **Sequences:** 1(0)
Cox13p [Saccharomyces cerevisiae YJM1386]
gb|AJS26639.1| **Mass:** 15069 **Score:** 40 **Matches:** 1(0) **Sequences:** 1(0)

Cox13p [Saccharomyces cerevisiae YJM1387]
gb|AJS27129.1| **Mass:** 15069 **Score:** 40 **Matches:** 1(0) **Sequences:** 1(0)
Cox13p [Saccharomyces cerevisiae YJM1388]
gb|AJS27621.1| **Mass:** 15069 **Score:** 40 **Matches:** 1(0) **Sequences:** 1(0)
Cox13p [Saccharomyces cerevisiae YJM1389]
gb|AJS28113.1| **Mass:** 15069 **Score:** 40 **Matches:** 1(0) **Sequences:** 1(0)
Cox13p [Saccharomyces cerevisiae YJM1399]
gb|AJS28600.1| **Mass:** 15069 **Score:** 40 **Matches:** 1(0) **Sequences:** 1(0)
Cox13p [Saccharomyces cerevisiae YJM1400]
gb|AJS29097.1| **Mass:** 15068 **Score:** 40 **Matches:** 1(0) **Sequences:** 1(0)
Cox13p [Saccharomyces cerevisiae YJM1401]
gb|AJS29593.1| **Mass:** 15069 **Score:** 40 **Matches:** 1(0) **Sequences:** 1(0)
Cox13p [Saccharomyces cerevisiae YJM1402]
gb|AJP38616.1| **Mass:** 15069 **Score:** 40 **Matches:** 1(0) **Sequences:** 1(0)
Cox13p [Saccharomyces cerevisiae YJM1078]
emb|CAA51479.1| **Mass:** 15069 **Score:** 40 **Matches:** 1(0) **Sequences:** 1(0)
cytochrome-c oxidase [Saccharomyces cerevisiae]
gb|AAS53567.1| **Mass:** 14584 **Score:** 40 **Matches:** 1(0) **Sequences:** 1(0)
AFR196Cp [Eremothecium gossypii ATCC 10895]
gb|AAS56815.1| **Mass:** 15069 **Score:** 40 **Matches:** 1(0) **Sequences:** 1(0)
YGL191W [Saccharomyces cerevisiae]
emb|CCF57287.1| **Mass:** 15832 **Score:** 40 **Matches:** 1(0) **Sequences:** 1(0)
hypothetical protein KAFR_0C02940 [Kazachstania africana CBS 2517]
emb|CEP60536.1| **Mass:** 14400 **Score:** 40 **Matches:** 1(0) **Sequences:** 1(0)
LALA0S01e13146g1_1 [Lachancea lanzarotensis]
emb|CAA62953.1| **Mass:** 15069 **Score:** 40 **Matches:** 1(0) **Sequences:** 1(0)
cytochrome-C oxidase chain VIa precursor [Saccharomyces cerevisiae]
emb|CCE64244.1| **Mass:** 14791 **Score:** 40 **Matches:** 1(0) **Sequences:** 1(0)
hypothetical protein TPHA_0H00340 [Tetrapisispora phaffii CBS 4417]
gb|AJR75939.1| **Mass:** 15069 **Score:** 40 **Matches:** 1(0) **Sequences:** 1(0)
Cox13p [Saccharomyces cerevisiae YJM189]
gb|AJR76437.1| **Mass:** 15069 **Score:** 40 **Matches:** 1(0) **Sequences:** 1(0)
Cox13p [Saccharomyces cerevisiae YJM193]
gb|AJR76935.1| **Mass:** 15069 **Score:** 40 **Matches:** 1(0) **Sequences:** 1(0)
Cox13p [Saccharomyces cerevisiae YJM195]
gb|AJR77434.1| **Mass:** 15069 **Score:** 40 **Matches:** 1(0) **Sequences:** 1(0)
Cox13p [Saccharomyces cerevisiae YJM244]
gb|AJR77937.1| **Mass:** 15069 **Score:** 40 **Matches:** 1(0) **Sequences:** 1(0)
Cox13p [Saccharomyces cerevisiae YJM248]
gb|AJR78422.1| **Mass:** 15069 **Score:** 40 **Matches:** 1(0) **Sequences:** 1(0)
Cox13p [Saccharomyces cerevisiae YJM270]
gb|AJR78917.1| **Mass:** 15069 **Score:** 40 **Matches:** 1(0) **Sequences:** 1(0)
Cox13p [Saccharomyces cerevisiae YJM271]
gb|AHY79199.1| **Mass:** 15069 **Score:** 40 **Matches:** 1(0) **Sequences:** 1(0)
Cox13p [Saccharomyces cerevisiae YJM993]
gb|AJR79417.1| **Mass:** 15069 **Score:** 40 **Matches:** 1(0) **Sequences:** 1(0)
Cox13p [Saccharomyces cerevisiae YJM320]
emb|CAY79576.1| **Mass:** 15069 **Score:** 40 **Matches:** 1(0) **Sequences:** 1(0)
Cox13p [Saccharomyces cerevisiae EC1118]
gb|AJR79919.1| **Mass:** 15069 **Score:** 40 **Matches:** 1(0) **Sequences:** 1(0)
Cox13p [Saccharomyces cerevisiae YJM326]
gb|AJR80379.1| **Mass:** 15069 **Score:** 40 **Matches:** 1(0) **Sequences:** 1(0)
Cox13p [Saccharomyces cerevisiae YJM428]
gb|AJR80881.1| **Mass:** 15069 **Score:** 40 **Matches:** 1(0) **Sequences:** 1(0)
Cox13p [Saccharomyces cerevisiae YJM450]
gb|AJR81386.1| **Mass:** 15069 **Score:** 40 **Matches:** 1(0) **Sequences:** 1(0)
Cox13p [Saccharomyces cerevisiae YJM451]
gb|AJR81881.1| **Mass:** 15069 **Score:** 40 **Matches:** 1(0) **Sequences:** 1(0)
Cox13p [Saccharomyces cerevisiae YJM453]
gb|AJR82374.1| **Mass:** 15069 **Score:** 40 **Matches:** 1(0) **Sequences:** 1(0)
Cox13p [Saccharomyces cerevisiae YJM456]
gb|AJR82874.1| **Mass:** 15069 **Score:** 40 **Matches:** 1(0) **Sequences:** 1(0)
Cox13p [Saccharomyces cerevisiae YJM470]
gb|AJR83367.1| **Mass:** 15069 **Score:** 40 **Matches:** 1(0) **Sequences:** 1(0)
Cox13p [Saccharomyces cerevisiae YJM541]
gb|AJR83868.1| **Mass:** 15069 **Score:** 40 **Matches:** 1(0) **Sequences:** 1(0)
Cox13p [Saccharomyces cerevisiae YJM554]
gb|AJR84369.1| **Mass:** 15069 **Score:** 40 **Matches:** 1(0) **Sequences:** 1(0)
Cox13p [Saccharomyces cerevisiae YJM555]
gb|AJR84871.1| **Mass:** 15069 **Score:** 40 **Matches:** 1(0) **Sequences:** 1(0)
Cox13p [Saccharomyces cerevisiae YJM627]
gb|AJR85367.1| **Mass:** 15069 **Score:** 40 **Matches:** 1(0) **Sequences:** 1(0)
Cox13p [Saccharomyces cerevisiae YJM681]
gb|AJR85858.1| **Mass:** 15069 **Score:** 40 **Matches:** 1(0) **Sequences:** 1(0)
Cox13p [Saccharomyces cerevisiae YJM682]
gb|AJR86356.1| **Mass:** 15069 **Score:** 40 **Matches:** 1(0) **Sequences:** 1(0)
Cox13p [Saccharomyces cerevisiae YJM683]
gb|AJR86856.1| **Mass:** 15069 **Score:** 40 **Matches:** 1(0) **Sequences:** 1(0)
Cox13p [Saccharomyces cerevisiae YJM689]
gb|AJR87356.1| **Mass:** 15069 **Score:** 40 **Matches:** 1(0) **Sequences:** 1(0)
Cox13p [Saccharomyces cerevisiae YJM693]
gb|AJR87810.1| **Mass:** 15069 **Score:** 40 **Matches:** 1(0) **Sequences:** 1(0)
Cox13p [Saccharomyces cerevisiae YJM969]
gb|AJR88307.1| **Mass:** 15069 **Score:** 40 **Matches:** 1(0) **Sequences:** 1(0)
Cox13p [Saccharomyces cerevisiae YJM972]
gb|AJR88808.1| **Mass:** 15069 **Score:** 40 **Matches:** 1(0) **Sequences:** 1(0)
Cox13p [Saccharomyces cerevisiae YJM975]
gb|AJR89280.1| **Mass:** 15069 **Score:** 40 **Matches:** 1(0) **Sequences:** 1(0)

Cox13p [Saccharomyces cerevisiae YJM978]
gb|AJR89779.1| **Mass:** 15069 **Score:** 40 **Matches:** 1(0) **Sequences:** 1(0)
Cox13p [Saccharomyces cerevisiae YJM981]
gb|AJR90281.1| **Mass:** 15069 **Score:** 40 **Matches:** 1(0) **Sequences:** 1(0)
Cox13p [Saccharomyces cerevisiae YJM984]
gb|AJR90767.1| **Mass:** 15069 **Score:** 40 **Matches:** 1(0) **Sequences:** 1(0)
Cox13p [Saccharomyces cerevisiae YJM987]
gb|AJR91263.1| **Mass:** 15069 **Score:** 40 **Matches:** 1(0) **Sequences:** 1(0)
Cox13p [Saccharomyces cerevisiae YJM990]
gb|AJR91764.1| **Mass:** 15069 **Score:** 40 **Matches:** 1(0) **Sequences:** 1(0)
Cox13p [Saccharomyces cerevisiae YJM996]
gb|AJR92264.1| **Mass:** 15069 **Score:** 40 **Matches:** 1(0) **Sequences:** 1(0)
Cox13p [Saccharomyces cerevisiae YJM1083]
gb|AJR92758.1| **Mass:** 15069 **Score:** 40 **Matches:** 1(0) **Sequences:** 1(0)
Cox13p [Saccharomyces cerevisiae YJM1129]
gb|AJR93264.1| **Mass:** 15069 **Score:** 40 **Matches:** 1(0) **Sequences:** 1(0)
Cox13p [Saccharomyces cerevisiae YJM1133]
gb|AJR93764.1| **Mass:** 15069 **Score:** 40 **Matches:** 1(0) **Sequences:** 1(0)
Cox13p [Saccharomyces cerevisiae YJM1190]
gb|AJR94260.1| **Mass:** 15069 **Score:** 40 **Matches:** 1(0) **Sequences:** 1(0)
Cox13p [Saccharomyces cerevisiae YJM1199]
gb|AJR94764.1| **Mass:** 15069 **Score:** 40 **Matches:** 1(0) **Sequences:** 1(0)
Cox13p [Saccharomyces cerevisiae YJM1202]
gb|AJR95261.1| **Mass:** 15069 **Score:** 40 **Matches:** 1(0) **Sequences:** 1(0)
Cox13p [Saccharomyces cerevisiae YJM1208]
gb|AJR95731.1| **Mass:** 15069 **Score:** 40 **Matches:** 1(0) **Sequences:** 1(0)
Cox13p [Saccharomyces cerevisiae YJM1242]
gb|AJR96175.1| **Mass:** 15069 **Score:** 40 **Matches:** 1(0) **Sequences:** 1(0)
Cox13p [Saccharomyces cerevisiae YJM1244]
gb|AJR96663.1| **Mass:** 15069 **Score:** 40 **Matches:** 1(0) **Sequences:** 1(0)
Cox13p [Saccharomyces cerevisiae YJM1248]
emb|CAA96903.1| **Mass:** 15069 **Score:** 40 **Matches:** 1(0) **Sequences:** 1(0)
COX13 [Saccharomyces cerevisiae]
gb|AJR97155.1| **Mass:** 15069 **Score:** 40 **Matches:** 1(0) **Sequences:** 1(0)
Cox13p [Saccharomyces cerevisiae YJM1250]
gb|AJR97654.1| **Mass:** 15069 **Score:** 40 **Matches:** 1(0) **Sequences:** 1(0)
Cox13p [Saccharomyces cerevisiae YJM1252]
gb|AEY97880.1| **Mass:** 14584 **Score:** 40 **Matches:** 1(0) **Sequences:** 1(0)
FAFR196Cp [Eremothecium gossypii FDAG1]
gb|AJR98154.1| **Mass:** 15069 **Score:** 40 **Matches:** 1(0) **Sequences:** 1(0)
Cox13p [Saccharomyces cerevisiae YJM1415]
gb|AJR98660.1| **Mass:** 15069 **Score:** 40 **Matches:** 1(0) **Sequences:** 1(0)
Cox13p [Saccharomyces cerevisiae YJM1417]
gb|AJR99152.1| **Mass:** 15069 **Score:** 40 **Matches:** 1(0) **Sequences:** 1(0)
Cox13p [Saccharomyces cerevisiae YJM1418]
gb|AJR99650.1| **Mass:** 15069 **Score:** 40 **Matches:** 1(0) **Sequences:** 1(0)
Cox13p [Saccharomyces cerevisiae YJM1419]

---

11. gb|AJT00070.1| **Mass:** 6928 **Score:** 30 **Matches:** 1(0) **Sequences:** 1(0)
Cox7p [Saccharomyces cerevisiae YJM1573]

☐ Check to include this hit in error tolerant search or archive report

Query	Observed	Mr(expt)	Mr(calc)	ppm	Miss	Score	Expect	Rank	Unique	Peptide
29	1548.9292	1547.9219	1547.8249	62.7	0	30	1.2	1	U	K.IFQSSTKPLWWR.H

Proteins matching the same set of peptides:
gb|AJT00513.1| **Mass:** 6928 **Score:** 30 **Matches:** 1(0) **Sequences:** 1(0)
Cox7p [Saccharomyces cerevisiae YJM1574]
gb|AJT00948.1| **Mass:** 6928 **Score:** 30 **Matches:** 1(0) **Sequences:** 1(0)
Cox7p [Saccharomyces cerevisiae YJM1592]
gb|AJT01389.1| **Mass:** 6928 **Score:** 30 **Matches:** 1(0) **Sequences:** 1(0)
Cox7p [Saccharomyces cerevisiae YJM1615]
gb|AAA34550.1| **Mass:** 6928 **Score:** 30 **Matches:** 1(0) **Sequences:** 1(0)
cytochrome c oxidase subunit VII (EC 1.9.3.1) [Saccharomyces cerevisiae]
emb|CAA35871.1| **Mass:** 6928 **Score:** 30 **Matches:** 1(0) **Sequences:** 1(0)
unnamed protein product [Saccharomyces cerevisiae]
gb|AJP40950.1| **Mass:** 6928 **Score:** 30 **Matches:** 1(0) **Sequences:** 1(0)
Cox7p [Saccharomyces cerevisiae YJM1078]
gb|AAS56455.1| **Mass:** 6928 **Score:** 30 **Matches:** 1(0) **Sequences:** 1(0)
YMR256C [Saccharomyces cerevisiae]
emb|CCF56677.1| **Mass:** 7195 **Score:** 30 **Matches:** 1(0) **Sequences:** 1(0)
hypothetical protein KAFR_0B03810 [Kazachstania africana CBS 2517]
gb|AJS62122.1| **Mass:** 6928 **Score:** 30 **Matches:** 1(0) **Sequences:** 1(0)
Cox7p [Saccharomyces cerevisiae YJM189]
gb|AJS62557.1| **Mass:** 6932 **Score:** 30 **Matches:** 1(0) **Sequences:** 1(0)
Cox7p [Saccharomyces cerevisiae YJM193]
gb|AJS62994.1| **Mass:** 6928 **Score:** 30 **Matches:** 1(0) **Sequences:** 1(0)
Cox7p [Saccharomyces cerevisiae YJM195]
gb|AJS63432.1| **Mass:** 6928 **Score:** 30 **Matches:** 1(0) **Sequences:** 1(0)
Cox7p [Saccharomyces cerevisiae YJM244]
gb|AJS63866.1| **Mass:** 6928 **Score:** 30 **Matches:** 1(0) **Sequences:** 1(0)
Cox7p [Saccharomyces cerevisiae YJM248]
gb|AJS64304.1| **Mass:** 6928 **Score:** 30 **Matches:** 1(0) **Sequences:** 1(0)
Cox7p [Saccharomyces cerevisiae YJM270]
gb|AJS64739.1| **Mass:** 6928 **Score:** 30 **Matches:** 1(0) **Sequences:** 1(0)
Cox7p [Saccharomyces cerevisiae YJM271]
gb|AJS65174.1| **Mass:** 6928 **Score:** 30 **Matches:** 1(0) **Sequences:** 1(0)

Cox7p [Saccharomyces cerevisiae YJM320]
gb|AJS65610.1| **Mass:** 6928 **Score:** 30 **Matches:** 1(0) **Sequences:** 1(0)
Cox7p [Saccharomyces cerevisiae YJM326]
gb|AJS66044.1| **Mass:** 6928 **Score:** 30 **Matches:** 1(0) **Sequences:** 1(0)
Cox7p [Saccharomyces cerevisiae YJM428]
gb|AJS66480.1| **Mass:** 6928 **Score:** 30 **Matches:** 1(0) **Sequences:** 1(0)
Cox7p [Saccharomyces cerevisiae YJM450]
gb|AJS66923.1| **Mass:** 6928 **Score:** 30 **Matches:** 1(0) **Sequences:** 1(0)
Cox7p [Saccharomyces cerevisiae YJM451]
gb|AJS67358.1| **Mass:** 6928 **Score:** 30 **Matches:** 1(0) **Sequences:** 1(0)
Cox7p [Saccharomyces cerevisiae YJM453]
gb|AJS67799.1| **Mass:** 6928 **Score:** 30 **Matches:** 1(0) **Sequences:** 1(0)
Cox7p [Saccharomyces cerevisiae YJM456]
gb|AJS68240.1| **Mass:** 6928 **Score:** 30 **Matches:** 1(0) **Sequences:** 1(0)
Cox7p [Saccharomyces cerevisiae YJM470]
gb|AJS68675.1| **Mass:** 6928 **Score:** 30 **Matches:** 1(0) **Sequences:** 1(0)
Cox7p [Saccharomyces cerevisiae YJM541]
gb|AJS69112.1| **Mass:** 6928 **Score:** 30 **Matches:** 1(0) **Sequences:** 1(0)
Cox7p [Saccharomyces cerevisiae YJM554]
gb|AJS69551.1| **Mass:** 6928 **Score:** 30 **Matches:** 1(0) **Sequences:** 1(0)
Cox7p [Saccharomyces cerevisiae YJM555]
gb|AJS69988.1| **Mass:** 6928 **Score:** 30 **Matches:** 1(0) **Sequences:** 1(0)
Cox7p [Saccharomyces cerevisiae YJM627]
gb|AJS70426.1| **Mass:** 6928 **Score:** 30 **Matches:** 1(0) **Sequences:** 1(0)
Cox7p [Saccharomyces cerevisiae YJM681]
gb|AJS70868.1| **Mass:** 6928 **Score:** 30 **Matches:** 1(0) **Sequences:** 1(0)
Cox7p [Saccharomyces cerevisiae YJM682]
gb|AJS71303.1| **Mass:** 6928 **Score:** 30 **Matches:** 1(0) **Sequences:** 1(0)
Cox7p [Saccharomyces cerevisiae YJM683]
gb|AJS71734.1| **Mass:** 6928 **Score:** 30 **Matches:** 1(0) **Sequences:** 1(0)
Cox7p [Saccharomyces cerevisiae YJM689]
emb|CCK72130.1| **Mass:** 6913 **Score:** 30 **Matches:** 1(0) **Sequences:** 1(0)
hypothetical protein KNAG_0J00470 [Kazachstania naganishii CBS 8797]
gb|AJS72175.1| **Mass:** 6928 **Score:** 30 **Matches:** 1(0) **Sequences:** 1(0)
Cox7p [Saccharomyces cerevisiae YJM693]
gb|AJS72611.1| **Mass:** 6928 **Score:** 30 **Matches:** 1(0) **Sequences:** 1(0)
Cox7p [Saccharomyces cerevisiae YJM969]
gb|AJS73049.1| **Mass:** 6928 **Score:** 30 **Matches:** 1(0) **Sequences:** 1(0)
Cox7p [Saccharomyces cerevisiae YJM972]
gb|AJS73484.1| **Mass:** 6928 **Score:** 30 **Matches:** 1(0) **Sequences:** 1(0)
Cox7p [Saccharomyces cerevisiae YJM975]
gb|AJS73922.1| **Mass:** 6928 **Score:** 30 **Matches:** 1(0) **Sequences:** 1(0)
Cox7p [Saccharomyces cerevisiae YJM978]
gb|AJS74364.1| **Mass:** 6928 **Score:** 30 **Matches:** 1(0) **Sequences:** 1(0)
Cox7p [Saccharomyces cerevisiae YJM981]
gb|AJS74788.1| **Mass:** 6928 **Score:** 30 **Matches:** 1(0) **Sequences:** 1(0)
Cox7p [Saccharomyces cerevisiae YJM984]
gb|AJS75229.1| **Mass:** 6928 **Score:** 30 **Matches:** 1(0) **Sequences:** 1(0)
Cox7p [Saccharomyces cerevisiae YJM987]
gb|AJS75668.1| **Mass:** 6928 **Score:** 30 **Matches:** 1(0) **Sequences:** 1(0)
Cox7p [Saccharomyces cerevisiae YJM990]
gb|AJS76110.1| **Mass:** 6928 **Score:** 30 **Matches:** 1(0) **Sequences:** 1(0)
Cox7p [Saccharomyces cerevisiae YJM996]
gb|AJS76547.1| **Mass:** 6928 **Score:** 30 **Matches:** 1(0) **Sequences:** 1(0)
Cox7p [Saccharomyces cerevisiae YJM1083]
gb|AHY76711.1| **Mass:** 6928 **Score:** 30 **Matches:** 1(0) **Sequences:** 1(0)
Cox7p [Saccharomyces cerevisiae YJM993]
gb|AJS76986.1| **Mass:** 6928 **Score:** 30 **Matches:** 1(0) **Sequences:** 1(0)
Cox7p [Saccharomyces cerevisiae YJM1129]
gb|AJS77425.1| **Mass:** 6928 **Score:** 30 **Matches:** 1(0) **Sequences:** 1(0)
Cox7p [Saccharomyces cerevisiae YJM1133]
gb|AJS77860.1| **Mass:** 6928 **Score:** 30 **Matches:** 1(0) **Sequences:** 1(0)
Cox7p [Saccharomyces cerevisiae YJM1190]
gb|AJS78299.1| **Mass:** 6928 **Score:** 30 **Matches:** 1(0) **Sequences:** 1(0)
Cox7p [Saccharomyces cerevisiae YJM1199]
gb|AJS78723.1| **Mass:** 6928 **Score:** 30 **Matches:** 1(0) **Sequences:** 1(0)
Cox7p [Saccharomyces cerevisiae YJM1202]
gb|AJS79160.1| **Mass:** 6928 **Score:** 30 **Matches:** 1(0) **Sequences:** 1(0)
Cox7p [Saccharomyces cerevisiae YJM1208]
gb|AJS79599.1| **Mass:** 6928 **Score:** 30 **Matches:** 1(0) **Sequences:** 1(0)
Cox7p [Saccharomyces cerevisiae YJM1242]
gb|AJS80037.1| **Mass:** 6928 **Score:** 30 **Matches:** 1(0) **Sequences:** 1(0)
Cox7p [Saccharomyces cerevisiae YJM1244]
gb|AJS80473.1| **Mass:** 6928 **Score:** 30 **Matches:** 1(0) **Sequences:** 1(0)
Cox7p [Saccharomyces cerevisiae YJM1248]
gb|AJS80902.1| **Mass:** 6928 **Score:** 30 **Matches:** 1(0) **Sequences:** 1(0)
Cox7p [Saccharomyces cerevisiae YJM1250]
gb|AJS81336.1| **Mass:** 6928 **Score:** 30 **Matches:** 1(0) **Sequences:** 1(0)
Cox7p [Saccharomyces cerevisiae YJM1252]
gb|AJS81774.1| **Mass:** 6928 **Score:** 30 **Matches:** 1(0) **Sequences:** 1(0)
Cox7p [Saccharomyces cerevisiae YJM1273]
emb|CAY82088.1| **Mass:** 6928 **Score:** 30 **Matches:** 1(0) **Sequences:** 1(0)
Cox7p [Saccharomyces cerevisiae EC1118]
gb|AJS82215.1| **Mass:** 6928 **Score:** 30 **Matches:** 1(0) **Sequences:** 1(0)
Cox7p [Saccharomyces cerevisiae YJM1304]
gb|AJS82643.1| **Mass:** 6928 **Score:** 30 **Matches:** 1(0) **Sequences:** 1(0)
Cox7p [Saccharomyces cerevisiae YJM1307]
gb|AJS83076.1| **Mass:** 6928 **Score:** 30 **Matches:** 1(0) **Sequences:** 1(0)

Cox7p [Saccharomyces cerevisiae YJM1311]
gb|AJS83511.1| **Mass:** 6928 **Score:** 30 **Matches:** 1(0) **Sequences:** 1(0)
Cox7p [Saccharomyces cerevisiae YJM1326]
gb|AJS83949.1| **Mass:** 6928 **Score:** 30 **Matches:** 1(0) **Sequences:** 1(0)
Cox7p [Saccharomyces cerevisiae YJM1332]
gb|AJS84386.1| **Mass:** 6928 **Score:** 30 **Matches:** 1(0) **Sequences:** 1(0)
Cox7p [Saccharomyces cerevisiae YJM1336]
gb|AJS84808.1| **Mass:** 6928 **Score:** 30 **Matches:** 1(0) **Sequences:** 1(0)
Cox7p [Saccharomyces cerevisiae YJM1338]
gb|AJS85252.1| **Mass:** 6928 **Score:** 30 **Matches:** 1(0) **Sequences:** 1(0)
Cox7p [Saccharomyces cerevisiae YJM1341]
gb|AJS85691.1| **Mass:** 6928 **Score:** 30 **Matches:** 1(0) **Sequences:** 1(0)
Cox7p [Saccharomyces cerevisiae YJM1342]
gb|AJS86128.1| **Mass:** 6928 **Score:** 30 **Matches:** 1(0) **Sequences:** 1(0)
Cox7p [Saccharomyces cerevisiae YJM1355]
gb|AJS86566.1| **Mass:** 6928 **Score:** 30 **Matches:** 1(0) **Sequences:** 1(0)
Cox7p [Saccharomyces cerevisiae YJM1356]
gb|AJS87000.1| **Mass:** 6928 **Score:** 30 **Matches:** 1(0) **Sequences:** 1(0)
Cox7p [Saccharomyces cerevisiae YJM1381]
gb|AJS87437.1| **Mass:** 6928 **Score:** 30 **Matches:** 1(0) **Sequences:** 1(0)
Cox7p [Saccharomyces cerevisiae YJM1383]
gb|AJS87874.1| **Mass:** 6928 **Score:** 30 **Matches:** 1(0) **Sequences:** 1(0)
Cox7p [Saccharomyces cerevisiae YJM1385]
gb|AJS88313.1| **Mass:** 6928 **Score:** 30 **Matches:** 1(0) **Sequences:** 1(0)
Cox7p [Saccharomyces cerevisiae YJM1386]
emb|CAA88583.1| **Mass:** 6928 **Score:** 30 **Matches:** 1(0) **Sequences:** 1(0)
Cox7p [Saccharomyces cerevisiae]
gb|AJS88741.1| **Mass:** 6928 **Score:** 30 **Matches:** 1(0) **Sequences:** 1(0)
Cox7p [Saccharomyces cerevisiae YJM1387]
gb|AJS89181.1| **Mass:** 6928 **Score:** 30 **Matches:** 1(0) **Sequences:** 1(0)
Cox7p [Saccharomyces cerevisiae YJM1388]
gb|AJS89619.1| **Mass:** 6928 **Score:** 30 **Matches:** 1(0) **Sequences:** 1(0)
Cox7p [Saccharomyces cerevisiae YJM1389]
gb|AJS90057.1| **Mass:** 6928 **Score:** 30 **Matches:** 1(0) **Sequences:** 1(0)
Cox7p [Saccharomyces cerevisiae YJM1399]
emb|CCE90219.1| **Mass:** 6876 **Score:** 30 **Matches:** 1(0) **Sequences:** 1(0)
hypothetical protein TDEL_0B00900 [Torulaspora delbrueckii]
gb|AJS90490.1| **Mass:** 6928 **Score:** 30 **Matches:** 1(0) **Sequences:** 1(0)
Cox7p [Saccharomyces cerevisiae YJM1400]
gb|AJS90924.1| **Mass:** 6928 **Score:** 30 **Matches:** 1(0) **Sequences:** 1(0)
Cox7p [Saccharomyces cerevisiae YJM1401]
gb|AJS91360.1| **Mass:** 6928 **Score:** 30 **Matches:** 1(0) **Sequences:** 1(0)
Cox7p [Saccharomyces cerevisiae YJM1402]
gb|AJS91803.1| **Mass:** 6928 **Score:** 30 **Matches:** 1(0) **Sequences:** 1(0)
Cox7p [Saccharomyces cerevisiae YJM1415]
gb|AJS92241.1| **Mass:** 6928 **Score:** 30 **Matches:** 1(0) **Sequences:** 1(0)
Cox7p [Saccharomyces cerevisiae YJM1417]
gb|AJS92680.1| **Mass:** 6928 **Score:** 30 **Matches:** 1(0) **Sequences:** 1(0)
Cox7p [Saccharomyces cerevisiae YJM1418]
gb|AJS93115.1| **Mass:** 6928 **Score:** 30 **Matches:** 1(0) **Sequences:** 1(0)
Cox7p [Saccharomyces cerevisiae YJM1419]
gb|AJS93554.1| **Mass:** 6928 **Score:** 30 **Matches:** 1(0) **Sequences:** 1(0)
Cox7p [Saccharomyces cerevisiae YJM1433]
gb|AJS93991.1| **Mass:** 6928 **Score:** 30 **Matches:** 1(0) **Sequences:** 1(0)
Cox7p [Saccharomyces cerevisiae YJM1434]
gb|AJS94420.1| **Mass:** 6928 **Score:** 30 **Matches:** 1(0) **Sequences:** 1(0)
Cox7p [Saccharomyces cerevisiae YJM1439]
gb|AJS94847.1| **Mass:** 6928 **Score:** 30 **Matches:** 1(0) **Sequences:** 1(0)
Cox7p [Saccharomyces cerevisiae YJM1443]
gb|AJS95287.1| **Mass:** 6928 **Score:** 30 **Matches:** 1(0) **Sequences:** 1(0)
Cox7p [Saccharomyces cerevisiae YJM1444]
gb|AJS95709.1| **Mass:** 6928 **Score:** 30 **Matches:** 1(0) **Sequences:** 1(0)
Cox7p [Saccharomyces cerevisiae YJM1447]
gb|AJS96144.1| **Mass:** 6928 **Score:** 30 **Matches:** 1(0) **Sequences:** 1(0)
Cox7p [Saccharomyces cerevisiae YJM1450]
gb|AJS96581.1| **Mass:** 6928 **Score:** 30 **Matches:** 1(0) **Sequences:** 1(0)
Cox7p [Saccharomyces cerevisiae YJM1460]
gb|AJS97016.1| **Mass:** 6942 **Score:** 30 **Matches:** 1(0) **Sequences:** 1(0)
Cox7p [Saccharomyces cerevisiae YJM1463]
gb|AJS97454.1| **Mass:** 6928 **Score:** 30 **Matches:** 1(0) **Sequences:** 1(0)
Cox7p [Saccharomyces cerevisiae YJM1477]
gb|AJS97890.1| **Mass:** 6928 **Score:** 30 **Matches:** 1(0) **Sequences:** 1(0)
Cox7p [Saccharomyces cerevisiae YJM1478]
gb|AJS98325.1| **Mass:** 6928 **Score:** 30 **Matches:** 1(0) **Sequences:** 1(0)
Cox7p [Saccharomyces cerevisiae YJM1479]
gb|AJS98758.1| **Mass:** 6928 **Score:** 30 **Matches:** 1(0) **Sequences:** 1(0)
Cox7p [Saccharomyces cerevisiae YJM1526]
gb|AJS99193.1| **Mass:** 6928 **Score:** 30 **Matches:** 1(0) **Sequences:** 1(0)
Cox7p [Saccharomyces cerevisiae YJM1527]
gb|AJS99631.1| **Mass:** 6928 **Score:** 30 **Matches:** 1(0) **Sequences:** 1(0)
Cox7p [Saccharomyces cerevisiae YJM1549]

---

12. emb|CAR57987.1| **Mass:** 7055 **Score:** 30 **Matches:** 1(0) **Sequences:** 1(0)
unnamed protein product [Candida glabrata]

☐ Check to include this hit in error tolerant search or archive report

Query	Observed	Mr(expt)	Mr(calc)	ppm	Miss	Score	Expect	Rank	Unique	Peptide
29	1548.9292	1547.9219	1547.8249	62.7	0	30	1.2	1	U	K.LFQSSTKPIWWR.H

13. pdb|2HLD|A **Mass:** 54968 **Score:** 29 **Matches:** 1(0) **Sequences:** 1(0)
Chain A, Crystal Structure Of Yeast Mitochondrial F1-Atpase
Check to include this hit in error tolerant search or archive report

Query	Observed	Mr(expt)	Mr(calc)	ppm	Miss	Score	Expect	Rank	Unique	Peptide
32	1563.9829	1562.9756	1562.8781	62.4	0	29	0.79	1	U	R.TGNIVDVPVGPGLLGR.V

Proteins matching the same set of peptides:
pdb|2HLD|B **Mass:** 54968 **Score:** 29 **Matches:** 1(0) **Sequences:** 1(0)
Chain B, Crystal Structure Of Yeast Mitochondrial F1-Atpase
pdb|2HLD|C **Mass:** 54968 **Score:** 29 **Matches:** 1(0) **Sequences:** 1(0)
Chain C, Crystal Structure Of Yeast Mitochondrial F1-Atpase
pdb|2HLD|J **Mass:** 54968 **Score:** 29 **Matches:** 1(0) **Sequences:** 1(0)
Chain J, Crystal Structure Of Yeast Mitochondrial F1-Atpase
pdb|2HLD|K **Mass:** 54968 **Score:** 29 **Matches:** 1(0) **Sequences:** 1(0)
Chain K, Crystal Structure Of Yeast Mitochondrial F1-Atpase
pdb|2HLD|L **Mass:** 54968 **Score:** 29 **Matches:** 1(0) **Sequences:** 1(0)
Chain L, Crystal Structure Of Yeast Mitochondrial F1-Atpase
pdb|2HLD|S **Mass:** 54968 **Score:** 29 **Matches:** 1(0) **Sequences:** 1(0)
Chain S, Crystal Structure Of Yeast Mitochondrial F1-Atpase
pdb|2HLD|T **Mass:** 54968 **Score:** 29 **Matches:** 1(0) **Sequences:** 1(0)
Chain T, Crystal Structure Of Yeast Mitochondrial F1-Atpase
pdb|2HLD|U **Mass:** 54968 **Score:** 29 **Matches:** 1(0) **Sequences:** 1(0)
Chain U, Crystal Structure Of Yeast Mitochondrial F1-Atpase
pdb|2WPD|A **Mass:** 54968 **Score:** 29 **Matches:** 1(0) **Sequences:** 1(0)
Chain A, The Mg.Adp Inhibited State Of The Yeast F1c10 Atp Synthase
pdb|2WPD|B **Mass:** 54968 **Score:** 29 **Matches:** 1(0) **Sequences:** 1(0)
Chain B, The Mg.Adp Inhibited State Of The Yeast F1c10 Atp Synthase
pdb|2WPD|C **Mass:** 54968 **Score:** 29 **Matches:** 1(0) **Sequences:** 1(0)
Chain C, The Mg.Adp Inhibited State Of The Yeast F1c10 Atp Synthase
pdb|2XOK|A **Mass:** 58629 **Score:** 29 **Matches:** 1(0) **Sequences:** 1(0)
Chain A, Refined Structure Of Yeast F1c10 Atpase Complex To 3 A Resolution
pdb|2XOK|B **Mass:** 58629 **Score:** 29 **Matches:** 1(0) **Sequences:** 1(0)
Chain B, Refined Structure Of Yeast F1c10 Atpase Complex To 3 A Resolution
pdb|2XOK|C **Mass:** 58629 **Score:** 29 **Matches:** 1(0) **Sequences:** 1(0)
Chain C, Refined Structure Of Yeast F1c10 Atpase Complex To 3 A Resolution
pdb|3FKS|A **Mass:** 54968 **Score:** 29 **Matches:** 1(0) **Sequences:** 1(0)
Chain A, Yeast F1 Atpase In The Absence Of Bound Nucleotides
pdb|3FKS|B **Mass:** 54968 **Score:** 29 **Matches:** 1(0) **Sequences:** 1(0)
Chain B, Yeast F1 Atpase In The Absence Of Bound Nucleotides
pdb|3FKS|C **Mass:** 54968 **Score:** 29 **Matches:** 1(0) **Sequences:** 1(0)
Chain C, Yeast F1 Atpase In The Absence Of Bound Nucleotides
pdb|3FKS|J **Mass:** 54968 **Score:** 29 **Matches:** 1(0) **Sequences:** 1(0)
Chain J, Yeast F1 Atpase In The Absence Of Bound Nucleotides
pdb|3FKS|K **Mass:** 54968 **Score:** 29 **Matches:** 1(0) **Sequences:** 1(0)
Chain K, Yeast F1 Atpase In The Absence Of Bound Nucleotides
pdb|3FKS|L **Mass:** 54968 **Score:** 29 **Matches:** 1(0) **Sequences:** 1(0)
Chain L, Yeast F1 Atpase In The Absence Of Bound Nucleotides
pdb|3FKS|S **Mass:** 54968 **Score:** 29 **Matches:** 1(0) **Sequences:** 1(0)
Chain S, Yeast F1 Atpase In The Absence Of Bound Nucleotides
pdb|3FKS|T **Mass:** 54968 **Score:** 29 **Matches:** 1(0) **Sequences:** 1(0)
Chain T, Yeast F1 Atpase In The Absence Of Bound Nucleotides
pdb|3FKS|U **Mass:** 54968 **Score:** 29 **Matches:** 1(0) **Sequences:** 1(0)
Chain U, Yeast F1 Atpase In The Absence Of Bound Nucleotides
pdb|3OE7|A **Mass:** 54968 **Score:** 29 **Matches:** 1(0) **Sequences:** 1(0)
Chain A, Structure Of Four Mutant Forms Of Yeast F1 Atpase: Gamma-I270t
pdb|3OE7|B **Mass:** 54968 **Score:** 29 **Matches:** 1(0) **Sequences:** 1(0)
Chain B, Structure Of Four Mutant Forms Of Yeast F1 Atpase: Gamma-I270t
pdb|3OE7|C **Mass:** 54968 **Score:** 29 **Matches:** 1(0) **Sequences:** 1(0)
Chain C, Structure Of Four Mutant Forms Of Yeast F1 Atpase: Gamma-I270t
pdb|3OE7|J **Mass:** 54968 **Score:** 29 **Matches:** 1(0) **Sequences:** 1(0)
Chain J, Structure Of Four Mutant Forms Of Yeast F1 Atpase: Gamma-I270t
pdb|3OE7|K **Mass:** 54968 **Score:** 29 **Matches:** 1(0) **Sequences:** 1(0)
Chain K, Structure Of Four Mutant Forms Of Yeast F1 Atpase: Gamma-I270t
pdb|3OE7|L **Mass:** 54968 **Score:** 29 **Matches:** 1(0) **Sequences:** 1(0)
Chain L, Structure Of Four Mutant Forms Of Yeast F1 Atpase: Gamma-I270t
pdb|3OE7|S **Mass:** 54968 **Score:** 29 **Matches:** 1(0) **Sequences:** 1(0)
Chain S, Structure Of Four Mutant Forms Of Yeast F1 Atpase: Gamma-I270t
pdb|3OE7|T **Mass:** 54968 **Score:** 29 **Matches:** 1(0) **Sequences:** 1(0)
Chain T, Structure Of Four Mutant Forms Of Yeast F1 Atpase: Gamma-I270t
pdb|3OE7|U **Mass:** 54968 **Score:** 29 **Matches:** 1(0) **Sequences:** 1(0)
Chain U, Structure Of Four Mutant Forms Of Yeast F1 Atpase: Gamma-I270t
pdb|3OEE|A **Mass:** 54908 **Score:** 29 **Matches:** 1(0) **Sequences:** 1(0)
Chain A, Structure Of Four Mutant Forms Of Yeast F1 Atpase: Alpha-F405s
pdb|3OEE|B **Mass:** 54908 **Score:** 29 **Matches:** 1(0) **Sequences:** 1(0)
Chain B, Structure Of Four Mutant Forms Of Yeast F1 Atpase: Alpha-F405s
pdb|3OEE|C **Mass:** 54908 **Score:** 29 **Matches:** 1(0) **Sequences:** 1(0)
Chain C, Structure Of Four Mutant Forms Of Yeast F1 Atpase: Alpha-F405s
pdb|3OEE|J **Mass:** 54908 **Score:** 29 **Matches:** 1(0) **Sequences:** 1(0)
Chain J, Structure Of Four Mutant Forms Of Yeast F1 Atpase: Alpha-F405s
pdb|3OEE|K **Mass:** 54908 **Score:** 29 **Matches:** 1(0) **Sequences:** 1(0)
Chain K, Structure Of Four Mutant Forms Of Yeast F1 Atpase: Alpha-F405s
pdb|3OEE|L **Mass:** 54908 **Score:** 29 **Matches:** 1(0) **Sequences:** 1(0)
Chain L, Structure Of Four Mutant Forms Of Yeast F1 Atpase: Alpha-F405s
pdb|3OEE|S **Mass:** 54908 **Score:** 29 **Matches:** 1(0) **Sequences:** 1(0)
Chain S, Structure Of Four Mutant Forms Of Yeast F1 Atpase: Alpha-F405s

pdb|3OEE|T **Mass:** 54908 **Score:** 29 **Matches:** 1(0) **Sequences:** 1(0)
Chain T, Structure Of Four Mutant Forms Of Yeast F1 Atpase: Alpha-F405s
pdb|3OEE|U **Mass:** 54908 **Score:** 29 **Matches:** 1(0) **Sequences:** 1(0)
Chain U, Structure Of Four Mutant Forms Of Yeast F1 Atpase: Alpha-F405s
pdb|3OEH|A **Mass:** 54968 **Score:** 29 **Matches:** 1(0) **Sequences:** 1(0)
Chain A, Structure Of Four Mutant Forms Of Yeast F1 Atpase: Beta-V279f
pdb|3OEH|B **Mass:** 54968 **Score:** 29 **Matches:** 1(0) **Sequences:** 1(0)
Chain B, Structure Of Four Mutant Forms Of Yeast F1 Atpase: Beta-V279f
pdb|3OEH|C **Mass:** 54968 **Score:** 29 **Matches:** 1(0) **Sequences:** 1(0)
Chain C, Structure Of Four Mutant Forms Of Yeast F1 Atpase: Beta-V279f
pdb|3OEH|J **Mass:** 54968 **Score:** 29 **Matches:** 1(0) **Sequences:** 1(0)
Chain J, Structure Of Four Mutant Forms Of Yeast F1 Atpase: Beta-V279f
pdb|3OEH|K **Mass:** 54968 **Score:** 29 **Matches:** 1(0) **Sequences:** 1(0)
Chain K, Structure Of Four Mutant Forms Of Yeast F1 Atpase: Beta-V279f
pdb|3OEH|L **Mass:** 54968 **Score:** 29 **Matches:** 1(0) **Sequences:** 1(0)
Chain L, Structure Of Four Mutant Forms Of Yeast F1 Atpase: Beta-V279f
pdb|3OEH|S **Mass:** 54968 **Score:** 29 **Matches:** 1(0) **Sequences:** 1(0)
Chain S, Structure Of Four Mutant Forms Of Yeast F1 Atpase: Beta-V279f
pdb|3OEH|T **Mass:** 54968 **Score:** 29 **Matches:** 1(0) **Sequences:** 1(0)
Chain T, Structure Of Four Mutant Forms Of Yeast F1 Atpase: Beta-V279f
pdb|3OEH|U **Mass:** 54968 **Score:** 29 **Matches:** 1(0) **Sequences:** 1(0)
Chain U, Structure Of Four Mutant Forms Of Yeast F1 Atpase: Beta-V279f
pdb|3OFN|A **Mass:** 54967 **Score:** 29 **Matches:** 1(0) **Sequences:** 1(0)
Chain A, Structure Of Four Mutant Forms Of Yeast F1 Atpase: Alpha-N67i
pdb|3OFN|B **Mass:** 54967 **Score:** 29 **Matches:** 1(0) **Sequences:** 1(0)
Chain B, Structure Of Four Mutant Forms Of Yeast F1 Atpase: Alpha-N67i
pdb|3OFN|C **Mass:** 54967 **Score:** 29 **Matches:** 1(0) **Sequences:** 1(0)
Chain C, Structure Of Four Mutant Forms Of Yeast F1 Atpase: Alpha-N67i
pdb|3OFN|J **Mass:** 54967 **Score:** 29 **Matches:** 1(0) **Sequences:** 1(0)
Chain J, Structure Of Four Mutant Forms Of Yeast F1 Atpase: Alpha-N67i
pdb|3OFN|K **Mass:** 54967 **Score:** 29 **Matches:** 1(0) **Sequences:** 1(0)
Chain K, Structure Of Four Mutant Forms Of Yeast F1 Atpase: Alpha-N67i
pdb|3OFN|L **Mass:** 54967 **Score:** 29 **Matches:** 1(0) **Sequences:** 1(0)
Chain L, Structure Of Four Mutant Forms Of Yeast F1 Atpase: Alpha-N67i
pdb|3OFN|S **Mass:** 54967 **Score:** 29 **Matches:** 1(0) **Sequences:** 1(0)
Chain S, Structure Of Four Mutant Forms Of Yeast F1 Atpase: Alpha-N67i
pdb|3OFN|T **Mass:** 54967 **Score:** 29 **Matches:** 1(0) **Sequences:** 1(0)
Chain T, Structure Of Four Mutant Forms Of Yeast F1 Atpase: Alpha-N67i
pdb|3OFN|U **Mass:** 54967 **Score:** 29 **Matches:** 1(0) **Sequences:** 1(0)
Chain U, Structure Of Four Mutant Forms Of Yeast F1 Atpase: Alpha-N67i
pdb|3ZIA|A **Mass:** 54968 **Score:** 29 **Matches:** 1(0) **Sequences:** 1(0)
Chain A, The Structure Of F1-atpase From Saccharomyces Cerevisiae Inhibited By Its Regulatory Protein If1
pdb|3ZIA|B **Mass:** 54968 **Score:** 29 **Matches:** 1(0) **Sequences:** 1(0)
Chain B, The Structure Of F1-atpase From Saccharomyces Cerevisiae Inhibited By Its Regulatory Protein If1
pdb|3ZIA|C **Mass:** 54968 **Score:** 29 **Matches:** 1(0) **Sequences:** 1(0)
Chain C, The Structure Of F1-atpase From Saccharomyces Cerevisiae Inhibited By Its Regulatory Protein If1
pdb|3ZIA|K **Mass:** 54968 **Score:** 29 **Matches:** 1(0) **Sequences:** 1(0)
Chain K, The Structure Of F1-atpase From Saccharomyces Cerevisiae Inhibited By Its Regulatory Protein If1
pdb|3ZIA|L **Mass:** 54968 **Score:** 29 **Matches:** 1(0) **Sequences:** 1(0)
Chain L, The Structure Of F1-atpase From Saccharomyces Cerevisiae Inhibited By Its Regulatory Protein If1
pdb|3ZIA|M **Mass:** 54968 **Score:** 29 **Matches:** 1(0) **Sequences:** 1(0)
Chain M, The Structure Of F1-atpase From Saccharomyces Cerevisiae Inhibited By Its Regulatory Protein If1
pdb|3ZRY|A **Mass:** 54968 **Score:** 29 **Matches:** 1(0) **Sequences:** 1(0)
Chain A, Rotor Architecture In The F(1)-C(10)-Ring Complex Of The Yeast F-Atp Synthase
pdb|3ZRY|B **Mass:** 54968 **Score:** 29 **Matches:** 1(0) **Sequences:** 1(0)
Chain B, Rotor Architecture In The F(1)-C(10)-Ring Complex Of The Yeast F-Atp Synthase
pdb|3ZRY|C **Mass:** 54968 **Score:** 29 **Matches:** 1(0) **Sequences:** 1(0)
Chain C, Rotor Architecture In The F(1)-C(10)-Ring Complex Of The Yeast F-Atp Synthase
pdb|4B2Q|A **Mass:** 52342 **Score:** 29 **Matches:** 1(0) **Sequences:** 1(0)
Chain A, Model Of The Yeast F1fo-atp Synthase Dimer Based On Subtomogram Average
pdb|4B2Q|AA **Mass:** 52342 **Score:** 29 **Matches:** 1(0) **Sequences:** 1(0)
Chain a, Model Of The Yeast F1fo-atp Synthase Dimer Based On Subtomogram Average
pdb|4B2Q|B **Mass:** 52413 **Score:** 29 **Matches:** 1(0) **Sequences:** 1(0)
Chain B, Model Of The Yeast F1fo-atp Synthase Dimer Based On Subtomogram Average
pdb|4B2Q|BB **Mass:** 52413 **Score:** 29 **Matches:** 1(0) **Sequences:** 1(0)
Chain b, Model Of The Yeast F1fo-atp Synthase Dimer Based On Subtomogram Average
pdb|4B2Q|C **Mass:** 52342 **Score:** 29 **Matches:** 1(0) **Sequences:** 1(0)
Chain C, Model Of The Yeast F1fo-atp Synthase Dimer Based On Subtomogram Average
pdb|4B2Q|CC **Mass:** 52342 **Score:** 29 **Matches:** 1(0) **Sequences:** 1(0)
Chain c, Model Of The Yeast F1fo-atp Synthase Dimer Based On Subtomogram Average
gb|AJQ00300.1| **Mass:** 58629 **Score:** 29 **Matches:** 1(0) **Sequences:** 1(0)
Atp1p [Saccharomyces cerevisiae YJM244]
gb|AJQ00689.1| **Mass:** 58629 **Score:** 29 **Matches:** 1(0) **Sequences:** 1(0)
Atp1p [Saccharomyces cerevisiae YJM248]
gb|AJQ01073.1| **Mass:** 58643 **Score:** 29 **Matches:** 1(0) **Sequences:** 1(0)
Atp1p [Saccharomyces cerevisiae YJM270]
gb|AJQ01458.1| **Mass:** 58629 **Score:** 29 **Matches:** 1(0) **Sequences:** 1(0)
Atp1p [Saccharomyces cerevisiae YJM271]
gb|AJQ01843.1| **Mass:** 58629 **Score:** 29 **Matches:** 1(0) **Sequences:** 1(0)
Atp1p [Saccharomyces cerevisiae YJM320]
gb|AJQ02231.1| **Mass:** 58629 **Score:** 29 **Matches:** 1(0) **Sequences:** 1(0)
Atp1p [Saccharomyces cerevisiae YJM326]
gb|AJQ02621.1| **Mass:** 58557 **Score:** 29 **Matches:** 1(0) **Sequences:** 1(0)
Atp1p [Saccharomyces cerevisiae YJM428]
gb|AJQ03008.1| **Mass:** 58629 **Score:** 29 **Matches:** 1(0) **Sequences:** 1(0)
Atp1p [Saccharomyces cerevisiae YJM450]
gb|AJQ03379.1| **Mass:** 58629 **Score:** 29 **Matches:** 1(0) **Sequences:** 1(0)

Atp1p [Saccharomyces cerevisiae YJM451]
gb|AJQ03763.1| **Mass:** 58629 **Score:** 29 **Matches:** 1(0) **Sequences:** 1(0)
Atp1p [Saccharomyces cerevisiae YJM453]
gb|AJQ04111.1| **Mass:** 58629 **Score:** 29 **Matches:** 1(0) **Sequences:** 1(0)
Atp1p [Saccharomyces cerevisiae YJM456]
gb|AJQ04497.1| **Mass:** 58629 **Score:** 29 **Matches:** 1(0) **Sequences:** 1(0)
Atp1p [Saccharomyces cerevisiae YJM470]
gb|AJQ04878.1| **Mass:** 58629 **Score:** 29 **Matches:** 1(0) **Sequences:** 1(0)
Atp1p [Saccharomyces cerevisiae YJM541]
gb|AJQ05264.1| **Mass:** 58629 **Score:** 29 **Matches:** 1(0) **Sequences:** 1(0)
Atp1p [Saccharomyces cerevisiae YJM554]
gb|AJQ05650.1| **Mass:** 58629 **Score:** 29 **Matches:** 1(0) **Sequences:** 1(0)
Atp1p [Saccharomyces cerevisiae YJM555]
gb|AJQ06036.1| **Mass:** 58629 **Score:** 29 **Matches:** 1(0) **Sequences:** 1(0)
Atp1p [Saccharomyces cerevisiae YJM627]
gb|AJQ06423.1| **Mass:** 58629 **Score:** 29 **Matches:** 1(0) **Sequences:** 1(0)
Atp1p [Saccharomyces cerevisiae YJM681]
gb|AJQ06787.1| **Mass:** 58629 **Score:** 29 **Matches:** 1(0) **Sequences:** 1(0)
Atp1p [Saccharomyces cerevisiae YJM682]
gb|AJQ07170.1| **Mass:** 58629 **Score:** 29 **Matches:** 1(0) **Sequences:** 1(0)
Atp1p [Saccharomyces cerevisiae YJM683]
gb|AJQ07558.1| **Mass:** 58629 **Score:** 29 **Matches:** 1(0) **Sequences:** 1(0)
Atp1p [Saccharomyces cerevisiae YJM689]
gb|AJQ07943.1| **Mass:** 58629 **Score:** 29 **Matches:** 1(0) **Sequences:** 1(0)
Atp1p [Saccharomyces cerevisiae YJM1252]
gb|AJQ08327.1| **Mass:** 58629 **Score:** 29 **Matches:** 1(0) **Sequences:** 1(0)
Atp1p [Saccharomyces cerevisiae YJM1273]
gb|AJQ08716.1| **Mass:** 58629 **Score:** 29 **Matches:** 1(0) **Sequences:** 1(0)
Atp1p [Saccharomyces cerevisiae YJM1304]
gb|AJQ09101.1| **Mass:** 58629 **Score:** 29 **Matches:** 1(0) **Sequences:** 1(0)
Atp1p [Saccharomyces cerevisiae YJM1307]
gb|AJQ09485.1| **Mass:** 58629 **Score:** 29 **Matches:** 1(0) **Sequences:** 1(0)
Atp1p [Saccharomyces cerevisiae YJM1311]
gb|AJQ09872.1| **Mass:** 58629 **Score:** 29 **Matches:** 1(0) **Sequences:** 1(0)
Atp1p [Saccharomyces cerevisiae YJM1326]
gb|AJQ10259.1| **Mass:** 58629 **Score:** 29 **Matches:** 1(0) **Sequences:** 1(0)
Atp1p [Saccharomyces cerevisiae YJM1332]
gb|AJQ10645.1| **Mass:** 58629 **Score:** 29 **Matches:** 1(0) **Sequences:** 1(0)
Atp1p [Saccharomyces cerevisiae YJM1336]
gb|AJQ10994.1| **Mass:** 58629 **Score:** 29 **Matches:** 1(0) **Sequences:** 1(0)
Atp1p [Saccharomyces cerevisiae YJM1338]
gb|AJQ11380.1| **Mass:** 58629 **Score:** 29 **Matches:** 1(0) **Sequences:** 1(0)
Atp1p [Saccharomyces cerevisiae YJM1341]
gb|AJQ11767.1| **Mass:** 58659 **Score:** 29 **Matches:** 1(0) **Sequences:** 1(0)
Atp1p [Saccharomyces cerevisiae YJM1342]
gb|AJQ12147.1| **Mass:** 58629 **Score:** 29 **Matches:** 1(0) **Sequences:** 1(0)
Atp1p [Saccharomyces cerevisiae YJM1355]
gb|AJQ12528.1| **Mass:** 58629 **Score:** 29 **Matches:** 1(0) **Sequences:** 1(0)
Atp1p [Saccharomyces cerevisiae YJM1356]
gb|AJQ12915.1| **Mass:** 58629 **Score:** 29 **Matches:** 1(0) **Sequences:** 1(0)
Atp1p [Saccharomyces cerevisiae YJM1381]
gb|AJQ13300.1| **Mass:** 58629 **Score:** 29 **Matches:** 1(0) **Sequences:** 1(0)
Atp1p [Saccharomyces cerevisiae YJM1383]
dbj|BAA13613.1| **Mass:** 58641 **Score:** 29 **Matches:** 1(0) **Sequences:** 1(0)
defective F1F0-ATPase alpha subunit precursor [Saccharomyces cerevisiae]
gb|AJQ13688.1| **Mass:** 58629 **Score:** 29 **Matches:** 1(0) **Sequences:** 1(0)
Atp1p [Saccharomyces cerevisiae YJM1385]
gb|AJQ14071.1| **Mass:** 58629 **Score:** 29 **Matches:** 1(0) **Sequences:** 1(0)
Atp1p [Saccharomyces cerevisiae YJM1386]
gb|AJQ14457.1| **Mass:** 58629 **Score:** 29 **Matches:** 1(0) **Sequences:** 1(0)
Atp1p [Saccharomyces cerevisiae YJM1387]
gb|AJQ14842.1| **Mass:** 58629 **Score:** 29 **Matches:** 1(0) **Sequences:** 1(0)
Atp1p [Saccharomyces cerevisiae YJM1388]
gb|AJQ15219.1| **Mass:** 58643 **Score:** 29 **Matches:** 1(0) **Sequences:** 1(0)
Atp1p [Saccharomyces cerevisiae YJM1389]
gb|AJQ15596.1| **Mass:** 58629 **Score:** 29 **Matches:** 1(0) **Sequences:** 1(0)
Atp1p [Saccharomyces cerevisiae YJM1399]
gb|AJQ15977.1| **Mass:** 58641 **Score:** 29 **Matches:** 1(0) **Sequences:** 1(0)
Atp1p [Saccharomyces cerevisiae YJM1400]
gb|AJQ16356.1| **Mass:** 58629 **Score:** 29 **Matches:** 1(0) **Sequences:** 1(0)
Atp1p [Saccharomyces cerevisiae YJM1401]
dbj|BAK20454.1| **Mass:** 58639 **Score:** 29 **Matches:** 1(0) **Sequences:** 1(0)
F1F0-ATPase complex [Saccharomyces cerevisiae]
dbj|BAK20455.1| **Mass:** 58639 **Score:** 29 **Matches:** 1(0) **Sequences:** 1(0)
F1F0-ATPase complex [Saccharomyces cerevisiae]
dbj|BAK20456.1| **Mass:** 58639 **Score:** 29 **Matches:** 1(0) **Sequences:** 1(0)
F1F0-ATPase complex [Saccharomyces cerevisiae]
dbj|BAA22508.1| **Mass:** 58629 **Score:** 29 **Matches:** 1(0) **Sequences:** 1(0)
F1F0-ATPase alpha subunit precursor [Saccharomyces cerevisiae]
dbj|BAA22509.1| **Mass:** 58687 **Score:** 29 **Matches:** 1(0) **Sequences:** 1(0)
defective F1F0-ATPase alpha subunit precursor [Saccharomyces cerevisiae]
gb|AJP36996.1| **Mass:** 58629 **Score:** 29 **Matches:** 1(0) **Sequences:** 1(0)
Atp1p [Saccharomyces cerevisiae YJM1078]
emb|CAA56001.1| **Mass:** 58639 **Score:** 29 **Matches:** 1(0) **Sequences:** 1(0)
C-545 protein [Saccharomyces cerevisiae]
gb|AAA66888.1| **Mass:** 58587 **Score:** 29 **Matches:** 1(0) **Sequences:** 1(0)
ATPase 1 alpha subunit [Saccharomyces cerevisiae]
gb|AHY74394.1| **Mass:** 58629 **Score:** 29 **Matches:** 1(0) **Sequences:** 1(0)

Atp1p [Saccharomyces cerevisiae YJM993]
emb|CAY77684.1| **Mass:** 58629 **Score:** 29 **Matches:** 1(0) **Sequences:** 1(0)
Atp1p [Saccharomyces cerevisiae EC1118]
gb|AJP81898.1| **Mass:** 58610 **Score:** 29 **Matches:** 1(0) **Sequences:** 1(0)
Atp1p [Saccharomyces cerevisiae YJM1402]
gb|AJP82278.1| **Mass:** 58629 **Score:** 29 **Matches:** 1(0) **Sequences:** 1(0)
Atp1p [Saccharomyces cerevisiae YJM1415]
gb|AJP82666.1| **Mass:** 58629 **Score:** 29 **Matches:** 1(0) **Sequences:** 1(0)
Atp1p [Saccharomyces cerevisiae YJM1417]
gb|AJP83059.1| **Mass:** 58629 **Score:** 29 **Matches:** 1(0) **Sequences:** 1(0)
Atp1p [Saccharomyces cerevisiae YJM1418]
gb|AJP83444.1| **Mass:** 58629 **Score:** 29 **Matches:** 1(0) **Sequences:** 1(0)
Atp1p [Saccharomyces cerevisiae YJM1419]
gb|AJP83832.1| **Mass:** 58629 **Score:** 29 **Matches:** 1(0) **Sequences:** 1(0)
Atp1p [Saccharomyces cerevisiae YJM1433]
gb|AJP84206.1| **Mass:** 58629 **Score:** 29 **Matches:** 1(0) **Sequences:** 1(0)
Atp1p [Saccharomyces cerevisiae YJM1434]
gb|AJP84595.1| **Mass:** 58629 **Score:** 29 **Matches:** 1(0) **Sequences:** 1(0)
Atp1p [Saccharomyces cerevisiae YJM1439]
emb|CAA84924.1| **Mass:** 58639 **Score:** 29 **Matches:** 1(0) **Sequences:** 1(0)
ATP1 [Saccharomyces cerevisiae]
gb|AJP84977.1| **Mass:** 58629 **Score:** 29 **Matches:** 1(0) **Sequences:** 1(0)
Atp1p [Saccharomyces cerevisiae YJM1443]
gb|AJP85351.1| **Mass:** 58629 **Score:** 29 **Matches:** 1(0) **Sequences:** 1(0)
Atp1p [Saccharomyces cerevisiae YJM1444]
gb|AJP85737.1| **Mass:** 58629 **Score:** 29 **Matches:** 1(0) **Sequences:** 1(0)
Atp1p [Saccharomyces cerevisiae YJM1447]
gb|AJP86110.1| **Mass:** 58629 **Score:** 29 **Matches:** 1(0) **Sequences:** 1(0)
Atp1p [Saccharomyces cerevisiae YJM1450]
gb|AJP86493.1| **Mass:** 58629 **Score:** 29 **Matches:** 1(0) **Sequences:** 1(0)
Atp1p [Saccharomyces cerevisiae YJM1460]
gb|AJP86877.1| **Mass:** 58629 **Score:** 29 **Matches:** 1(0) **Sequences:** 1(0)
Atp1p [Saccharomyces cerevisiae YJM1463]
gb|AJP87262.1| **Mass:** 58629 **Score:** 29 **Matches:** 1(0) **Sequences:** 1(0)
Atp1p [Saccharomyces cerevisiae YJM1477]
gb|AJP87651.1| **Mass:** 58629 **Score:** 29 **Matches:** 1(0) **Sequences:** 1(0)
Atp1p [Saccharomyces cerevisiae YJM1478]
gb|AJP88001.1| **Mass:** 58641 **Score:** 29 **Matches:** 1(0) **Sequences:** 1(0)
Atp1p [Saccharomyces cerevisiae YJM1479]
gb|AJP88381.1| **Mass:** 58629 **Score:** 29 **Matches:** 1(0) **Sequences:** 1(0)
Atp1p [Saccharomyces cerevisiae YJM1526]
gb|AJP88770.1| **Mass:** 58645 **Score:** 29 **Matches:** 1(0) **Sequences:** 1(0)
Atp1p [Saccharomyces cerevisiae YJM1527]
gb|AJP89159.1| **Mass:** 58629 **Score:** 29 **Matches:** 1(0) **Sequences:** 1(0)
Atp1p [Saccharomyces cerevisiae YJM1549]
gb|AJP89542.1| **Mass:** 58610 **Score:** 29 **Matches:** 1(0) **Sequences:** 1(0)
Atp1p [Saccharomyces cerevisiae YJM1573]
gb|AJP89920.1| **Mass:** 58643 **Score:** 29 **Matches:** 1(0) **Sequences:** 1(0)
Atp1p [Saccharomyces cerevisiae YJM1574]
gb|AJP90240.1| **Mass:** 58643 **Score:** 29 **Matches:** 1(0) **Sequences:** 1(0)
Atp1p [Saccharomyces cerevisiae YJM1592]
gb|AJP90625.1| **Mass:** 58629 **Score:** 29 **Matches:** 1(0) **Sequences:** 1(0)
Atp1p [Saccharomyces cerevisiae YJM1615]
gb|AJP91008.1| **Mass:** 58629 **Score:** 29 **Matches:** 1(0) **Sequences:** 1(0)
Atp1p [Saccharomyces cerevisiae YJM693]
gb|AJP91395.1| **Mass:** 58629 **Score:** 29 **Matches:** 1(0) **Sequences:** 1(0)
Atp1p [Saccharomyces cerevisiae YJM969]
gb|AJP91782.1| **Mass:** 58629 **Score:** 29 **Matches:** 1(0) **Sequences:** 1(0)
Atp1p [Saccharomyces cerevisiae YJM972]
gb|AJP92172.1| **Mass:** 58629 **Score:** 29 **Matches:** 1(0) **Sequences:** 1(0)
Atp1p [Saccharomyces cerevisiae YJM975]
gb|AJP92560.1| **Mass:** 58629 **Score:** 29 **Matches:** 1(0) **Sequences:** 1(0)
Atp1p [Saccharomyces cerevisiae YJM978]
gb|AJP92949.1| **Mass:** 58629 **Score:** 29 **Matches:** 1(0) **Sequences:** 1(0)
Atp1p [Saccharomyces cerevisiae YJM981]
gb|AAT92988.1| **Mass:** 58629 **Score:** 29 **Matches:** 1(0) **Sequences:** 1(0)
YBL099W [Saccharomyces cerevisiae]
gb|AJP93339.1| **Mass:** 58629 **Score:** 29 **Matches:** 1(0) **Sequences:** 1(0)
Atp1p [Saccharomyces cerevisiae YJM984]
gb|AJP93728.1| **Mass:** 58629 **Score:** 29 **Matches:** 1(0) **Sequences:** 1(0)
Atp1p [Saccharomyces cerevisiae YJM987]
gb|AJP94118.1| **Mass:** 58629 **Score:** 29 **Matches:** 1(0) **Sequences:** 1(0)
Atp1p [Saccharomyces cerevisiae YJM990]
gb|AJP94509.1| **Mass:** 58629 **Score:** 29 **Matches:** 1(0) **Sequences:** 1(0)
Atp1p [Saccharomyces cerevisiae YJM996]
gb|AJP94898.1| **Mass:** 58629 **Score:** 29 **Matches:** 1(0) **Sequences:** 1(0)
Atp1p [Saccharomyces cerevisiae YJM1083]
gb|AJP95283.1| **Mass:** 58629 **Score:** 29 **Matches:** 1(0) **Sequences:** 1(0)
Atp1p [Saccharomyces cerevisiae YJM1129]
gb|AJP95672.1| **Mass:** 58629 **Score:** 29 **Matches:** 1(0) **Sequences:** 1(0)
Atp1p [Saccharomyces cerevisiae YJM1133]
gb|AJP96059.1| **Mass:** 58629 **Score:** 29 **Matches:** 1(0) **Sequences:** 1(0)
Atp1p [Saccharomyces cerevisiae YJM1190]
gb|AJP96441.1| **Mass:** 58629 **Score:** 29 **Matches:** 1(0) **Sequences:** 1(0)
Atp1p [Saccharomyces cerevisiae YJM1199]
gb|AJP96827.1| **Mass:** 58629 **Score:** 29 **Matches:** 1(0) **Sequences:** 1(0)
Atp1p [Saccharomyces cerevisiae YJM1202]
gb|AJP97210.1| **Mass:** 58629 **Score:** 29 **Matches:** 1(0) **Sequences:** 1(0)

Atp1p [Saccharomyces cerevisiae YJM1208]
gb|AJP97595.1| **Mass:** 58629 **Score:** 29 **Matches:** 1(0) **Sequences:** 1(0)
Atp1p [Saccharomyces cerevisiae YJM1242]
gb|AJP97983.1| **Mass:** 58629 **Score:** 29 **Matches:** 1(0) **Sequences:** 1(0)
Atp1p [Saccharomyces cerevisiae YJM1244]
gb|AJP98370.1| **Mass:** 58629 **Score:** 29 **Matches:** 1(0) **Sequences:** 1(0)
Atp1p [Saccharomyces cerevisiae YJM1248]
gb|AJP98754.1| **Mass:** 58629 **Score:** 29 **Matches:** 1(0) **Sequences:** 1(0)
Atp1p [Saccharomyces cerevisiae YJM1250]
gb|AJP99142.1| **Mass:** 58629 **Score:** 29 **Matches:** 1(0) **Sequences:** 1(0)
Atp1p [Saccharomyces cerevisiae YJM189]
gb|AJP99532.1| **Mass:** 58629 **Score:** 29 **Matches:** 1(0) **Sequences:** 1(0)
Atp1p [Saccharomyces cerevisiae YJM193]
gb|AJP99916.1| **Mass:** 58629 **Score:** 29 **Matches:** 1(0) **Sequences:** 1(0)
Atp1p [Saccharomyces cerevisiae YJM195]

---

14. emb|CAF06149.1| **Mass:** 68898 **Score:** 20 **Matches:** 1(0) **Sequences:** 1(0)
related to Pol II transcription elongation factor [Neurospora crassa]
☐ Check to include this hit in error tolerant search or archive report

Query	Observed	Mr(expt)	Mr(calc)	ppm	Miss	Score	Expect	Rank	Unique	Peptide
28	1541.8912	1540.8839	1540.8321	33.6	2	20	16	1	U	R.EIEREQILAERR.E

---

15. emb|CCA61330.1| **Score:** 19 **Matches:** 1(0) **Sequences:** 1(0)
ER membrane localized phosphoryltransferase [Saccharomyces uvarum]
☐ Check to include this hit in error tolerant search or archive report

Query	Observed	Mr(expt)	Mr(calc)	ppm	Miss	Score	Expect	Rank	Unique	Peptide
9	1297.6991	1296.6918	1296.5946	75.0	0	19	25	2	U	K.DSSNYDISALGR.D

Proteins matching the same set of peptides:
emb|CCA61331.1| **Score:** 19 **Matches:** 1(0) **Sequences:** 1(0)
emb|CCA61332.1| **Score:** 19 **Matches:** 1(0) **Sequences:** 1(0)
emb|CCA61335.1| **Score:** 19 **Matches:** 1(0) **Sequences:** 1(0)

---

16. pdb|1AY0|A **Mass:** 73808 **Score:** 19 **Matches:** 1(0) **Sequences:** 1(0)
Chain A, Identification Of Catalytically Important Residues In Yeast Transketolase
☐ Check to include this hit in error tolerant search or archive report

Query	Observed	Mr(expt)	Mr(calc)	ppm	Miss	Score	Expect	Rank	Unique	Peptide
12	1356.7451	1355.7379	1355.6510	64.1	0	19	24	1	U	K.FFGFTPEGVAER.A

Proteins matching the same set of peptides:
pdb|1AY0|B **Mass:** 73808 **Score:** 19 **Matches:** 1(0) **Sequences:** 1(0)
Chain B, Identification Of Catalytically Important Residues In Yeast Transketolase
pdb|1GPU|A **Mass:** 73874 **Score:** 19 **Matches:** 1(0) **Sequences:** 1(0)
Chain A, Transketolase Complex With Reaction Intermediate
pdb|1GPU|B **Mass:** 73874 **Score:** 19 **Matches:** 1(0) **Sequences:** 1(0)
Chain B, Transketolase Complex With Reaction Intermediate
pdb|1NGS|A **Mass:** 73874 **Score:** 19 **Matches:** 1(0) **Sequences:** 1(0)
Chain A, Complex Of Transketolase With Thiamin Diphosphate, Ca2+ And Acceptor Substrate Erythrose-4-phosphate
pdb|1NGS|B **Mass:** 73874 **Score:** 19 **Matches:** 1(0) **Sequences:** 1(0)
Chain B, Complex Of Transketolase With Thiamin Diphosphate, Ca2+ And Acceptor Substrate Erythrose-4-phosphate
pdb|1TKA|A **Mass:** 73642 **Score:** 19 **Matches:** 1(0) **Sequences:** 1(0)
Chain A, Specificity Of Coenzyme Binding In Thiamin Diphosphate Dependent Enzymes: Crystal Structures Of Yeast Transketolase In Complex
pdb|1TKA|B **Mass:** 73642 **Score:** 19 **Matches:** 1(0) **Sequences:** 1(0)
Chain B, Specificity Of Coenzyme Binding In Thiamin Diphosphate Dependent Enzymes: Crystal Structures Of Yeast Transketolase In Complex
pdb|1TKB|A **Mass:** 73642 **Score:** 19 **Matches:** 1(0) **Sequences:** 1(0)
Chain A, Specificity Of Coenzyme Binding In Thiamin Diphosphate Dependent Enzymes: Crystal Structures Of Yeast Transketolase In Complex
pdb|1TKB|B **Mass:** 73642 **Score:** 19 **Matches:** 1(0) **Sequences:** 1(0)
Chain B, Specificity Of Coenzyme Binding In Thiamin Diphosphate Dependent Enzymes: Crystal Structures Of Yeast Transketolase In Complex
pdb|1TKC|A **Mass:** 73642 **Score:** 19 **Matches:** 1(0) **Sequences:** 1(0)
Chain A, Specificity Of Coenzyme Binding In Thiamin Diphosphate Dependent Enzymes: Crystal Structures Of Yeast Transketolase In Complex
pdb|1TKC|B **Mass:** 73642 **Score:** 19 **Matches:** 1(0) **Sequences:** 1(0)
Chain B, Specificity Of Coenzyme Binding In Thiamin Diphosphate Dependent Enzymes: Crystal Structures Of Yeast Transketolase In Complex
pdb|1TRK|A **Mass:** 73874 **Score:** 19 **Matches:** 1(0) **Sequences:** 1(0)
Chain A, Refined Structure Of Transketolase From Saccharomyces Cerevisiae At 2.0 Angstroms Resolution
pdb|1TRK|B **Mass:** 73874 **Score:** 19 **Matches:** 1(0) **Sequences:** 1(0)
Chain B, Refined Structure Of Transketolase From Saccharomyces Cerevisiae At 2.0 Angstroms Resolution
gb|AJW00215.1| **Mass:** 73874 **Score:** 19 **Matches:** 1(0) **Sequences:** 1(0)
Tkl1p [Saccharomyces cerevisiae YJM450]
gb|AJW00644.1| **Mass:** 73874 **Score:** 19 **Matches:** 1(0) **Sequences:** 1(0)
Tkl1p [Saccharomyces cerevisiae YJM1356]
gb|AJW01083.1| **Mass:** 73874 **Score:** 19 **Matches:** 1(0) **Sequences:** 1(0)
Tkl1p [Saccharomyces cerevisiae YJM1381]
gb|AJW01519.1| **Mass:** 73874 **Score:** 19 **Matches:** 1(0) **Sequences:** 1(0)
Tkl1p [Saccharomyces cerevisiae YJM1383]
gb|AJW01954.1| **Mass:** 73874 **Score:** 19 **Matches:** 1(0) **Sequences:** 1(0)
Tkl1p [Saccharomyces cerevisiae YJM1385]
gb|AJW02371.1| **Mass:** 73874 **Score:** 19 **Matches:** 1(0) **Sequences:** 1(0)
Tkl1p [Saccharomyces cerevisiae YJM1386]
gb|AJW02796.1| **Mass:** 73874 **Score:** 19 **Matches:** 1(0) **Sequences:** 1(0)
Tkl1p [Saccharomyces cerevisiae YJM1387]
gb|AJW03238.1| **Mass:** 73874 **Score:** 19 **Matches:** 1(0) **Sequences:** 1(0)
Tkl1p [Saccharomyces cerevisiae YJM1388]
gb|AJW03675.1| **Mass:** 73874 **Score:** 19 **Matches:** 1(0) **Sequences:** 1(0)

Tkl1p [Saccharomyces cerevisiae YJM1389]
gb|AJW04111.1| **Mass:** 73874 **Score:** 19 **Matches:** 1(0) **Sequences:** 1(0)
Tkl1p [Saccharomyces cerevisiae YJM1399]
gb|AJW04542.1| **Mass:** 73874 **Score:** 19 **Matches:** 1(0) **Sequences:** 1(0)
Tkl1p [Saccharomyces cerevisiae YJM1400]
gb|AJW04977.1| **Mass:** 73874 **Score:** 19 **Matches:** 1(0) **Sequences:** 1(0)
Tkl1p [Saccharomyces cerevisiae YJM1401]
gb|AJW05415.1| **Mass:** 73874 **Score:** 19 **Matches:** 1(0) **Sequences:** 1(0)
Tkl1p [Saccharomyces cerevisiae YJM1402]
gb|AJW05857.1| **Mass:** 73874 **Score:** 19 **Matches:** 1(0) **Sequences:** 1(0)
Tkl1p [Saccharomyces cerevisiae YJM1415]
gb|AJW06295.1| **Mass:** 73874 **Score:** 19 **Matches:** 1(0) **Sequences:** 1(0)
Tkl1p [Saccharomyces cerevisiae YJM1417]
gb|AJW06733.1| **Mass:** 73864 **Score:** 19 **Matches:** 1(0) **Sequences:** 1(0)
Tkl1p [Saccharomyces cerevisiae YJM1418]
gb|AJW07167.1| **Mass:** 73874 **Score:** 19 **Matches:** 1(0) **Sequences:** 1(0)
Tkl1p [Saccharomyces cerevisiae YJM1419]
gb|AJW07605.1| **Mass:** 73874 **Score:** 19 **Matches:** 1(0) **Sequences:** 1(0)
Tkl1p [Saccharomyces cerevisiae YJM1433]
gb|AJW08035.1| **Mass:** 73874 **Score:** 19 **Matches:** 1(0) **Sequences:** 1(0)
Tkl1p [Saccharomyces cerevisiae YJM1434]
gb|AJW08473.1| **Mass:** 73876 **Score:** 19 **Matches:** 1(0) **Sequences:** 1(0)
Tkl1p [Saccharomyces cerevisiae YJM1439]
gb|AJW08899.1| **Mass:** 73874 **Score:** 19 **Matches:** 1(0) **Sequences:** 1(0)
Tkl1p [Saccharomyces cerevisiae YJM1443]
gb|AJW09338.1| **Mass:** 73874 **Score:** 19 **Matches:** 1(0) **Sequences:** 1(0)
Tkl1p [Saccharomyces cerevisiae YJM1444]
gb|AJW09773.1| **Mass:** 73874 **Score:** 19 **Matches:** 1(0) **Sequences:** 1(0)
Tkl1p [Saccharomyces cerevisiae YJM1447]
gb|AJW10210.1| **Mass:** 73874 **Score:** 19 **Matches:** 1(0) **Sequences:** 1(0)
Tkl1p [Saccharomyces cerevisiae YJM1450]
gb|AJW10644.1| **Mass:** 73874 **Score:** 19 **Matches:** 1(0) **Sequences:** 1(0)
Tkl1p [Saccharomyces cerevisiae YJM1083]
gb|AJW10914.1| **Mass:** 73874 **Score:** 19 **Matches:** 1(0) **Sequences:** 1(0)
Tkl1p [Saccharomyces cerevisiae YJM1129]
gb|AJW11354.1| **Mass:** 73874 **Score:** 19 **Matches:** 1(0) **Sequences:** 1(0)
Tkl1p [Saccharomyces cerevisiae YJM1133]
gb|AJW11797.1| **Mass:** 73874 **Score:** 19 **Matches:** 1(0) **Sequences:** 1(0)
Tkl1p [Saccharomyces cerevisiae YJM1190]
gb|AJW12236.1| **Mass:** 73874 **Score:** 19 **Matches:** 1(0) **Sequences:** 1(0)
Tkl1p [Saccharomyces cerevisiae YJM1199]
gb|AJW12679.1| **Mass:** 73888 **Score:** 19 **Matches:** 1(0) **Sequences:** 1(0)
Tkl1p [Saccharomyces cerevisiae YJM1202]
gb|AJW13115.1| **Mass:** 73874 **Score:** 19 **Matches:** 1(0) **Sequences:** 1(0)
Tkl1p [Saccharomyces cerevisiae YJM1208]
gb|AJW13540.1| **Mass:** 73874 **Score:** 19 **Matches:** 1(0) **Sequences:** 1(0)
Tkl1p [Saccharomyces cerevisiae YJM1242]
gb|AJW13979.1| **Mass:** 73874 **Score:** 19 **Matches:** 1(0) **Sequences:** 1(0)
Tkl1p [Saccharomyces cerevisiae YJM1244]
gb|AJW14417.1| **Mass:** 73876 **Score:** 19 **Matches:** 1(0) **Sequences:** 1(0)
Tkl1p [Saccharomyces cerevisiae YJM1248]
gb|AJW14840.1| **Mass:** 73873 **Score:** 19 **Matches:** 1(0) **Sequences:** 1(0)
Tkl1p [Saccharomyces cerevisiae YJM1250]
gb|AJW15264.1| **Mass:** 73874 **Score:** 19 **Matches:** 1(0) **Sequences:** 1(0)
Tkl1p [Saccharomyces cerevisiae YJM1252]
gb|AJW15701.1| **Mass:** 73874 **Score:** 19 **Matches:** 1(0) **Sequences:** 1(0)
Tkl1p [Saccharomyces cerevisiae YJM1273]
gb|AJW16144.1| **Mass:** 73874 **Score:** 19 **Matches:** 1(0) **Sequences:** 1(0)
Tkl1p [Saccharomyces cerevisiae YJM1304]
gb|AJW16579.1| **Mass:** 73874 **Score:** 19 **Matches:** 1(0) **Sequences:** 1(0)
Tkl1p [Saccharomyces cerevisiae YJM1307]
gb|AJW17017.1| **Mass:** 73874 **Score:** 19 **Matches:** 1(0) **Sequences:** 1(0)
Tkl1p [Saccharomyces cerevisiae YJM1311]
gb|AJW17441.1| **Mass:** 73874 **Score:** 19 **Matches:** 1(0) **Sequences:** 1(0)
Tkl1p [Saccharomyces cerevisiae YJM1326]
gb|AJW17887.1| **Mass:** 73874 **Score:** 19 **Matches:** 1(0) **Sequences:** 1(0)
Tkl1p [Saccharomyces cerevisiae YJM1332]
gb|AJW18326.1| **Mass:** 73874 **Score:** 19 **Matches:** 1(0) **Sequences:** 1(0)
Tkl1p [Saccharomyces cerevisiae YJM1336]
gb|AJW18763.1| **Mass:** 73874 **Score:** 19 **Matches:** 1(0) **Sequences:** 1(0)
Tkl1p [Saccharomyces cerevisiae YJM1338]
gb|AJW19201.1| **Mass:** 73874 **Score:** 19 **Matches:** 1(0) **Sequences:** 1(0)
Tkl1p [Saccharomyces cerevisiae YJM1341]
gb|AJW19626.1| **Mass:** 73892 **Score:** 19 **Matches:** 1(0) **Sequences:** 1(0)
Tkl1p [Saccharomyces cerevisiae YJM1342]
gb|AJW20043.1| **Mass:** 73874 **Score:** 19 **Matches:** 1(0) **Sequences:** 1(0)
Tkl1p [Saccharomyces cerevisiae YJM1355]
gb|AJW20480.1| **Mass:** 73874 **Score:** 19 **Matches:** 1(0) **Sequences:** 1(0)
Tkl1p [Saccharomyces cerevisiae YJM451]
gb|AJW20925.1| **Mass:** 73874 **Score:** 19 **Matches:** 1(0) **Sequences:** 1(0)
Tkl1p [Saccharomyces cerevisiae YJM453]
gb|AJW21360.1| **Mass:** 73874 **Score:** 19 **Matches:** 1(0) **Sequences:** 1(0)
Tkl1p [Saccharomyces cerevisiae YJM456]
gb|AJW21801.1| **Mass:** 73860 **Score:** 19 **Matches:** 1(0) **Sequences:** 1(0)
Tkl1p [Saccharomyces cerevisiae YJM470]
gb|AJW22229.1| **Mass:** 73874 **Score:** 19 **Matches:** 1(0) **Sequences:** 1(0)
Tkl1p [Saccharomyces cerevisiae YJM541]
gb|AJW22667.1| **Mass:** 73874 **Score:** 19 **Matches:** 1(0) **Sequences:** 1(0)

Tkl1p [Saccharomyces cerevisiae YJM554]
gb|AJW23108.1| **Mass:** 73874 **Score:** 19 **Matches:** 1(0) **Sequences:** 1(0)
Tkl1p [Saccharomyces cerevisiae YJM555]
gb|AJW23550.1| **Mass:** 73873 **Score:** 19 **Matches:** 1(0) **Sequences:** 1(0)
Tkl1p [Saccharomyces cerevisiae YJM627]
gb|AJW23983.1| **Mass:** 73904 **Score:** 19 **Matches:** 1(0) **Sequences:** 1(0)
Tkl1p [Saccharomyces cerevisiae YJM681]
gb|AJW24421.1| **Mass:** 73874 **Score:** 19 **Matches:** 1(0) **Sequences:** 1(0)
Tkl1p [Saccharomyces cerevisiae YJM682]
gb|AJW24860.1| **Mass:** 73874 **Score:** 19 **Matches:** 1(0) **Sequences:** 1(0)
Tkl1p [Saccharomyces cerevisiae YJM683]
gb|AJW25302.1| **Mass:** 73874 **Score:** 19 **Matches:** 1(0) **Sequences:** 1(0)
Tkl1p [Saccharomyces cerevisiae YJM689]
gb|AJW25708.1| **Mass:** 73888 **Score:** 19 **Matches:** 1(0) **Sequences:** 1(0)
Tkl1p [Saccharomyces cerevisiae YJM693]
gb|AJW25959.1| **Mass:** 73874 **Score:** 19 **Matches:** 1(0) **Sequences:** 1(0)
Tkl1p [Saccharomyces cerevisiae YJM969]
gb|AJW26226.1| **Mass:** 73874 **Score:** 19 **Matches:** 1(0) **Sequences:** 1(0)
Tkl1p [Saccharomyces cerevisiae YJM972]
gb|AJW26624.1| **Mass:** 73874 **Score:** 19 **Matches:** 1(0) **Sequences:** 1(0)
Tkl1p [Saccharomyces cerevisiae YJM975]
gb|AJW26891.1| **Mass:** 73874 **Score:** 19 **Matches:** 1(0) **Sequences:** 1(0)
Tkl1p [Saccharomyces cerevisiae YJM978]
gb|AJW27159.1| **Mass:** 73874 **Score:** 19 **Matches:** 1(0) **Sequences:** 1(0)
Tkl1p [Saccharomyces cerevisiae YJM981]
gb|AJW27587.1| **Mass:** 73874 **Score:** 19 **Matches:** 1(0) **Sequences:** 1(0)
Tkl1p [Saccharomyces cerevisiae YJM984]
gb|AJW27854.1| **Mass:** 73874 **Score:** 19 **Matches:** 1(0) **Sequences:** 1(0)
Tkl1p [Saccharomyces cerevisiae YJM987]
gb|AJW28297.1| **Mass:** 73874 **Score:** 19 **Matches:** 1(0) **Sequences:** 1(0)
Tkl1p [Saccharomyces cerevisiae YJM990]
gb|AJW28566.1| **Mass:** 73874 **Score:** 19 **Matches:** 1(0) **Sequences:** 1(0)
Tkl1p [Saccharomyces cerevisiae YJM996]
emb|CAY37264.1| **Mass:** 73874 **Score:** 19 **Matches:** 1(0) **Sequences:** 1(0)
unnamed protein product [Saccharomyces cerevisiae]
gb|AJP42205.1| **Mass:** 73874 **Score:** 19 **Matches:** 1(0) **Sequences:** 1(0)
Tkl1p [Saccharomyces cerevisiae YJM1078]
emb|CAA51693.1| **Mass:** 73874 **Score:** 19 **Matches:** 1(0) **Sequences:** 1(0)
transketolase [Saccharomyces cerevisiae]
gb|AAB68125.1| **Mass:** 73874 **Score:** 19 **Matches:** 1(0) **Sequences:** 1(0)
Tkl1p: Transketolase 1 [Saccharomyces cerevisiae]
gb|AHY78237.1| **Mass:** 73874 **Score:** 19 **Matches:** 1(0) **Sequences:** 1(0)
Tkl1p [Saccharomyces cerevisiae YJM993]
emb|CAA89191.1| **Mass:** 73874 **Score:** 19 **Matches:** 1(0) **Sequences:** 1(0)
Tkl1p [Saccharomyces cerevisiae]
gb|AJV91295.1| **Mass:** 73874 **Score:** 19 **Matches:** 1(0) **Sequences:** 1(0)
Tkl1p [Saccharomyces cerevisiae YJM1460]
gb|AJV91732.1| **Mass:** 73874 **Score:** 19 **Matches:** 1(0) **Sequences:** 1(0)
Tkl1p [Saccharomyces cerevisiae YJM1463]
gb|AJV92173.1| **Mass:** 73874 **Score:** 19 **Matches:** 1(0) **Sequences:** 1(0)
Tkl1p [Saccharomyces cerevisiae YJM1477]
gb|AJV92612.1| **Mass:** 73888 **Score:** 19 **Matches:** 1(0) **Sequences:** 1(0)
Tkl1p [Saccharomyces cerevisiae YJM1478]
gb|AJV93045.1| **Mass:** 73874 **Score:** 19 **Matches:** 1(0) **Sequences:** 1(0)
Tkl1p [Saccharomyces cerevisiae YJM1479]
gb|AJV93311.1| **Mass:** 73874 **Score:** 19 **Matches:** 1(0) **Sequences:** 1(0)
Tkl1p [Saccharomyces cerevisiae YJM1526]
gb|AJV93750.1| **Mass:** 73874 **Score:** 19 **Matches:** 1(0) **Sequences:** 1(0)
Tkl1p [Saccharomyces cerevisiae YJM1527]
gb|AJV94017.1| **Mass:** 73874 **Score:** 19 **Matches:** 1(0) **Sequences:** 1(0)
Tkl1p [Saccharomyces cerevisiae YJM1549]
gb|AJV94456.1| **Mass:** 73874 **Score:** 19 **Matches:** 1(0) **Sequences:** 1(0)
Tkl1p [Saccharomyces cerevisiae YJM1573]
gb|AJV94726.1| **Mass:** 73860 **Score:** 19 **Matches:** 1(0) **Sequences:** 1(0)
Tkl1p [Saccharomyces cerevisiae YJM1574]
emb|CAA94982.1| **Mass:** 73874 **Score:** 19 **Matches:** 1(0) **Sequences:** 1(0)
Tkl1p [Saccharomyces cerevisiae]
gb|AJV95158.1| **Mass:** 73874 **Score:** 19 **Matches:** 1(0) **Sequences:** 1(0)
Tkl1p [Saccharomyces cerevisiae YJM1592]
gb|AJV95594.1| **Mass:** 73874 **Score:** 19 **Matches:** 1(0) **Sequences:** 1(0)
Tkl1p [Saccharomyces cerevisiae YJM1615]
gb|AJV95863.1| **Mass:** 73874 **Score:** 19 **Matches:** 1(0) **Sequences:** 1(0)
Tkl1p [Saccharomyces cerevisiae YJM189]
gb|AJV96303.1| **Mass:** 73874 **Score:** 19 **Matches:** 1(0) **Sequences:** 1(0)
Tkl1p [Saccharomyces cerevisiae YJM193]
gb|AJV96719.1| **Mass:** 73886 **Score:** 19 **Matches:** 1(0) **Sequences:** 1(0)
Tkl1p [Saccharomyces cerevisiae YJM195]
gb|AJV97156.1| **Mass:** 73874 **Score:** 19 **Matches:** 1(0) **Sequences:** 1(0)
Tkl1p [Saccharomyces cerevisiae YJM244]
gb|AJV97599.1| **Mass:** 73874 **Score:** 19 **Matches:** 1(0) **Sequences:** 1(0)
Tkl1p [Saccharomyces cerevisiae YJM248]
gb|AJV98038.1| **Mass:** 73874 **Score:** 19 **Matches:** 1(0) **Sequences:** 1(0)
Tkl1p [Saccharomyces cerevisiae YJM270]
gb|AJV98475.1| **Mass:** 73874 **Score:** 19 **Matches:** 1(0) **Sequences:** 1(0)
Tkl1p [Saccharomyces cerevisiae YJM271]
gb|AJV98909.1| **Mass:** 73874 **Score:** 19 **Matches:** 1(0) **Sequences:** 1(0)
Tkl1p [Saccharomyces cerevisiae YJM320]
gb|AJV99349.1| **Mass:** 73874 **Score:** 19 **Matches:** 1(0) **Sequences:** 1(0)

Tkl1p [Saccharomyces cerevisiae YJM326]
gb|AJV99790.1| **Mass:** 73874 **Score:** 19 **Matches:** 1(0) **Sequences:** 1(0)
Tkl1p [Saccharomyces cerevisiae YJM428]

---

17. dbj|BAA14841.2| **Score:** 16 **Matches:** 1(0) **Sequences:** 1(0)
enoyl-[acyl-carrier-protein] reductase, NADH-dependent [Escherichia coli str. K-12 substr. W3110]
☐ Check to include this hit in error tolerant search or archive report

Query	Observed	Mr(expt)	Mr(calc)	ppm	Miss	Score	Expect	Rank	Unique	Peptide
28	1541.8912	1540.8839	1540.7854	63.9	1	16	40	2	U	R.KMLAHCEAVTPIR.R + Oxidation (M)

---

18. emb|CAR57985.1| **Score:** 16 **Matches:** 1(0) **Sequences:** 1(0)
unnamed protein product [Candida glabrata]
☐ Check to include this hit in error tolerant search or archive report

Query	Observed	Mr(expt)	Mr(calc)	ppm	Miss	Score	Expect	Rank	Unique	Peptide
28	1541.8912	1540.8839	1540.9049	-13.63	2	16	40	3	U	K.QVSTNNLKKLLQR.L

---

19. dbj|BAC56234.1| **Score:** 14 **Matches:** 1(0) **Sequences:** 1(0)
putative SSK22 like MAPKK kinase [Neurospora crassa]
☐ Check to include this hit in error tolerant search or archive report

Query	Observed	Mr(expt)	Mr(calc)	ppm	Miss	Score	Expect	Rank	Unique	Peptide
15	1372.8112	1371.8039	1371.6783	91.6	0	14	60	2	U	R.HILGLGEDEYAR.L

---

20. emb|CAR28930.1| **Score:** 14 **Matches:** 1(0) **Sequences:** 1(0)
ZYRO0F15268p [Zygosaccharomyces rouxii]
☐ Check to include this hit in error tolerant search or archive report

Query	Observed	Mr(expt)	Mr(calc)	ppm	Miss	Score	Expect	Rank	Unique	Peptide
9	1297.6991	1296.6918	1296.6786	10.2	0	14	75	3	U	K.SLISQSHEAGLR.T

Peptide matches not assigned to protein hits: (no details means no match)

	Query	Observed	Mr(expt)	Mr(calc)	ppm	Miss	Score	Expect	Rank	Unique	Peptide
☑	25	**1510.9030**	**1509.8957**	**1509.8337**	**41.1**	**1**	**13**	**64**	**1**		**DVIPNMSALLGPKR**
☑	20	**1420.7913**	**1419.7841**	**1419.8099**	**-18.17**	**2**	**10**	**1.8e+002**	**1**		**WSRLYAITVRR**
☑	30	**1555.8383**	**1554.8310**	**1554.7255**	**67.8**	**0**	**9**	**2.4e+002**	**1**		**AAWYISYGEWGPR**
☑	53	**1801.0359**	**1800.0287**	**1799.9564**	**40.2**	**0**	**9**	**2.1e+002**	**1**		**QTHTSIGLLLGMSASLR + Oxidation (M)**
☑	46	**1747.0132**	**1746.0060**	**1745.9536**	**30.0**	**2**	**7**	**2.9e+002**	**1**		**RGLPLDADNIEIHRK**
☑	16	**1394.7938**	**1393.7865**	**1393.7071**	**57.0**	**2**	**7**	**3.3e+002**	**1**		**MYRLNIRCPR + Oxidation (M)**
☑	31	**1557.9539**	**1556.9466**	**1556.8457**	**64.9**	**2**	**6**	**3e+002**	**1**		**KSLSCDRIPNLVR**
☑	64	**1939.1585**	**1938.1512**	**1938.1514**	**-0.07**	**2**	**6**	**2.8e+002**	**1**		**TLTLPSKSITTLPPVSKR**
☑	2	*1116.7099*	*1115.7026*								
☑	4	*1141.7389*	*1140.7316*								
☑	5	*1152.7009*	*1151.6936*								
☑	6	*1208.7621*	*1207.7548*								
☑	10	*1338.8118*	*1337.8045*								
☑	11	*1346.7832*	*1345.7759*								
☑	13	*1362.7925*	*1361.7852*								
☑	14	*1366.8490*	*1365.8417*								
☑	18	*1405.9084*	*1404.9011*								
☑	19	*1413.7942*	*1412.7870*								
☑	22	*1438.9404*	*1437.9331*								
☑	23	*1498.8679*	*1497.8606*								
☑	24	*1507.7557*	*1506.7485*								
☑	27	*1537.8484*	*1536.8412*								
☑	33	*1567.9141*	*1566.9068*								
☑	34	*1570.9155*	*1569.9082*								
☑	36	*1589.9001*	*1588.8928*								
☑	37	*1628.9967*	*1627.9894*								
☑	38	*1670.0910*	*1669.0837*								
☑	40	*1703.9990*	*1702.9918*								
☑	42	*1727.9225*	*1726.9152*								
☑	44	*1733.9175*	*1732.9102*								
☑	45	*1743.9614*	*1742.9541*								
☑	47	*1752.0388*	*1751.0315*								
☑	48	*1763.1123*	*1762.1050*								
☑	49	*1764.9726*	*1763.9653*								
☑	50	*1768.9906*	*1767.9833*								
☑	51	*1781.1490*	*1780.1417*								
☑	54	*1812.9982*	*1811.9910*								
☑	55	*1822.9920*	*1821.9847*								
☑	57	*1864.0121*	*1863.0048*								
☑	59	*1889.0990*	*1888.0917*								
☑	62	*1931.0702*	*1930.0629*								
☑	63	*1935.1382*	*1934.1310*								
☑	65	*1958.2170*	*1957.2097*								
☑	67	*2044.1251*	*2043.1178*								
☑	68	*2074.2043*	*2073.1970*								
☑	71	*2183.1657*	*2182.1585*								

☑	72	*2191.1320*	*2190.1248*
☑	73	*2201.2418*	*2200.2346*
☑	75	*2351.2742*	*2350.2669*
☑	76	*2387.3799*	*2386.3726*
☑	77	*2456.3299*	*2455.3226*
☑	78	*2464.3910*	*2463.3837*
☑	79	*2478.3526*	*2477.3453*
☑	80	*2530.3976*	*2529.3903*
☑	81	*2552.3880*	*2551.3807*
☑	82	*2591.4719*	*2590.4646*
☑	83	*2713.4115*	*2712.4043*
☑	84	*2748.4453*	*2747.4380*
☑	85	*2783.3997*	*2782.3924*
☑	86	*2790.5867*	*2789.5795*
☑	87	*2807.4466*	*2806.4393*

Search Parameters

```
Type of search         : MS/MS Ion Search
Enzyme                 : Trypsin
Fixed modifications    : Carbamidomethyl (C)
Variable modifications : Oxidation (M)
Mass values            : Monoisotopic
Protein Mass           : Unrestricted
Peptide Mass Tolerance : ± 100 ppm
Fragment Mass Tolerance: ± 0.7 Da
Max Missed Cleavages   : 2
Instrument type        : Default
Number of queries      : 87
```

Mascot: http://www.matrixscience.com/

Mascot Search Results

```
User          :
Email         :
Search title  :
MS data file  : DATA.TXT
Database      : SwissProt 2013_10 (541561 sequences; 192480382 residues)
Taxonomy      : Saccharomyces Cerevisiae (baker's yeast) (7802 sequences)
Timestamp     : 23 Nov 2016 at 12:32:43 GMT
Protein hits  : ATPA_YEAST  ATP synthase subunit alpha, mitochondrial OS=Saccharomyces cerevisiae (strain ATCC 204508 / S288c) GN=ATP1 PE=1
                ATPB_YEAST  ATP synthase subunit beta, mitochondrial OS=Saccharomyces cerevisiae (strain ATCC 204508 / S288c) GN=ATP2 PE=1 S
                ATPG_YEAST  ATP synthase subunit gamma, mitochondrial OS=Saccharomyces cerevisiae (strain ATCC 204508 / S288c) GN=ATP3 PE=1
                ATP7_YEAST  ATP synthase subunit d, mitochondrial OS=Saccharomyces cerevisiae (strain ATCC 204508 / S288c) GN=ATP7 PE=1 SV=2
                QCR1_YEAST  Cytochrome b-c1 complex subunit 1, mitochondrial OS=Saccharomyces cerevisiae (strain ATCC 204508 / S288c) GN=COR
                ATPO_YEAST  ATP synthase subunit 5, mitochondrial OS=Saccharomyces cerevisiae (strain ATCC 204508 / S288c) GN=ATP5 PE=1 SV=1
                NAM7_YEAST  ATP-dependent helicase NAM7 OS=Saccharomyces cerevisiae (strain ATCC 204508 / S288c) GN=NAM7 PE=1 SV=1
                QCR2_YEAST  Cytochrome b-c1 complex subunit 2, mitochondrial OS=Saccharomyces cerevisiae (strain ATCC 204508 / S288c) GN=QCR
                RS25A_YEAST 40S ribosomal protein S25-A OS=Saccharomyces cerevisiae (strain ATCC 204508 / S288c) GN=RPS25A PE=1 SV=1
                ATPF_YEAST  ATP synthase subunit 4, mitochondrial OS=Saccharomyces cerevisiae (strain ATCC 204508 / S288c) GN=ATP4 PE=1 SV=2
                YJ94_YEAST  Uncharacterized membrane protein YJR124C OS=Saccharomyces cerevisiae (strain ATCC 204508 / S288c) GN=YJR124C PE=
                SPT6_YEAST  Transcription elongation factor SPT6 OS=Saccharomyces cerevisiae (strain ATCC 204508 / S288c) GN=SPT6 PE=1 SV=1
                YM35_YEAST  Uncharacterized protein YMR160W OS=Saccharomyces cerevisiae (strain ATCC 204508 / S288c) GN=YMR160W PE=1 SV=1
                MLF3_YEAST  Protein MLF3 OS=Saccharomyces cerevisiae (strain ATCC 204508 / S288c) GN=MLF3 PE=1 SV=2
                YSH1_YEAST  Endoribonuclease YSH1 OS=Saccharomyces cerevisiae (strain ATCC 204508 / S288c) GN=YSH1 PE=1 SV=1
                GPI7_YEAST  GPI ethanolamine phosphate transferase 2 OS=Saccharomyces cerevisiae (strain ATCC 204508 / S288c) GN=LAS21 PE=1
                HXKG_YEAST  Glucokinase-1 OS=Saccharomyces cerevisiae (strain ATCC 204508 / S288c) GN=GLK1 PE=1 SV=1
                PRP22_YEAST Pre-mRNA-splicing factor ATP-dependent RNA helicase PRP22 OS=Saccharomyces cerevisiae (strain ATCC 204508 / S288
                NOP4_YEAST  Nucleolar protein 4 OS=Saccharomyces cerevisiae (strain ATCC 204508 / S288c) GN=NOP4 PE=1 SV=1
                MGR3L_YEAST Protein MRG3-like OS=Saccharomyces cerevisiae (strain ATCC 204508 / S288c) GN=YKL133C PE=3 SV=2
```

Mascot Score Histogram

Ions score is -10*Log(P), where P is the probability that the observed match is a random event.
Individual ions scores > 23 indicate identity or extensive homology (p<0.05).
Protein scores are derived from ions scores as a non-probabilistic basis for ranking protein hits.

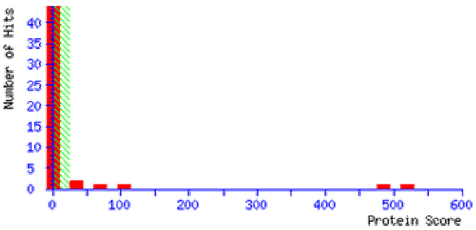

Peptide Summary Report

Format As | Peptide Summary | Help

Significance threshold p< 0.05 | Max. number of hits 20

Standard scoring ○ MudPIT scoring ◉ | Ions score or expect cut-off 0 | Show sub-sets 0

Show pop-ups ◉ Suppress pop-ups ○ | Sort unassigned Decreasing Score | Require bold red ☐

Overview Table

Click on column header to jump to entry in results list.
Move mouse over any indicator to highlight identical peptides.
Click on an indicator to see details of individual match.
Use check boxes to select sub-set of queries for new search.

Mouse over: -Query-
-Accession- -Sequence-

Hit:	1	2	3	4	5	6	7	8	9	10	11	12	13	14	15	16	17	18	19	20
☑ 1104.6534 (1+)							●				●	●	●		●	●				●
☑ 1111.7417 (1+)																				
☑ 1116.7267 (1+)		●																		
☑ 1137.6675 (1+)																				
☑ 1208.7777 (1+)																				
☑ 1227.7552 (1+)																				
☑ 1246.7945 (1+)																				
☑ 1262.7345 (1+)																				
☑ 1269.7774 (1+)																				
☑ 1273.7737 (1+)																				
☑ 1297.7167 (1+)																				
☑ 1325.7824 (1+)	●																			
☑ 1356.7607 (1+)																				
☑ 1366.8670 (1+)		●																		

☑ 1367.8611 (1+)									●											
☑ 1383.8702 (1+)																				
☑ 1388.9660 (1+)																				
☑ 1394.8149 (1+)																				
☑ 1402.8350 (1+)																				
☑ 1420.8075 (1+)		●															●			
☑ 1435.8671 (1+)		●																		
☑ 1438.9628 (1+)	●																			
☑ 1442.7891 (1+)																				
☑ 1442.9411 (1+)																				
☑ 1453.9159 (1+)																				
☑ 1458.8573 (1+)	●																			
☑ 1500.8708 (1+)										●										
☑ 1502.9674 (1+)																				
☑ 1519.8826 (1+)	●																			
☑ 1524.9529 (1+)																				
☑ 1541.8757 (1+)																				
☑ 1553.8581 (1+)	●																			
☑ 1564.0066 (1+)	●																			
☑ 1575.8404 (1+)																				
☑ 1585.9429 (1+)		●																		
☑ 1601.9367 (1+)		●												●						
☑ 1607.9274 (1+)															●					
☑ 1647.0285 (1+)																				
☑ 1660.1134 (1+)						●														
☑ 1678.0556 (1+)		●																		
☑ 1700.0337 (1+)																				
☑ 1720.1115 (1+)	●																			
☑ 1752.0710 (1+)																				
☑ 1756.9614 (1+)																				
☑ 1763.1339 (1+)		●																		
☑ 1774.0510 (1+)		●																		
☑ 1778.9494 (1+)																				
☑ 1781.1707 (1+)																				
☑ 1785.1082 (1+)																				
☑ 1789.9799 (1+)																				
☑ 1796.0377 (1+)																				
☑ 1801.0618 (1+)																				
☑ 1815.0531 (1+)		●																		
☑ 1842.0508 (1+)					●															
☑ 1857.0363 (1+)																				
☑ 1867.1383 (1+)								●												
☑ 1869.1148 (1+)																				
☑ 1874.0954 (1+)																				
☑ 1880.1715 (1+)																				
☑ 1899.0930 (1+)	●																			
☑ 1908.1535 (1+)																				
☑ 1921.0813 (1+)																				
☑ 1930.1440 (1+)																				
☑ 1935.1635 (1+)	●																			
☑ 1946.1203 (1+)																				
☑ 2005.1438 (1+)																				
☑ 2036.3179 (1+)																				
☑ 2055.2018 (1+)																				
☑ 2073.2189 (1+)	●																			
☑ 2089.2123 (1+)																				
☑ 2180.2590 (1+)																				
☑ 2190.2251 (1+)				●															●	
☑ 2202.2520 (1+)																				
☑ 2260.3106 (1+)		●																		
☑ 2276.3062 (1+)																				
☑ 2282.2992 (1+)																		●		
☑ 2298.2858 (1+)																				
☑ 2305.2968 (1+)																				
☑ 2315.3213 (1+)																				
☑ 2346.3304 (1+)																				
☑ 2351.3017 (1+)			●																	

☑ 2666.5136 (1+)																				
☑ 2748.4741 (1+)																				
☑ 2807.4779 (1+)																				
☑ 2834.5611 (1+)																				
☑ 2935.7216 (1+)		●																		
☑ 2974.6325 (1+)																				
☑ 3411.9157 (1+)	●																			

Select All | Select None | Search Selected | ☐ **Error tolerant** | Archive Report

1. ATPA_YEAST **Mass:** 58629 **Score:** 519 **Matches:** 11(11) **Sequences:** 11(11)
ATP synthase subunit alpha, mitochondrial OS=Saccharomyces cerevisiae (strain ATCC 204508 / S288c) GN=ATP1 PE=1 SV=5
☐ Check to include this hit in error tolerant search or archive report

Query	Observed	Mr(expt)	Mr(calc)	ppm	Miss	Score	Expect	Rank	Unique	Peptide
12	1325.7824	1324.7751	1324.6735	76.7	0	48	0.00024	1	U	K.SNHNELLTEIR.E
22	1438.9628	1437.9555	1437.8416	79.2	0	34	0.0011	1	U	K.GIRPAINVGLSVSR.V
26	1458.8573	1457.8501	1457.7362	78.1	0	57	3.6e-005	1	U	K.AQPTEVSSILEER.I
29	1519.8826	1518.8753	1518.7606	75.6	0	58	3.5e-005	1	U	R.IGEFESSFLSYLK.S
32	1553.8581	1552.8508	1552.7310	77.1	0	68	2.5e-006	1	U	R.EAYPGDVFYLHSR.L
33	1564.0066	1562.9993	1562.8781	77.6	0	87	1.1e-008	1	U	R.TGNIVDVPVGPGLLGR.V
42	1720.1115	1719.1043	1718.9792	72.8	1	23	0.019	1	U	K.RTGNIVDVPVGPGLLGR.V
60	1899.0930	1898.0857	1897.9568	67.9	0	53	7.3e-005	1	U	R.STVAQLVQTLEQHDAMK.Y
64	1935.1635	1934.1562	1934.0221	69.3	1	97	2.4e-009	1	U	R.VVDALGNPIDGKGPIDAAGR.S
69	2073.2189	2072.2116	2072.0725	67.2	0	59	1.4e-005	1	U	K.GMALNLEPGQVGIVLFGSDR.L
88	3411.9157	3410.9084	3410.7106	58.0	0	110	7e-011	1	U	K.QNQYSPLATEEQVPLIYAGVNGHLDGIELSR.I

2. ATPB_YEAST **Mass:** 54817 **Score:** 488 **Matches:** 12(9) **Sequences:** 11(9)
ATP synthase subunit beta, mitochondrial OS=Saccharomyces cerevisiae (strain ATCC 204508 / S288c) GN=ATP2 PE=1 SV=2
☐ Check to include this hit in error tolerant search or archive report

Query	Observed	Mr(expt)	Mr(calc)	ppm	Miss	Score	Expect	Rank	Unique	Peptide
3	1116.7267	1115.7194	1115.6339	76.7	0	53	4.2e-005	1	U	K.VVDLLAPYAR.G
14	1366.8670	1365.8597	1365.7616	71.8	0	39	0.00094	1	U	K.VLDTGGPISVPVGR.E
20	1420.8075	1419.8002	1419.6895	77.9	0	88	2.9e-008	1	U	K.AHGGFSVFTGVGER.T
21	1435.8671	1434.8598	1434.7467	78.9	0	56	3e-005	1	U	R.FTQAGSEVSALLGR.I
35	1585.9429	1584.9356	1584.8082	80.4	0	53	8.4e-005	1	U	K.VALVFGQMNEPPGAR.A
36	1601.9367	1600.9295	1600.8032	78.9	0	(23)	0.08	1	U	K.VALVFGQMNEPPGAR.A + Oxidation (M)
40	1678.0556	1677.0483	1676.9210	75.9	0	80	8.3e-008	1	U	K.LVLEVAQHLGENTVR.T
45	1763.1339	1762.1266	1761.9989	72.5	1	57	9.6e-006	1	U	R.IINVIGEPIDERGPIK.S
46	1774.0510	1773.0437	1772.9196	70.0	0	11	1.1	1	U	R.GISELGIYPAVDPLDSK.S
53	1815.0531	1814.0458	1813.9210	68.8	0	51	0.00013	1	U	R.LLDAAVVGQEHYDVASK.V
74	2260.3106	2259.3033	2259.1569	64.8	0	156	3.2e-015	1	U	R.IPSAVGYQPTLATDMGLLQER.I
86	2935.7216	2934.7143	2934.5450	57.7	1	14	0.43	1	U	K.LRKPIHADPPSFAEQSTSAEILETGIK.V

3. ATPG_YEAST **Mass:** 34443 **Score:** 114 **Matches:** 1(1) **Sequences:** 1(1)
ATP synthase subunit gamma, mitochondrial OS=Saccharomyces cerevisiae (strain ATCC 204508 / S288c) GN=ATP3 PE=1 SV=1
☐ Check to include this hit in error tolerant search or archive report

Query	Observed	Mr(expt)	Mr(calc)	ppm	Miss	Score	Expect	Rank	Unique	Peptide
81	2351.3017	2350.2944	2350.1441	64.0	1	114	7.1e-011	1	U	K.TIEQSPSFGKFEIDTDANVPR.D

4. ATP7_YEAST **Mass:** 19797 **Score:** 80 **Matches:** 1(1) **Sequences:** 1(1)
ATP synthase subunit d, mitochondrial OS=Saccharomyces cerevisiae (strain ATCC 204508 / S288c) GN=ATP7 PE=1 SV=2
☐ Check to include this hit in error tolerant search or archive report

Query	Observed	Mr(expt)	Mr(calc)	ppm	Miss	Score	Expect	Rank	Unique	Peptide
72	2190.2251	2189.2179	2189.0753	65.1	0	80	2e-007	1	U	R.QLLELQSQPTEVDFSHYR.S

5. QCR1_YEAST **Mass:** 50254 **Score:** 23 **Matches:** 1(0) **Sequences:** 1(0)
Cytochrome b-c1 complex subunit 1, mitochondrial OS=Saccharomyces cerevisiae (strain ATCC 204508 / S288c) GN=COR1 PE=1 SV=1
☐ Check to include this hit in error tolerant search or archive report

Query	Observed	Mr(expt)	Mr(calc)	ppm	Miss	Score	Expect	Rank	Unique	Peptide
54	1842.0508	1841.0435	1840.9108	72.1	0	23	0.068	1	U	K.LAAQIFGSYNAFEPASR.L

6. ATPO_YEAST **Mass:** 22857 **Score:** 20 **Matches:** 1(1) **Sequences:** 1(1)
ATP synthase subunit 5, mitochondrial OS=Saccharomyces cerevisiae (strain ATCC 204508 / S288c) GN=ATP5 PE=1 SV=1
☐ Check to include this hit in error tolerant search or archive report

Query	Observed	Mr(expt)	Mr(calc)	ppm	Miss	Score	Expect	Rank	Unique	Peptide
39	1660.1134	1659.1061	1658.9831	74.1	1	20	0.018	1	U	K.LGHLLLNPALSLKDR.N

7. NAM7_YEAST **Mass:** 110445 **Score:** 0 **Matches:** 1(0) **Sequences:** 1(0)
ATP-dependent helicase NAM7 OS=Saccharomyces cerevisiae (strain ATCC 204508 / S288c) GN=NAM7 PE=1 SV=1
☐ Check to include this hit in error tolerant search or archive report

Query	Observed	Mr(expt)	Mr(calc)	ppm	Miss	Score	Expect	Rank	Unique	Peptide
1	1104.6534	1103.6461	1103.6815	-32.08	0	14	0.76	1	U	R.LISLGHVPIR.L

8. QCR2_YEAST **Mass:** 40510 **Score:** 0 **Matches:** 1(0) **Sequences:** 1(0)
Cytochrome b-c1 complex subunit 2, mitochondrial OS=Saccharomyces cerevisiae (strain ATCC 204508 / S288c) GN=QCR2 PE=1 SV=1
Check to include this hit in error tolerant search or archive report

Query	Observed	Mr(expt)	Mr(calc)	ppm	Miss	Score	Expect	Rank	Unique	Peptide
**56**	**1867.1383**	**1866.1310**	**1865.9999**	**70.2**	**0**	**11**	**0.83**	**1**	**U**	**K.TAFKPHELTESVLPAAR.Y**

9. RS25A_YEAST **Mass:** 12032 **Score:** 0 **Matches:** 1(0) **Sequences:** 1(0)
40S ribosomal protein S25-A OS=Saccharomyces cerevisiae (strain ATCC 204508 / S288c) GN=RPS25A PE=1 SV=1
Check to include this hit in error tolerant search or archive report

Query	Observed	Mr(expt)	Mr(calc)	ppm	Miss	Score	Expect	Rank	Unique	Peptide
**15**	**1367.8611**	**1366.8538**	**1366.8772**	**-17.11**	**2**	**9**	**1.1**	**1**	**U**	**R.LKIGGSLARIALR.H**

Proteins matching the same set of peptides:
RS25B_YEAST **Mass:** 12002 **Score:** 0 **Matches:** 1(0) **Sequences:** 1(0)
40S ribosomal protein S25-B OS=Saccharomyces cerevisiae (strain ATCC 204508 / S288c) GN=RPS25B PE=1 SV=1

10. ATPF_YEAST **Mass:** 26965 **Score:** 0 **Matches:** 1(0) **Sequences:** 1(0)
ATP synthase subunit 4, mitochondrial OS=Saccharomyces cerevisiae (strain ATCC 204508 / S288c) GN=ATP4 PE=1 SV=2
Check to include this hit in error tolerant search or archive report

Query	Observed	Mr(expt)	Mr(calc)	ppm	Miss	Score	Expect	Rank	Unique	Peptide
**27**	**1500.8708**	**1499.8635**	**1499.7408**	**81.8**	**1**	**8**	**2.5**	**1**	**U**	**K.YLAPAYKDFADAR.M**

11. YJ94_YEAST **Score:** 0 **Matches:** 1(0) **Sequences:** 1(0)
Uncharacterized membrane protein YJR124C OS=Saccharomyces cerevisiae (strain ATCC 204508 / S288c) GN=YJR124C PE=1 SV=1
Check to include this hit in error tolerant search or archive report

Query	Observed	Mr(expt)	Mr(calc)	ppm	Miss	Score	Expect	Rank	Unique	Peptide
1	1104.6534	1103.6461	1103.6491	-2.75	0	8	3.2	2	U	K.LLWASVFLR.L

12. SPT6_YEAST **Score:** 0 **Matches:** 1(0) **Sequences:** 1(0)
Transcription elongation factor SPT6 OS=Saccharomyces cerevisiae (strain ATCC 204508 / S288c) GN=SPT6 PE=1 SV=1
Check to include this hit in error tolerant search or archive report

Query	Observed	Mr(expt)	Mr(calc)	ppm	Miss	Score	Expect	Rank	Unique	Peptide
1	1104.6534	1103.6461	1103.5757	63.8	0	6	4.9	3	U	K.ILSLTCGQGR.F

13. YM35_YEAST **Score:** 0 **Matches:** 1(0) **Sequences:** 1(0)
Uncharacterized protein YMR160W OS=Saccharomyces cerevisiae (strain ATCC 204508 / S288c) GN=YMR160W PE=1 SV=1
Check to include this hit in error tolerant search or archive report

Query	Observed	Mr(expt)	Mr(calc)	ppm	Miss	Score	Expect	Rank	Unique	Peptide
1	1104.6534	1103.6461	1103.5360	99.8	0	6	5	4	U	R.IAEYPNGQGR.S

14. MLF3_YEAST **Score:** 0 **Matches:** 1(0) **Sequences:** 1(0)
Protein MLF3 OS=Saccharomyces cerevisiae (strain ATCC 204508 / S288c) GN=MLF3 PE=1 SV=2
Check to include this hit in error tolerant search or archive report

Query	Observed	Mr(expt)	Mr(calc)	ppm	Miss	Score	Expect	Rank	Unique	Peptide
36	1601.9367	1600.9295	1600.8719	36.0	0	6	4.5	2	U	R.RPSSAVSLNMALLAR.T + Oxidation (M)

15. YSH1_YEAST **Score:** 0 **Matches:** 2(0) **Sequences:** 2(0)
Endoribonuclease YSH1 OS=Saccharomyces cerevisiae (strain ATCC 204508 / S288c) GN=YSH1 PE=1 SV=1
Check to include this hit in error tolerant search or archive report

Query	Observed	Mr(expt)	Mr(calc)	ppm	Miss	Score	Expect	Rank	Unique	Peptide
1	1104.6534	1103.6461	1103.6240	20.0	1	5	6.9	7	U	R.WLLRDFVR.V
37	**1607.9274**	**1606.9201**	**1606.9096**	**6.56**	**2**	**1**	**12**	**3**	**U**	**K.AIYRWLLRDFVR.V**

16. GPI7_YEAST **Score:** 0 **Matches:** 1(0) **Sequences:** 1(0)
GPI ethanolamine phosphate transferase 2 OS=Saccharomyces cerevisiae (strain ATCC 204508 / S288c) GN=LAS21 PE=1 SV=1
Check to include this hit in error tolerant search or archive report

Query	Observed	Mr(expt)	Mr(calc)	ppm	Miss	Score	Expect	Rank	Unique	Peptide
1	1104.6534	1103.6461	1103.6702	-21.87	1	6	5.4	5	U	K.VLNKNYLIK.D

17. HXKG_YEAST **Score:** 0 **Matches:** 1(0) **Sequences:** 1(0)
Glucokinase-1 OS=Saccharomyces cerevisiae (strain ATCC 204508 / S288c) GN=GLK1 PE=1 SV=1
Check to include this hit in error tolerant search or archive report

Query	Observed	Mr(expt)	Mr(calc)	ppm	Miss	Score	Expect	Rank	Unique	Peptide
20	1420.8075	1419.8002	1419.7470	37.5	0	5	5.8	2	U	R.HALALSPLGAEGER.K

18. PRP22_YEAST **Mass:** 130613 **Score:** 0 **Matches:** 1(0) **Sequences:** 1(0)
Pre-mRNA-splicing factor ATP-dependent RNA helicase PRP22 OS=Saccharomyces cerevisiae (strain ATCC 204508 / S288c) GN=PRP22 PE=1 SV=1
Check to include this hit in error tolerant search or archive report

Query	Observed	Mr(expt)	Mr(calc)	ppm	Miss	Score	Expect	Rank	Unique	Peptide
76	**2282.2992**	**2281.2919**	**2281.1756**	**51.0**	**1**	**5**	**3.8**	**1**	**U**	**K.AKFHHPYGDHLTLLNVYTR.W**

19. NOP4_YEAST **Score:** 0 **Matches:** 1(0) **Sequences:** 1(0)
Nucleolar protein 4 OS=Saccharomyces cerevisiae (strain ATCC 204508 / S288c) GN=NOP4 PE=1 SV=1
☐ Check to include this hit in error tolerant search or archive report

Query	Observed	Mr(expt)	Mr(calc)	ppm	Miss	Score	Expect	Rank	Unique	Peptide
72	2190.2251	2189.2179	2189.0344	83.8	2	5	5.8	2	U	K.MADLLTNTDMEIREKSYK.L + 2 Oxidation (M)

20. MGR3L_YEAST **Score:** 0 **Matches:** 1(0) **Sequences:** 1(0)
Protein MRG3-like OS=Saccharomyces cerevisiae (strain ATCC 204508 / S288c) GN=YKL133C PE=3 SV=2
☐ Check to include this hit in error tolerant search or archive report

Query	Observed	Mr(expt)	Mr(calc)	ppm	Miss	Score	Expect	Rank	Unique	Peptide
1	1104.6534	1103.6461	1103.6121	30.8	1	5	6.7	6	U	K.AIRISEMIR.F + Oxidation (M)

Peptide matches not assigned to protein hits: (no details means no match)

	Query	Observed	Mr(expt)	Mr(calc)	ppm	Miss	Score	Expect	Rank	Unique	Peptide
☑	37	1607.9274	1606.9201	1606.8427	48.2	0	4	5.7	1		ITQVLAQNNEVHNK
☑	59	**1880.1715**	**1879.1642**	**1879.0315**	**70.6**	**1**	**3**	**4.4**	**1**		**KAGISYAAYLNVAAQAIR**
☑	23	**1442.7891**	**1441.7819**	**1441.6831**	**68.5**	**1**	**3**	**9.1**	**1**		**RMSSISSISSESR + Oxidation (M)**
☑	38	**1647.0285**	**1646.0212**	**1645.9589**	**37.9**	**2**	**1**	**6.8**	**1**		**IVIGLYGKVCPKTAK**
☑	24	**1442.9411**	**1441.9338**	**1441.8405**	**64.7**	**1**	**0**	**6**	**1**		**LPHIPAGSNPLKAK**
☑	34	**1575.8404**	**1574.8331**	**1574.7875**	**29.0**	**2**	**0**	**20**	**1**		**MAKDLNDSGFPPKR**
☑	2	*1111.7417*	*1110.7344*								
☑	4	*1137.6675*	*1136.6602*								
☑	5	*1208.7777*	*1207.7704*								
☑	6	*1227.7552*	*1226.7479*								
☑	7	*1246.7945*	*1245.7872*								
☑	8	*1262.7345*	*1261.7272*								
☑	9	*1269.7774*	*1268.7701*								
☑	10	*1273.7737*	*1272.7664*								
☑	11	*1297.7167*	*1296.7094*								
☑	13	*1356.7607*	*1355.7534*								
☑	16	*1383.8702*	*1382.8629*								
☑	17	*1388.9660*	*1387.9587*								
☑	18	*1394.8149*	*1393.8076*								
☑	19	*1402.8350*	*1401.8277*								
☑	25	*1453.9159*	*1452.9086*								
☑	28	*1502.9674*	*1501.9602*								
☑	30	*1524.9529*	*1523.9456*								
☑	31	*1541.8757*	*1540.8684*								
☑	41	*1700.0337*	*1699.0264*								
☑	43	*1752.0710*	*1751.0637*								
☑	44	*1756.9614*	*1755.9541*								
☑	47	*1778.9494*	*1777.9422*								
☑	48	*1781.1707*	*1780.1635*								
☑	49	*1785.1082*	*1784.1009*								
☑	50	*1789.9799*	*1788.9726*								
☑	51	*1796.0377*	*1795.0304*								
☑	52	*1801.0618*	*1800.0545*								
☑	55	*1857.0363*	*1856.0291*								
☑	57	*1869.1148*	*1868.1075*								
☑	58	*1874.0954*	*1873.0882*								
☑	61	*1908.1535*	*1907.1462*								
☑	62	*1921.0813*	*1920.0741*								
☑	63	*1930.1440*	*1929.1367*								
☑	65	*1946.1203*	*1945.1130*								
☑	66	*2005.1438*	*2004.1365*								
☑	67	*2036.3179*	*2035.3106*								
☑	68	*2055.2018*	*2054.1945*								
☑	70	*2089.2123*	*2088.2050*								
☑	71	*2180.2590*	*2179.2517*								
☑	73	*2202.2520*	*2201.2447*								
☑	75	*2276.3062*	*2275.2989*								
☑	77	*2298.2858*	*2297.2786*								
☑	78	*2305.2968*	*2304.2895*								
☑	79	*2315.3213*	*2314.3140*								
☑	80	*2346.3304*	*2345.3231*								
☑	82	*2666.5136*	*2665.5064*								
☑	83	*2748.4741*	*2747.4668*								
☑	84	*2807.4779*	*2806.4706*								
☑	85	*2834.5611*	*2833.5538*								
☑	87	*2974.6325*	*2973.6253*								

Search Parameters

Type of search : **MS/MS Ion Search**
Enzyme : **Trypsin**

```
Fixed modifications    : Carbamidomethyl (C)
Variable modifications : Oxidation (M)
Mass values            : Monoisotopic
Protein Mass           : Unrestricted
Peptide Mass Tolerance : ± 100 ppm
Fragment Mass Tolerance: ± 0.7 Da
Max Missed Cleavages   : 2
Instrument type        : Default
Number of queries      : 88
```

**Mascot:** http://www.matrixscience.com/
